# LRTK: a platform agnostic toolkit for linked-read analysis of both human genome and metagenome

**DOI:** 10.1093/gigascience/giae028

**Published:** 2024-06-13

**Authors:** Chao Yang, Zhenmiao Zhang, Yufen Huang, Xuefeng Xie, Herui Liao, Jin Xiao, Werner Pieter Veldsman, Kejing Yin, Xiaodong Fang, Lu Zhang

**Affiliations:** Department of Computer Science, Hong Kong Baptist University, Hong Kong SAR 999077, Hong Kong; Department of Computer Science, Hong Kong Baptist University, Hong Kong SAR 999077, Hong Kong; BGI Research, Shenzhen 518083, China; BGI Genomics, Shenzhen 518083, China; BGI Research, Sanya 572025, China; Department of Electrical Engineering, City University of Hong Kong, Hong Kong SAR 999077, Hong Kong; Department of Computer Science, Hong Kong Baptist University, Hong Kong SAR 999077, Hong Kong; Department of Computer Science, Hong Kong Baptist University, Hong Kong SAR 999077, Hong Kong; Department of Computer Science, Hong Kong Baptist University, Hong Kong SAR 999077, Hong Kong; BGI Genomics, Shenzhen 518083, China; BGI Research, Sanya 572025, China; Department of Computer Science, Hong Kong Baptist University, Hong Kong SAR 999077, Hong Kong; Institute for Research and Continuing Education, Hong Kong Baptist University, Hong Kong SAR 999077, Hong Kong

**Keywords:** linked-read sequencing, 10x Genomics, TELL-Seq, stLFR, metagenome, human genome

## Abstract

**Background:**

Linked-read sequencing technologies generate high-base quality short reads that contain extrapolative information on long-range DNA connectedness. These advantages of linked-read technologies are well known and have been demonstrated in many human genomic and metagenomic studies. However, existing linked-read analysis pipelines (e.g., Long Ranger) were primarily developed to process sequencing data from the human genome and are not suited for analyzing metagenomic sequencing data. Moreover, linked-read analysis pipelines are typically limited to 1 specific sequencing platform.

**Findings:**

To address these limitations, we present the Linked-Read ToolKit (LRTK), a unified and versatile toolkit for platform agnostic processing of linked-read sequencing data from both human genome and metagenome. LRTK provides functions to perform linked-read simulation, barcode sequencing error correction, barcode-aware read alignment and metagenome assembly, reconstruction of long DNA fragments, taxonomic classification and quantification, and barcode-assisted genomic variant calling and phasing. LRTK has the ability to process multiple samples automatically and provides users with the option to generate reproducible reports during processing of raw sequencing data and at multiple checkpoints throughout downstream analysis. We applied LRTK on linked reads from simulation, mock community, and real datasets for both human genome and metagenome. We showcased LRTK’s ability to generate comparative performance results from preceding benchmark studies and to report these results in publication-ready HTML document plots.

**Conclusions:**

LRTK provides comprehensive and flexible modules along with an easy-to-use Python-based workflow for processing linked-read sequencing datasets, thereby filling the current gap in the field caused by platform-centric genome-specific linked-read data analysis tools.

## Introduction

Linked-read sequencing generates short reads with high base quality and extrapolative information on long-range DNA connectedness, which has led to significant advancements in human genome and metagenome research [1–4]. It circumvents the typical lack of long-range DNA information in short-read sequencing and the high error rates and large initial DNA load requirements of long-read sequencing (e.g., Oxford Nanopore and Pacific Bioscience). These advantages of linked-read sequencing are invaluable when dealing with challenging cases of low-input clinical samples, such as cancer tissues or infectious disease samples. Linked-read technology furthermore promotes haplotype construction and the detection of complex structural variations [5], and its relatively low cost enables the application in large cohort studies.

Linked-read sequencing platforms, such as 10x Genomics linked read (10x Genomics; now discontinued) and the newly developed single-tube long fragment read (stLFR) [6] and transposase enzyme-linked long-read sequencing (TELL-seq) [7], hold much promise in the metagenomics area. The hidden long-range information they provide enables local assembly of co-barcoded reads and thus significantly increases the number of high-quality metagenome-assembled genomes (MAGs) [8]. In longitudinal sequencing datasets [3, 9, 10], the barcodes associated with linked reads promote the phasing of genomic variants and refine the identification of intrahost evolution of gut microbiota. In some complex environments, such as soil, linked-read sequencing has been shown to aid in the investigation of the involved microbial genomes [11, 12]. However, existing linked-read pipelines are mainly designed for use with the human genome, which points to an urgent need for appropriate metagenome analysis toolkits.

Despite the limitations that genome specificity places on research scope, linked-read sequencing has already been successfully applied to many human genomic studies [2, 13–15]. Some toolkits have been developed to facilitate the investigation of the human genome. For example, Long Ranger [16] performs barcode-aware read alignment and implements modules for genomic variant calling and phasing using 10x Genomics linked reads. Tell-Sort [7] is a Docker-based pipeline to process TELL-seq linked reads for genomic variants detection and phasing. stLFR has found application in a customized pipeline that has been developed to first convert its raw reads into a 10x-compatible format, after which Long Ranger is applied for downstream analysis. This pipeline, however, typically requires a lot of random-access memory, and its data format conversion procedure is time-consuming. In addition, the format conversion may induce the loss of barcode specificity as the type of barcodes decreases dramatically. This occurs when the stLFR and TELL-seq linked reads are typically converted into a 10x-compatible format to run Long Ranger and Supernova [17, 18]. Beyond the preceding examples that validate the inherent usefulness of linked-read technologies, our search of the literature furthermore revealed a lack of unified and open-source toolkits that are compatible with the different linked-read platforms.

To this end, we present Linked-Read ToolKit (LRTK), a unified and versatile toolkit to analyze both metagenomic and human genome linked-read sequencing data derived from any of the 3 major linked-read sequencing platforms. LRTK delivers a suite of utilities to simulate linked-read sequencing data, barcode sequencing error correction, barcode-aware read alignment and metagenome assembly, reconstruction of long DNA fragments, and barcode-assisted genomic variant detection and phasing. LRTK is open-source, automatically produces HTML reports to summarize quality statistics as part of its pipeline, and generates publication-ready visualizations. We applied LRTK to linked reads from simulation, mock community, and real datasets to evaluate the performance of different technologies and demonstrate the potential applications of LRTK. Our results show that LRTK performs favorably on human genome linked-read sequencing data when compared to the pipelines designed specifically for a single platform and that it adequately allows for data analysis of metagenomic sequencing data.

## Results

### Overview of LRTK

**Figure 1:**
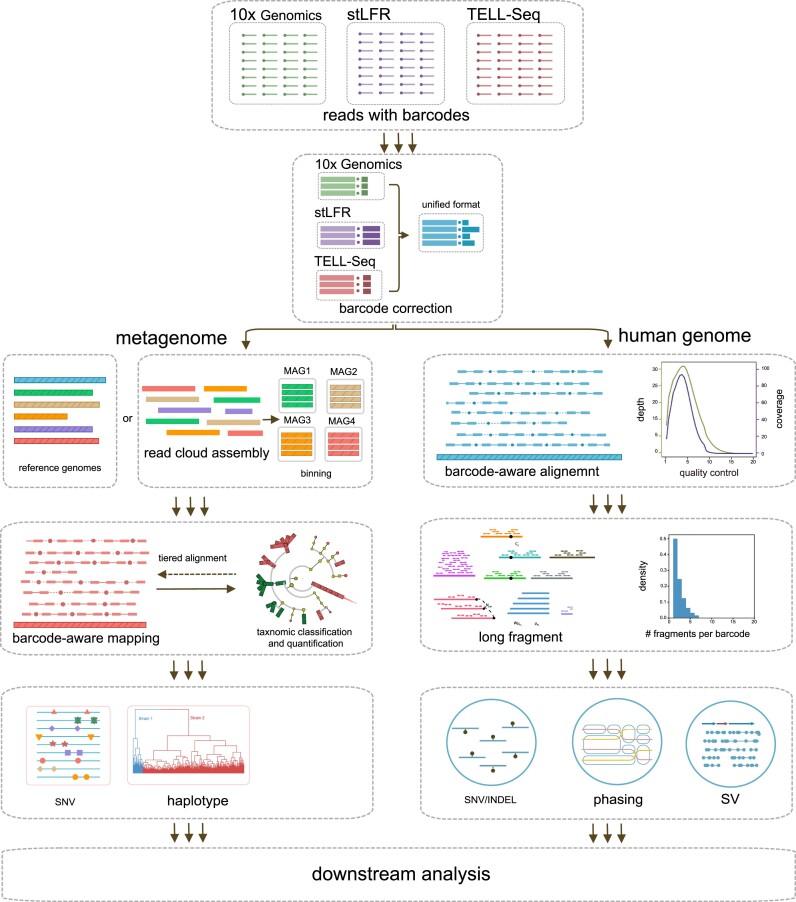
Overview of LRTK. The LRTK workflow includes a metagenomic section (left panel) and human genomic section (right panel). The metagenomic section implements barcode correction, barcode-aware alignment and metagenome assembly, long DNA fragment reconstruction, taxonomic classification and quantification, and SNV detection and phasing. The human genomic section implements barcode correction, barcode-aware alignment, long DNA fragment reconstruction, and detection and phasing of SNVs, INDELs, and SVs.

We developed Linked-Read ToolKit (which we refer to as LRTK) that takes raw linked-reads from 10x Genomics, stLFR, or TELL-seq technologies and analyzes these inputs in a multistep checkpointed pipeline that ends with the generation of user-friendly reports. LRTK consists of 2 main sections to process metagenomic and human genome linked-read sequencing data from mainstream technologies (Fig. 1). For metagenomic sequencing, LRTK includes the representative human gut microbial genomes from the UHGG [19] project as its default reference genomes. We modified EMA [20] to perform barcode-aware alignment to be compatible with different platforms. To further reduce spurious mapping errors, LRTK eliminates the genomes if their coverage is below 40%. It performs genomic variation calling and phasing for these candidate genomes if the single nucleotide variant (SNV) calling function is enabled. In addition, LRTK is equipped with the functions to perform barcode-aware metagenome assembly and reconstruct MAGs from the linked-read metagenomic sequencing data.

In the human genome processing section, LRTK directly aligns linked reads to the human reference genome using the same modified EMA followed by marking PCR duplicates for each barcode. LRTK reconstructs long DNA fragments through greedy extension based on the alignment coordinates of co-barcoded linked reads [21]. After read alignment, LRTK offers users an option to select one of the well-known tools for variant calling, including FreeBayes [22], SAMtools [23], and GATK [24] for SNV and small insertion and deletion (INDEL; <50 bps) calls and Aquila [25], LinkedSV [26], and VALOR2 [27] for structural variant (SV) calls (>50 bps). For variant phasing, LRTK utilizes HapCUT2 [28] and WhatsHap [29] to explore phasing blocks for SNVs and small INDELs (Supplementary Table S1). Further details are available in the Methods section.

### Data description

We incorporated 1 simulated dataset (S1), 1 dataset from a mock community (B1), and 2 real datasets from human gut microbiomes (D1 and D2) to evaluate the metagenomic sequencing analysis module of LRTK (Supplementary Table S2). For dataset S1, we simulated 11.9 Gb and 9.2 Gb linked reads from 10x Genomics and stLFR for 40 complete bacterial genomes from the NCBI RefSeq database, with lognormal abundance distribution (Methods), respectively (Supplementary Table S3). The dataset B1 was generated from ATCC-MSA-1003, containing 20 bacterial species with abundances varying from 0.02% to 18% (Supplementary Table S4). For the 2 real metagenomic datasets, D1 consists of 16 longitudinal human gut metagenomic sequencing datasets from a single individual, sequenced with an average of 24 Gb linked reads on a 10x Genomics platform [3]. The other dataset D2 contains around 99 Gb stLFR linked reads from the human gut metagenome [30]. For the human genome analysis section, we collected the linked-read sequencing datasets from NA12878, NA24143, NA24149, and NA24385 (Supplementary Table S2). In addition, we performed linked-reads down-sampling to ensure fair comparisons among the 3 sequencing technologies. We down-sampled around 20 Gb metagenomic linked reads from ATCC-MSA-1003 for each platform. Similarly, around 110 Gb (∼35×) linked reads from NA12878 were extracted for the 3 technologies (Supplementary Table S3).

### LRTK supports multiple linked-read sequencing technologies

LRTK can handle linked reads from various sequencing technologies, including but not limited to 10x Genomics, stLFR, or TELL-seq. Initially, LRTK converts the raw linked reads into a unified FASTQ format, which contains a new field “BX:Z:” to include 16 bps (10x Genomics linked reads), 18 bps (TELL-seq), and 30 bps (stLFR) barcode sequences (Supplementary Fig. S1).

Sequencing errors are often enriched at the start or end of linked reads, where barcode sequences are typically found. LRTK includes functions to correct potential sequencing errors in barcodes. After error correction, there are approximately 94.8% and 94.1% of barcode sequences on the whitelist of 10x Genomics linked reads for NA12878 and ATCC-MSA-1003 (Supplementary Table S2), respectively. The performance is comparable to the results obtained from Long Ranger (94.4% for NA12878 and 93.6% for ATCC-MSA-1003). The corresponding rates are slightly lower for stLFR linked reads (85.4% for NA12878 and 90.7% for ATCC-MSA-1003) compared to 10x Genomics linked reads. As there is no whitelist for TELL-seq, we could not perform the analysis for NA12878 and ATCC-MSA-1003 TELL-seq linked-read sequencing data.

### LRTK reconstructs long DNA fragments by barcode deconvolution

The quality of DNA sequencing library may significantly affect the performance of metagenome assembly [31], human genome assembly [21], and structural variant calling [32]. To evaluate the quality of linked-read sequencing libraries, we reconstructed the input long DNA fragments for both human genome and metagenomic sequencing data based on co-barcoded read alignments (Methods). We also calculated several key statistics to comprehensively compare the libraries from different linked-read sequencing technologies [21]. The statistics include average coverage of short reads per fragment (C_R_), average physical coverage of the genome by long DNA fragments (C_F_), number of fragments per partition/beads (N_F/P_), and unweighted and length-weighted average DNA fragment length (μ_FL_ and Wμ_FL_) (Supplementary Fig. S2).

**Figure 2:**
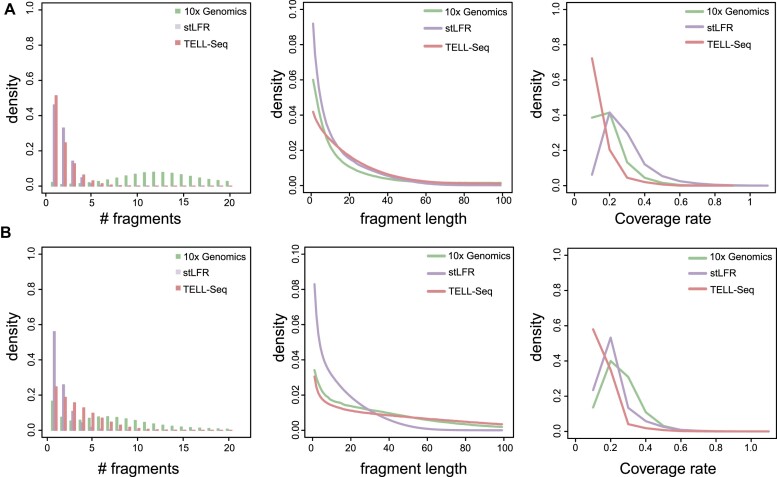
Distribution of quality metrics for different linked-read sequencing platforms. (A) Metagenome sequencing. (B) Human genome sequencing. The left panel displays the distribution of the number of long DNA fragments per barcode. The middle panel shows the length distributions of reconstructed long DNA fragments. The right panel shows the distribution of short-read coverage of fragments.

For NA12878 (Supplementary Table S3), LRTK detected approximately 7.34, 1.95, and 3.60 fragments per barcode and achieved μ_FL_ of 45.9 kb, 16.46 kb, and 54.78 kb for the libraries from 10x Genomics, stLFR, and TELL-seq, respectively (Fig. 2B). For ATCC-MSA-1003 (Supplementary Table S3), stLFR linked reads yielded the lowest N_F/P_ (N_F/P_ = 1.48), while TELL-seq linked-reads yielded a slightly higher number (N_F/P_ = 4.59). Both values were much lower than that obtained from 10x Genomics (N_F/P_ = 13.6) (Fig. 2A), indicating that stLFR and TELL-seq have superior performance in deconvolving linked reads from different species.

### LRTK enables metagenome taxonomic quantification and genomic variant detection using barcode-aware alignment

**Figure 3:**
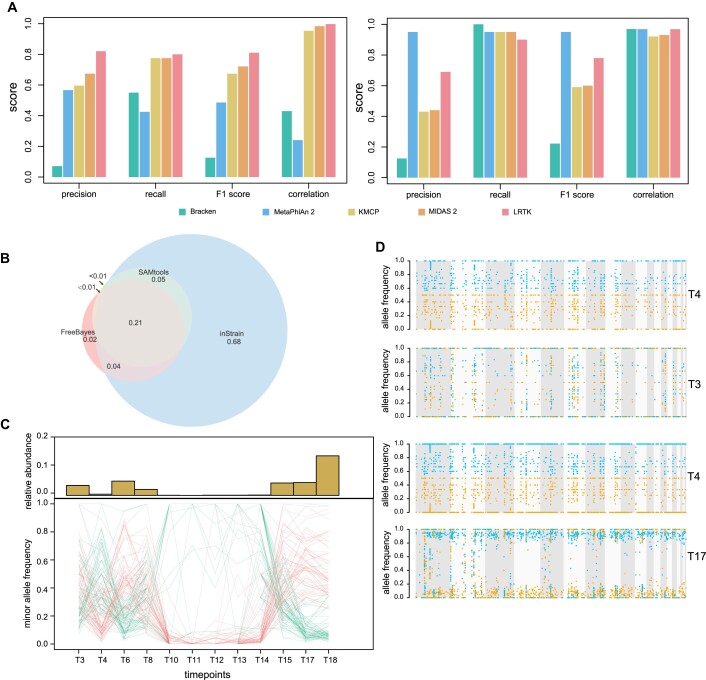
Comparison of linked read–based metagenomic quantification, SNV identification, and phasing. (A) Evaluation of tools to quantify taxonomic abundance based on the linked reads from the simulated dataset (left panel) and ATCC-MSA-1003 (right panel). (B) Comparing the performance of SAMtools, FreeBayes, and inStrain to detect metagenomic SNVs. (C) Dynamic changes of taxonomic abundance and allele frequency. (D) SNV-based strain phasing in pairwise samples. The right label shows the sample name in D1.

Previous studies have amply demonstrated the huge potential of linked reads in metagenomic studies [3, 8, 9, 11]. Here, we evaluated the performance of linked reads in detecting taxonomic abundance and genomic variants using LRTK. In LRTK, we developed a computational pipeline to detect and quantify microbes using microbial reference genomes. LRTK can better identify the involved microbes based on their genome coverage (F1 = 0.81) than the existing *k*-mer–based tools (Bracken [33] [F1 = 0.13] and KMCP [34] [F1 = 0.67]) and marker gene–based tools (MetaPhlAn2 [35] [F1 = 0.49] and MIDAS 2 [36] [F1 = 0.72]) on the simulated stLFR linked reads (Fig. 3A). For the stLFR linked reads from ATCC-MSA-1003, LRTK also demonstrated a superior performance (F1 = 0.78) compared with Bracken (F1 = 0.22), KMCP (F1 = 0.59), and MIDAS 2 (F1 = 0.6) but was inferior to MetaPhlAn 2 (F1 = 0.95). We also evaluated the LRTK performance of taxonomic quantification by comparing the benchmark microbial abundance (from simulation and ATCC-MSA-1003) and the predicted values using Spearman correlation coefficient (SCC). For the simulated dataset, LRTK (SCC = 0.99), MIDAS 2 (SCC = 0.98), and KMCP (SCC = 0.95) exhibited superior performance. For stLFR linked reads in ATCC-MSA-1003, LRTK (SCC = 0.97), Bracken (SCC = 0.97), and MetaPhlAn 2 (SCC = 0.97) were the top performers. Comparable findings were also observed in the linked-reads data generated from other technologies in simulation (Supplementary Fig. S3A) and ATCC-MSA-1003 (Supplementary Fig. S3B, C). In addition, LRTK also enables the identification of microbial SNVs and could reconstruct their potential haplotypes based on co-barcoded linked reads. Comparing different SNV callers implemented in LRTK, we discovered that approximately 176,891 SNVs were jointly detected by FreeBayes [22], SAMtools [23], and inStrain [37] on stLFR linked reads for ATCC-MSA-1003 (Fig. 3B). These SNVs account for around 76%, 80%, and 21% of the total SNVs detected by FreeBayes and SAMtools and inStrain, respectively (Fig. 3B). More than 50% of SNVs from inStrain cannot be detected by FreeBayes and SAMtools, suggesting inStrain is able to detect many unique microbial SNVs. We further applied LRTK to explore the taxonomic composition and genomic variants for each sample from a longitudinal linked-read metagenomic dataset (D1, Supplementary Table S2) [3]. Using multiple related samples, LRTK identified a strain frequency change (Methods) of the species *Alistipes finegoldii*: the minor alleles of some SNVs in some time points became the major alleles in other time points, which may suggest intrahost strain evolution over time (Fig. 3C, D).

### LRTK promotes metagenome assembly using linked reads with high barcode specificity

**Figure 4:**
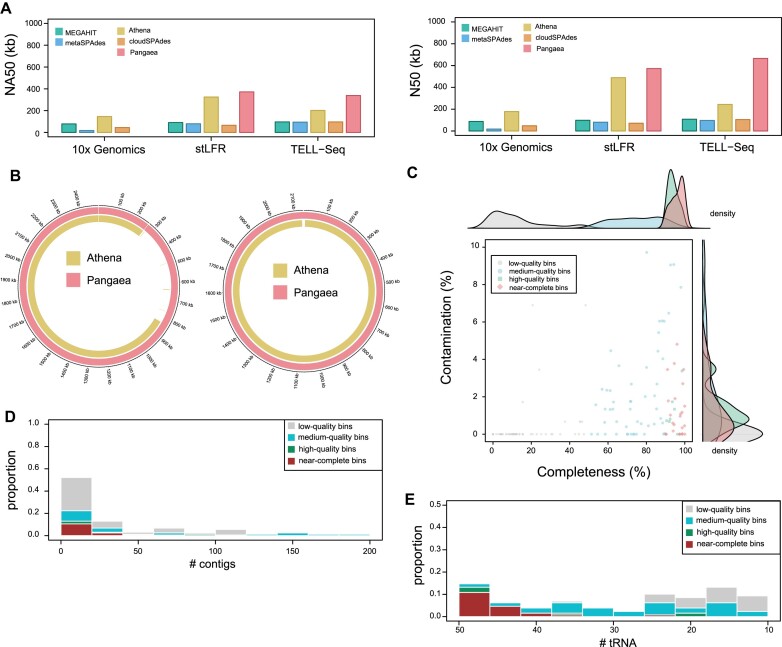
Evaluation of metagenome assemblies on linked-read sequencing. (A) Evaluation of the assembly performance for MEGAHIT, metaSPAdes, Athena, cloudSPAdes, and Pangaea on 10x Genomics, stLFR, and TELL-seq linked-read sequencing data from ATCC-MSA-1003. Pangaea does not support 10x Genomics linked reads. The left panel demonstrates the calculated NA50 values while the right panel shows the calculated N50 values. (B) Illustration of the 2 assembled circular contigs. (C) The distribution of completeness and contamination for reconstructed bins. (D) The number of contigs in each contig group. (E) The number of detected transfer RNAs in each contig group.

In our previous study, we showed that metagenome assembly on linked reads could improve assembly length and the number of near-complete MAGs [30]. We compared the performance of 2 well-known short-read assemblers, MEGAHIT [38] and metaSPAdes [39], and 3 linked-read assemblers, Athena [40], CloudSPAdes [41], and Pangaea [30], using the linked reads from ATCC-MSA-1003 and simulation (Supplementary Table S3). Among them, LRTK (Pangaea module) achieves the highest NA50 values for stLFR (NA50 = 372 kb) and TELL-seq (NA50 = 339 kb) linked reads, respectively (Fig. 4A). As Pangaea is not compatible with 10x Genomics linked reads, Athena becomes the best tool on 10x Genomics in terms of NA50 (Athena: 146 kb; cloudSPAdes: 45 kb; metaSPAdes: 17 kb; MEGAHIT: 79 kb). We also examined the assembly quality of each species in ATCC-MSA-1003 and observed that Pangaea always obtained the highest NA50 and N50 values for stLFR and TELL-seq linked reads while the assembly length is comparable (Supplementary Fig. S4A). For simulated linked reads, Pangaea and Athena also show superior performances compared with the other metagenome assemblers (Supplementary Fig. S4B and Supplementary Fig. S5). We applied LRTK to a human gut metagenomic dataset (D2) [30] and found 2 contigs were circularized, which showed near-perfect collinearity with the closest reference genomes (Fig. 4B). LRTK could also automatically perform contig binning using MetaBAT 2 [42] after metagenome assembly. In the D2 dataset, LRTK recovers 24 near-complete, 7 high-quality, and 52 medium-quality bins for D2 (Methods, Fig. 4C–E). The superior assembly performance we have observed affirms the efficacy of linked-read sequencing technologies on metagenome assembly.

### LRTK provides best practices for human genomic variant detection and phasing

**Figure 5:**
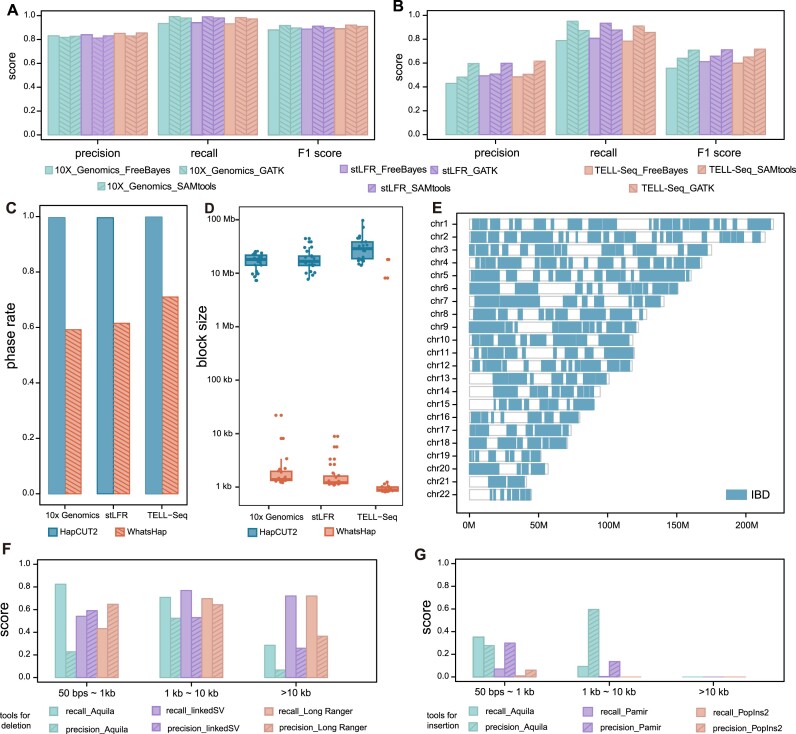
Evaluation of linked-read based detection of variation in the human genome. (A, B) Performance metrics on the detection of SNVs and INDELs using FreeBayes, GATK, and SAMtools for 10x Genomics, stLFR, and TELL-seq. (C, D) The performance on phasing of small variants using HapCUT2 and WhatsHap for 10x Genomics, stLFR, and TELL-seq. (E) Illustration of the performance on IBD detection. (F) The performance on detection of deletions using Aquila, LinkedSV, and LongRanger. (G) The performance on detection of insertions using Aquila, Pamir, and PopIns2.

Previous studies have shown that genomic variant detection [43] and phasing could benefit from the high base quality and long DNA fragments provided by linked reads [44]. Here, we benchmarked the computational tools for human genomic variant detection and phasing and demonstrated a best practice guideline for human genome linked-read analysis using LRTK. We applied the modified version of EMA to align linked reads from different sequencing technologies to the human reference genome (GRCh38). We first benchmarked the commonly used tools FreeBayes [22], GATK [24], and SAMtools [23] to detect SNVs and small INDELs. Among them, GATK (F1 = 0.90) achieves the best average F1 score across the 3 technologies in detecting SNVs, followed by SAMtools (F1 = 0.89) and FreeBayes (F1 = 0.87) (Fig. 5A). For small INDEL calling, SAMtools demonstrated the best average F1 score (SAMtools: F1 = 0.71; GATK: F1 = 0.65; FreeBayes: F1 = 0.59) while GATK had a better recall value (SAMtools: average recall = 0.87; GATK: average recall = 0.91; FreeBayes: average recall = 0.79) (Fig. 5B). We then compared the linked-read phasing tools, HapCUT2 [28] and WhatsHap [29], for genomic variant phasing. We observed that HapCUT2 (Fig. 5C, D) achieved longer average length of phasing blocks (HapCUT2: 23.2 Mb and WhatsHap: 0.4 Mb) and a higher average phased heterozygous SNV rate compared to WhatsHap (HapCUT2: 0.99 and WhatsHap: 0.63). We only evaluated the performance of SV detection tools on 10x Genomics linked reads of NA24385 because some of the tools do not support stLFR and TELL-seq. As the recall values illustrated in Fig. 5F, Aquila has a higher recall value for 50 bps—1 kb deletions (Aquila: 0.82; LinkedSV: 0.54; Long Ranger: 0.43). LinkedSV [26] and Long Ranger perform better in detecting deletions longer than 1 kb (Aquila: 0.29; LinkedSV: 0.71; Long Ranger: 0.72). Aquila also shows a better performance than Pamir [45] and PopIns2 [46] in detecting insertions (Aquila: 0.35; Pamir: 0.07; PopIns2: 0.01) (Fig. 5G). We also applied LRTK to linked reads from a trio (D3; Father: NA24149; Mother: NA24143; and Son: NA24385) and found it demonstrated excellent variant phasing performance and identity-by-descent segment detection in pairwise samples (Fig. 5E).

### LRTK provides flexible commands to process sequencing data

A primary advantage of LRTK is its flexible, user-defined settings for different tasks. Users have a choice to run each LRTK module independently and generate separate results for each module. For instance, the MKFQ function could be independently used to simulate linked reads from 10x Genomics and stLFR platforms. For some functions, LRTK provides multiple tools for users to choose from. For microbial SNV detection, LRTK offers users a choice between SAMtools [23], FreeBayes [22], and inStrain [37]. To accommodate user-specific requirements on LRTK command, LRTK also allows users to set different parameters and save the results for further comparison.

### LRTK provides automated analysis and user-friendly reports

LRTK provides an automated analysis pipeline, starting from raw linked reads to performing diverse data analysis and generating publication-ready visualizations. Specifically, LRTK investigates different types of data features, calculates their corresponding statistical indicators, and presents them together in an HTML report. Taking the aforementioned longitudinal linked-read sequencing dataset D1 as an example, LRTK generates a systematic summary of the input sequencing libraries and analysis outcomes of each step. First, LRTK produces summaries of the FASTQ files obtained from the read quality control tools (Supplementary Fig. S6A, -B). After aligning reads to the reference genomes, LRTK calculates the key parameters for the sequencing libraries and reconstructs long DNA fragments (Supplementary Fig. S6C, D). For microbial species and genomic variants, LRTK generates basic statistics and presents them using concise distribution plots (Supplementary Fig. S6E, F). For downstream analysis, LRTK conducts principal components analysis on the relative abundance profiles from multiple samples and performs clustering analysis on allele frequencies of microbial SNVs (Supplementary Fig. S6G). Similar reports could be produced for human genome analysis using LRTK (Supplementary Fig. S7).

### Evaluation of the computational resources required for LRTK

We would focus on evaluating computational resources required by LRTK for linked-read preprocessing and alignment. The computational resources needed for genomic variants calling and phasing depend on the chosen software, while metagenome sequencing data typically demand fewer computational resources due to the lower volume of required sequencing reads. We extracted approximately the same data volume (around 35×, 110 G bases) of linked reads from NA12878 for 10x Genomics, stLFR, and TELL-seq and aligned these reads to the human reference genomes. As shown in Supplementary Fig. S8, LRTK required around 26.3, 37.6, and 19.8 hours to align reads from the 3 platforms with a maximum memory usage of around 74 Gb using 64 threads. In comparison to Long Ranger, LRTK reduced memory requirements (maximum memory for Long Ranger: >100 Gb) at the expense of increased running time. The experiments were carried out on the computational nodes with Xeon Gold 6330 @ 2.0 GHz (2S/28C)/1T RAM/900 GB SWAP.

## Discussion

Seeing that multiple linked-read sequencing technologies are extensively utilized in scientific studies, a platform-agnostic linked-read processing tool would be an intuitive solution to ensure reproducibility and robustness. Unfortunately, a cross-platform software solution is currently unavailable to the research community. Accordingly, we introduce LRTK, a unified and versatile computational framework to efficiently process sequencing data from 10x Genomics, stLFR, and TELL-seq technologies. LRTK includes separately invokable commands to perform linked-read simulation, barcode sequencing error correction, barcode-aware alignment and metagenome assembly, reconstruction of long DNA fragments, and other barcode-assisted genomic variant calling and phasing. LRTK also provides automated and complete analysis, from raw data quality control through advanced downstream analysis to generation of publication-ready visualization. LRTK is also open source and allows easy integration with other scientific pipelines.

Short-read sequencing has led to significant discoveries in large-scale population sequencing studies within the human genome and metagenome fields [47, 48]. However, the limited sequencing length poses challenges for tasks like complex structural variant detection in the human genome and ribosomal RNA assembly from metagenomic data. Long-read sequencing, such as single-molecule real-time sequencing by Pacific Biosciences (PacBio) and nanopore sequencing by Oxford Nanopore Technologies (ONT), is gaining attention for their improved performance in addressing these challenges. Despite their advantages, long-read sequencing can be costly for large-cohort studies. Linked-read sequencing technologies offer a cost-effective solution for large population studies by attaching barcodes to short reads to establish long-range DNA connectedness. Previous studies have shown 10x Genomics linked reads have facilitated the discovery of complex structural variants, such as chromothripsis [5], large rearrangements [49], and tandem duplications [14], in cancer studies. Additionally, 10x Genomics linked reads have enhanced metagenome assembly contiguity [40] and enabled haplotype construction from time-series metagenomic data [3]. The newly emerging stLFR and TELL-seq have further improved barcode specificity aiming for 1 fragment per barcode. These advancements have shown superior performance in distinguishing linked reads from different species [30]. Our study benchmarks tools developed for linked-read sequencing and integrates them into LRTK to support academic applications. Furthermore, LRTK is the only tool that can accept linked reads from all 3 platforms and avoid loss of barcode specificity.

Our finding revealed that there was a consistent decrease of N_F/P_ for stLFR and TELL-seq technologies compared with those obtained from 10x Genomics. For human genome sequencing, the average N_F/P_ values were approximately 7 for 10x Genomics linked reads and declined to around 2 and 4 for stLFR and TELL-seq sequencing, respectively (Fig. 2B). For metagenomic sequencing, the average values were around 14 for 10x Genomics linked reads, 1 for stLFR, and 5 for TELL-seq sequencing (Fig. 2A). These differences may be attributed to the barcoding approaches employed. In the case of 10x Genomics, barcoding takes place within water-in-oil droplets, which require a specialized instrument for droplet generation. The number of DNA fragments present in each droplet is influenced by the size of the DNA fragments. The DNA fragments from the human genome are long, resulting in fewer fragments being included in each droplet. The microbial DNA fragments are relatively shorter, allowing for more fragments to be accommodated within a droplet. In contrast, neither TELL-seq nor stLFR utilizes droplets for barcoding. Instead, the barcoding reactions occur in an open environment and are partitioned using beads alone. Generally, each bead is conjugated with at least 1 unique barcode sequence on its surface and can capture 1–2 DNA fragments. For stLFR and TELL-seq, the number of DNA fragments is not dependent on the length of the DNA fragments, resulting in a similar number of fragments per barcode for both human and metagenomic sequencing. According to Bishara et al. [40], assembly using 10x Genomics linked reads often struggles with high-copy genomic repeat regions due to the many long fragments per barcode. The long-fragment barcoding approaches adopted by stLFR and TELL-seq have notably reduced the number of long fragments per barcode, which may improve the assembly performance of microbial genomes containing high-copy repeats. In our previous study, we demonstrated that the characteristics of the reconstructed long DNA fragments (e.g., C_R_, C_F_, et al.) can significantly affect human genome assembly [21] and structural variant calling [32]. The improved stLFR and TELL-seq may also further refine the structural variant calling and variant phasing using the barcode specificity. We anticipate that future linked-read sequencing technologies will improve both DNA extraction techniques and long-fragment barcoding approaches to achieve 1 fragment per barcode.

Finally, it is worth mentioning that LRTK has the potential to be extended to handle other types of linked-read sequencing technologies. For example, in 2017, Illumina introduced the bead-based barcode partitioning in a single tube to phase human genomes. It further proposed the complete long-read technology for complex genomes in 2022 [50]. Additionally, Meier et al.[51] developed haplotype tagging to investigate the butterfly species. Redin et al. [52] recently introduced a novel library preparation method for high-throughput barcoding of short reads. The new single-cell metagenomic sequencing technologies employ highly accurate barcoded reads and provide inferable long-range information, which could potentially be used in combination with current linked-read technology in future studies [53]. We are actively developing LRTK to incorporate these technologies.

## Methods

### Data collection

We included 1 simulated metagenomic dataset (S1), 1 mock microbial community (B1), and 2 human gut metagenomic sequencing datasets (D1, D2) to evaluate the performance of the metagenomic data analysis section of LRTK (Supplementary Table S2). For S1, we simulated 11.8 Gb 10x Genomics and 9.2 Gb stLFR linked reads using LRTK-SIM [31] for 40 complete bacterial genomes extracted from the NCBI RefSeq database (December 2023) with the same abundances (lognormal distribution) (Supplementary Table S5). LRTK-SIM allows flexible parameters, such as C_F_, C_R_, N_F/P,_ μ_FL_, and Wμ_FL_, to simulate linked-read data. We set the parameters “C_F_ = 500, C_R_ = 0.2, N_F/P_ = 16, μ_FL_ = 20” to simulate 10x Genomics linked reads and “C_F_ = 500, C_R_ = 0.2, N_F/P_ = 1, μ_FL_ = 20” to simulate stLFR linked reads. The dataset B1 contains linked reads from a mock microbial community ATCC-MSA-1003 from 3 different platforms (SRR12283286 for 10x Genomics and PRJNA875547 for stLFR and TELL-seq). The ATCC-MSA-1003 mock community is composed of 20 bacterial species represented at staggered abundances—specifically, 5 species at 18%, 1.8%, 0.18%, and 0.02% abundance levels, respectively. The complete descriptions of the mock metagenomic sample, including the genome sizes, individual bacterial species, and their corresponding reference sequence accessions, have been included in Supplementary Table S4. The D1 contains 16 longitudinal human gut metagenomic 10x Genomics linked-read sequencing datasets from a single individual (accession number: SRP323279) (Supplementary Table S2). The D2 was downloaded from the China National GeneBank (CNGB) under project CNP0003432. It contains around 99 Gb stLFR human gut metagenomic sequencing data. In the human genome section, we collected the available linked-read sequencing data for the 3 technologies from NA12878, NA24143, NA24149, and NA24385. The detailed information has been included in Supplementary Table S2. For ATCC-MSA-1003 and NA12878, we further performed linked-read down-sampling at the barcode level by using in-house scripts.

### Data preprocessing

LRTK converts the raw linked reads from 10x Genomics, stLFR, and TELL-seq into a unified FASTQ format (Supplementary Fig. S1) and corrects potential sequencing errors in barcodes. For 10x Genomics and stLFR linked reads, the barcodes are aligned to their respective barcode whitelists using the “BWA aln” command (BWA, RRID:SCR_010,910). The barcodes with fewer than 2 mismatches in alignments are then corrected as the corresponding barcodes in the whitelist. LRTK adopts the approach described by Chen et al. [7] to correct barcode errors for TELL-seq due to the lack of barcode whitelist. In general, LRTK tallies the supporting reads for each barcode derived from TELL-seq linked reads and distinguishes between barcodes with a single supporting read and those with multiple supporting reads. It then corrects possible sequencing errors in barcodes that initially had 1 mismatch by comparing them to those with multiple supporting reads. The linked reads in the unified FASTQ file are then provided to fastp (RRID:SCR_016,962) [54] to remove adapter sequences and low-quality reads. For metagenomic sequencing data, the sequencing reads are aligned to the human genome first, and only unmapped reads are used for subsequent analysis.

### Metagenome assembly and contig binning

We evaluated the performance of 5 metagenome assemblers on linked reads—Athena (RRID:SCR_008,110) [40], Pangaea [30], cloudSPAdes [41], MEGAHIT (RRID:SCR_018,551) [38], and metaSPAdes [39]—and observed superior performance of Pangaea on stLFR and TELL-seq sequencing data. Therefore, for LRTK, we chose Pangaea as the default assembler to assemble linked-read metagenomic sequencing data for LRTK. After the initial assembly, LRTK extracts the circular contigs that are at least 1 Mb in length. The uncircularized contigs are grouped into MAGs using MetaBAT 2 (MetaBAT, RRID:SCR_019,134) [42]. According to standard criteria of the minimum information about MAGs [55], they could be classified into near complete (completeness >90%, contamination <5%, and could be detected 5S, 16S, and 23S ribosomal RNAs and at least 18 transfer RNAs), high quality (completeness >90% and contamination <5%), medium quality (completeness ≥50% and contamination <10%), and low quality (the other MAGs).

### Barcode-aware read alignment

For human genome sequencing data, LRTK utilizes EMA, a barcode-aware alignment approach, to map high-quality 10x Genomics linked reads to the human reference genome [56]. We further modified EMA to be compatible with the barcodes from stLFR (30 bps) and TELL-seq (18 bps). LRTK marks PCR duplicates for each barcode using the “BARCODE_TAG” parameter in Picard [57] (RRID:SCR_006,525). The alignment files are then sorted according to the genomic coordinates of alignments for further analysis.

For metagenomic sequencing data, we developed a tiered alignment approach to align the linked reads to microbial genomes using the aforementioned modified EMA. The default reference genomes for the human gut metagenome were downloaded from UHGG [19]. For nongut samples, we used the GTDB [58] as the reference database, but users could also incorporate their own custom database.

In the first round of alignment, linked reads were directly mapped to the 4,724 representative genomes in UHGG (by default). For each genome, LRTK calculates the coverage rate and covered base and then extracts genomes with “coverage rate >40% and covered base >500 kb.” These genomes will be used as the candidate reference genomes for the second round of alignment.

In the second round of alignment, linked reads are only mapped to those candidate reference genomes to reduce multiple alignment errors. For each candidate genome, LRTK partitions it into 10-kb windows and calculates the number of mapped reads on each window. The mapped reads are categorized into 2 types: unique-mapped reads (U) and multimapped reads (M), which will be processed separately. LRTK uses the formulas below to determine the total read count of a window (RC(W)), unique-mapped read count ($RC( U )$), and multimapped read count ($RC( M )$), where $l$ is the window size.


\begin{eqnarray*}
RC\left( W \right) &=& RC\left( U \right) + RC\left( M \right)\\ RC\left( U \right) &=& \frac{U}{l}\\ RC\left( M \right) &=& \mathop \sum \limits_{i = 1}^M {\omega }_i*\left\{ M \right\}/l\\ \end{eqnarray*}

A coefficient $\omega $ is introduced for multimapped reads. When a multimapped in M is aligned to N different genomes, $\omega $ is calculated using the following formula:


\begin{eqnarray*}
\omega = U/\mathop \sum \limits_{i = 1}^N RC\left( U \right) \end{eqnarray*}

LRTK removes the windows with an extreme number of reads and calculates the average depth using the remaining windows as the depth of the corresponding genome. The relative abundance is then calculated by aggregating the average depth of all identified genomes.

In the experiments, we further evaluated the performance (F1 score, precision, and recall) of LRTK, KMCP [34], and MIDAS 2 [36] to identify microbes and quantify their abundances. The reference databases were prepared using the genome sequences from the GTDB database for the 3 tools. We also compared LRTK with the widely used taxonomic classification tools: Bracken [33] and MetaPhlAn 2 (MetaPhlAn, RRID:SCR_004,915) [35], using their default databases.

### Reconstruction of long DNA fragments

Initially, LRTK extracts unique-mapped co-barcoded paired-end short reads from the alignment file to evaluate the distribution of insert sizes (with a mean of μ_PE_ and a standard deviation of σ_PE_). Alignments are removed if the distance between 2 reads in a paired-end read (R1 and R2) exceeds a certain threshold, that is,


\begin{eqnarray*}
Dis\; \left( {{\mathrm{R}}1,{\mathrm{R}}2} \right) > {\mu }_{PE} + 3 * {\sigma }_{PE}. \end{eqnarray*}

The remaining paired-end reads are used as seeds and extended to both directions to connect with other seeds sharing the same barcode until no more eligible seeds can be found within a specified distance (200 kb by default). All of these co-barcoded reads are considered to be derived from the same long DNA fragment.

### Identify and phase genomic variants

LRTK supports multiple popular variant detection tools and adheres to best practices for linked-read sequencing. For human genome sequencing data, LRTK provides FreeBayes (RRID:SCR_010,761) [22], SAMtools RRID:SCR_002,105) [23], and GATK (RRID:SCR_001,876) [24] to call SNVs and small INDELs. FreeBayes is a Bayesian genetic variant detection tool designed to identify SNVs, INDELs, multinucleotide polymorphisms, and more complex events, which is an easy-to-use and time-saving tool [59]. GATK, while also employing a Bayesian framework, enhances its detection capabilities for insertions and deletions through specialized techniques such as read realignment and base recalibration. Although these steps add value, they also increase GATK’s computational runtime. In contrast, SAMtools uses a hidden Markov model for the identification of small variants and has demonstrated robust performance across various studies. The available phasing tools include HapCUT2 (HapCUT, RRID:SCR_010,791) [28] and WhatsHap [29]. HapCUT2 demonstrates excellent performance in phasing heterozygous SNVs within a diploid context, such as the human genome. WhatsHap, however, introduces an innovative clustering and threading approach that delivers precise phasing in polyploid genomes. Having obtained the phased SNVs, we used PhaseME [60] to calculate the phasing block and phasing rate to assess the phasing quality. Subsequently, we used hap-ibd [61] to detect pairwise identity-by-descent segments across multiple samples. By default, large SVs are identified using Aquila [25]. However, users have the flexibility to select other tools such as LinkedSV [26] and VALOR2 [27] for SV analysis. LinkedSV leverages barcode overlapping, read depth, paired-end signals, and local assembly to detect deletions, although it currently lacks support for insertion detection. In contrast, the assembly-based tool Aquila is capable of detecting both insertions and deletions, offering a comprehensive solution.

For metagenome sequencing data, LRTK offers 3 metagenomic SNV callers: FreeBayes [22], inStrain [37], and SAMtools [23]. For FreeBayes and SAMtools, LRTK further removes the SNVs if the total depths are less than 6, the number of reads supporting the alternative allele is less than 2, and SNV qualities are below 15. LRTK does not perform quality control for the SNVs called by inStrain as it does not output SNV quality scores. The SNV phasing was performed on high-abundance species using WhatsHap [29] with the inferred ploidy. LRTK also selected SNVs located on certain high-abundance species and compared the SNV profiles across multiple samples. Based on the allele frequency of SNVs, we used an unsupervised clustering method to detect potential strain frequency change events for pairwise samples. To detect strain frequency change, 1 sample was chosen as the reference for all the contigs. For each species that was present in at least 2 samples, the minor allele was determined based on the reference sample. The minor allele frequency (MAF) was calculated as the ratio of the minor allele count to the total allele count. For each interested species, MAFs of SNVs (minimum supported read number exceeding 2) were extracted from each sample and merged into a combined SNV set. Based on the combined SNV set, The MAFs for the interested sample were then compared with the MAFs of the reference sample. We used the *k*-means clustering method to separate the SNVs into distinct groups using the minor allele frequency matrix. The number of clusters was determined by using the Calinski–Harabasz index.

### Downstream analysis and HTML-based visualization

The human genome analysis report begins with generating the FASTQ quality control statistics during the preprocessing step. The barcode-aware alignment step presents the distribution of several key statistics about the DNA sequencing library, including the number of fragments per barcode, fragment length, and average read coverage per fragment. It also includes information about the sequencing coverage and insert size. The variant calling step summarizes the number of SNVs, small INDELs, and large SVs and illustrates their distributions.

Similarly, the metagenome analysis report illustrates the quality control results for preprocessing, alignment, and variant calling steps. Additionally, it includes an optional report about the automatic analysis of multiple related samples. The report shows the distribution of high abundant species across multiple samples and uses a principal component analysis plot to visualize the divergence across them. The report also depicts the distribution of SNVs of these high-abundant species and the minor allele frequency distribution in pairwise samples.

## Code Availability and Requirements

Project name: Linked Read ToolKits project

Project homepage: [62]

Packaged conda environment: [63]

Operating system(s): Linux and macOS

Programming language: C and Python (Python Programming Language, RRID:SCR_008394)

Other requirements: Conda (Conda, RRID:SCR_018317), Python 3.6 or higher

License: MIT

RRID:SCR_023,945

Biotools ID: biotools:lrtk

## Additional Files

**Supplementary Fig. S1**. LRTK text file specification.

**Supplementary Fig. S2**. Ideogrammatic definitions of C_R_, C_F_, N_F/P_, μ_FL_, and Wμ_FL_ metrics.

**Supplementary Fig. S3**. Evaluation of taxonomic quantification performance for 10x Genomics and TELL-seq linked reads.

**Supplementary Fig. S4**. Evaluation of assembly performance at species level.

**Supplementary Fig. S5**. Evaluation of metagenomic assemblers on simulated linked-read data.

**Supplementary Fig. S6**. Demo reports for metagenomic sequencing.

**Supplementary Fig. S7**. Demo reports for human genome sequencing.

**Supplementary Fig. S8**. Computational requirements for linked-reads preprocessing and alignment between LRTK and Long Ranger on NA12878.

**Supplementary Table S1**. Bioinformatics tools included in LRTK.

**Supplementary Table S2**. Linked-read sequencing data used in the article.

**Supplementary Table S3**. Data descriptions for the simulated and down-sampled linked reads from ATCC-MSA-1003 and NA12878.

**Supplementary Table S4**. Reference genomes for the ATCC-MSA-1003 mock sample.

**Supplementary Table S5**. Reference genomes for the simulated dataset.

## Abbreviations

INDEL: small insertion and deletion; MAF: minor allele frequency; MAG: metagenome-assembled genome; NCBI: National Center for Biotechnology Information; SCC: Spearman correlation coefficient; SNV: single nucleotide variant; stLFR: single-tube long fragment read; SV: structural variation; TELL-seq: transposase enzyme-linked long-read sequencing.

## Supplementary Material

giae028_Supplemental_Files

giae028_GIGA-D-23-00278_Original_Submission

giae028_GIGA-D-23-00278_Revision_1

giae028_GIGA-D-23-00278_Revision_2

giae028_Response_to_Reviewer_Comments_Original_Submission

giae028_Response_to_Reviewer_Comments_Revision_1

giae028_Reviewer_1_Report_Original_Submission Brock Peters, Ph.D. -- 10/20/2023

giae028_Reviewer_2_Report_Original_Submission Lauren Mak, MSc -- 11/12/2023

giae028_Reviewer_2_Report_Revision_1 Lauren Mak, MSc -- 3/29/2024

giae028_Reviewer_3_Report_Original_Submission Dmitrii Meleshko -- 11/13/2023

## Data Availability

We included 1 simulated metagenomic dataset (S1), 1 mock microbial community (B1), and 2 human gut metagenomic sequencing datasets (D1, D2) in the metagenome section and the human genome sequencing data for 4 samples (D3) in the human genome section (Supplementary Table S2). **B1:** The mock microbial community B1 contains the 10x Genomics, stLFR, and TELL-seq sequencing data for the ATCC-MSA-1003 mock community. These datasets were obtained from the NCBI with the following accession numbers: SRR12283286 for 10x Genomics and PRJNA875547 for stLFR and TELL-seq sequencing technologies. **D1:** The first real metagenomic dataset (D1), consisting of longitudinal 10x Genomics linked-read sequencing data, was downloaded from the NCBI under accession number SRP323279. **D2:** The second real metagenomic dataset (D2), containing deep stLFR sequencing data, was downloaded from the China National GeneBank (CNGB) under project CNP0003432. **D3:** The human genome dataset D3 contains the linked-read sequencing data for NA12878, NA24143, NA24149, and NA24385. The 10x Genomics and stLFR sequencing data were downloaded following the links in Supplementary Table S2. For TELL-seq sequencing data, we only obtained the raw TELL-seq data from NA12878 and NA24385 from the SRA database under accession SRX7264479 and SRX7264481, respectively. Supporting data are also available via the *GigaScience* database, GigaDB [64]. An archival copy of the code is available via Software Heritage [65].
