# Supplementary figures and images for: LRTK: a platform agnostic toolkit for linked-read analysis of both human genome and metagenome
Source: Gigascience. 2024 Jun 13;13:giae028. doi: 10.1093/gigascience/giae028 (PMC11170215; doi:10.1093/gigascience/giae028)

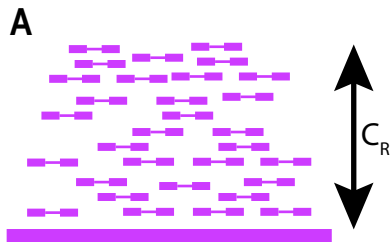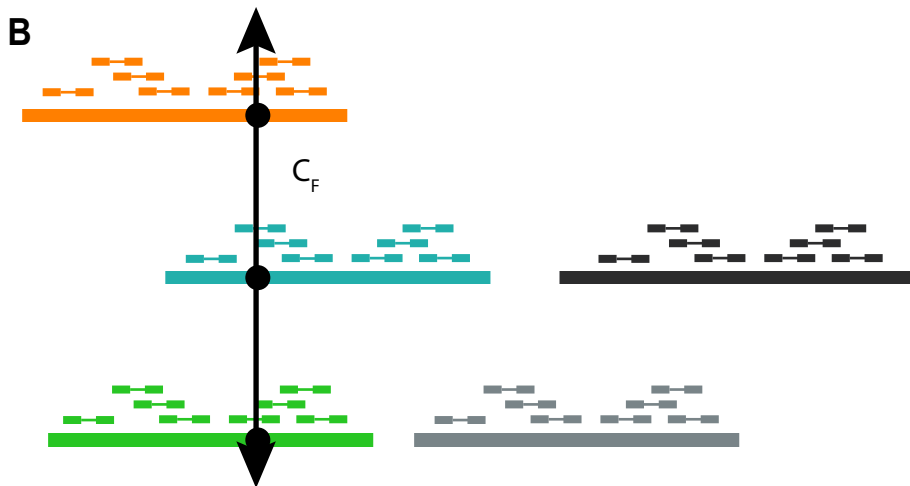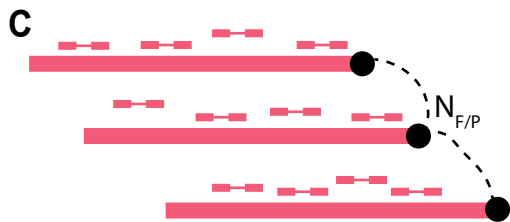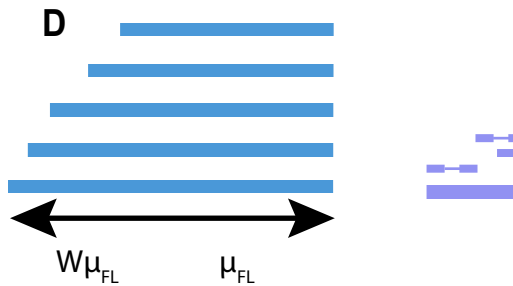

Supplement: giae028_Supplemental_Files [file giae028_supplemental_files.zip › FigureS2.pdf]

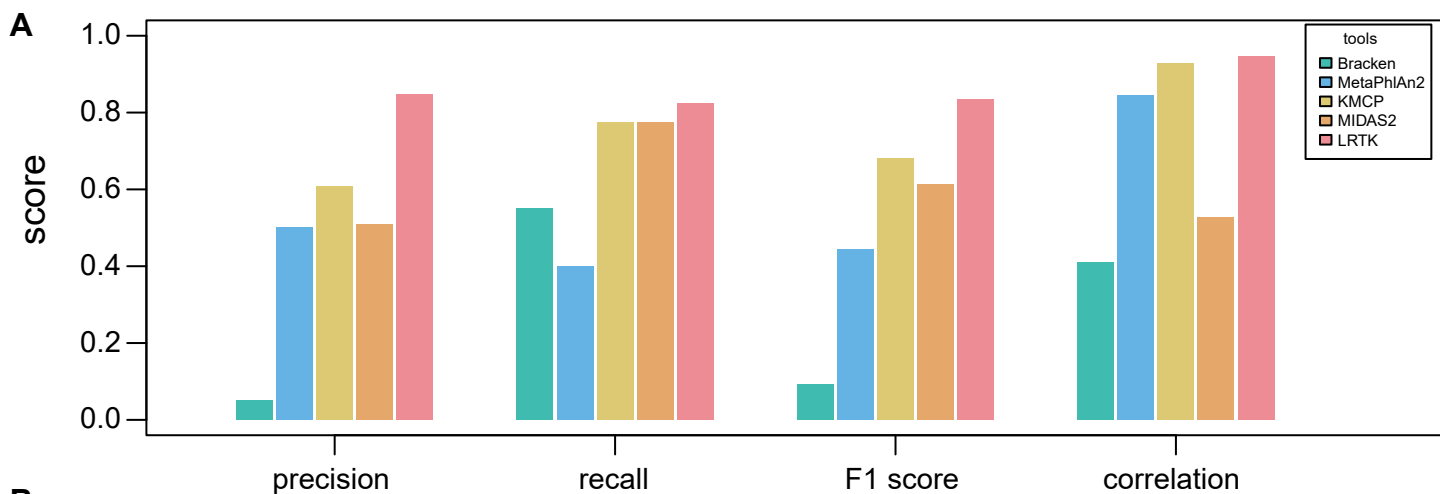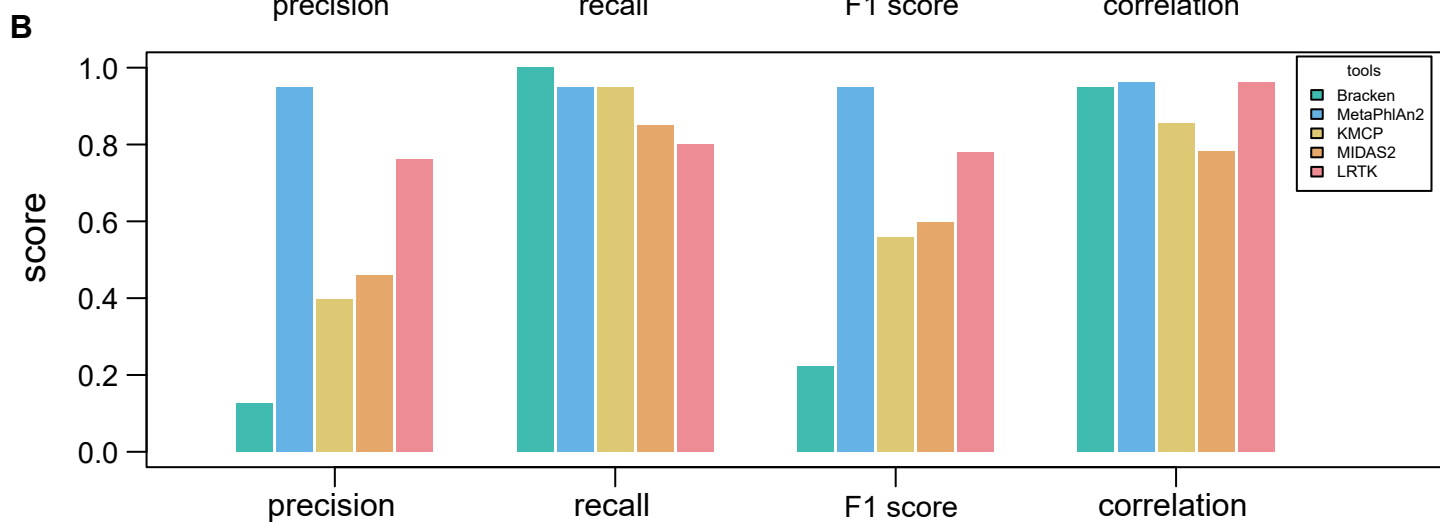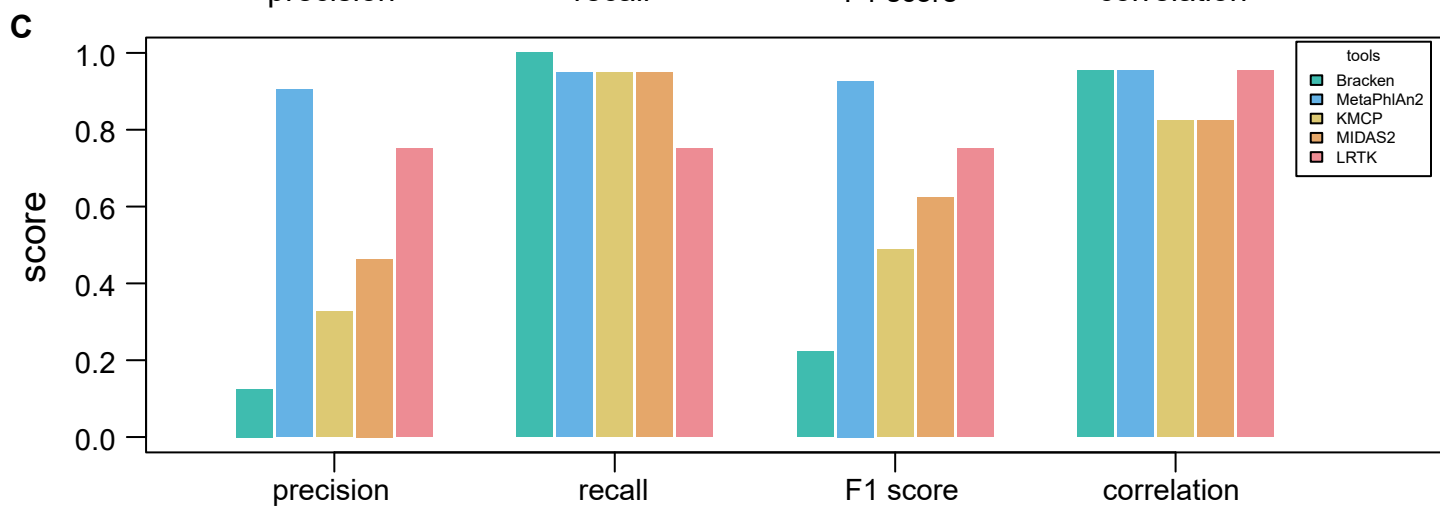

Supplement: giae028_Supplemental_Files [file giae028_supplemental_files.zip › FigureS3.pdf]

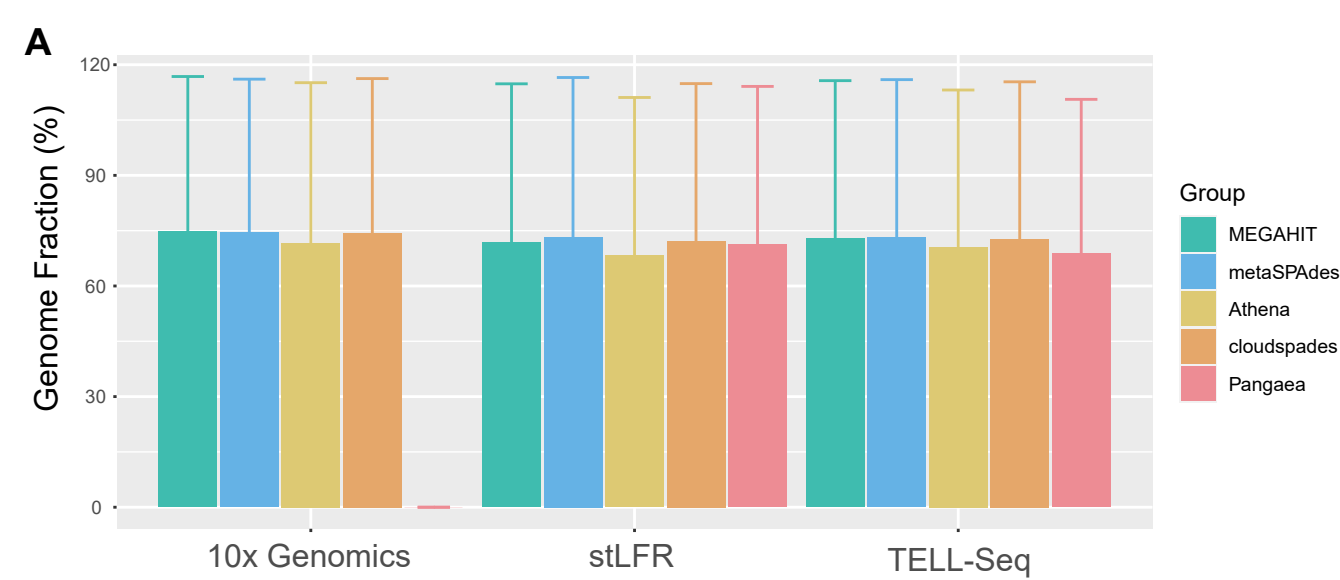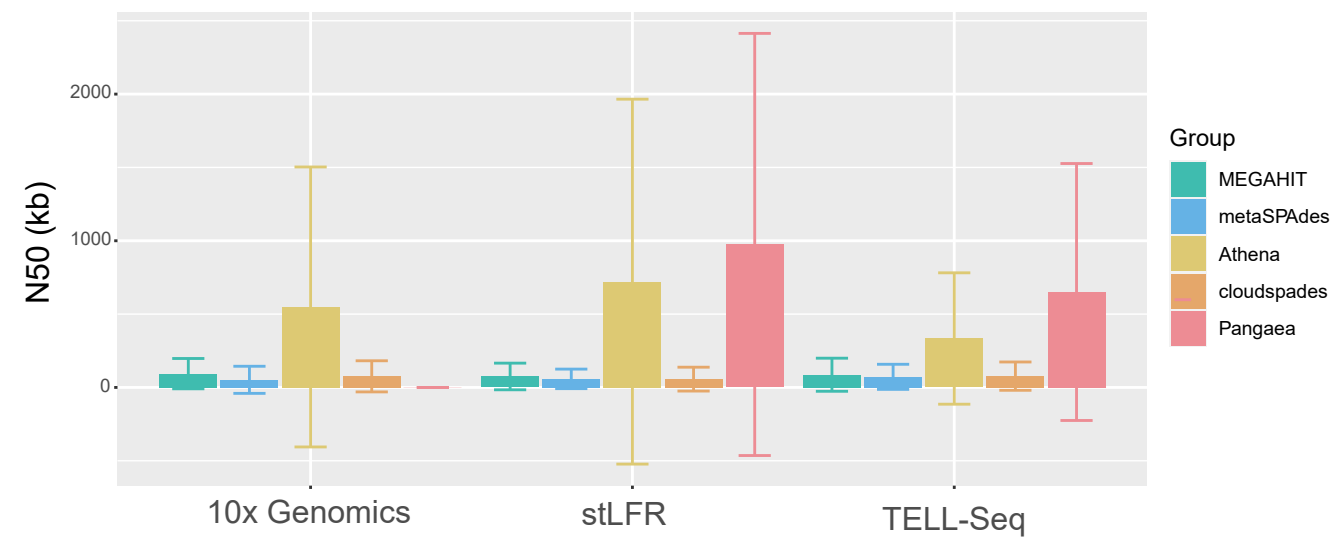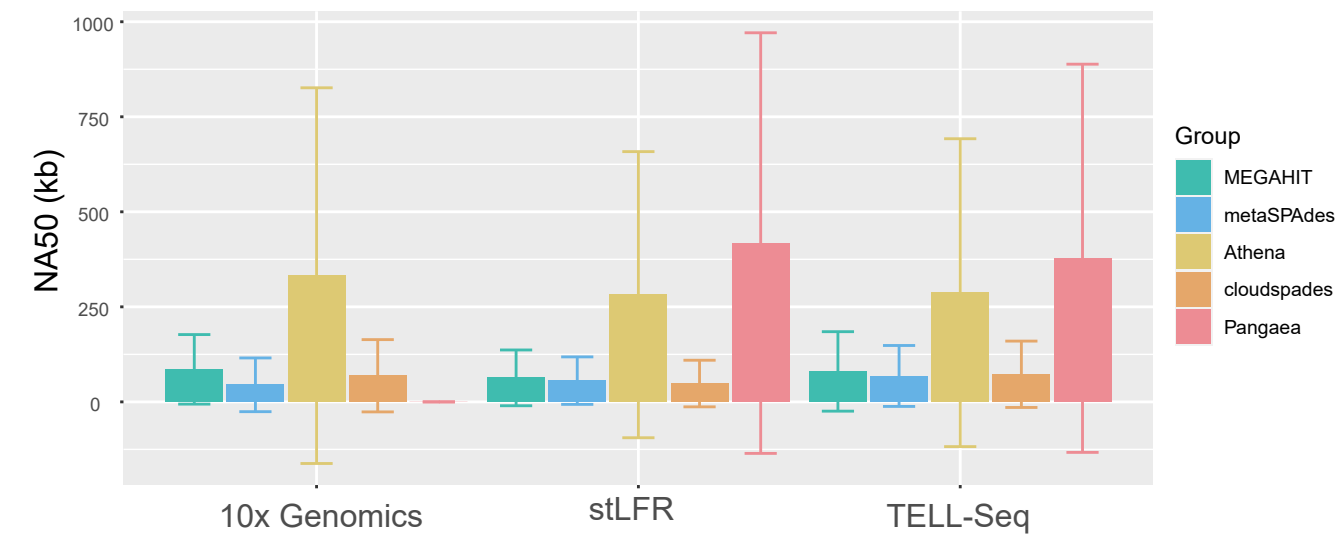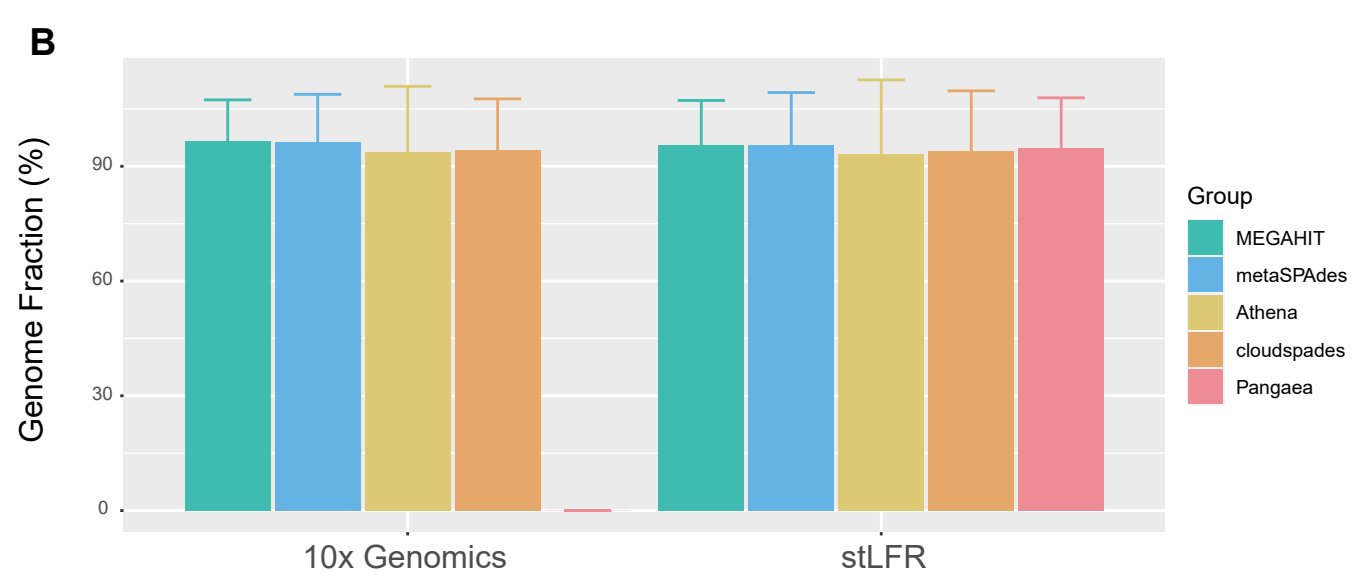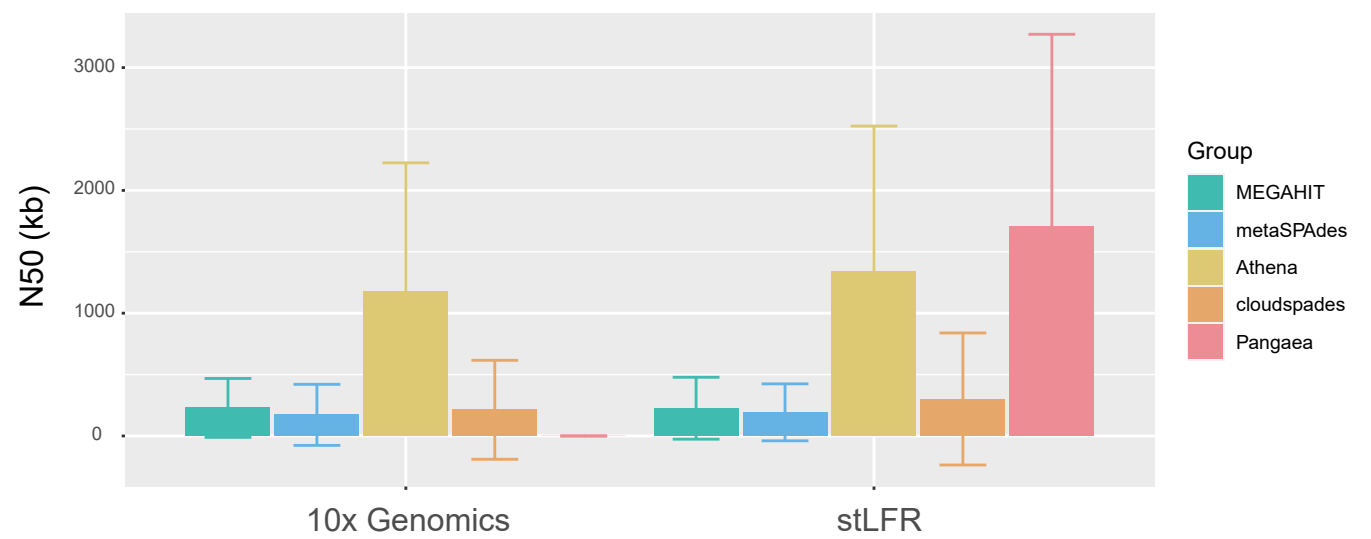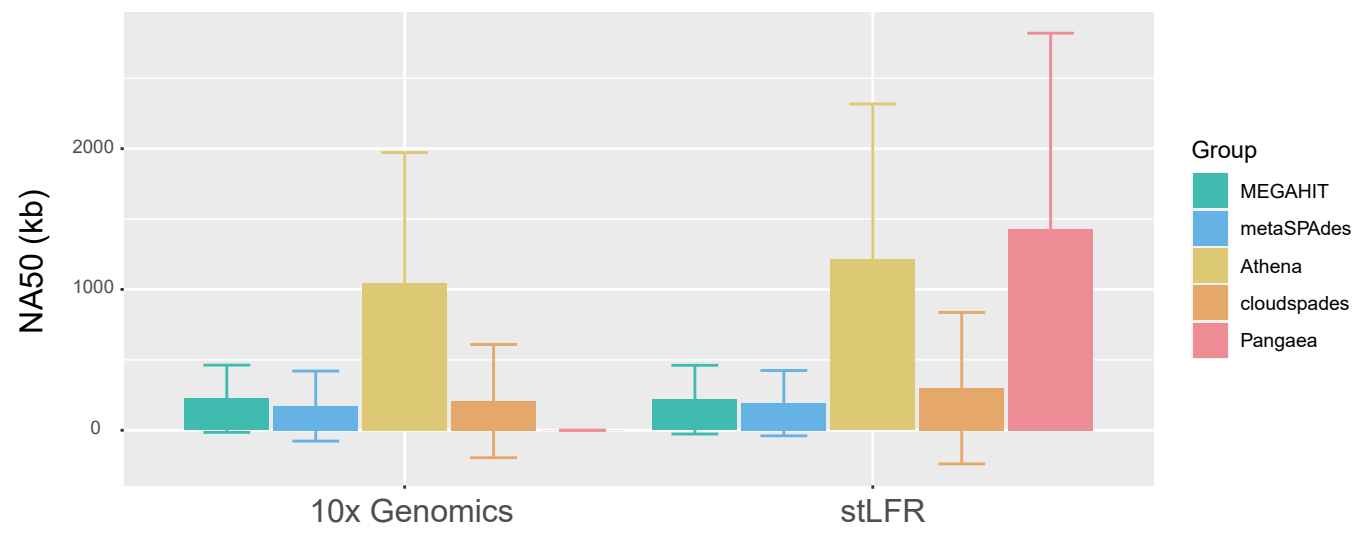

Supplement: giae028_Supplemental_Files [file giae028_supplemental_files.zip › FigureS4.pdf]

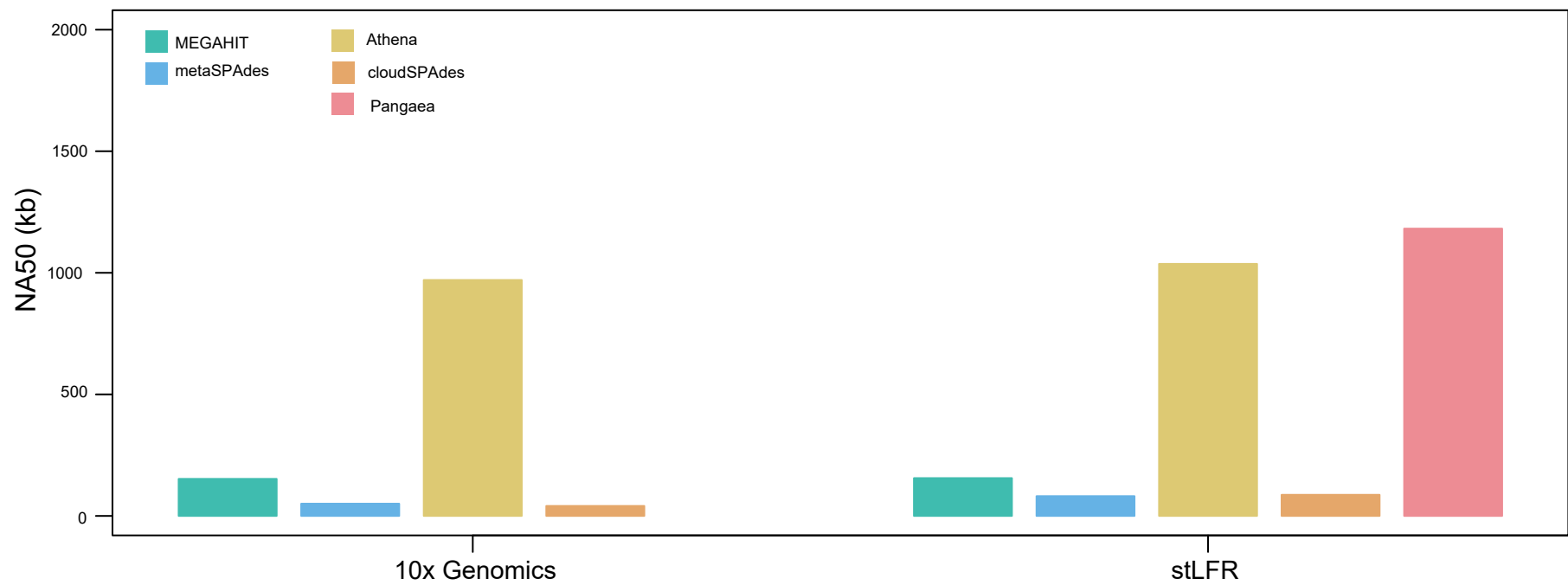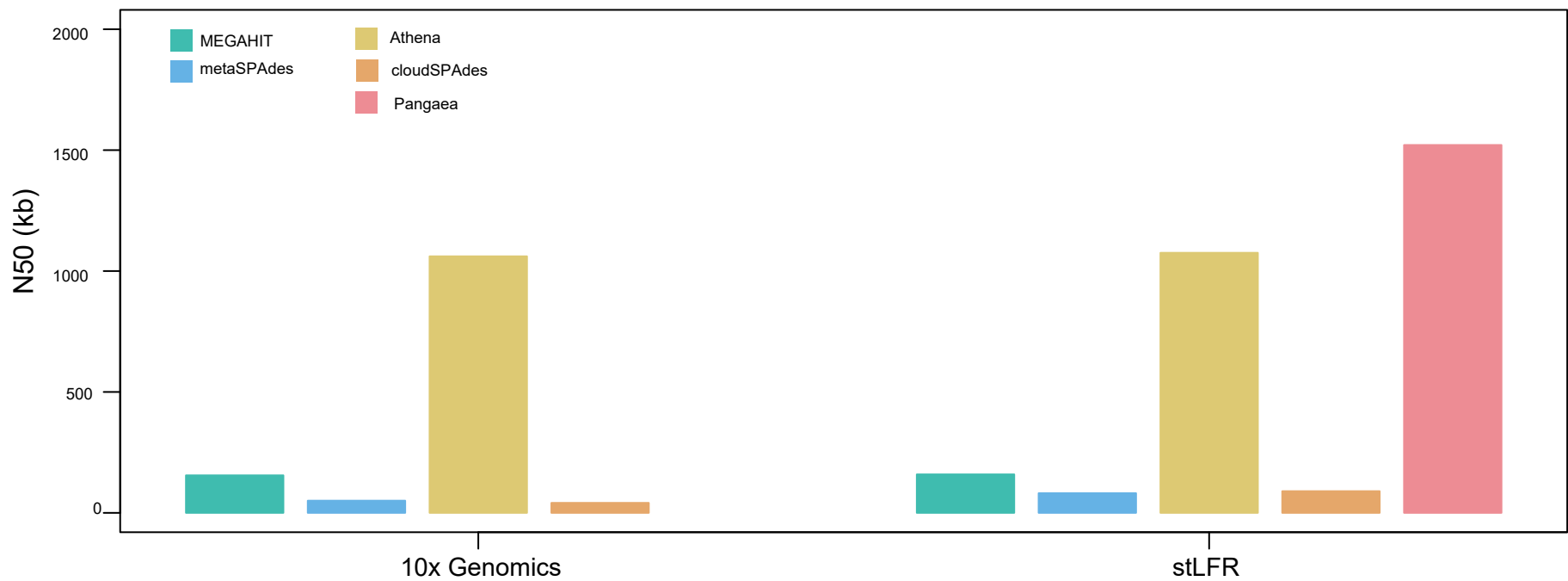

Supplement: giae028_Supplemental_Files [file giae028_supplemental_files.zip › FigureS5.pdf]

**A**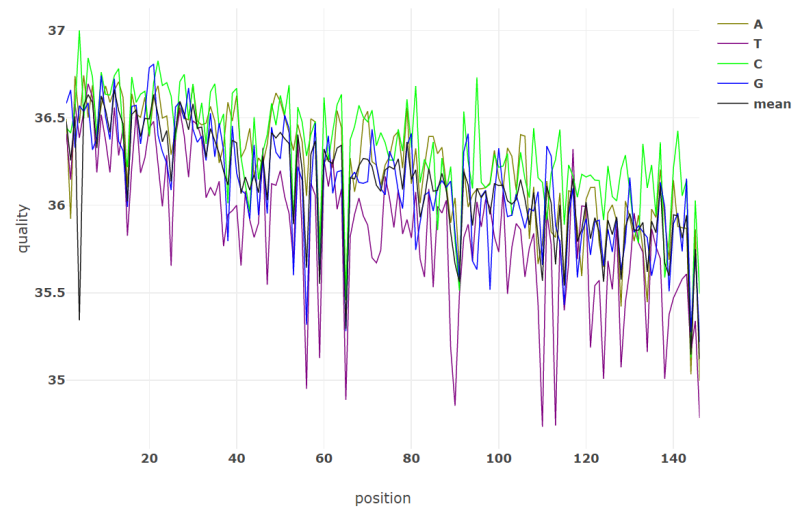**B**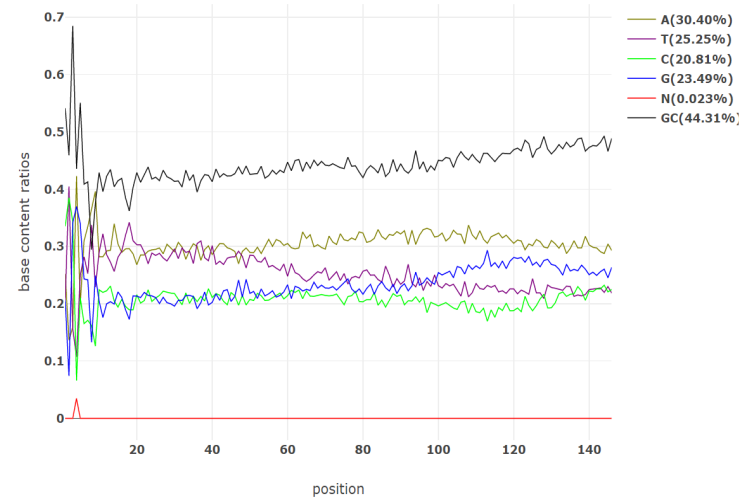**C**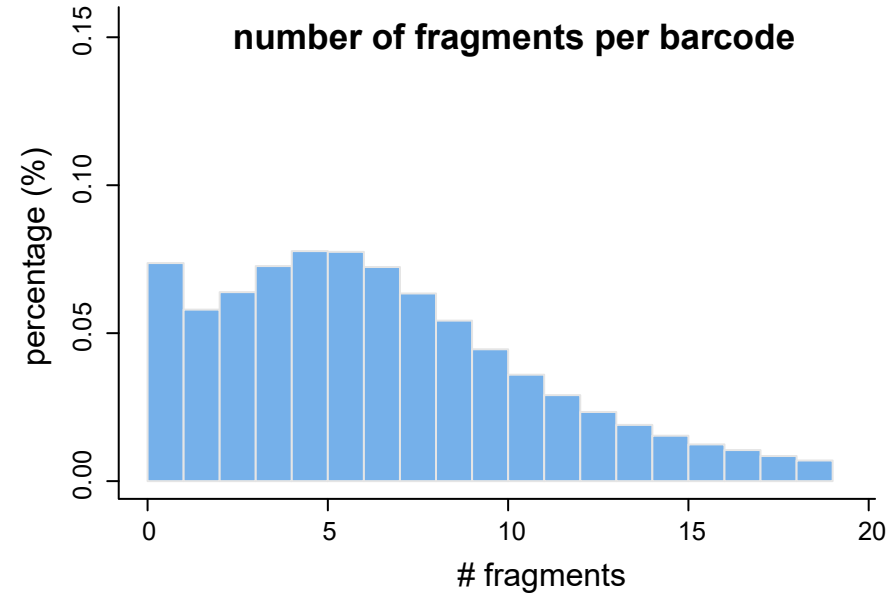**D**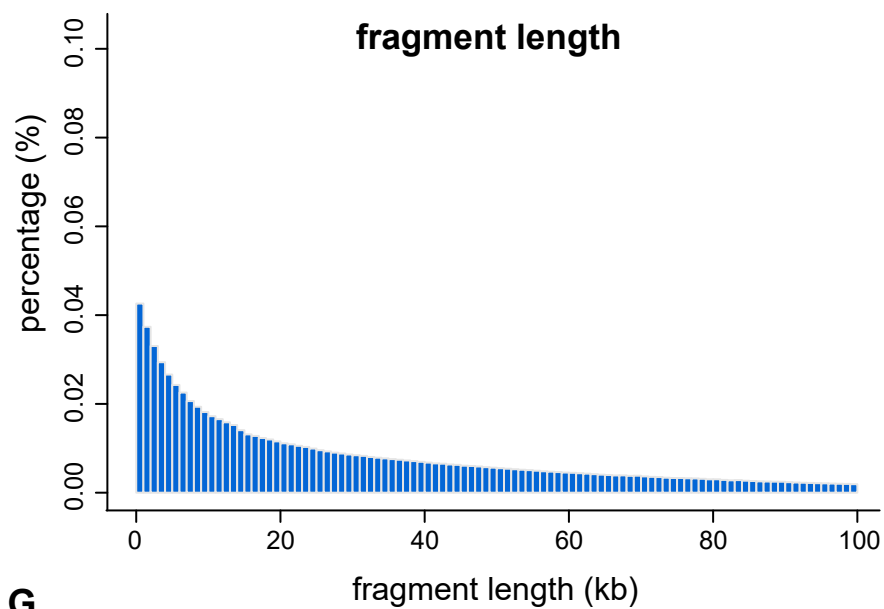**G**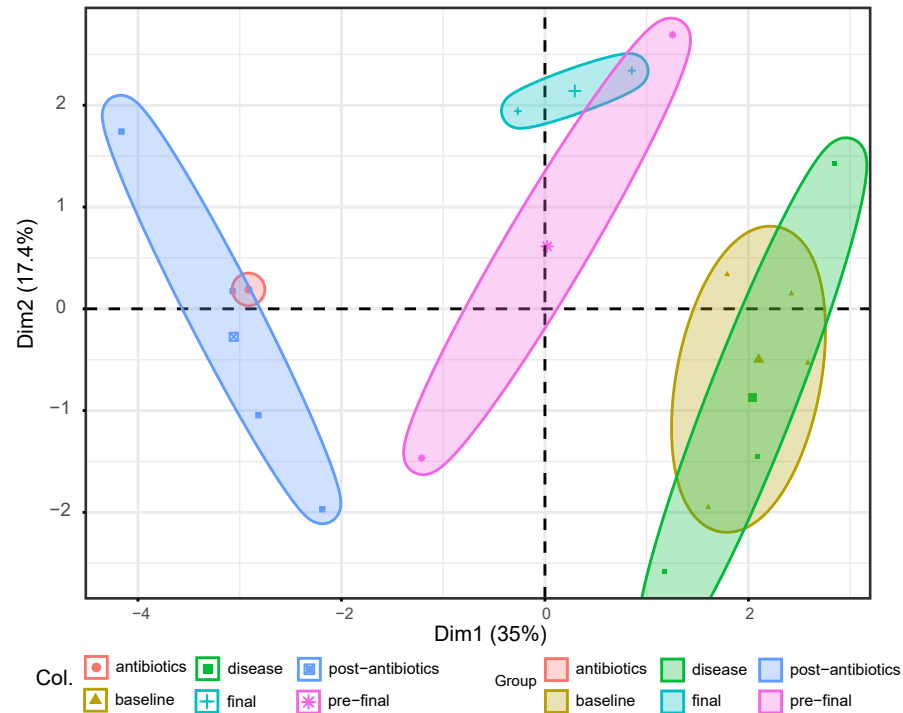**E**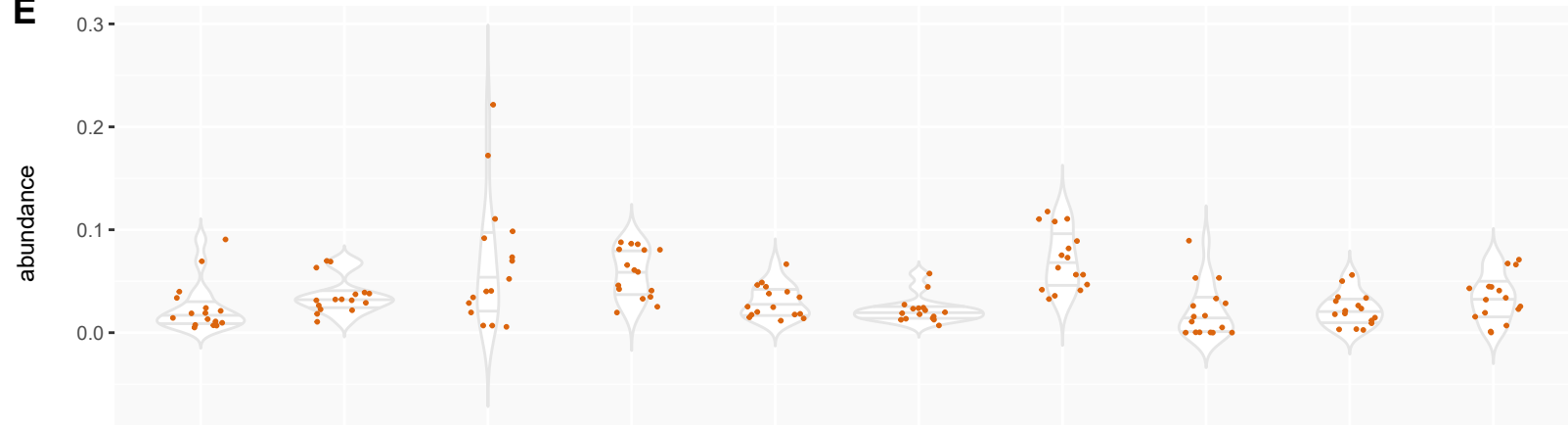**F**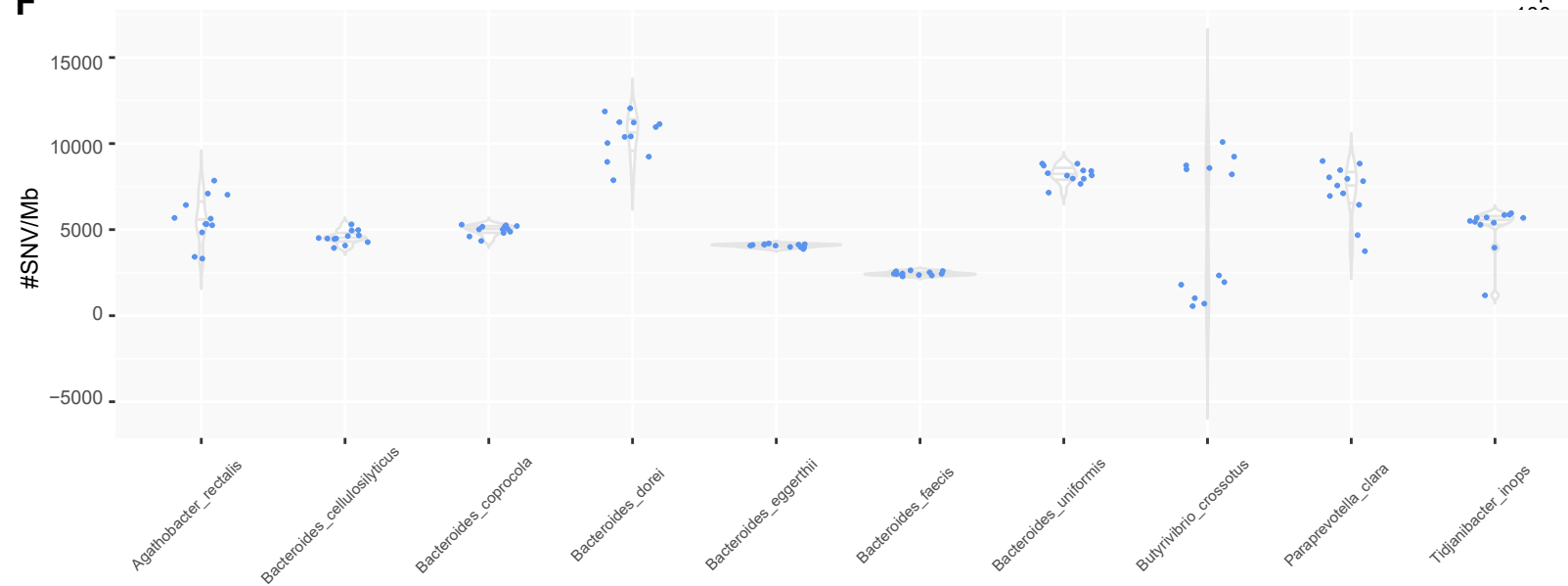

Supplement: giae028_Supplemental_Files [file giae028_supplemental_files.zip › FigureS6.pdf]

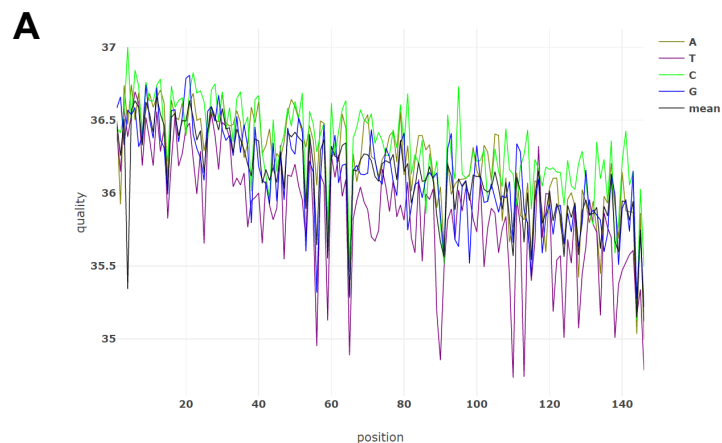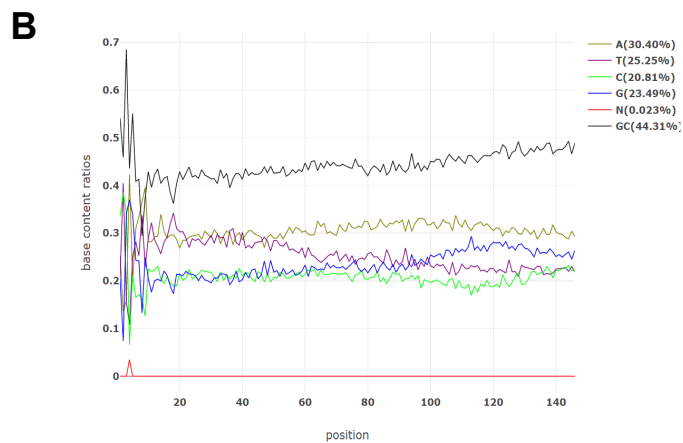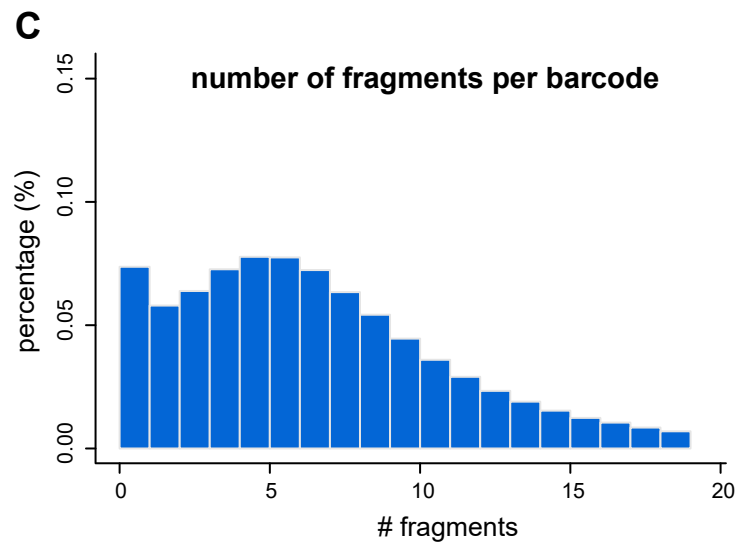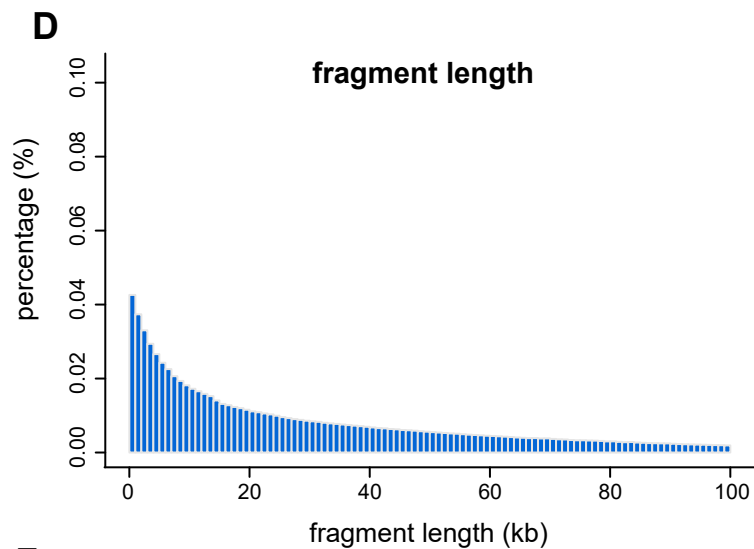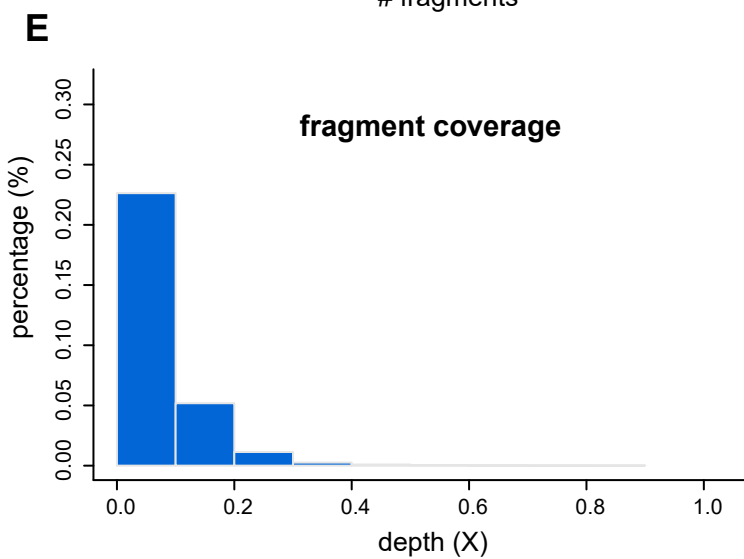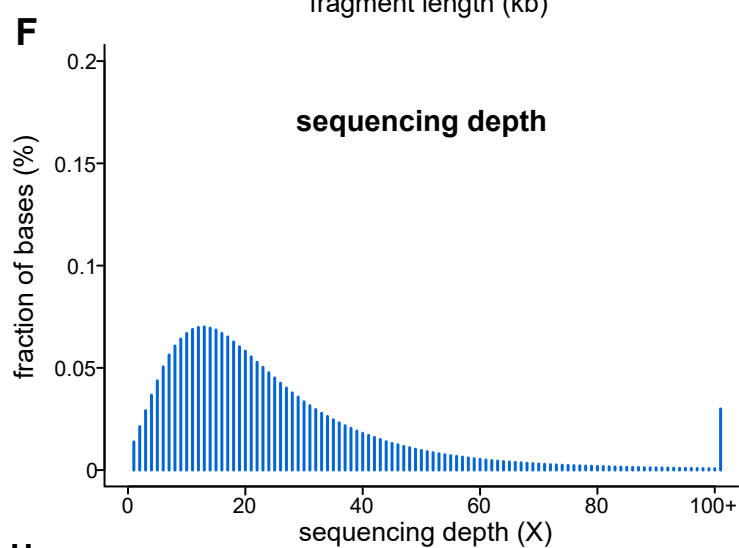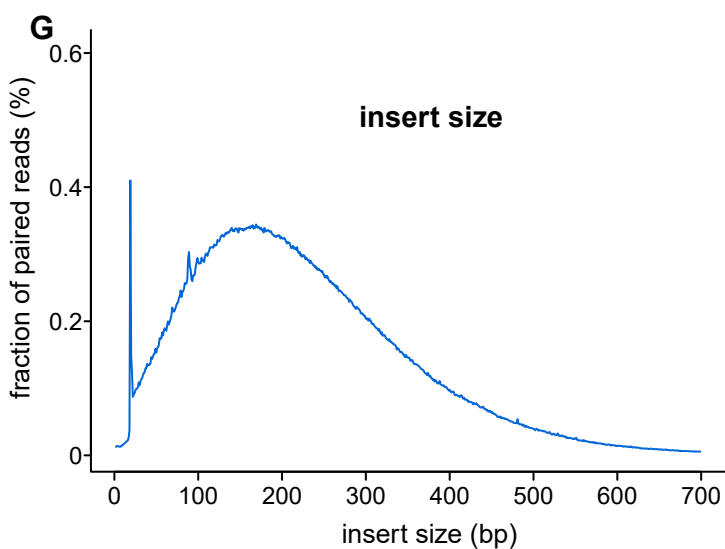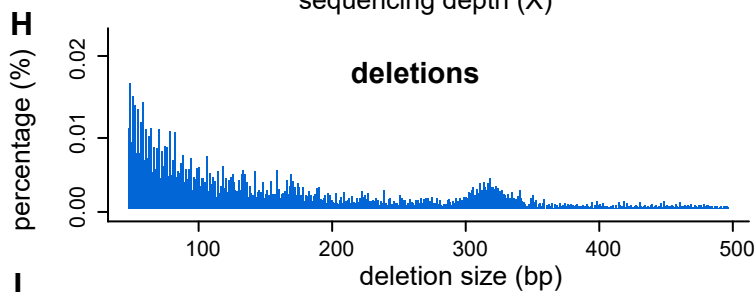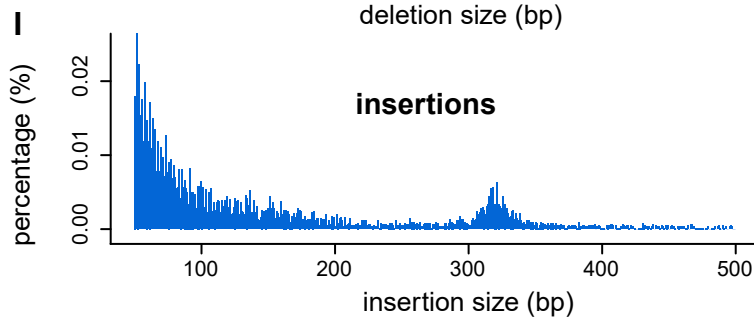

Supplement: giae028_Supplemental_Files [file giae028_supplemental_files.zip › FigureS7.pdf]

**A**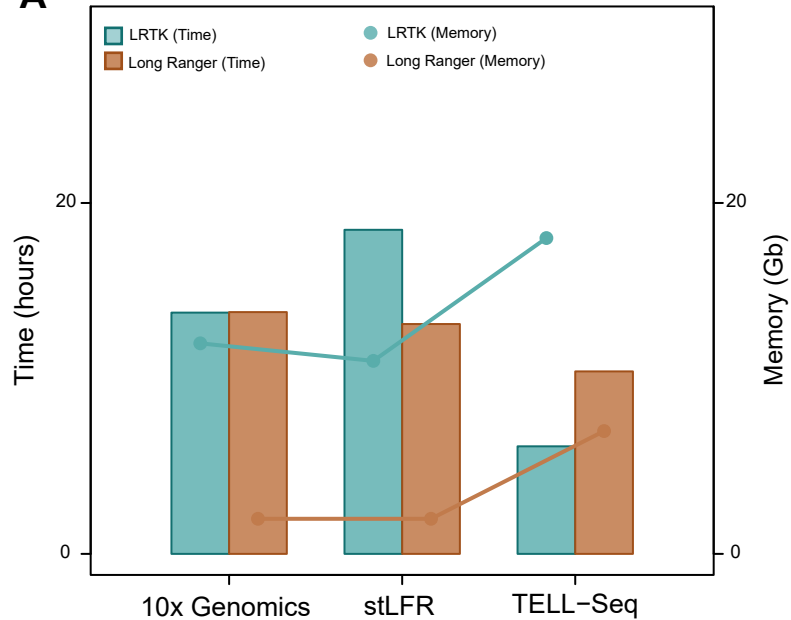**B**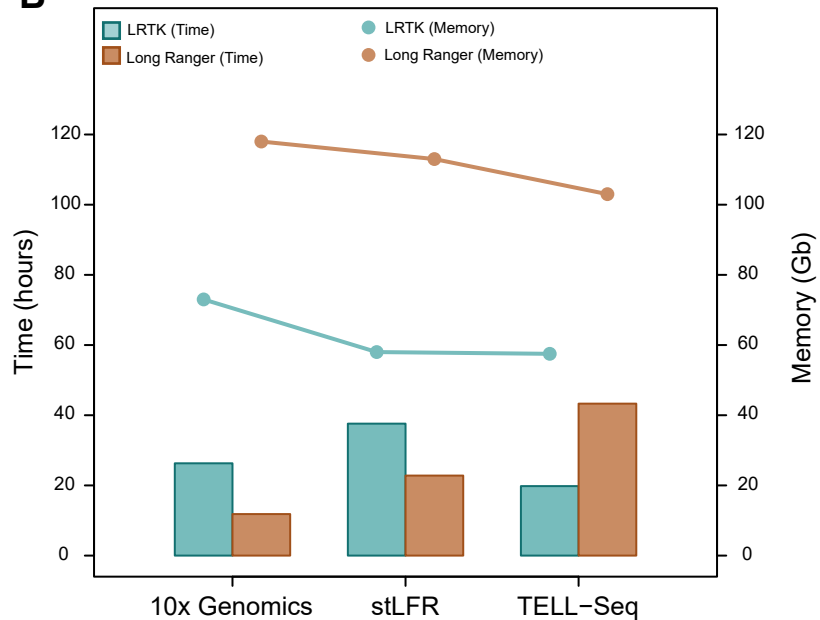

Supplement: giae028_Supplemental_Files [file giae028_supplemental_files.zip › FigureS8.pdf]
