# Supplementary material for: LRTK: a platform agnostic toolkit for linked-read analysis of both human genome and metagenome
Source: Gigascience. 2024 Jun 13;13:giae028. doi: 10.1093/gigascience/giae028 (PMC11170215; doi:10.1093/gigascience/giae028)
Supplement: giae028_GIGA-D-23-00278_Revision_1 [file giae028_giga-d-23-00278_revision_1.pdf]

GigaScience

LRTK: A platform agnostic toolkit for linked-read analysis of both human genome and metagenome

--Manuscript Draft--

**Manuscript Number:**	GIGA-D-23-00278R1
**Full Title:**	LRTK: A platform agnostic toolkit for linked-read analysis of both human genome and metagenome
**Article Type:**	Technical Note
**Funding Information:**	open project of BGI-Shenzhen (BGIRSZ20220012) — Dr. Lu Zhang<br>Hong Kong Research Grant Council Early Career Scheme (HKBU 22201419) — Dr. Lu Zhang<br>HKBU Start-up Grant Tier 2 (RC-SGT2/19-20/SCI/007) — Dr. Lu Zhang<br>HKBU IRCMS (No. IRCMS/19-20/D02) — Dr. Lu Zhang<br>Basic and Applied Basic Research Foundation of Guangdong Province (No. 2021A1515012226) — Dr. Lu Zhang<br>Science Technology and Innovation Committee of Shenzhen Municipality (SGDX20190919142801722) — Dr. Xiaodong Fang<br>Shenzhen Science and Technology Innovation Commission (SZSTI) - Shenzhen Virtual University Park (SZVUP) Special Fund Project (No. 2021Szvup135) — Dr. Lu Zhang
**Abstract:**	Linked-read sequencing technologies generate high-base quality short-reads that contain extrapolative information on long-range DNA connectedness. These advantages of linked-read technologies are well-known and have been demonstrated in many human genomic and metagenomic studies. However, existing linked-read analysis pipelines (e.g., Long Ranger) were primarily developed to process sequencing data from the human genome and are not suited for analyzing metagenomic sequencing data. Moreover, linked-read analysis pipelines are typically limited to one specific sequencing platform. To address these limitations, we present the Linked-Read ToolKit (LRTK), a unified and versatile toolkit for platform agnostic processing of linked-read sequencing data from both human genome and metagenome. LRTK provides functions to perform linked-read simulation, barcode sequencing error correction, barcode-aware read alignment and metagenome assembly, reconstruction of long DNA fragments, taxonomic classification and quantification, as well as barcode-assisted genomic variant calling and phasing. LRTK has the ability to process multiple samples automatically, and provides users with the option to generate reproducible reports during processing of raw sequencing data and at multiple checkpoints throughout downstream analysis. We applied LRTK on linked-reads from simulation, mock community and real datasets for both human genome and metagenome. We showcased LRTK's ability to generate comparative performance results from preceding benchmark studies and to report these results in publication-ready HTML document plots. LRTK provides comprehensive and flexible modules along with an easy-to-use Python-based workflow for processing linked-read sequencing datasets, thereby filling the current gap in the field caused by platform-centric genome-specific linked-read data analysis tools.
**Corresponding Author:**	Lu Zhang<br>Hong Kong Baptist University<br>Hong Kong, Please select a state CHINA
**Corresponding Author Secondary Information:**	
**Corresponding Author's Institution:**	Hong Kong Baptist University

Powered by Editorial Manager® and ProduXion Manager® from Aries Systems Corporation

**Corresponding Author's Secondary Institution:**	
**First Author:**	Lu Zhang
**First Author Secondary Information:**	
**Order of Authors:**	Lu Zhang
	Chao Yang
	Zhenmiao Zhang
	Yufen Huang
	Xuefeng Xie
	Herui Liao
	Jin Xiao
	Werner Pieter Veldsman
	Kejing Yin
	Xiaodong Fang
**Order of Authors Secondary Information:**	
**Response to Reviewers:**	Cover Letter<br>Dear editor and reviewers,<br>Many thanks indeed for all your hard work on our manuscript. The comments raised by reviewers are very constructive and valuable, providing us lots of insights and inspirations. We have made careful data re-analyzing and literature research, and made extensive revision to our manuscript and supplemented data. All the comments from reviewers have been addressed point by point as shown below, and corresponding corrections have been made in the revised manuscript.<br>Thanks for your consideration of our paper and kindly let us know if there's any further explanation needed!<br><br>Sincerely,<br>Lu Zhang<br>Department of Computer Science, Faculty of Science, Hong Kong Baptist University, Hong Kong<br>E-mail: ericluzhang@hkbu.edu.hk<br>Phone: +852 3411 5880
**Additional Information:**	
**Question**	**Response**
Are you submitting this manuscript to a special series or article collection?	No
**Experimental design and statistics**<br><br>Full details of the experimental design and statistical methods used should be given in the Methods section, as detailed in our Minimum Standards Reporting Checklist. Information essential to interpreting the data presented should be made available in the figure legends.<br><br>Have you included all the information	Yes

requested in your manuscript?	
**Resources**<br><br>A description of all resources used, including antibodies, cell lines, animals and software tools, with enough information to allow them to be uniquely identified, should be included in the Methods section. Authors are strongly encouraged to cite Research Resource Identifiers (RRIDs) for antibodies, model organisms and tools, where possible.<br><br>Have you included the information requested as detailed in our Minimum Standards Reporting Checklist?	Yes
**Availability of data and materials**<br><br>All datasets and code on which the conclusions of the paper rely must be either included in your submission or deposited in publicly available repositories (where available and ethically appropriate), referencing such data using a unique identifier in the references and in the "Availability of Data and Materials" section of your manuscript.<br><br>Have you have met the above requirement as detailed in our Minimum Standards Reporting Checklist?	Yes

Manuscript
Click here to access/download;Manuscript;LRTK.revised.clean.final.docx

LRTK: A platform agnostic toolkit for linked-read analysis of both human genome and metagenome

Chao Yang[1†], Zhenmiao Zhang[1†], Yufen Huang[2,3], Xuefeng Xie[4], Herui Liao[5], Jin Xiao[1], Werner Pieter Veldsman[1], Kejing Yin[1], Xiaodong Fang[3,4*], Lu Zhang[1,6*]

[1]Department of Computer Science, Hong Kong Baptist University, Hong Kong SAR, Hong Kong

[2]BGI Research, Shenzhen 518083, China

[3]BGI Genomics, Shenzhen 518083, China

[4]BGI Research, Sanya 572025, China

[5]Department of Electrical Engineering, City University of Hong Kong, Hong Kong SAR, Hong Kong

[6]Institute for Research and Continuing Education, Hong Kong Baptist University, China

[†]These authors contributed equally to this work.

*To whom correspondence should be addressed: E-mail:

fangxd@genomics.cn ,

ericluzhang@hkbu.edu.hk.

Abstract

Linked-read sequencing technologies generate high-base quality short-reads that contain extrapolative information on long-range DNA connectedness. These advantages of linked-read technologies are well-known and have been demonstrated in many human genomic and metagenomic studies. However, existing linked-read analysis pipelines (e.g., Long Ranger) were primarily developed to process sequencing data from the human genome and are not suited for analyzing metagenomic sequencing data. Moreover, linked-read analysis pipelines are typically limited to one specific sequencing platform. To address these limitations, we present the Linked-Read ToolKit (LRTK), a unified and versatile toolkit for platform agnostic processing of linked-read sequencing data from both human genome and metagenome. LRTK provides functions to perform linked-read simulation, barcode sequencing error correction, barcode-aware read alignment and metagenome assembly, reconstruction of long DNA fragments, taxonomic classification and quantification, as well as barcode-assisted genomic variant calling and phasing. LRTK has the ability to process multiple samples automatically, and provides users with the option to generate reproducible reports during processing of raw sequencing data and at multiple checkpoints throughout downstream analysis. We applied LRTK on linked-reads from simulation, mock community and real datasets for both human genome and metagenome. We showcased LRTK's ability to generate comparative performance results from preceding benchmark studies and to report these results in publication-ready HTML document plots. LRTK provides comprehensive and

flexible modules along with an easy-to-use Python-based workflow for processing linked-read sequencing datasets, thereby filling the current gap in the field caused by platform-centric genome-specific linked-read data analysis tools.

Keywords: linked-read sequencing, 10x Genomics, TELL-Seq, stLFR, metagenome, human genome

Introduction

Linked-read sequencing generates short-reads with high base quality and extrapolative information on long-range DNA connectedness, which has led to significant advancements in human genome and metagenome research [1–4]. It circumvents the typical lack of long-range DNA information in short-read sequencing, and the high error rates and large initial DNA load requirements of long-read sequencing (e.g., Oxford Nanopore and Pacific Bioscience). These advantages of linked-read sequencing are invaluable when dealing with challenging cases of low-input clinical samples, such as cancer tissues or infectious disease samples. Linked-read technology furthermore promotes haplotype construction and the detection of complex structural variations [5], and its relatively low cost enables the application in large cohort studies.

Linked-read sequencing platforms, such as 10x Genomics linked-read (10x Genomics; now discontinued) and the newly developed single-tube long fragment read (stLFR) [6] and transposase enzyme linked long-read sequencing (TELL-Seq) [7], hold much promise in the metagenomics area. The hidden long-range information they provide enables local assembly of co-barcoded reads and thus significantly increases the number of high-quality metagenome-assembled genomes (MAGs) [8]. In longitudinal sequencing datasets [3,9,10], the barcodes associated with linked-read promote the phasing of genomic variants and refine the identification of intra-host evolution of gut microbiota. In some complex environments, such as soil, linked-read

sequencing has been shown to aid in the investigation of the involved microbial genomes [11,12]. However, existing linked-read pipelines are mainly designed for use with the human genome, which points to an urgent need for appropriate metagenome analysis toolkits.

Despite the limitations that genome specificity places on research scope, linked-read sequencing has already been successfully applied to many human genomic studies [2,13–15]. Some toolkits have been developed to facilitate the investigation of the human genome. For example, Long Ranger [16] performs barcode-aware read alignment and implements modules for genomic variant calling and phasing using 10x Genomics linked-reads. Tell-Sort [7] is a Docker-based pipeline to process TELL-Seq linked-reads, for genomic variants detection and phasing. stLFR has found application in a customized pipeline that has been developed to first convert its raw reads into a 10x-compatible format, after which Long Ranger is applied for downstream analysis. This pipeline, however, typically requires a lot of random-access memory and its data format conversion procedure is time-consuming. In addition, the format conversion may induce the loss of barcode specificity as the type of barcodes decreases dramatically. This occurs when the stLFR and TELL-Seq linked-reads are typically converted into a 10x-compatible format to run Long Ranger and Supernova (https://github.com/BGI-Qingdao/stlfr2supernova_pipeline and https://sagescience.com/wp-content/uploads/2020/10/TELL-Seq-Software-Roadmap-User-Guide-2.pdf). Beyond the preceding examples that validate the inherent usefulness of linked-read technologies, our search of the literature furthermore

revealed a lack of unified and open-source toolkits that are compatible with the different linked-read platforms.

To this end, we present Linked-Read ToolKit (LRTK), a unified and versatile toolkit to analyze both metagenomic and human genome linked-read sequencing data derived from any of the three major linked-read sequencing platforms. LRTK delivers a suite of utilities to simulate linked-read sequencing data, barcode sequencing error correction, barcode-aware alignment and metagenome assembly, reconstruction of long DNA fragments, and genomic variant detection and phasing. LRTK is open-source, automatically produces HTML reports to summarize quality statistics as part of its pipeline, and generates publication-ready visualizations. We applied LRTK to linked-reads from simulation, mock community and real datasets to evaluate the performance of different technologies and demonstrate its potential applications. Our results show that LRTK performs favorably on human genome linked-read sequencing data when compared to the pipelines designed specifically for a single platform and that it adequately allows for data analysis of metagenomic sequencing data.

Results

Overview of LRTK

We developed Linked-Read ToolKit (which we refer to as LRTK) that takes raw linked-reads from 10x Genomics, stLFR, or TELL-Seq technologies, and analyzes these inputs in a multi-step checkpointed pipeline that ends with the generation of user-friendly

reports. LRTK consists of two main sections to process metagenomic and human genome linked-read sequencing data from mainstream technologies **(Figure 1)**. For metagenomic sequencing, LRTK includes the representative human gut microbial genomes from the UHGG [17] project as its default reference genomes. We modified EMA [18] to perform barcode-aware alignment to be compatible with different platforms. To further reduce spurious mapping errors, LRTK eliminates the genomes if their coverage is below 40%. It performs genomic variation calling and phasing for these candidate genomes if the single nucleotide variant (SNV) calling function is enabled. In addition, LRTK is equipped with the functions to perform barcode-aware metagenome assembly and reconstruct MAGs from the metagenomic linked-read sequencing data.

In the human genome processing section, LRTK directly aligns linked-reads to the human reference genome using the same modified EMA followed by marking PCR duplicates for each barcode. LRTK reconstructs long DNA fragments through greedy extension based on the alignment coordinates of co-barcoded linked-reads [19]. After reads alignment, LRTK offers users an option to select one of the well-known tools for variant calling, including FreeBayes [20], SAMtools [21], GATK [22] for SNV and small INDEL (Insertion and Deletion, < 50 bps) calls; and Aquila [23], LinkedSV [24] and VALOR2 [25] for structural variant calls (SVs) (>50 bps). For variant phasing, LRTK utilizes HapCUT2 [26] and WhatsHap [27] to explore phasing blocks for SNVs and small INDELs **(Table S1)**. Further details are available in the **Methods** section.

Data Description

We incorporated one simulated dataset (S1), one dataset from a mock community (B1), and two real datasets from human gut microbiomes (D1 and D2) to evaluate metagenomic sequencing analysis module of LRTK **(Table S2)**. For dataset S1**,** we simulated 11.9 Gb and 9.2 Gb linked-reads from 10x Genomics and stLFR for 40 complete bacterial genomes from NCBI RefSeq database, with lognormal abundance distribution (**Methods**), respectively **(Table S3)**. The dataset B1 was generated from ATCC-MSA-1003, containing 20 bacterial species with abundances varying from 0.02% to 18% **(Table S4).** The sequencing data volumes are 37.7 Gb, 111 Gb and 55 Gb for 10x Genomics, stLFR and TELL-Seq linked-reads, respectively. For the two real metagenomic datasets, D1 consists of 16 longitudinal human gut metagenomic sequencing datasets from a single individual, sequenced with an average of 24 Gb linked-reads on 10x Genomics platform [3]. The other dataset D2 contains around 99 Gb stLFR linked-reads from human gut metagenome[28]. For the human genome analysis section, we collected the linked-read sequencing datasets from NA12878, NA24143, NA24149 and NA24385 (**Table S2).** In addition, we performed linked-reads down-sampling to ensure fair comparisons among the three sequencing technologies. We down-sampled around 20 Gb metagenomic linked reads from ATCC-MSA-1003 for each platform. Similarly, around 110 Gb (~35X) linked reads from NA12878 were extracted for the three technologies **(Table S3)**.

LRTK supports multiple linked-read sequencing technologies

LRTK can handle linked-reads from various sequencing technologies, including but not limited to 10x Genomics, stLFR, or TELL-Seq. Initially, LRTK converts the raw linked-reads into a unified FASTQ format, which contains a new field “BX:Z:” to include 16 bps (10x Genomics linked-read), 18 bps (TELL-Seq) and 30 bps (stLFR) barcode sequences **(Figure S1)**.

Sequencing errors are often enriched at the start or end of linked-reads, where barcode sequences are typically found. LRTK includes functions to correct potential sequencing errors in barcodes. After error correction, there are approximately 94.8% and 94.1% of barcode sequences on the whitelist of 10x Genomics linked-reads for NA12878 and ATCC-MSA-1003 **(Table S2)**, respectively. The performance is comparable to the results obtained from Long Ranger (94.4% for NA12878 and 93.6% for ATCC-MSA-1003). The corresponding rates are slightly lower for stLFR linked-read (85.4% for NA12878 and 90.7% for ATCC-MSA-1003) compared to 10x Genomics linked-read. As there is no whitelist for TELL-Seq, we could not perform the analysis for NA12878 and ATCC-MSA-1003 TELL-Seq linked-read sequencing data.

LRTK reconstructs long DNA fragments by barcode deconvolution

The quality of DNA sequencing library may significantly affect the performance of metagenome assembly [29], human genome assembly [19] and structural variant calling

[30]. To evaluate the quality of linked-read sequencing libraries, we reconstructed the input long DNA fragments for both human genome and metagenomic sequencing data based on co-barcoded read alignments (**Methods**). We also calculated several key statistics to comprehensively compare the libraries from different linked-read sequencing technologies [19]. The statistics include average coverage of short-reads per fragment (C_R), average physical coverage of the genome by long DNA fragments (C_F), number of fragments per partition/beads ($N_{F/P}$), unweighted and length-weighted average DNA fragment length (μ_{FL} and $W\mu_{FL}$) **(Figure S2)**.

For NA12878 (**Table S3**), LRTK detected approximately 7.34, 1.95 and 3.60 fragments per barcode and achieved μ_{FL} of 45.9 kb, 16.46 kb and 54.78 kb for the libraries from 10x Genomics, stLFR and TELL-Seq, respectively **(Figure 2B)**. For ATCC-MSA-1003 (**Table S3**), stLFR linked-reads yielded the lowest $N_{F/P}$ ($N_{F/P}$ =1.48), while TELL-Seq linked-reads yielded a slightly higher number ($N_{F/P}$ =4.59). Both values were much lower than that obtained from 10x Genomics ($N_{F/P}$ =13.6) **(Figure 2A)**, indicating that stLFR and TELL-Seq have superior performance in deconvolving linked-reads from different species.

LRTK enables metagenome taxonomic quantification and genomic variant detection using barcode-aware alignment

Previous studies have amply demonstrated the huge potential of linked-reads in metagenomic researches [3,8,9,11]. Here, we evaluated the performance of linked-reads in detecting taxonomic abundance and genomic variants using LRTK. In LRTK,

we developed a computational pipeline to detect and quantify microbes using microbial reference genomes. LRTK can better identify the involved microbes based on their genome coverage (F1=0.81) than the existing k-mer based tools: Bracken [31] (F1=0.13), and KMCP [32] (F1=0.67); and marker gene based tools: MetaPhlAn2 [33] (F1=0.49), and MIDAS 2 [34] (F1=0.72), on the simulated stLFR linked-reads (**Figure 3A**). For the stLFR linked-reads from ATCC-MSA-1003, LRTK also demonstrated a superior performance (F1=0.78) than Bracken (F1=0.22), KMCP (F1=0.59) and MIDAS 2 (F1=0.6), but was inferior to MetaPhlAn 2 (F1: 0.95). We also evaluated the LRTK performance of taxonomic quantification by comparing the benchmark microbial abundance (from simulation and ATCC-MSA-1003) and the predicted values using Spearman correlation coefficient (SCC). For the simulated dataset, LRTK (SCC=0.99), MIDAS 2 (SCC=0.98), and KMCP (SCC=0.95) exhibited superior performance. For stLFR linked-reads in ATCC-MSA-1003, LRTK (SCC=0.97), Bracken (SCC=0.97), and MetaPhlAn 2 (SCC=0.97) were the top performers. Comparable findings were also observed in the linked-reads data generated from other technologies in simulation **(Figure S3A)** and ATCC-MSA-1003 **(Figure S3B and S3C).** In addition, LRTK also enables the identification of microbial SNVs and could reconstruct their potential haplotypes based on co-barcoded linked-reads. Comparing different SNV callers implemented in LRTK, we discovered that approximately 176,891 SNVs were jointly detected by FreeBayes [17], SAMtools [18] and inStrain [35] on stLFR linked-reads for ATCC-MSA-1003 **(Figure 3B).** These SNVs account around 76%, 80% and 21% of the total SNVs

detected by FreeBayes and SAMtools and inStrain, respectively **(Figure 3B)**. More than 50% of SNVs from inStrain can not be detected by FreeBayes and SAMtools, suggesting inStrain is the most sensitive tool to detect microbial SNVs. We further applied LRTK to explore the taxonomic composition and genomic variants for each sample from a longitudinal linked-read metagenomic dataset **(D1, Table S2)** [3]. Using multiple related samples, LRTK identified a genome-wide mirrored allele imbalance **(Methods)** for species Alistipes finegoldii: the minor alleles of some SNVs at some timepoints became the major alleles at another timepoints, which may suggest intra-host strain evolution over time **(Figure 3C and D)**.

LRTK promotes metagenome assembly using linked-reads with high barcode specificity

In our previous study, we have shown metagenome assembly on linked-reads could improve assembly length and the number of near-complete MAGs[28]. We compared the performance of two well-known short-read assemblers: MEGAHIT [36] and metaSPAdes [37], and three linked-read assemblers: Athena [38], CloudSPAdes [39] and Pangaea [28] using the linked-reads from ATCC-MSA-1003 and simulation **(Table S3).** Among them, LRTK (Pangaea module) achieves the highest NA50 values for stLFR (NA50=372kb) and TELL-Seq (NA50=339kb) linked-reads, respectively **(Figure 4A)**. As Pangaea is not compatible with 10x Genomics linked-reads, Athena becomes the best tool on 10x Genomics in terms for NA50 (Athena:146 kb; cloudSPAdes: 45 kb; metaSPAdes: 17 kb and MEGAHIT:79 kb). We also examined

the assembly quality of each species in ATCC-MSA-1003, and observed that Pangaea always obtained the highest NA50 and N50 values for stLFR and TELL-Seq linked-reads while the assembly length is comparable **(Figure S4A)**. For simulated linked-reads, Pangaea and Athena also show superior performances than the other metagenome assemblers (**Figure S4B and Figure S5**). We applied LRTK to a human gut metagenomic dataset (D2) [28] and found two contigs were circularized, which showed near perfect collinearity with the closest reference genomes **(Figure 4B)**. LRTK could also automatically perform contig binning using MetaBAT 2 [40] after metagenome assembly. In the D2 data set, LRTK recovers 24 near-complete, 7 high-quality and 52 medium-quality bins for D2 **(Methods, Figure 4C-E)**. The superior assembly performance we have observed affirms the efficacy of linked-read sequencing technologies on metagenome assembly.

LRTK provides best practices for human genomic variant detection and phasing

Previous studies have shown that genomic variant detection [41] and phasing could benefit from the high base quality and long DNA fragments provided by linked-read[42]. Here, we benchmarked the computational tools for human genomic variant detection and phasing, and demonstrated a best practice guideline for human genome linked-read analysis using LRTK. We applied the modified version of EMA to align linked-reads from different sequencing technologies to the human reference genome (GRCh38). We first benchmarked the commonly used tools FreeBayes [20], GATK

[22] and SAMtools [21] to detect SNVs and small INDELs. Among them, GATK (F1=0.90) achieves the best average F1 score across the three technologies in detecting SNVs, followed by SAMtools (F1=0.89) and FreeBayes (F1=0.87) **(Figure 5A)**. For small INDEL calling, SAMtools demonstrated the best average F1 score (SAMtools: F1= 0.71; GATK: F1= 0.65; FreeBayes: F1 = 0.59) while GATK has a better recall value (SAMtools: average recall = 0.87; GATK: average recall = 0.91; FreeBayes: average recall = 0.79) (**Figure 5B)**. We then compared the linked-read phasing tools, HapCUT2 [26] and WhatsHap [27], for genomic variant phasing. We observed that HapCUT2 **(Figure 5C and D)** achieved longer average length of phasing blocks (HapCUT2: 23.2 Mb and WhatsHap: 0.4 Mb) and a higher average phased heterozygous SNV rate compared to WhatsHap (HapCUT2: 0.99 and WhatsHap: 0.63). We only evaluated the performance of SV detection tools on 10x Genomics linked-reads of NA24385 because some of the tools do not support stLFR and TELL-Seq. As the recall values illustrated in **Figure 5F**, Aquila has a higher recall value for 50 bps – 1 kb deletions (Aquila: 0.82; LinkedSV: 0.54; Long Ranger: 0.43). LinkedSV [24] and Long Ranger perform better in detecting deletions longer than 1 kb (Aquila: 0.29; LinkedSV: 0.71; Long Ranger: 0.72). Aquila also shows a better performance than Pamir [43] and PopIns2 [44] to detect insertions (Aquila: 0.35; Pamir: 0.07; PopIns2: 0.01) (**Figure 5G**). We also applied LRTK to linked-read from a trio (D3; Father: NA24149; Mother: NA24143; and Son:NA24385) and found it demonstrated excellent variant phasing performance and identity-by-descent segment detection in pairwise samples **(Figure 5E)**.

LRTK provides flexible commands to process sequencing data

A primary advantage of LRTK is its flexible, user-defined settings for different tasks. Users have a choice to run each LRTK module independently and generate separate results for each module. For instance, the MKFQ function could be independently used to simulate linked- reads from 10x Genomics and stLFR platforms. For some functions, LRTK provides multiple tools for users to choose. For microbial SNVs detection, LRTK offers users a choice between SAMtools [21], FreeBayes [20], and inStrain [35]. To accommodate user-specific requirements on LRTK command, LRTK also allows users to set different parameters and save the results for further comparison.

LRTK provides automated analysis and user-friendly reports

LRTK provides an automated analysis pipeline, starting from raw linked-reads to performing diverse data analysis and generating publication-ready visualizations. Specifically, LRTK investigates different types of data features, calculates their corresponding statistical indicators, and presents them together in a HTML report. Taking the aforementioned longitudinal linked-read sequencing dataset D1 as an example, LRTK generates a systematic summary of the input sequencing libraries and analysis outcomes of each step. Firstly, LRTK produces summary of the FASTQ files obtained from the read quality control tools **(Figure S6A-B)**. After aligning reads to the reference genomes, LRTK calculates the key parameters for the sequencing libraries and reconstructed long DNA fragments **(Figure S6C-D)**. For microbial

species and genomic variants, LRTK generates basic statistics and presents them using concise distribution plots **(Figure S6E-F)**. For downstream analysis, LRTK conducts principal components analysis on the relative abundance profiles from multiple samples and performs clustering analysis on allele frequencies of microbial SNVs **(Figure S6G)**. Similar reports could be produced for human genome analysis using LRTK **(Figure S7)**.

Evaluation of the computational resources required for LRTK

We would focus on evaluating computational resources required by LRTK for linked-read preprocessing and alignment. The computational resources needed for genomic variants calling and phasing depend on the chosen software, while metagenome sequencing data typically demand fewer computational resources due to the lower volume of required sequencing reads. We extracted approximately the same data volume (around 35X, 110G bases) of linked-reads from NA12878 for 10x Genomics, stLFR and TELL-Seq and aligned these reads to the human reference genomes. As shown in **Figure S8**, LRTK required around 26.3, 37.6 and 19.8 hours to align reads from the three platforms with a maximum memory usage of around 74Gb using 64 threads. In comparison to Long Range, LRTK reduced memory requirements (maximum memory for Long Ranger: >100 Gb) at the expense of increased running time. The experiments were carried out on the computational nodes with Xeon Gold 6330 @ 2.0GHz (2S/28C) / 1T RAM / 900GB SWAP.

Discussion

Seeing that multiple linked-read sequencing technologies are extensively utilized in scientific studies, a platform agnostic linked-read processing tool would be an intuitive solution to ensure reproducibility and robustness. Unfortunately, a cross-platform software solution is currently unavailable to the research community. Accordingly, we introduce LRTK, a unified and versatile computational framework to efficiently process sequencing data from 10x Genomics, stLFR and TELL-Seq technologies. LRTK includes separately invokable commands to perform linked-read simulation, barcode sequencing error correction, barcode-aware alignment and metagenome assembly, reconstruction of long DNA fragments and other barcode-assisted genomic variant calling and phasing. LRTK also provides automated and complete analysis, from raw data QC through advanced downstream analysis to generation of publication-ready visualization. LRTK is also open source, allows easy integration with other scientific pipelines.

Short-read sequencing has led to significant discoveries in large-scale population sequencing studies within the human genome and metagenome fields [45,46]. However, the limited sequencing length poses challenges for tasks like complex structural variant detection in the human genome and ribosomal RNAs assembly from metagenomic data. Long-read sequencing, such as single-molecule real-time sequencing by Pacific Biosciences (PacBio), nanopore sequencing by Oxford Nanopore Technologies (ONT), are gaining attentions for their improved performance

in addressing these challenges. Despite their advantages, long-read sequencing can be costly for large-cohort studies. Linked-read sequencing technologies, offer a cost-effective solution for large population studies by attaching barcodes to short-reads to establish long-range DNA connectedness. Previous researches have shown 10x Genomics linked-reads have facilitated the discovery of complex structural variants, such as chromothripsis [5], large rearrangements [47] and tandem duplications [14], in cancer studies. Additionally, 10x Genomics linked-reads have enhanced metagenome assembly contiguity [38] and enabled haplotype construction from time-series metagenomic data [3]. The newly emerging stLFR and TELL-Seq have further improved barcode specificity aiming for one fragment per barcode. These advancements have shown superior performance in distinguishing linked-reads from different species [28]. Our study benchmarks tools developed for linked-read sequencing and integrates them into LRTK to support academic applications. Furthermore, LRTK is the only tool that can accept linked-reads from all three platforms and avoid loss of barcode specificity.

Our finding revealed that in practice, the $N_{F/P}$ values for stLFR and TELL-Seq were much lower than those obtained from 10x Genomics. According to Bishara, Alex, et al. [38], assembly using 10x Genomics linked-reads often struggles with high-copy genomic repeat regions. The long-fragment barcoding approaches adopted by stLFR and TELL-seq has notably reduced the number of long fragments per barcode. This reduction could improve the assembly of microbial genomes containing high-copy repeats. In our previous study, we demonstrated that the characteristics of

the reconstructed long DNA fragments (E.g. C_R, C_F et al.) can significantly affect human genome assembly [19] and structural variant calling [30]. The improved stLFR and TELL-Seq may also further refine the structural variants calling and variant phasing on human genome. We anticipate that future linked-read sequencing technologies will improve both DNA-extraction techniques and long-fragment barcoding approaches to achieve one fragment per barcode.

Finally, it is worth mentioning that LRTK has the potential to be extended to handle other types of linked-read sequencing technologies. For example, in 2017, Illumina introduced the bead-based barcode partitioning in a single tube to phase human genomes. It further proposed the complete long-read technology for complex genomes in 2022 [48]. Additionally, Meier J I, *et al.* developed Haplotype tagging to investigate the butterfly species [49]. Redin D, *et al.* recently introduced a novel library preparation method for high throughput barcoding of short reads [50]. The new single-cell metagenomic sequencing technologies employ highly accurate barcoded reads and provide inferable long-range information, which could potentially be used in combination with current linked-read technology in future studies [51]. We are actively developing LRTK to incorporate these technologies.

Methods

Data collection

We included one simulated metagenomic dataset (S1), one mock microbial community (B1) and two human gut metagenomic sequencing datasets (D1, D2) to

evaluate the performance of the metagenomic data analysis section of LRTK **(Table S2)**. For S1, we simulated 11.8 Gb 10x Genomics and 9.2 Gb stLFR linked-reads using LRTK-SIM [29] for 40 complete bacterial genomes extracted from the NCBI RefSeq database (December, 2023) with the same abundances (Log Normal Distribution) **(Table S5)**. LRTK-SIM allows flexible parameters, such as C_F, C_R, $N_{F/P}$, μ_{FL} and $W\mu_{FL}$, to simulate linked-read data. We set the parameters, "C_F =500, C_R =0.2, $N_{F/P}$ =16, μ_{FL} =20" to simulate 10x Genomics linked-reads while "C_F =500, C_R =0.2, $N_{F/P}$ =1, μ_{FL} =20" to simulate stLFR linked-reads. The dataset B1 contains linked-reads from a mock microbial community ATCC-MSA-1003, from three different platforms (SRR12283286 for 10x Genomics, and PRJNA875547 for stLFR and TELL-Seq). The ATCC-MSA-1003 mock community is composed of 20 bacterial species represented at staggered abundances—specifically, five species at 18%, 1.8%, 0.18%, and 0.02% abundance levels, respectively. The complete descriptions of the mock metagenomic sample, including the genome sizes, individual bacterial species, and their corresponding reference sequence accessions, have been included in **Table S4.** The D1, contains 16 longitudinal human gut metagenomic 10x Genomics linked-read sequencing datasets from one single individual (accession number: SRP323279) **(Table S2)**. The D2 was downloaded from the China National GeneBank (CNGB) under project CNP0003432. It contains around 99 Gb stLFR human gut metagenomic sequencing data. In the human genome section, we collected the available linked-read sequencing data for the three technologies from NA12878, NA24143, NA24149 and NA24385. The detailed information has been included in

Table S2. For ATCC-MSA-1003 and NA12878, we further performed linked-read down-sampling at the barcode level by using in-house scripts.

Data preprocessing

LRTK converts the raw linked-reads from 10x Genomics, stLFR and TELL-Seq into a unified FASTQ format (Figure S1) and corrects potential sequencing errors in barcodes. For 10x Genomics and stLFR linked-read, the barcodes are aligned to their respective barcode whitelists using the "BWA aln" command. The barcodes with fewer than 2 mismatches in alignments are then corrected as the corresponding barcodes in the whitelist. LRTK adopts the approach described by Chen *et al.* [7] to correct barcode errors for TELL-Seq due to the lack of barcode whitelist. In general, LRTK tallies the supporting reads for each barcode derived from TELL-Seq linked-reads and distinguishes between barcodes with a single supporting read and those with multiple supporting reads. It then corrects possible sequencing errors in barcodes that initially had one mismatch by comparing them to those with multiple supporting reads. The linked-reads in the unified FASTQ file are then provided to fastp [52] to remove adapter sequences and low-quality reads. For metagenomic sequencing data, the sequencing reads are aligned to the human genome first and only unmapped reads are used for subsequent analysis.

Metagenome assembly and contig binning

We evaluated the performance of five metagenome assemblers on linked-reads: Athena [38], Pangaea [28], cloudSPAdes [39], MEGAHIT [36] and metaSPAdes [37], and observed superior performance of Pangaea on stLFR and TELL-Seq sequencing data. Therefore, for LRTK, we chose Pangaea as the default assembler to assemble linked-reads metagenomic sequencing data for LRTK. After the initial assembly, LRTK extracts the circular contigs that are at least 1Mb in length. The uncircularized contigs are grouped into MAGs using MetaBAT 2 [40]. According to standard criteria of the minimum information about MAGs [53], they could be classified into near-complete (completeness > 90%, contamination < 5%, and could be detected 5S, 16S, and 23S rRNAs, and at least 18 tRNAs), high-quality (completeness > 90%, and contamination < 5%), medium-quality (completeness ≥ 50%, and contamination < 10%), and low-quality (the other MAGs).

Barcode-aware read alignment

For human genome sequencing data, LRTK utilizes EMA, a barcode-aware alignment approach, to map high-quality 10x Genomics linked-reads to the human reference genome [54]. We further modified EMA to be compatible with the barcodes from stLFR (30 bps) and TELL-Seq (18 bps). LRTK marks PCR duplicates for each barcode using the "BARCODE_TAG" parameter in Picard (https://broadinstitute.github.io/picard/). The alignment files are then sorted according to the genomic coordinates of alignments for further analysis.

For metagenomic sequencing data, we developed a tiered alignment approach to align the linked-reads to microbial genomes using the aforementioned modified EMA. The default reference genomes for the human gut metagenome were downloaded from UHGG [17]. For non-gut samples, we used the GTDB [55] as the reference database, but users could also incorporate their own custom database.

In the first round of alignment, linked-reads were directly mapped to the 4,724 representative genomes in UHGG (by default). For each genome, LRTK calculates the coverage rate and covered base, and then extracts genomes with “coverage rate > 40% and covered base > 500 kb”. These genomes will be used as the candidate reference genomes for the second round of alignment.

In the second round of alignment, linked reads are only mapped to those candidate reference genomes to reduce multiple alignment errors. For each candidate genome, LRTK partitions it into 10 kb windows and calculated the number of mapped reads on each window. The mapped reads are categorized into two types: unique-mapped reads (U) and multi-mapped reads (M), which will be processed separately. LRTK uses the formulas below to determine the total read count of a window (RC(W)), unique-mapped read count ($RC(U)$) and multi-mapped read count ($RC(M)$), where l is the window size.

$$RC(W) = RC(U) + RC(M)$$

$$RC(U) = \frac{U}{l}$$

$$RC(M) = \sum_{i=1}^{M} \omega_i * \{M\}/l$$

A coefficient ω is introduced for multi-mapped reads. When a multi-mapped in M is aligned to N different genomes, ω is calculated using the following formula:

$$\omega = U/\sum_{i=1}^{N} RC(U)$$

LRTK removes the windows with an extreme number of reads (> <), and calculates the average depth using the remaining windows as the depth of the corresponding genome. The relative abundance is then calculated by aggregating the average depth of all identified genomes.

In the experiments, we further evaluated the performance (F1 score, precision and recall) of LRTK, KMCP [32], and MIDAS 2 [34] to identify microbes and quantify their abundances. The reference databases were prepared using the genome sequences from the GTDB database for the three tools. We also compared LRTK with the widely used taxonomic classification tools: Bracken [31] and MetaPhlAn 2 [33] using their default databases.

Reconstruction of long DNA fragments

Initially LRTK extracts unique-mapped co-barcoded paired-end short-reads from the alignment file to evaluate the distribution of insert sizes (with a mean of μ_{PE} and a

standard deviation of σ_{PE}). Alignments are removed if the distance between two reads in a paired-end read (R1 and R2) exceed a certain threshold, that is,

$$Dis(\text{R1}, \text{R2}) > \mu_{PE} + 3 * \sigma_{PE}.$$

The remaining paired-end reads are used as seeds and extended to both directions to connect with other seeds sharing the same barcode until no more eligible seeds can be found within a specified distance (200 kb by default). All of these co-barcoded reads are considered to be derived from the same long DNA fragment.

Identify and phase genomic variants

LRTK supports multiple popular variant detection tools and adheres to best practices for linked-read sequencing. For human genome sequencing data, LRTK provides FreeBayes [20], SAMtools [21] and GATK [22] to call SNVs and small INDELs. FreeBayes is a Bayesian genetic variant detection tool designed to identify SNPs, INDELs, multinucleotide polymorphisms, and more complex events. It is straightforward, easy to use and timesaving. GATK, while also employing a Bayesian framework, enhances its detection capabilities for insertions and deletions through specialized techniques such as read realignment and base recalibration. Although these steps add value, they also increase GATK's computational runtime. In contrast, SAMtools uses a hidden Markov model for the identification of small variants and has demonstrated robust performance across various studies. The available phasing tools

include HapCUT2 [26] and WhatsHap [27]. HapCUT2 demonstrated excellent performance to phase heterozygous SNVs within a diploid context, such as the human genome. WhatsHap, however, introduces an innovative clustering and threading approach that delivers precise phasing in polyploid genomes. Having obtained the phased SNVs, we used PhaseME [56] to calculate the phasing block and phasing rate to assess the phasing quality. Subsequently, we used hap-ibd [57] to detect pairwise identity-by-descent segments across multiple samples. By default, large SVs were identified using Aquila[23]. However, Users have the flexibility to select other tools such as LinkedSV [24] and VALOR2 [25] for SV analysis. LinkedSV leverages barcode overlapping, read depth, paired-end signals and local assembly to detect deletions, although it currently lacks support for insertion detection. In contrast, the assembly-based tool Aquila is capable of detecting both insertions and deletions, offering a comprehensive solution.

For metagenome sequencing data, LRTK offers three metagenome SNV callers: FreeBayes [20], inStrain [35] and SAMtools [21]. The SNV phasing was performed on high abundance species using WhatsHap [27] with the inferred ploidy. LRTK also selected SNVs located on certain high abundance species and compared the SNV profiles across multiple samples. Based on the allele frequency of SNVs, we used an unsupervised clustering method to detect potential mirrored allele imbalance (MAI) events for pairwise samples. MAI is derived from the notion of mirrored subclonal allelic imbalance observed in cancer genomes, where the maternal allele is gained or lost in one tumor region and the paternal allele is gained or lost in a different region

[58]. In metagenome, MAI means that the reference allele is gained or lost at one time point while the altered allele (minor) allele is gained or lost at a different time point. To detect MAI, one sample was chosen as the reference for all the contigs. For each species which was present in at least two sample, the minor allele was determined based on the reference sample. The BAF was calculated as the ratio of the minor allele count to the total allele count. For each interested species, BAFs of SNPs (minimum supported read number exceeding 2) were extracted from each sample and merged into a combined SNP set. Based on the combined SNP set, BAF values for the interested sample were then compared with the BAFs of the reference sample. We used the k-means clustering method to separate the SNVs into distinct groups using the minor allele frequency matrix. The number of clusters were determined by using the Calinski-Harabasz index.

Downstream analysis and HTML-based visualization

The human genome analysis report begins with generating the FASTQ quality control statistics during the preprocessing step. The barcode-aware alignment step presents the distribution of several key statistics about the DNA sequencing library, including the number of fragments per barcode, fragment length and average read coverage per fragment. It also includes information about the sequencing coverage and insert size. The variant calling step summarizes the number of SNVs, small INDELs and large SVs and illustrates their distributions.

Similarly, the metagenome analysis report illustrates the QC results for preprocessing, alignment and variant calling steps. Additionally, it includes an optional report about the automatic analysis of multiple related samples. The report shows the distribution of high abundant species across multiple samples, and uses a principal component analysis plot to visualize the divergence across them. The report also depicts the distribution of SNVs of these high abundant species, and the minor allele frequency distribution in pairwise samples.

Code Availability and Requirements

Source code is available at https://github.com/ericcombiolab/LRTK. LRTK can be downloaded as a packaged conda environment (https://anaconda.org/bioconda/lrtk).

Project name: Linked Read ToolKits project

Project home page: https://github.com/ericcombiolab/LRTK

Operating system(s): Linux and macOS

Programming language: C and Python

Other requirements: Conda, Python 3.6 or higher

License: MIT

RRID: SCR_023945

Data Availability

We included one simulated metagenomic dataset (S1), one mock microbial community (B1) and two human gut metagenomic sequencing datasets (D1, D2) in the metagenome section and the human genome sequencing data for four samples (D3) in the human genome section **(Table S2).**

B1: The mock microbial community B1 contains the 10x Genomics, stLFR and TELL-Seq sequencing data for ATCC MSA-1003 mock community. These datasets were obtained from the NCBI with the following accession numbers: SRR12283286 for 10x Genomics, and PRJNA875547 for stLFR and TELL-Seq sequencing technologies.

D1: The first real metagenomic dataset (D1), consisting of longitudinal 10x Genomics linked-read sequencing data, was downloaded from the NCBI under accession number SRP323279.

D2: The second real metagenomic dataset (D2), containing deep stLFR sequencing data, was downloaded from the China National GeneBank (CNGB) under project CNP0003432.

D3: The human genome dataset D3 contains the linked-read sequencing data for NA12878, NA24143, NA24149 and NA24385. The 10x Genomics and stLFR sequencing data was downloaded following the links in **Table S2**. For TELL-Seq sequencing data, we only obtained the raw TELL-Seq data from NA12878 and

NA24385 from SRA database under the accession SRX7264479 and SRX7264481, respectively.

Abbreviations

stLFR: single-tube long fragment read

TELL-Seq: transposase enzyme linked long-read sequencing

SNV: single nucleotide variant

INDEL: small insertion and deletion

SV: structural variation

CRediT authorship contribution statement:

Chao Yang: Writing - original draft, and preparing figures and tables, preparing source codes; **Zhenmiao Zhang:** preparing source codes; **Xuefeng Xie:** Consolidating resources; **Yufen Huang:** Consolidating resources; **Herui Liao:** preparing source codes; **Werner P Veldsman:** Revising - original draft, and visualizations; **Jin Xiao:** Revising - original draft, and visualizations; **Kejing Yin:** Supervision; **Xiaodong Fang:** Supervision; **Lu Zhang:** Project administer, Writing – review & editing, Supervision, and funding acquisition.

Declaration of Competing Interest:

The authors declare that they have no known competing financial interests

Acknowledgments:

This research was partially supported by the open project of BGI-Shenzhen, Shenzhen 518000, China (BGIRSZ20220012), the Hong Kong Research Grant Council Early Career Scheme (HKBU 22201419), HKBU Start-up Grant Tier 2 (RC-SGT2/19-20/SCI/007), HKBU IRCMS (No. IRCMS/19-20/D02), the Guangdong Basic and Applied Basic Research Foundation (No. 2021A1515012226), the Science Technology and Innovation Committee of Shenzhen Municipality, China (SGDX20190919142801722) and Shenzhen Science and Technology Innovation Commission (SZSTI) - Shenzhen Virtual University Park (SZVUP) Special Fund Project (No. 2021Szvup135).

Additional Files

Figure S1. LRTK text file specification.

Figure S2. Ideogrammatic definitions of C_R, C_F, $N_{F/P}$, μ_{FL}, and $W\mu_{FL}$ metrics.

Figure S3. Evaluation of taxonomic quantification performance for 10x Genomics and TELL-Seq linked-reads.

Figure S4. Evaluation of assembly performance at species level.

Figure S5. Evaluation of metagenomic assemblers on simulated linked-read data.

Figure S6. Demo reports for metagenomics sequencing.

Figure S7. Demo reports for human genome sequencing.

Figure S8. Computational requirements for linked-reads preprocessing and alignment between LRTK and Long Ranger on NA12878.

Table S1. Bioinformatics tools included in LRTK.

Table S2. Linked-read sequencing data sources.

Table S3. Data descriptions for the simulated and down-sampled linked-reads from ATCC-MSA-1003 and NA12878.

Table S4. Reference genomes for the ATCC-MSA-1003 mock sample.

Table S5. Reference genomes for the simulated dataset.

References

1. Bergström A, McCarthy SA, Hui R, Almarri MA, Ayub Q, Danecek P, et al.. Insights into human genetic variation and population history from 929 diverse genomes. *Science (80-)*. American Association for the Advancement of Science; 2020; doi: 10.1126/SCIENCE.AAY5012/SUPPL_FILE/AAY5012-BERGSTROM-SM.PDF.

2. Dréau A, Venu V, Avdievich E, Gaspar L, Jones FC. Genome-wide recombination map construction from single individuals using linked-read sequencing. *Nat Commun 2019 101*. Nature Publishing Group; 2019; doi: 10.1038/s41467-019-12210-9.

3. Roodgar M, Good BH, Garud NR, Martis S, Avula M, Zhou W, et al.. Longitudinal linked-read sequencing reveals ecological and evolutionary responses of a human gut microbiome during antibiotic treatment. *Genome Res*. Cold Spring Harbor Laboratory Press; 2021; doi: 10.1101/GR.265058.120.

4. Hadi K, Yao X, Behr JM, Deshpande A, Xanthopoulakis C, Tian H, et al.. Distinct Classes of Complex Structural Variation Uncovered across Thousands of Cancer Genome Graphs. *Cell*. Cell Press; 2020; doi: 10.1016/J.CELL.2020.08.006.

5. Spies N, Weng Z, Bishara A, McDaniel J, Catoe D, Zook JM, et al.. Genome-wide reconstruction of complex structural variants using read clouds. *Nat Methods 2017 149*. Nature Publishing Group; 2017; doi: 10.1038/nmeth.4366.

6. Wang O, Chin R, Cheng X, Yan Wu MK, Mao Q, Tang J, et al.. Efficient and unique co-barcoding of second-generation sequencing reads from long DNA

molecules enabling cost effective and accurate sequencing, haplotyping, and de novo assembly. *Genome Res*. Cold Spring Harbor Laboratory Press; 2019; doi: 10.1101/GR.245126.118.

7. Chen Z, Pham L, Wu TC, Mo G, Xia Y, Chan PL, et al.. Ultralow-input single-tube linked-read library method enables short-read second-generation sequencing systems to routinely generate highly accurate and economical long-range sequencing information. *Genome Res*. Cold Spring Harbor Laboratory Press; 2020; doi: 10.1101/gr.260380.119.

8. Siranosian BA, Brooks EF, Andermann T, Rezvani AR, Banaei N, Tang H, et al.. Rare transmission of commensal and pathogenic bacteria in the gut microbiome of hospitalized adults. *Nat Commun 2022 131*. Nature Publishing Group; 2022; doi: 10.1038/s41467-022-28048-7.

9. Huang Y, Jiang P, Liang Z, Chen R, Yue Z, Xie X, et al.. Assembly and analytical validation of a metagenomic reference catalog of human gut microbiota based on co-barcoding sequencing. *Front Microbiol*. Frontiers; 2023; doi: 10.3389/FMICB.2023.1145315.

10. Davila Aleman FD. Microbiome and aging: A study of microbial evolution and community structure across model organisms. Abtract and Metadata. 2022;

11. Tracanna V, Ossowicki A, Petrus MLC, Overduin S, Terlouw BR, Lund G, et al.. Dissecting Disease-Suppressive Rhizosphere Microbiomes by Functional Amplicon Sequencing and 10× Metagenomics. *mSystems*. American Society for Microbiology;

2021; doi: 10.1128/MSYSTEMS.01116-20/SUPPL_FILE/MSYSTEMS.0116-20-S0001.PDF.

12. Tolstoganov I, Pevzner PA, Korobeynikov A. SpLitteR: Diploid genome assembly using linked TELL-Seq reads and assembly graphs. *bioRxiv*. Cold Spring Harbor Laboratory; 2022; doi: 10.1101/2022.12.08.519233.

13. Marks P, Garcia S, Barrio AM, Belhocine K, Bernate J, Bharadwaj R, et al.. Resolving the full spectrum of human genome variation using Linked-Reads. *Genome Res*. 2019; doi: 10.1101/gr.234443.118.

14. Viswanathan SR, Ha G, Hoff AM, Wala JA, Carrot-Zhang J, Whelan CW, et al.. Structural Alterations Driving Castration-Resistant Prostate Cancer Revealed by Linked-Read Genome Sequencing. *Cell*. Cell Press; 2018; doi: 10.1016/J.CELL.2018.05.036.

15. Greer SU, Nadauld LD, Lau BT, Chen J, Wood-Bouwens C, Ford JM, et al.. Linked read sequencing resolves complex genomic rearrangements in gastric cancer metastases. *Genome Med*. Genome Medicine; 2017; doi: 10.1186/s13073-017-0447-8.

16. Zheng GXY, Lau BT, Schnall-Levin M, Jarosz M, Bell JM, Hindson CM, et al.. Haplotyping germline and cancer genomes with high-throughput linked-read sequencing. *Nat Biotechnol 2016 343*. Nature Publishing Group; 2016; doi: 10.1038/nbt.3432.

17. A A, S N, M B, F S, M B, ZJ S, et al.. A unified catalog of 204,938 reference genomes from the human gut microbiome. *Nat Biotechnol*. Nat Biotechnol; 2021; doi: 10.1038/S41587-020-0603-3.

18. Shajii A, Numanagić I, Whelan C, Berger B. Statistical Binning for Barcoded Reads Improves Downstream Analyses. *Cell Syst*. Cell Press; 2018; doi: 10.1016/J.CELS.2018.07.005/ATTACHMENT/8C829484-1F22-47F4-A513-B90845BD41D7/MMC1.PDF.

19. Zhang L, Zhou X, Weng Z, Sidow A. Assessment of human diploid genome assembly with 10x Linked-Reads data. *Gigascience*. Gigascience; 2019; doi: 10.1093/GIGASCIENCE/GIZ141.

20. Garrison E, Marth G. Haplotype-based variant detection from short-read sequencing. 2012; doi: 10.48550/arxiv.1207.3907.

21. Li H, Handsaker B, Wysoker A, Fennell T, Ruan J, Homer N, et al.. The Sequence Alignment/Map format and SAMtools. *Bioinformatics*. Oxford Academic; 2009; doi: 10.1093/BIOINFORMATICS/BTP352.

22. McKenna A, Hanna M, Banks E, Sivachenko A, Cibulskis K, Kernytsky A, et al.. The Genome Analysis Toolkit: A MapReduce framework for analyzing next-generation DNA sequencing data. *Genome Res*. Cold Spring Harbor Laboratory Press; 2010; doi: 10.1101/GR.107524.110.

23. Zhou X, Zhang L, Weng Z, Dill DL, Sidow A. Aquila enables reference-assisted diploid personal genome assembly and comprehensive variant detection based on linked reads. *Nat Commun 2021 121*. Nature Publishing Group; 2021; doi: 10.1038/s41467-021-21395-x.

24. Fang L, Kao C, Gonzalez M V., Mafra FA, Pellegrino da Silva R, Li M, et al.. LinkedSV for detection of mosaic structural variants from linked-read exome and

genome sequencing data. *Nat Commun 2019 101*. Nature Publishing Group; 2019; doi: 10.1038/s41467-019-13397-7.

25. Karaoğlanoğlu F, Ricketts C, Ebren E, Rasekh ME, Hajirasouliha I, Alkan C. VALOR2: characterization of large-scale structural variants using linked-reads. *Genome Biol*. BioMed Central Ltd.; 2020; doi: 10.1186/S13059-020-01975-8/TABLES/2.

26. Edge P, Bafna V, Bansal V. HapCUT2: robust and accurate haplotype assembly for diverse sequencing technologies. *Genome Res*. Genome Res; 2017; doi: 10.1101/GR.213462.116.

27. Patterson MD, Marschall T, Pisanti N, Van Iersel L, Stougie L, Klau GW, et al.. WhatsHap: Weighted Haplotype Assembly for Future-Generation Sequencing Reads. *https://home.liebertpub.com/cmb*. Mary Ann Liebert, Inc. 140 Huguenot Street, 3rd Floor New Rochelle, NY 10801 USA ; 2015; doi: 10.1089/CMB.2014.0157.

28. Zhang L, Kong Baptist H, Zhang Z, Kong H, Xiao J, Wang H, et al.. Exploring high-quality microbial genomes by assembling short-reads with long-range connectivity. 2023; doi: 10.21203/RS.3.RS-3280231/V1.

29. Zhang L, Fang X, Liao H, Zhang Z, Zhou X, Han L, et al.. A comprehensive investigation of metagenome assembly by linked-read sequencing. *Microbiome*. BioMed Central Ltd; 2020; doi: 10.1186/S40168-020-00929-3/FIGURES/4.

30. Zhang L, Zhou X, Weng Z, Sidow A. De novo diploid genome assembly for genome-wide structural variant detection. *NAR Genomics Bioinforma*. Oxford Academic; 2020; doi: 10.1093/NARGAB/LQZ018.

31. Lu J, Breitwieser FP, Thielen P, Salzberg SL. Bracken: estimating species abundance in metagenomics data. *PeerJ Comput Sci*. PeerJ Inc.; 2017; doi: 10.7717/PEERJ-CS.104.

32. Shen W, Xiang H, Huang T, Tang H, Peng M, Cai D, et al.. KMCP: accurate metagenomic profiling of both prokaryotic and viral populations by pseudo-mapping. *Bioinformatics*. Oxford Academic; 2023; doi: 10.1093/BIOINFORMATICS/BTAC845.

33. Segata N, Waldron L, Ballarini A, Narasimhan V, Jousson O, Huttenhower C. Metagenomic microbial community profiling using unique clade-specific marker genes. *Nat Methods*. 2012; doi: 10.1038/nmeth.2066.

34. Zhao C, Dimitrov B, Goldman M, Nayfach S, Pollard KS. MIDAS2: Metagenomic Intra-species Diversity Analysis System. *Bioinformatics*. Oxford Academic; 2023; doi: 10.1093/BIOINFORMATICS/BTAC713.

35. Olm MR, Crits-Christoph A, Bouma-Gregson K, Firek BA, Morowitz MJ, Banfield JF. inStrain profiles population microdiversity from metagenomic data and sensitively detects shared microbial strains. *Nat Biotechnol 2021 396*. Nature Publishing Group; 2021; doi: 10.1038/s41587-020-00797-0.

36. D L, CM L, R L, K S, TW L. MEGAHIT: an ultra-fast single-node solution for large and complex metagenomics assembly via succinct de Bruijn graph. *Bioinformatics*. Bioinformatics; 2015; doi: 10.1093/BIOINFORMATICS/BTV033.

37. Nurk S, Meleshko D, Korobeynikov A, Pevzner PA. metaSPAdes: a new versatile metagenomic assembler. *Genome Res*. Cold Spring Harbor Laboratory Press; 2017; doi: 10.1101/GR.213959.116.

38. A B, EL M, M K, AE P, Z W, A S, et al.. High-quality genome sequences of uncultured microbes by assembly of read clouds. *Nat Biotechnol*. Nat Biotechnol; 2018; doi: 10.1038/NBT.4266.

39. I T, A B, Z C, PA P. cloudSPAdes: assembly of synthetic long reads using de Bruijn graphs. *Bioinformatics*. Bioinformatics; 2019; doi: 10.1093/BIOINFORMATICS/BTZ349.

40. Kang DD, Li F, Kirton E, Thomas A, Egan R, An H, et al.. MetaBAT 2: An adaptive binning algorithm for robust and efficient genome reconstruction from metagenome assemblies. *PeerJ*. PeerJ Inc.; 2019; doi: 10.7717/PEERJ.7359/SUPP-3.

41. Zhou X, Batzoglou S, Sidow A, Zhang L. HAPDeNovo: A haplotype-based approach for filtering and phasing de novo mutations in linked read sequencing data. *BMC Genomics*. BioMed Central Ltd.; 2018; doi: 10.1186/S12864-018-4867-7/TABLES/3.

42. Chaisson MJP, Sanders AD, Zhao X, Malhotra A, Porubsky D, Rausch T, et al.. Multi-platform discovery of haplotype-resolved structural variation in human genomes. *Nat Commun 2019 101*. Nature Publishing Group; 2019; doi: 10.1038/s41467-018-08148-z.

43. Kavak P, Lin YY, Numanagić I, Asghari H, Güngör T, Alkan C, et al.. Discovery and genotyping of novel sequence insertions in many sequenced individuals. *Bioinformatics*. Oxford Academic; 2017; doi: 10.1093/BIOINFORMATICS/BTX254.

44. Krannich T, Timothy W, Niehus S, Holley G, Halldorsson B V., Kehr B. Population-scale detection of non-reference sequence variants using colored de Bruijn graphs. *Bioinformatics*. Oxford Academic; 2022; doi: 10.1093/BIOINFORMATICS/BTAB749.

45. Altshuler DL, Durbin RM, Abecasis GR, Bentley DR, Chakravarti A, Clark AG, et al.. A map of human genome variation from population-scale sequencing. *Nature*. 2010; doi: 10.1038/nature09534.

46. E P, F A, S M, M Z, N K, F A, et al.. Extensive Unexplored Human Microbiome Diversity Revealed by Over 150,000 Genomes from Metagenomes Spanning Age, Geography, and Lifestyle. *Cell*. Cell; 2019; doi: 10.1016/J.CELL.2019.01.001.

47. Xia LC, Bell JM, Wood-Bouwens C, Chen JJ, Zhang NR, Ji HP. Identification of large rearrangements in cancer genomes with barcode linked reads. *Nucleic Acids Res*. Oxford Academic; 2018; doi: 10.1093/NAR/GKX1193.

48. Zhang F, Christiansen L, Thomas J, Pokholok D, Jackson R, Morrell N, et al.. Haplotype phasing of whole human genomes using bead-based barcode partitioning in a single tube. *Nat Biotechnol 2017 359*. Nature Publishing Group; 2017; doi: 10.1038/nbt.3897.

49. Meier JI, Salazar PA, Kučka M, Davies RW, Dréau A, Aldás I, et al.. Haplotype tagging reveals parallel formation of hybrid races in two butterfly species. *Proc Natl*

Acad Sci U S A. National Academy of Sciences; 2021; doi: 10.1073/PNAS.2015005118/SUPPL_FILE/PNAS.2015005118.SD06.XLSX.

50. Redin D, Frick T, Aghelpasand H, Käller M, Borgström E, Olsen RA, et al.. High throughput barcoding method for genome-scale phasing. *Sci Reports 2019 91*. Nature Publishing Group; 2019; doi: 10.1038/s41598-019-54446-x.

51. Zheng W, Zhao S, Yin Y, Zhang H, Needham DM, Evans ED, et al.. High-throughput, single-microbe genomics with strain resolution, applied to a human gut microbiome. *Science (80-)*. American Association for the Advancement of Science; 2022; doi: 10.1126/SCIENCE.ABM1483/SUPPL_FILE/SCIENCE.ABM1483_MOVIES_S1_TO_S10.ZIP.

52. Chen S, Zhou Y, Chen Y, Gu J. fastp: an ultra-fast all-in-one FASTQ preprocessor. *Bioinformatics*. Oxford Academic; 2018; doi: 10.1093/BIOINFORMATICS/BTY560.

53. Bowers RM, Kyrpides NC, Stepanauskas R, Harmon-Smith M, Doud D, Reddy TBK, et al.. Minimum information about a single amplified genome (MISAG) and a metagenome-assembled genome (MIMAG) of bacteria and archaea. *Nat Biotechnol 2017 358*. Nature Publishing Group; 2017; doi: 10.1038/nbt.3893.

54. Schneider VA, Graves-Lindsay T, Howe K, Bouk N, Chen HC, Kitts PA, et al.. Evaluation of GRCh38 and de novo haploid genome assemblies demonstrates the enduring quality of the reference assembly. *Genome Res*. Cold Spring Harbor Laboratory Press; 2017; doi: 10.1101/GR.213611.116.

55. Parks DH, Chuvochina M, Rinke C, Mussig AJ, Chaumeil P-A, Hugenholtz P. GTDB: an ongoing census of bacterial and archaeal diversity through a phylogenetically consistent, rank normalized and complete genome-based taxonomy. *Nucleic Acids Res*. Oxford University Press (OUP); 2021; doi: 10.1093/NAR/GKAB776.

56. Majidian S, Sedlazeck FJ. PhaseME: Automatic rapid assessment of phasing quality and phasing improvement. *Gigascience*. Oxford University Press; 2020; doi: 10.1093/gigascience/giaa078.

57. Zhou Y, Browning SR, Browning BL. A Fast and Simple Method for Detecting Identity-by-Descent Segments in Large-Scale Data. *Am J Hum Genet*. Cell Press; 2020; doi: 10.1016/J.AJHG.2020.02.010.

58. Jamal-Hanjani M, Wilson GA, McGranahan N, Birkbak NJ, Watkins TBK, Veeriah S, et al.. Tracking the Evolution of Non-Small-Cell Lung Cancer. *N Engl J Med*. N Engl J Med; 2017; doi: 10.1056/NEJMOA1616288.

59. Bishara A, Liu Y, Weng Z, Kashef-Haghighi D, Newburger DE, West R, et al.. Read clouds uncover variation in complex regions of the human genome. *Genome Res*. Genome Res; 2015; doi: 10.1101/GR.191189.115.

Figures and Tables

Figure 1. Overview of LRTK. The LRTK workflow includes a metagenomic section (left panel) and human genomic section (right panel). The metagenomic section

implements barcode correction, barcode-aware alignment and metagenome assembly, long DNA fragment reconstruction, taxonomic classification and quantification, as well as SNV detection and phasing. The human genomic section implements barcode correction, barcode-aware alignment, long DNA fragment reconstruction, and detection and phasing of SNVs, INDELs and SVs.

Figure 2. Distribution of quality metrics for different linked-read sequencing platforms. (A) metagenome sequencing. **(B)** human genome sequencing. The left panel displays the distribution of the number of long DNA fragments per barcode. The middle panel shows the length distributions of reconstructed long DNA fragments. The right panel shows the distribution of short-read coverage of fragments.

Figure 3. Comparison of linked-read based metagenomic quantification, SNV identification and phasing. (A) Evaluation of tools to quantify taxonomic abundance based on the linked-reads from simulated dataset (left panel) and ATCC-MSA-1003 (right panel). **(B)** Comparing the performance of SAMtools, FreeBayes and inStrain to detect metagenomic SNVs. **(C)** Dynamic changes of taxonomic abundance and allele frequency. **(D)** SNV based strain phasing in pairwise samples. The right label shows the sample name in D1. BAF is the abbreviation of B allele frequency.

Figure 4. Evaluation of metagenome assemblies on linked-read sequencing. (A) Evaluation of the assembly performance for MEGAHIT, metaSPAdes, Athena, cloudSPAdes and Pangaea on 10x Genomics, stLFR and TELL-Seq linked-read sequencing data from ATCC-MSA-1003. Pangaea does not support 10x Genomics linked-reads. The left panel demonstrates the calculated NA50 values while the right

panel shows the calculated N50 values. **(B)** Illustration of the two assembled circular contigs. **(C)** The distribution of completeness and contamination for reconstructed bins. **(D)** The number of contigs in each contig group. **(E)** The number of detected tRNAs in each contig group.

Figure 5. Evaluation of linked-read based detection of variation in the human genome. (A, B) Performance metrics on the detection of SNVs and INDELs using FreeBayes, GATK and SAMtools for 10x Genomics, stLFR and TELL-Seq. **(C, D)** The performance on phasing of small variants using HapCUT2 and WhatsHap for 10x Genomics, stLFR and TELL-Seq. **(E)** Illustration of the performance on IBD detection. **(F)** The performance on detection of deletions using Aquila, LinkedSV and LongRanger. **(G)** The performance on detection of insertions using Aquila, Pamir and PopIns2.

Figure S1: LRTK text file specification. (A) An example of the unified FASTQ format. Each read contains a barcode field “BX:Z:barcode” after the read names. The lengths of barcodes are 16 bps, 30 bps and 18 bps for 10x Genomics linked-read, stLFR and TELL-Seq, respectively. **(B)** An example of the BAM file generated by LRTK. The barcode information is stored in the “BX:Z:barcode” field.

Figure S2. Ideogrammatic definitions of C_R, C_F, $N_{F/P}$, μ_{FL}, and $W\mu_{FL}$ metrics. (**A**) C_R: Average depth of short reads per fragment. (**B**) C_F: Average physical depth of the genome by long DNA fragments. (**C**) $N_{F/P}$: Number of fragments per barcode. (**D**) Length-weighted average (μ_{FL}) and unweighted average ($W\mu_{FL}$) of DNA fragment

lengths. Here, μFL was calculated as the mean DNA fragment length while WμFL was calculated as the N50 value of all the DNA fragments.

Figure S3. Evaluation of taxonomic quantification performance for 10x Genomics and TELL-Seq linked-reads. (**A**) Evaluation of taxonomic quantification across LRTK, Bracken, KMCP, MetaPhlAn 2 and MIDAS 2 using the simulated 10x Genomics linked-reads. (**B**) Evaluation of taxonomic quantification across LRTK, Bracken, KMCP, MetaPhlAn 2 and MIDAS 2 using the 10x Genomics linked-reads from ATCC-MSA-1003. (**C**) Evaluation of taxonomic quantification across LRTK, Bracken, KMCP, MetaPhlAn 2 and MIDAS 2 using the TELL-Seq linked-reads from ATCC-MSA-1003.

Figure S4: Evaluation of assembly performance at species level. The left panel demonstrates the average assembled genome fraction, NA50 and N50 for ATCC-MSA-1003. The right panel demonstrates the average assembled genome fraction, NA50 and N50 from the simulated dataset.

Figure S5: Evaluation of metagenomic assemblers on simulated linked-read data. The top panel illustrates the NA50 values across five assemblers based on the simulated data. The bottom panel illustrates the N50 values across five assemblers based on the simulated sequencing data.

Figure S6. Demo reports for metagenomics sequencing. (**A**) Per base sequencing quality scores along the reads. (**B**) Per Base GC content along the reads. (**C**) The number of fragments per barcode. (**D**) Fragment length distribution. (**E**). The

abundance of the top 10 most abundant species. (**F**) The number of SNVs per species. (**G**) A principal component analysis using sample data from different groups.

Figure S7: Demo reports for human genome sequencing. (**A**) Per base sequencing quality scores along the reads. (**B**) Per Base GC content along the reads. (**C**) The number of fragments per barcode. (**D**) Fragment length distribution. (**E**) Sequencing depth frequency. (**F**) The distribution of sequencing depth along a whole genome. (**G**) The distribution of inferred insert sizes. (**H**) The distribution of detected deletions. (**I**) The distribution of detected insertions.

Figure S8: Computational requirements for linked-reads preprocessing and alignment between LRTK and Long Ranger on NA12878. (A) linked-reads preprocessing; **(B)** linked-reads alignment.

Table S1. Bioinformatics tools included in LRTK.

Categories	Tools
Linked read simulation	LRTK-SIM [29]
Read preprocessing	fastp [52]
Barcode-aware metagenome assembly	Pangaea [28]
Barcode-aware read alignment	Modified EMA [18] , lariat [59]
SNV detection	FreeBayes [20], GATK [22], inStrain [35], SAMtools [21]
Barcode-assited SV detection	Aquila [23], LinkedSV [24]
Barcode-assited phasing	HapCUT2 [26], WhatsHap [27]

Table S2. Linked-read sequencing data sources.

Cohort	Sample	Sequencing Technologies	Data sources	Size
B1	ATCC-MSA-1003	10x Genomics	SRR12283286	37.7G
		stLFR	PRJNA875547	111G
		TELL-Seq	PRJNA875547	55G
D1	T10	10x Genomics	SRR14763277	14.8G
	T8		SRR14763279	6.9G
	T6		SRR14763281	8.2G
	T5		SRR14763282	40.6G
	T4		SRR14763283	39.5G
	T3		SRR14763284	4.1G
	T18		SRR14763286	10.4G
	T17		SRR14763287	18G
	T16		SRR14763288	20.4G
	T15		SRR14763289	8.4G
	T14		SRR14763290	38.5G

	T13		SRR14763291	10.3G
	T12		SRR14763292	11.6G
	T11		SRR14763293	75G
	T2		SRR14763294	38.1G
	T1		SRR14763295	38.6G
D2	S1	stLFR	CNP0003432 (CNR0585102)	98.97 G
B2	NA12878	10x Genomics	https://ftp-trace.ncbi.nlm.nih.gov/giab/ftp/data/NA12878/10Xgenomics_ChromiumGenome_LongRanger2.0_06202016/NA12878.fastqs/	142.1 G
		stLFR	https://ftp-trace.ncbi.nlm.nih.gov/giab/ftp/data/NA12878/stLFR/	251 G
		TELL-Seq	https://www.ncbi.nlm.nih.gov/sra/SRX7264479	100.4G
D3	NA24385	10x Genomics	https://ftp-trace.ncbi.nlm.nih.gov/giab/ftp/data/AshkenazimTrio/HG002_NA24385_son/10Xgenomics_ChromiumGenome/NA24385.fastqs/	160.3 G
		stLFR	https://ftp-trace.ncbi.nlm.nih.gov/giab/ftp/data/AshkenazimTrio/HG002_NA24385_son/stLFR/	227 G
		TELL-Seq	https://www.ncbi.nlm.nih.gov/sra/SRX7264481	109.5 G

	NA24143	10x Genomics	https://ftp-trace.ncbi.nlm.nih.gov/giab/ftp/data/AshkenazimTrio/HG004_NA24143_mother/10Xgenomics_ChromiumGenome/NA24143.fastqs/	83.3 G
		stLFR	https://ftp-trace.ncbi.nlm.nih.gov/giab/ftp/data/AshkenazimTrio/HG004_NA24143_mother/stLFR/	222 G
		TELL-Seq	NA	NA
	NA24149	10x Genomics	https://ftp-trace.ncbi.nlm.nih.gov/giab/ftp/data/AshkenazimTrio/HG003_NA24149_father/10Xgenomics_ChromiumGenome/NA24149.fastqs/	78.3 G
		stLFR	https://ftp-trace.ncbi.nlm.nih.gov/giab/ftp/data/AshkenazimTrio/HG003_NA24149_father/stLFR/	248 G
		TELL-Seq	NA	NA

Table S3. Data descriptions for the simulated and down-sampled linked-reads from ATCC-MSA-1003 and NA12878.

	**Simulated dataset**	
**Statistics**	**10x Genomics**	**stLFR**

Total Reads (Mb)	134.1	92.17	
Total Bases (Gb)	11.87	9.22	
Q20 Bases (Gb)	11.07	8.57	
Q30 Bases (Gb)	10.41	8.02	
GC content	54.87%	50.63%	
reads passed filters	99.99%	99.99%	
reads with low quality	0	0	
reads with too many N	< 0.01%	< 0.01%	
reads too short	< 0.01%	< 0.01%	
Total number of barcodes	228,824	1,887,428	
Mean number of read pairs per barcode	293.02	24.42	
Median number of read pairs per barcode	284	17	
	**ATCC1003**		
**Statistics**	**10x Genomics**	**stLFR**	**TELL-Seq**
Total Reads (Mb)	151.85	201.2	156.34
Total Bases (Gb)	19.27	20.12	20.25
Q20 Bases (Gb)	19.17	19.12	19.61
Q30 Bases (Gb)	18.9	17.07	18.69
GC content	53.14%	48.75%	46.20%
reads passed filters	99.59%	94.71%	99.99%
reads with low quality	0	0.0348	0
reads with too many N	< 0.01%	< 0.01%	< 0.01%
reads too short	< 0.01%	0.0186	< 0.01%
Total number of barcodes	710,283	8,894,042	3,652,328
Mean number of read pairs per barcode	106.89	12.0864	22.81
Median number of read pairs per barcode	26	6	3
	**NA12878**		
**Statistics**	**10x Genomics**	**stLFR**	**TELL-Seq**
Total Reads (Mb)	794.35	1,097.29	754.79
Total Bases (Gb)	108.85	109.73	110.2
Q20 Bases (Gb)	102.84	104.02	105.27
Q30 Bases (Gb)	96.57	92.3	99.73
GC content	41.50%	41.91%	45.00%
reads passed filters	99.99%	95.73%	94.15%
reads with low quality	< 0.01%	3.09%	0.43%
reads with too many N	< 0.01%	< 0.01%	< 0.01%
reads too short	< 0.01%	1.18%	5.41%
Total number of barcodes	2,331,331	15,826,079	16,909,555

Mean number of read pairs per barcode	170.36	35.61966	23.42
Median number of read pairs per barcode	7	2	1

Table S4. Reference genomes for the ATCC-MSA-1003 mock sample.

**Organism name**	**Relative abundance**	**Reference genome**	**Genome size (bp)**	**ATCC links**
Acinetobacter_baumannii	0.18%	ATCC 17978	4,075,779	https://genomes.atcc.org/genomes/e1d18ea4273549a0
Bacillus_pacificus	1.80%	ATCC 10987	5,442,819	https://genomes.atcc.org/genomes/687931d9b06b4cb4
Bifidobacterium_adolescentis	0.02%	ATCC 15703	2,089,630	https://genomes.atcc.org/genomes/90eb97d11e4b445f
Cereibacter_sphaeroides	18.00%	ATCC 17029	2,089,630	https://genomes.atcc.org/genomes/3a2ecd8337b14710
Clostridium_beijerinckii	1.80%	ATCC 35702	6,007,460	https://genomes.atcc.org/genomes/3210fc7fdeb14ad2
Cutibacterium_acnes	0.18%	ATCC 11828	2,497,484	https://genomes.atcc.org/genomes/070cc66203ff4f5a
Deinococcus_radiodurans	0.02%	ATCC BAA-816	3,280,465	https://genomes.atcc.org/genomes/2c9f475933234e78
Enterococcus_faecalis	0.02%	ATCC 47077	2,738,556	https://genomes.atcc.org/genomes/d8b30e7d0fd74a12
Escherichia_coli	18.00%	ATCC 700926	4,642,497	https://genomes.atcc.org/genomes/8f5bf4f0d4a04c50

Helicobacter_pylori	0.18%	ATCC 700392	1,667,794	https://genomes.atcc.org/genomes/9038b5a9e94245e8
Lactobacillus_gasseri	0.18%	ATCC 33323	1,854,273	https://genomes.atcc.org/genomes/b926c8c7fe4440b5
Neisseria_meningitidis	0.18%	ATCC BAA-335	2,243,896	https://genomes.atcc.org/genomes/261b0e41db924d0f
Phocaeicola_vulgatus	0.02%	ATCC 8482	5,163,177	https://genomes.atcc.org/genomes/b7dcc8d57632456b
Porphyromonas_gingivalis	18.00%	ATCC 33277	2,399,479	https://genomes.atcc.org/genomes/0472781aae86424a
Pseudomonas_paraeruginosa	1.80%	ATCC 9027	6,374,461	https://genomes.atcc.org/genomes/c1a92e4fc09a4ed2
Schaalia_odontolytica	0.02%	ATCC 17982	2,396,044	https://genomes.atcc.org/genomes/6823ab7475dd4769
Staphylococcus_aureus	1.80%	ATCC BAA-1556	2,923,627	https://genomes.atcc.org/genomes/63b4b3239efa42df
Staphylococcus_epidermidis	18.00%	ATCC 12228	2,575,951	https://genomes.atcc.org/genomes/d1ef0271f5b14846
Streptococcus_agalactiae	1.80%	ATCC BAA-611	2,159,783	https://genomes.atcc.org/genomes/e74f122703624e54
Streptococcus_mutans	18.00%	ATCC 700610	2,031,444	https://genomes.atcc.org/genomes/d0622592e00d4ee0

Table S5. Reference genomes for the simulated dataset.

Organism name	Relative abundance	Reference genome	Genome size (bp)
Thermoanaerobacter pseudethanolicus ATCC 33223	0.003055997	NC_010321.1	2,362,816
Desulfitobacterium hafniense DCB-2	0.00607231	NC_011830.1	5,279,134
Desulfofarcimen acetoxidans DSM 771	0.004800511	NC_013216.1	4,545,624
Xylanimonas cellulosilytica DSM 15894	0.039646357	NC_013530.1	3,831,380
Sulfurimonas autotrophica DSM 16294	0.088701172	NC_014506.1	2,153,198
Corynebacterium resistens DSM 45100	0.002236753	NC_015673.1	2,601,311
Bartonella clarridgeiae 73	0.020130366	NC_014932.1	1,522,743
Isoalcanivorax pacificus W11-5	0.015516627	NZ_CP004387.1	4,168,427
Pseudobdellovibrio exovorus JSS	0.003212947	NC_020813.1	2,657,893
Psychromicrobium lacuslunae	0.010557049	NZ_CP011005.1	3,599,434
Helicobacter typhlonius	0.020004482	NZ_LN907858.1	1,920,832
Wolbachia endosymbiont of Folsomia candida	0.05823542	NZ_CP015510.2	1,801,626
Sporosarcina ureae	0.017837164	NZ_CP015108.1	3,362,333
Sedimentisphaera salicampi	0.003382715	NZ_CP021023.1	3,192,293
Fuscovulum blasticum	0.027337676	NZ_CP020470.1	3,706,095
Dongshaea marina	0.030624348	NZ_CP028897.1	5,112,152

Runella rosea	0.006440272	NZ_CP030850.1	7,162,093
Desulfovibrio ferrophilus	0.010084579	NZ_AP017378.1	3,720,107
Bradyrhizobium guangzhouense	0.018027951	NZ_CP030053.1	8,138,177
Haematobacter massiliensis	0.004961852	NZ_CP035510.1	4,232,912
Cellulomonas shaoxiangyii	0.09751048	NZ_CP039291.1	3,909,366
Microlunatus elymi	0.011982578	NZ_CP041692.1	5,121,194
Kushneria phosphatilytica	0.033312055	NZ_CP043420.1	3,624,619
Lactiplantibacillus plantarum	0.007816798	NZ_CP028221.1	3,223,643
Mycolicibacterium tokaiense	0.013904496	NZ_AP022600.1	6,328,149
Mycolicibacillus koreensis	0.008194722	NZ_AP022594.1	4,155,701
Lactococcus garvieae	0.00591555	NZ_CP065637.1	2,084,337
Rhizorhabdus wittichii	0.036707087	NZ_CP059319.1	6,073,182
Parabacteroides goldsteinii	0.006468574	NZ_CP081906.1	7,053,599
Pseudomonas savastanoi	0.007014885	NZ_CP076652.1	5,999,881
Escherichia coli str. K-12 substr. MG1655	0.175270285	NZ_CP097882.1	4,675,188
Salinimicrobium tongyeongense	0.013594968	NZ_CP069620.1	3,509,958
Iamia majanohamensis	0.005263885	NZ_CP116942.1	4,576,919
Ligilactobacillus faecis	0.012418841	NZ_CP123639.1	2,382,213
Borreliella kurtenbachii	0.014520015	NZ_CP124058.1	1,015,378
Staphylococcus schweitzeri	0.004400918	NZ_LR134304.1	2,784,939
Cardiobacterium hominis	0.00908479	NZ_LR134365.1	2,670,755

Slackia heliotrinireducens	0.005737567	NZ_LR134379.1	3,166,158
Wolbachia endosymbiont (group A) of Icerya purchasi	0.007627304	NZ_OX366357.1	1,372,034
Wolbachia endosymbiont (group A) of Hylaeus communis	0.132387656	NZ_OX366396.1	1,526,905

Manuscript

Click here to access/download;Manuscript;ResponseLetter.LRTK.final.docx

Cover Letter

Dear editor and reviewers,

Many thanks indeed for all your hard work on our manuscript. The comments raised by reviewers are very constructive and valuable, providing us lots of insights and inspirations. We have made careful data re-analyzing and literature research, and made extensive revision to our manuscript and supplemented data. All the comments from reviewers have been addressed point by point as shown below, and corresponding corrections have been made in the revised manuscript.

Thanks for your consideration of our paper and kindly let us know if there's any further explanation needed!

Sincerely,

Lu Zhang
Department of Computer Science, Faculty of Science, Hong Kong Baptist University, Hong Kong
E-mail: ericluzhang@hkbu.edu.hk
Phone: +852 3411 5880

GIGA-D-23-00278
LRTK: A platform agnostic toolkit for linked-read analysis of both human genomes and metagenomes
Lu Zhang; Chao Yang; Zhenmiao Zhang; Yufen Huang; Xuefeng Xie; Herui Liao; Jin Xiao; Werner Pieter Veldsman; Kejing Yin; Xiaodong Fang
GigaScience

Dear Dr. Zhang,

Your manuscript "LRTK: A platform agnostic toolkit for linked-read analysis of both human genomes and metagenomes" (GIGA-D-23-00278) has been assessed by three reviewers. Although it is of interest, we are unable to consider it for publication in its current form. The reviewers have raised a number of points which we believe would improve the manuscript and may allow a revised version to be published in GigaScience.

Their reports, together with any other comments, are below.

The reviewers agree that the toolkit you present is useful; however, they also agree that the manuscript needs some major improvement before it can be considered for publication (clarifications and corrections of a number of points, and improved benchmarking and analysis).

If you are able to fully address these points, we would encourage you to submit a revised manuscript to GigaScience. Once you have made the necessary corrections, please submit online at:

https://www.editorialmanager.com/giga/

If you have forgotten your username or password please use the "Send Login Details" link to get your login information. For security reasons, your password will be reset.

Please include a point-by-point within the 'Response to Reviewers' box in the submission system. Please ensure you describe additional experiments that were carried out and include a detailed rebuttal of any criticisms or requested revisions that you disagreed with. Please also ensure that your revised manuscript conforms to the journal style, which can be found in the Instructions for Authors on the journal homepage. If the data and code has been modified in the revision process please be sure to update the public versions of this too.

The due date for submitting the revised version of your article is 13 Feb 2024.

I look forward to receiving your revised manuscript soon.

Best wishes,

Hans Zauner
GigaScience
www.gigasciencejournal.com

Reviewer reports:

Reviewer #1: Yang et al. describe a package of tools, LRTK, for cobarcoded reads (linked reads) agnostic of library preparation methods and sequencing platforms. In general, it appears to be a very useful tool. I have a few concerns with the manuscript as it is currently written:

We thank the reviewer for the comments and address each point below.

1. Line 203 "With Pangaea, LRTK achieves NA50 values of 1.8 Mb and 1.2 Mb for stLFR and TELL-Seq sequencing data, respectively. On 10x Genomics sequencing data, Athena exhibited superior assembly performance, with a NGA50 of 245 Kb."

This is a bit of an awkward two sentences as you are comparing NA50 values for stLFR and TELL-Seq and then NGA50 for 10X Genomics and it makes it sound like 10X Genomics performed the best. Also, these numbers don't seem to agree with the figure.

Responses:

Sorry for the confusion and inconsistency between text and figures in the original manuscript. We found the values in Line 203 should be N50 rather than NA50. In the revised manuscript, we have corrected this typo and replaced NGA50 with NA50 for 10x Genomics assembly.

The two sentences have been revised as **(from line 248 to 252)**:

“Among them, LRTK (Pangaea module) achieves the highest NA50 values for stLFR (NA50=372kb) and TELL-Seq (NA50=339kb) linked-reads, respectively **(Figure 4A)**. As Pangaea is not compatible with 10x Genomics linked-reads, Athena becomes the best tool on 10x Genomics in terms for NA50 (Athena:146 kb; cloudSPAdes: 45 kb; metaSPAdes: 17 kb and MEGAHIT:79 kb).”

Figure 4A:

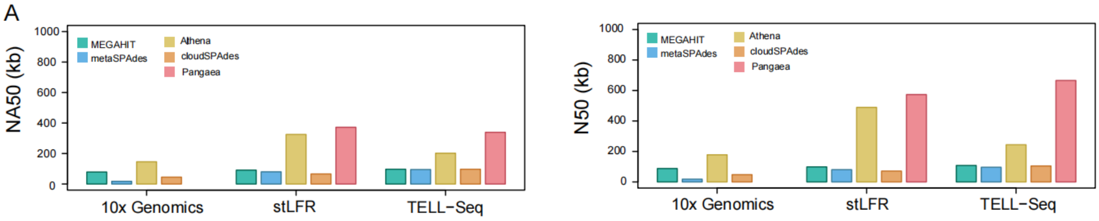

Figure 4. Evaluation of metagenome assemblies on linked-read sequencing. (A). Evaluation of the assembly performance for MEGAHIT, metaSPAdes, Athena, cloudSPAdes and Pangaea on 10x Genomics, stLFR and TELL-Seq linked-read sequencing data from MSA-ATCC-1003. Pangaea does not support 10x Genomics linked-reads.

2. How long does an average run take to process? Say a 35X human genome coverage sample? Are there requirements for memory? A figure and metrics around this sort of thing would be helpful.

Responses:

We compared computational resources, including runtime and memory requirements of LRTK for data preprocessing and performing read alignments on NA12878 using the same data volume (around 35X, 110G base) across three linked-read sequencing platforms. The analysis was conducted on computational nodes equipped with Xeon Gold 6330 CPUs at 2.0GHz (2S/28C), with

1TB of RAM and 900GB of swap space.

We added section in Results (**from lines 323 to 336**):

“Evaluation of the computational resources required for LRTK
We would focus on evaluating computational resources required by LRTK for linked-read preprocessing and alignment. The computational resources needed for genomic variants calling and phasing depend on the chosen software, while metagenome sequencing data typically demand fewer computational resources due to the lower volume of required sequencing reads. We extracted approximately the same data volume (around 35X, 110G bases) of linked-reads from NA12878 for 10x Genomics, stLFR and TELL-Seq and aligned these reads to the human reference genomes. As shown in **Figure S8**, LRTK required around 26.3, 37.6 and 19.8 hours to align reads from the three platforms with a maximum memory usage of around 74Gb using 64 threads. In comparison to Long Range, LRTK reduced memory requirements (maximum memory for Long Ranger: >100 Gb) at the expense of increased running time. The experiments were carried out on the computational nodes with Xeon Gold 6330 @ 2.0GHz (2S/28C) / 1T RAM / 900GB SWAP.”

Figure S8:

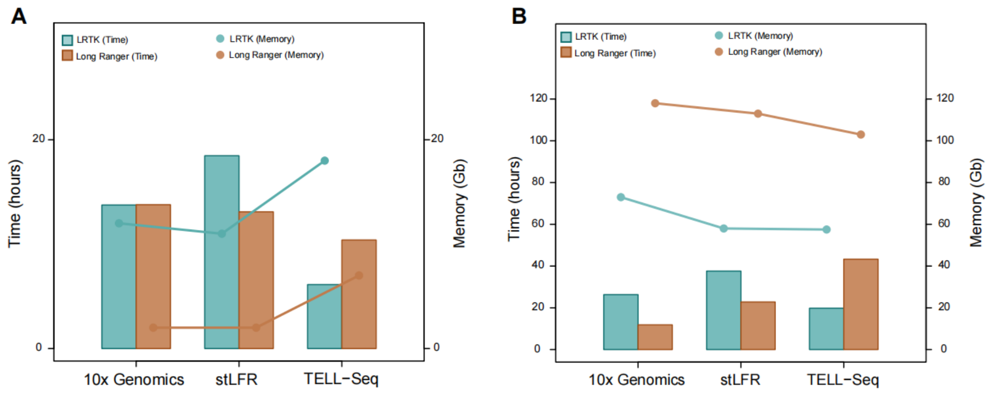

Figure S8: Computational requirements for linked-reads preprocessing and alignment between LRTK and Long Ranger on NA12878. **(A)** linked-reads preprocessing; **(B)** linked-reads alignment.

3. How much data was used per library? What was the total coverage? Was the data normalized to have the same coverage per library? If not, it's very difficult to make fair comparisons between the different technologies.

Response:

Thank you for your comments. To ensure comparability, we subsampled linked-reads from the three technologies to achieve the same data volume. For each technology, we extracted approximately 35X (110Gb) linked-reads for NA12878 and 20Gb for ATCC-MAS-1003. We have re-calculated the key parameters and updated their distributions in **Figure 2** and **Table S3**. The results have been also revised in the manuscript **(from line 197 to line 204).**

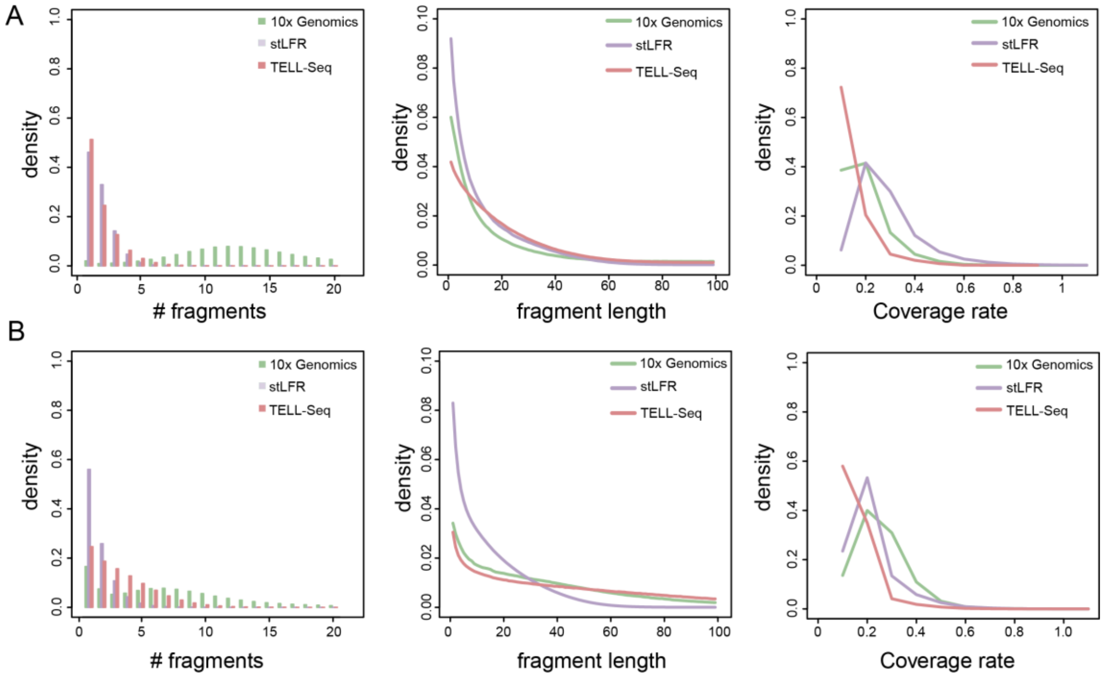

Figure 2. Distribution of quality metrics for different linked-read sequencing platforms. (A) metagenomic sequencing. (B) human genome sequencing. The left panel displays the distribution of the number of long DNA fragments per barcode; The middle panel shows the length distributions of reconstructed long DNA fragments. The right panel shows the distribution of short-read coverage of fragments.

	Simulated dataset		ATCC1003			NA12878		
Statistics	10x Genomics	stLFR	10x Genomics	stLFR	TELL-Seq	10x Genomics	stLFR	TELL-Seq
Total Reads (Mb)	134.1	92.17	151.85	201.2	156.34	794.35	1,097.29	754.79
Total Bases (Gb)	11.87	9.22	19.27	20.12	20.25	108.85	109.73	110.2
Q20 Bases (Gb)	11.07	8.57	19.17	19.12	19.61	102.84	104.02	105.27
Q30 Bases (Gb)	10.41	8.02	18.9	17.07	18.69	96.57	92.3	99.73
GC content	54.87%	50.63%	53.14%	48.75%	46.20%	41.50%	41.91%	45.00%
reads passed filters	99.99%	99.99%	99.59%	94.71%	99.99%	99.99%	95.73%	94.15%
reads with low quality	0	0	0	0.0348	0	< 0.01%	3.09%	0.43%
reads with too many N	< 0.01%	< 0.01%	< 0.01%	< 0.01%	< 0.01%	< 0.01%	< 0.01%	< 0.01%
reads too short	< 0.01%	< 0.01%	< 0.01%	0.0186	< 0.01%	< 0.01%	1.18%	5.41%
Total number of barcodes	228,824	1,887,428	710,283	8,894,042	3,652,328	2,331,331	15,826,079	16,909,555
Mean number of read pairs per barcode	293.02	24.42	106.89	12.0864	22.81	170.36	35.61966	23.42
Median number of read pairs per barcode	284	17	26	6	3	7	2	1

Table S3. Data descriptions for the simulated and downsampled linked-reads from ATCC-MSA-1003 and NA12878.

4. There's a section on reconstruction of long fragments, but then there really isn't any evaluation of this result and it's not clear if these are even used for anything. For all of these sequencing types I would assume that you can't really do much in the way of seed extension since the coverage across long fragments for these methods is much less than 1X. I think this needs to be developed a little more or it needs to be explained how these are used in your process or you just need to say you didn't use them for anything but here's some potential applications they could be used for. What type of file is output from this process? I think it's interesting, but just not clear how to use this data.

Response:

In our previous study, we have shown the statistics of the reconstructed long DNA fragments (E.g. C_F, C_R et al.) can significantly affect human genome assembly [1], structural variants call [2] and metagenome assembly [3]. Also, these statistics are also critical indicators to evaluate the quality of

the library and provide essential insights for its improvement. For example, a low C_F suggests the need for increased DNA input, while the same low C_R indicates a requirement for generating additional reads. Once we obtain results from data analysis, it is crucial to discern whether any issues arise from the library's quality or from the data analysis tools themselves, such as those used for structural variant calls and phasing, which heavily rely on long DNA fragments. We add a paragraph in the discussion to further discuss the long fragment in the revised manuscript **(from line 373 to line 385)**.

LRTK generates two files for these reconstructed long DNA fragments. The first file provides comprehensive details for each long fragment, such as the associated barcode, chromosome/species, start and end positions, the length of fragments, and the number of co-barcoded read pairs involved. The second file provides a summary of statistics, including the number of fragments per barcode, the average fragment length, and the average coverage of these fragments.

The first file:

```
ID      Barcode Reference_Name  Start_Pos       End_Pos Length  Read_Pairs
1       GGAAGCACTAGTCTACCTTCACATTCTTAT-1        chr1    248929029       248938412       9383    4
2       TTCGCCGTTACATTGTTATAAGCGAGAGCT-1        chr1    248926185       248928628       2443    3
3       CACTAGATTCTCGATGCGCGAATGCCAGTT-1        chr1    248924545       248930702       6157    5
4       GGTTAGTGGCAAGGTTGGCCGCTTATTCTT-1        chr1    248923492       248925305       1813    2
5       TTCCACTACAGCCGTGTCGGCCGTGCAACC-1        chr1    248916165       248924946       8781    2
6       GTCGTCGATCCTATCACTAGTCGATATCCT-1        chr1    248909656       248913280       3624    2
7       TTATTGGACCCTGGATTAAGCCATGCTCAG-1        chr1    248908233       248925412       17179   3
8       TGCACTGTCTTAATATTCAGCCGGTCACTA-1        chr1    248896733       248905979       9246    3
[illegible]
```

The second file:

```
Read coverage (C): 52.5236
Mean fragment length (U_FL): 16111.5
Fragment length max, N25, N50 (WU_FL), N75, min: 1232271, 38308, 25950, 15507, 1000
Fragment coverage (C_F): 319.723
Read coverage per fragment (C_R): 0.164279
Fragments per barcode (N_F/P): 1.77108
```

5. I did try to install the software using Conda, but it failed and it's not clear to me why. Perhaps it's something about my environment, but you might want to have some colleagues located in different institutions try to install it to make sure it is easy to do so.

Response:

The installation process of LRTK can be a little bit time-consuming due to the numerous packages and third-party tools involved. In our experience, it typically takes around 30 minutes to complete the entire installation procedure. We have successfully installed LRTK on our server in Hong Kong and also on a HPC system of UCSD, with both installations taking approximately the same amount of time (as shown in the screenshot below). There may be environmental factors influencing the installation time that we are still investigating. Users facing challenges during installation are encouraged to report issues on our GitHub repository or reach out to us via email for direct assistance.

The HK cluster:

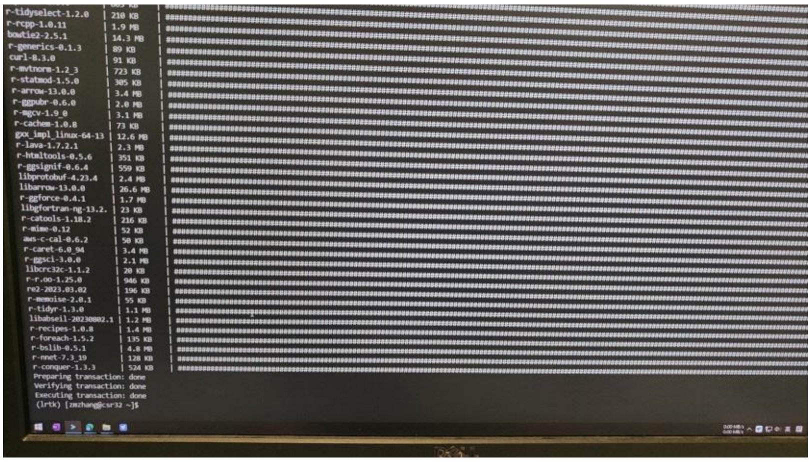

```
r-tidyselect-1.2.0     | 210 KB  |
r-rcpp-1.0.11          | 1.9 MB  |
bowtie2-2.5.1          | 14.3 MB |
r-generics-0.1.3       | 89 KB   |
curl-8.3.0             | 91 KB   |
r-mvtnorm-1.2_3        | 723 KB  |
r-statmod-1.5.0        | 305 KB  |
r-arrow-13.0.0         | 3.4 MB  |
r-ggpubr-0.6.0         | 2.0 MB  |
r-mgcv-1.9_0           | 3.1 MB  |
r-cachem-1.0.8         | 73 KB   |
gxx_impl_linux-64-13   | 12.6 MB |
r-lava-1.7.2.1         | 2.3 MB  |
r-htmltools-0.5.6      | 351 KB  |
r-ggsignif-0.6.4       | 559 KB  |
libprotobuf-4.23.4     | 2.4 MB  |
libarrow-13.0.0        | 26.6 MB |
r-ggforce-0.4.1        | 1.7 MB  |
libgfortran-ng-13.2.   | 23 KB   |
r-catools-1.18.2       | 216 KB  |
r-mime-0.12            | 52 KB   |
aws-c-cal-0.6.2        | 50 KB   |
r-caret-6.0_94         | 3.4 MB  |
r-ggsci-3.0.0          | 2.1 MB  |
libcrc32c-1.1.2        | 20 KB   |
r-r.oo-1.25.0          | 946 KB  |
re2-2023.03.02         | 196 KB  |
r-memoise-2.0.1        | 55 KB   |
r-tidyr-1.3.0          | 1.1 MB  |
libabseil-20230802.1   | 1.2 MB  |
r-recipes-1.0.8        | 1.4 MB  |
r-foreach-1.5.2        | 135 KB  |
r-bslib-0.5.1          | 4.8 MB  |
r-nnet-7.3_19          | 128 KB  |
r-conquer-1.3.3        | 524 KB  |
Preparing transaction: done
Verifying transaction: done
Executing transaction: done
(lrtk) [zmzhang@csr32 ~]$
```

The UCSD cluster:

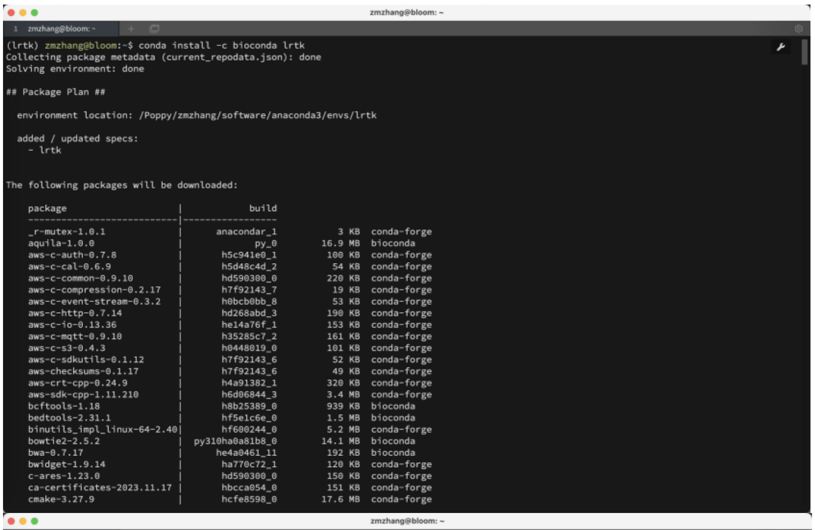

```
zmzhang@bloom: ~
(lrtk) zmzhang@bloom:~$ conda install -c bioconda lrtk
Collecting package metadata (current_repodata.json): done
Solving environment: done

## Package Plan ##

  environment location: /Poppy/zmzhang/software/anaconda3/envs/lrtk

  added / updated specs:
    - lrtk


The following packages will be downloaded:

    package                    |            build
    ---------------------------|-----------------
    _r-mutex-1.0.1             |      anacondar_1           3 KB  conda-forge
    aquila-1.0.0               |             py_0        16.9 MB  bioconda
    aws-c-auth-0.7.8           |       h5c941e0_1         100 KB  conda-forge
    aws-c-cal-0.6.9            |       h5d48c4d_2          54 KB  conda-forge
    aws-c-common-0.9.10        |       hd590300_0         220 KB  conda-forge
    aws-c-compression-0.2.17   |       h7f92143_7          19 KB  conda-forge
    aws-c-event-stream-0.3.2   |       h0bcb0bb_8          53 KB  conda-forge
    aws-c-http-0.7.14          |       hd268abd_3         190 KB  conda-forge
    aws-c-io-0.13.36           |       he14a76f_1         153 KB  conda-forge
    aws-c-mqtt-0.9.10          |       h35285c7_2         161 KB  conda-forge
    aws-c-s3-0.4.3             |       h0448019_0         101 KB  conda-forge
    aws-c-sdkutils-0.1.12      |       h7f92143_6          52 KB  conda-forge
    aws-checksums-0.1.17       |       h7f92143_6          49 KB  conda-forge
    aws-crt-cpp-0.24.9         |       h4a91382_1         320 KB  conda-forge
    aws-sdk-cpp-1.11.210       |       h6d06844_3         3.4 MB  conda-forge
    bcftools-1.18              |       h8b25389_0         939 KB  bioconda
    bedtools-2.31.1            |       hf5e1c6e_0         1.5 MB  bioconda
    binutils_impl_linux-64-2.40|       hf600244_0         5.2 MB  conda-forge
    bowtie2-2.5.2              |  py310ha0a81b8_0        14.1 MB  bioconda
    bwa-0.7.17                 |      he4a0461_11         192 KB  bioconda
    bwidget-1.9.14             |       ha770c72_1         120 KB  conda-forge
    c-ares-1.23.0              |       hd590300_0         150 KB  conda-forge
    ca-certificates-2023.11.17 |       hbcca054_0         151 KB  conda-forge
    cmake-3.27.9               |       hcfe8598_0        17.6 MB  conda-forge
```

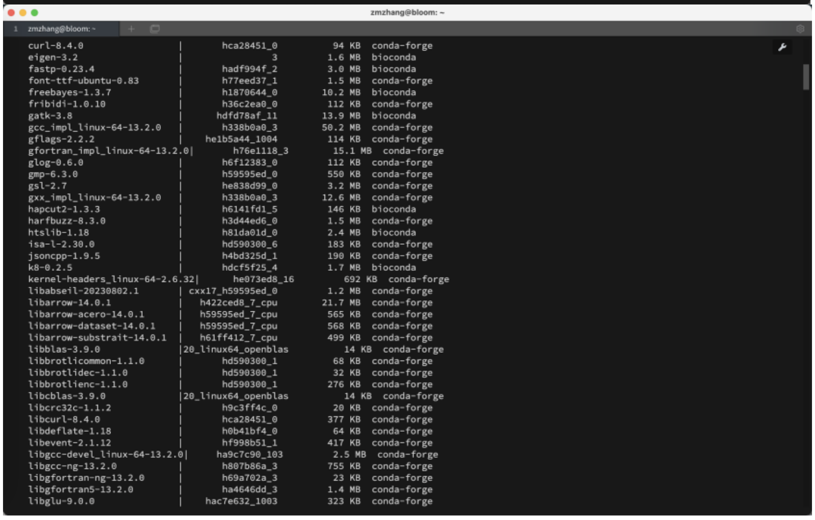

```
zmzhang@bloom: ~
    curl-8.4.0                 |       hca28451_0          94 KB  conda-forge
    eigen-3.2                  |                3         1.6 MB  bioconda
    fastp-0.23.4               |       hadf994f_2         3.0 MB  bioconda
    font-ttf-ubuntu-0.83       |       h77eed37_1         1.5 MB  conda-forge
    freebayes-1.3.7            |       h1870644_0        10.2 MB  bioconda
    fribidi-1.0.10             |       h36c2ea0_0         112 KB  conda-forge
    gatk-3.8                   |      hdfd78af_11        13.9 MB  bioconda
    gcc_impl_linux-64-13.2.0   |       h338b0a0_3        50.2 MB  conda-forge
    gflags-2.2.2               |    he1b5a44_1004         114 KB  conda-forge
    gfortran_impl_linux-64-13.2.0|       h76e1118_3        15.1 MB  conda-forge
    glog-0.6.0                 |       h6f12383_0         112 KB  conda-forge
    gmp-6.3.0                  |       h59595ed_0         550 KB  conda-forge
    gsl-2.7                    |       he838d99_0         3.2 MB  conda-forge
    gxx_impl_linux-64-13.2.0   |       h338b0a0_3        12.6 MB  conda-forge
    hapcut2-1.3.3              |       h6141fd1_5         146 KB  bioconda
    harfbuzz-8.3.0             |       h3d44ed6_0         1.5 MB  conda-forge
    htslib-1.18                |       h81da01d_0         2.4 MB  bioconda
    isa-l-2.30.0               |       hd590300_6         183 KB  conda-forge
    jsoncpp-1.9.5              |       h4bd325d_1         190 KB  conda-forge
    k8-0.2.5                   |       hdcf5f25_4         1.7 MB  bioconda
    kernel-headers_linux-64-2.6.32|      he073ed8_16         692 KB  conda-forge
    libabseil-20230802.1       | cxx17_h59595ed_0         1.2 MB  conda-forge
    libarrow-14.0.1            |   h422ced8_7_cpu        21.7 MB  conda-forge
    libarrow-acero-14.0.1      |   h59595ed_7_cpu         565 KB  conda-forge
    libarrow-dataset-14.0.1    |   h59595ed_7_cpu         568 KB  conda-forge
    libarrow-substrait-14.0.1  |   h61ff412_7_cpu         499 KB  conda-forge
    libblas-3.9.0              |20_linux64_openblas          14 KB  conda-forge
    libbrotlicommon-1.1.0      |       hd590300_1          68 KB  conda-forge
    libbrotlidec-1.1.0         |       hd590300_1          32 KB  conda-forge
    libbrotlienc-1.1.0         |       hd590300_1         276 KB  conda-forge
    libcblas-3.9.0             |20_linux64_openblas          14 KB  conda-forge
    libcrc32c-1.1.2            |       h9c3ff4c_0          20 KB  conda-forge
    libcurl-8.4.0              |       hca28451_0         377 KB  conda-forge
    libdeflate-1.18            |       h0b41bf4_0          64 KB  conda-forge
    libevent-2.1.12            |       hf998b51_1         417 KB  conda-forge
    libgcc-devel_linux-64-13.2.0|      ha9c7c90_103         2.5 MB  conda-forge
    libgcc-ng-13.2.0           |       h807b86a_3         755 KB  conda-forge
    libgfortran-ng-13.2.0      |       h69a702a_3          23 KB  conda-forge
    libgfortran5-13.2.0        |       ha4646dd_3         1.4 MB  conda-forge
    libglu-9.0.0               |    hac7e632_1003         323 KB  conda-forge
```

```
  tzdata             conda-forge/noarch::tzdata-2023c-h71feb2d_0
  vcflib             bioconda/linux-64::vcflib-1.0.9-h146fbdb_3
  vcftools           bioconda/linux-64::vcftools-0.1.16-h9a82719_5
  wfa2-lib           bioconda/linux-64::wfa2-lib-2.3.4-h4ac6f70_0
  wheel              conda-forge/noarch::wheel-0.42.0-pyhd8ed1ab_0
  xorg-kbproto       conda-forge/linux-64::xorg-kbproto-1.0.7-h7f98852_1002
  xorg-libice        conda-forge/linux-64::xorg-libice-1.1.1-hd590300_0
  xorg-libsm         conda-forge/linux-64::xorg-libsm-1.2.4-h7391055_0
  xorg-libx11        conda-forge/linux-64::xorg-libx11-1.8.7-h8ee46fc_0
  xorg-libxau        conda-forge/linux-64::xorg-libxau-1.0.11-hd590300_0
  xorg-libxdmcp      conda-forge/linux-64::xorg-libxdmcp-1.1.3-h7f98852_0
  xorg-libxext       conda-forge/linux-64::xorg-libxext-1.3.4-h0b41bf4_2
  xorg-libxrender    conda-forge/linux-64::xorg-libxrender-0.9.11-hd590300_0
  xorg-libxt         conda-forge/linux-64::xorg-libxt-1.3.0-hd590300_1
  xorg-renderproto   conda-forge/linux-64::xorg-renderproto-0.11.1-h7f98852_1002
  xorg-xextproto     conda-forge/linux-64::xorg-xextproto-7.3.0-h0b41bf4_1003
  xorg-xproto        conda-forge/linux-64::xorg-xproto-7.0.31-h7f98852_1007
  xz                 conda-forge/linux-64::xz-5.2.6-h166bdaf_0
  zlib               conda-forge/linux-64::zlib-1.2.13-hd590300_5
  zstd               conda-forge/linux-64::zstd-1.5.5-hfc55251_0


Proceed ([y]/n)? y


Downloading and Extracting Packages
s2n-1.3.56           | 380 KB    | ################################################################################################## | 100%

Preparing transaction: done
Verifying transaction: done
Executing transaction: /
To install TinyTeX with `tinytex::install_tinytex()` the system must have a functional Perl
installation with a `File::Find` module. Most end-user systems will already satisfy this
requirement; however, some minimal contexts (e.g., containers) may not. Perl is available
via Conda Forge as the package `perl`. See https://github.com/rstudio/tinytex/issues/419


done
(lrtk) zmzhang@bloom:~$
(lrtk) zmzhang@bloom:~$
```

Reviewer #2: Summary: This manuscript describes the need for a generalized linked-read (LR) analysis package and showcases the package the authors developed to address this need. Overall, the workflow is well-designed but there are major gaps in the benchmarking, analysis, and documentation process that need to be addressed before publication.

We thank the reviewer for these positive comments and address each point below.

Documentation:

The purpose of multiple tool options: While the analysis package is technically sound, one major aspect is left unexplained- why are there so many algorithm options included without guidance as to which one to use? There are clearly performance differences by different algorithms (combinations of 2+ not considered either) on different types of LR sequence.

Response:

Thank you for the suggestions. Our investigations have revealed that different variant calling tools possess unique characteristics and could potentially complement each other in practical scenarios. Therefore, we believe that providing multiple options will greatly benefit users in their projects with different purpose. In our study, we benchmarked different computational tools for the three linked-read sequencing technologies to establish a best practice guideline. For metagenome assembly, we compared three linked-reads assemblers: Athena, cloudSPAdes and Pangaea, and observed Pangaea achieved the highest NA50 values for stLFR (NA50=372kb) and TELL-Seq (NA50=339kb) linked-reads, respectively. For variant phasing on human genome, we compared linked-read phasing tools: HapCUT2 and WhatsHap, and found that HapCUT2 had a larger block size (23.2 Mb) and higher phased rate (99%). For structural variant calling, we observed that Aquila had the highest recall to detect deletions (50 bps – 1 kb) and insertion. We then constructed a best practice framework based on our benchmarking results and set the top-performing tool as the default options in LRTK. In the future, we will investigate the combination of different tools to improve overall performance.

In the revised manuscripts, we have included detailed descriptions in the Method section (Identify and phase genomic variants subsection, from **line 514 to 527** and **line 532 to line 536**):

"For human genomic sequencing data, LRTK provides FreeBayes, SAMtools and GATK to call SNVs and INDELs. FreeBayes is a Bayesian genetic variant detection tool designed to identify SNPs, INDELs, multinucleotide polymorphisms, and more complex events. Its straightforward, easy to use and timesaving. GATK, while also employing a Bayesian framework, enhances its detection capabilities for insertions and deletions through specialized techniques such as read realignment and base recalibration. Although these steps add value, they also increase GATK's computational runtime. In contrast, SAMtools uses a hidden Markov model for the identification of small variants and has demonstrated robust performance across various studies. The available phasing tools include HapCUT2 and WhatsHap. HapCUT2 demonstrated excellent performance to phase heterozygous SNVs within a diploid context, such as the human genome. WhatsHap, however, introduces an innovative clustering and threading approach that delivers precise phasing in polyploid genomes."

and

"LinkedSV leverages barcode overlapping, read depth, paired-end signals and local assembly to detect deletions, although it currently lacks support for insertion detection. In contrast, the

assembly-based tool Aquila is capable of detecting both insertions and deletions, offering a comprehensive solution."

Provenance of ATCC-MSA-1003: Nowhere in the manuscript is the biological and technical composition of the metagenomics control described. It would be helpful to mention that this is specifically a mock gut microbiome sample, as well as the relative abundances of the originating species as well as the absolute amounts of genetic material per species (ex. as measured by genomic coverage) in the actual dataset. As a corollary, there should be standard deviations in any figures that display a summary statistic (ex. Figure 3A- precision, recall, etc.) that seems to be averaged across the species in a sample. This includes Figure 3A and Figure 4A.

Response:

Thank you for the comments. We have provided comprehensive descriptions of ATCC-MSA-1003 in the revised manuscript **(lines 408 to 416)**:

"The dataset B1 contains linked-reads from a mock microbial community ATCC-MSA-1003, from three different platforms (SRR12283286 for 10x Genomics, and PRJNA875547 for stLFR and TELL-Seq). The ATCC-MSA-1003 mock community is composed of 20 bacterial species represented at staggered abundances—specifically, five species at 18%, 1.8%, 0.18%, and 0.02% abundance levels, respectively. The complete descriptions of the mock metagenomic sample, including the genome sizes, individual bacterial species, and their corresponding reference sequence accessions, have been included in **Table S4.**"

We have revised two subfigures in **Figure 4A** and added a new **Figure S4A** to incorporate standard deviations. We benchmarked the assembly performance using tools such as LRTK (Pangaea), MEGAHIT, MetaSPAdes, cloudSPAdes, and Athena. Subsequently, we computed the genome fraction, NA50, and N50 metrics for the overall community (as shown in **Figure 4A**) and each species (as shown in **Figure S4A**). For Figure 3A, we evaluated the quantification performance using LRTK, MIDAS2, KMCP, MetaPhlAn2, and Bracken2, where precision, recall, F1 score, and correlation score were determined based on the all species within a sample, rather than an average across all species. Therefore, these scores were not computed for an individual species and we could not provide standard deviations for them. The related descriptions were updated in the revised manuscript from **line 252 to line 255**：

"We also examined the assembly quality of each species in ATCC-MSA-1003, and observed that Pangaea always obtained the highest NA50 and N50 values for stLFR and TELL-Seq linked-reads while the assembly length is comparable **(Figure S4A)**."

Figure S4:

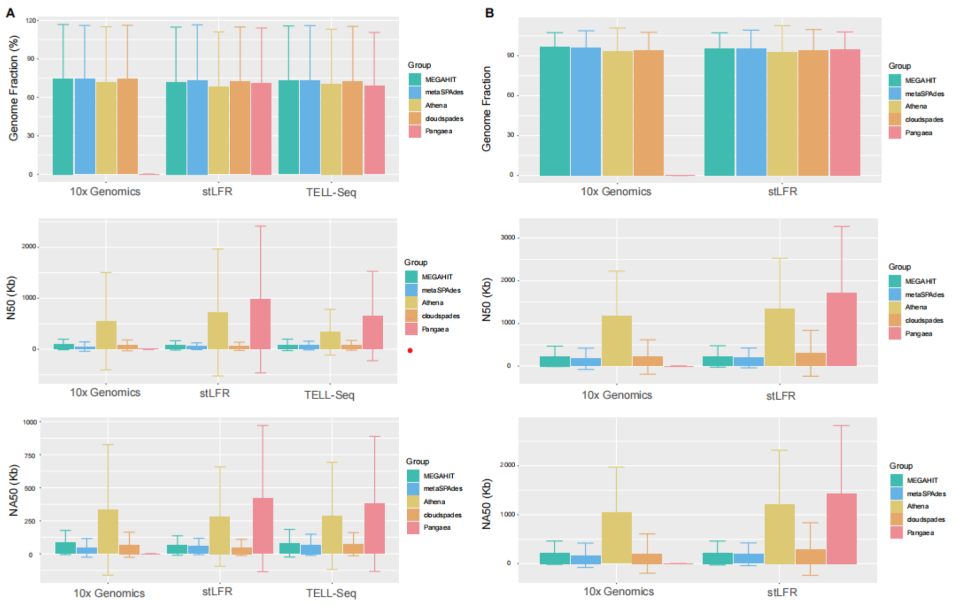

Figure S4: Evaluation of assembly performance at species level. The left panel demonstrates the average assembled genome fraction, NA50 and N50 for ATCC-MSA-1003. The right panel demonstrates the average assembled genome fraction, NA50 and N50 from the simulated dataset.

Figure 4A:

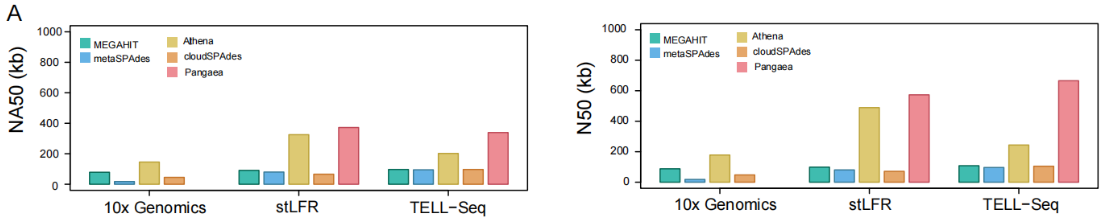

Figure 4. Evaluation of metagenome assemblies on linked-read sequencing. (A). Evaluation of the assembly performance for MEGAHIT, metaSPAdes, Athena, cloudSPAdes and Pangaea on 10x Genomics, stLFR and TELL-Seq linked-read sequencing data from MSA-ATCC-1003. Pangaea does not support 10x Genomics linked-reads.

Dataset details: There is no table indicating the number of reads for each dataset, which would be helpful in interpreting Figures 3 and 4.

Response:

In the revised manuscript, we have included one simulated metagenomic dataset, one dataset from a mock microbial community, two real datasets from human gut microbiomes for metagenomic analysis and four human linked-read sequencing datasets from NA12878, NA24143, NA24149 and NA24385. We added **Table S3** to describe the quality control (QC) about the simulated dataset. For the mock dataset, two human gut microbiome datasets and human genome related datasets, we added the data size in **Table S2**. In addition, we performed down-sampling for linked-read sequencing data from mock dataset and NA12878 dataset and described the QC results in **Table S3**. We added a paragraph to describe these datasets in the revised manuscript **(from line 148 to 167):**

"**Data Description**

We incorporated one simulated dataset (S1), one dataset from a mock community (B1), and two real datasets from human gut microbiomes (D1 and D2) to evaluate metagenomic sequencing analysis module of LRTK **(Table S2)**. For dataset S1**,** we simulated 11.9 Gb and 9.2 Gb linked-reads from 10x Genomics and stLFR for 40 complete bacterial genomes from NCBI RefSeq database, with lognormal abundance distribution (**Methods**), respectively **(Table S3)**. The dataset B1 was generated from ATCC-MSA-1003, containing 20 bacterial species with abundances varying from 0.02% to 18% **(Table S4).** The sequencing data volumes are 37.7 Gb, 111 Gb and 55 Gb for 10x Genomics, stLFR and TELL-Seq linked-reads, respectively. For the two real metagenomic datasets, D1 consists of 16 longitudinal human gut metagenomic sequencing datasets from a single individual, sequenced with an average of 24 Gb linked-reads on 10x Genomics platform. The other dataset D2 contains around 99 Gb stLFR linked-reads from human gut metagenome. For the human genome analysis section, we collected the linked-read sequencing datasets from NA12878, NA24143, NA24149 and NA24385 (**Table S2).** In addition, we performed linked-reads down-sampling to ensure fair comparisons among the three sequencing technologies. We down-sampled around 20 Gb metagenomic linked reads from ATCC-MSA-1003 for each platform. Similarly, around 110 Gb (~35X) linked reads from NA12878 were extracted for the three technologies **(Table S3)**."

Table S3:

	Simulated dataset		ATCC1003			NA12878		
Statistics	10x Genomics	stLFR	10x Genomics	stLFR	TELL-Seq	10x Genomics	stLFR	TELL-Seq
Total Reads (Mb)	134.1	92.17	151.85	201.2	156.34	794.35	1,097.29	754.79
Total Bases (Gb)	11.87	9.22	19.27	20.12	20.25	108.85	109.73	110.2
Q20 Bases (Gb)	11.07	8.57	19.17	19.12	19.61	102.84	104.02	105.27
Q30 Bases (Gb)	10.41	8.02	18.9	17.07	18.69	96.57	92.3	99.73
GC content	54.87%	50.63%	53.14%	48.75%	46.20%	41.50%	41.91%	45.00%
reads passed filters	99.99%	99.99%	99.59%	94.71%	99.99%	99.99%	95.73%	94.15%
reads with low quality	0	0	0	0.0348	0	< 0.01%	3.09%	0.43%
reads with too many N	< 0.01%	< 0.01%	< 0.01%	< 0.01%	< 0.01%	< 0.01%	< 0.01%	< 0.01%
reads too short	< 0.01%	< 0.01%	< 0.01%	0.0186	< 0.01%	< 0.01%	1.18%	5.41%
Total number of barcodes	228,824	1,887,428	710,283	8,894,042	3,652,328	2,331,331	15,826,079	16,909,555
Mean number of read pairs per barcode	293.02	24.42	106.89	12.0864	22.81	170.36	35.61966	23.42
Median number of read pairs per barcode	284	17	26	6	3	7	2	1

Table S3: Data descriptions for the simulated and down-sampled linked-reads from ATCC-MSA-1003 and NA12878.

Table S2:

Table S3. Linked-read sequencing data used in the paper

Cohort	Sample	Sequencing Technologies	Data sources	Size
B1	ATCC-MSA-1003	10x Genomics	SRR12283286	37.7G
		stLFR	PRJNA875547	111G
		TELL-Seq	PRJNA875547	55G
D1	T10	10x Genomics	SRR14763277	14.8G
	T8		SRR14763279	6.9G
	T6		SRR14763281	8.2G
	T5		SRR14763282	40.6G
	T4		SRR14763283	39.5G
	T3		SRR14763284	4.1G
	T18		SRR14763286	10.4G
	T17		SRR14763287	18G
	T16		SRR14763288	20.4G
	T15		SRR14763289	8.4G
	T14		SRR14763290	38.5G
	T13		SRR14763291	10.3G
	T12		SRR14763292	11.6G
	T11		SRR14763293	75G
	T2		SRR14763294	38.1G
	T1		SRR14763295	38.6G
D2	S1	stLFR	CNP0003432	98.97 G
B2	NA12878	10x Genomics	https://ftp-trace.ncbi.nlm.nih.gov/giab/ftp/data/NA12878/10Xgenomics_ChromiumGenome_LongRanger2.0_06202016/NA12878.fastqs/	142.1 G
		stLFR	https://ftp-trace.ncbi.nlm.nih.gov/giab/ftp/data/NA12878/stLFR/	251 G
		TELL-Seq	https://www.ncbi.nlm.nih.gov/sra/SRX7264479	100.4G
D3	NA24385	10x Genomics	https://ftp-	160.3 G
		stLFR	https://ftp-trace.ncbi.nlm.nih.gov/giab/ftp/data/AshkenazimTrio/HG002_NA24385_son/stLFR/	227 G
		TELL-Seq	https://www.ncbi.nlm.nih.gov/sra/SRX7264481	109.5 G
	NA24143	10x Genomics	https://ftp-	83.3 G
		stLFR	https://ftp-trace.ncbi.nlm.nih.gov/giab/ftp/data/AshkenazimTrio/HG004_NA24143_mother/stLFR/	222 G
		TELL-Seq		
	NA24149	10x Genomics	https://ftp-	78.3 G
		stLFR	https://ftp-trace.ncbi.nlm.nih.gov/giab/ftp/data/AshkenazimTrio/HG003_NA24149_father/stLFR/	248 G
		TELL-Seq		

Table S2. Linked-read sequencing data sources.

Open source?: However, there was no Github link provided, only a link to the Conda landing page. Are there thorough instructions provided for the package's installation, input, output, and environment management?

Response:

Thank you for the comments. We have included the GitHub link of LRTK (https://github.com/ericcombiolab/LRTK) in the "Code Availability and Requirements" section. On the GitHub page, users can find comprehensive instructions for tool installation and utilization of LRTK. We also provided a directory named "example" to include an example of necessary inputs. There is also a 'demo_report' directory showing the LRTK final outputs from the example dataset.

Benchmarking:

The lack of simulated tests: The above concern (expected performance on idealized datasets) is best addressed with simulated data, which was not done despite the fact that LR-Sim exists (and apparently the authors have written a tool for stLFR as well previously).

Response:

Thank you for the comments. We agree with the comments that evaluations on simulated data are necessary to demonstrate the pros and cons of the included tools. In the revised manuscript, we simulated around 10Gb of linked-reads for 10x Genomics and stLFR sequencing technologies using 40 bacterial reference genomes. Detailed descriptions about the simulated datasets are included in the **Table S3**.

Using the simulated datasets, we first compared the assembly performance of five assemblers: metaSPAdes, MEGAHIT, Athena, cloudSPAdes, and Pangaea. We added **Figure S4B** and **Figure S5** to compare the assembled NA50 and N50 values. The related results were also updated in the revised manuscript in **lines 255-257:**

"For simulated linked-reads, Pangaea and Athena also show superior performances than the other metagenome assemblers (Figure S4B, Figure S5)."

In addition, we compared the performance of taxonomic classification and abundance quantification among LRTK, MIDAS2, KMCP, Bracken2 and MetaPhlAn 2. We added **Figure S3** and updated the results in **Figure 3A** and related descriptions in the revised manuscript **(lines 211-215; lines 217-222; lines 224-225)**:

"LRTK can better identify the involved microbes based on their genome coverage (F1=0.81) than the existing k-mer based tools: Bracken (F1=0.13), and KMCP (F1=0.67); and marker gene based tools: MetaPhlAn 2 (F1=0.49), and MIDAS 2 (F1=0.72), on the simulated stLFR linked-reads (Figure 3A)."

And

"We also evaluated the LRTK performance of taxonomic quantification by comparing the benchmark microbial abundance (from simulation and ATCC-MSA-1003) and the predicted values using Spearman correlation coefficient (SCC). For the simulated dataset, LRTK

(SCC=0.99), MIDAS 2 (SCC=0.98), and KMCP (SCC=0.95) exhibited superior performance."
And

"Comparable findings were also observed in the linked-reads data generated from other technologies in simulation (Figure S3A) and ATCC-MSA-1003 (Figure S3B and S3C)."

Figure S3:

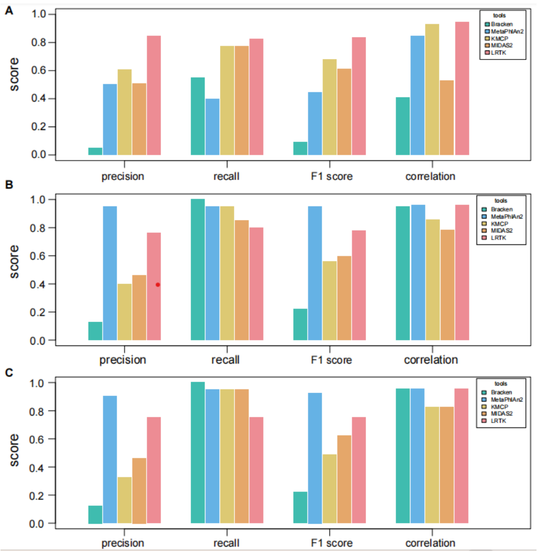

Figure S3. Evaluation of taxonomic quantification performance for 10x Genomics and TELL-Seq linked-reads. (A) Evaluation of taxonomic quantification across LRTK, Bracken, KMCP, MetaPhlAn 2 and MIDAS 2 using the simulated 10x Genomics linked-reads. (B) Evaluation of taxonomic quantification across LRTK, Bracken, KMCP, MetaPhlAn 2 and MIDAS 2 using the 10x Genomics linked-reads from ATCC-MSA-1003. (C) Evaluation of taxonomic quantification across LRTK, Bracken, KMCP, MetaPhlAn 2 and MIDAS 2 using the TELL-Seq linked-reads from ATCC-MSA-1003.

Figure S5:

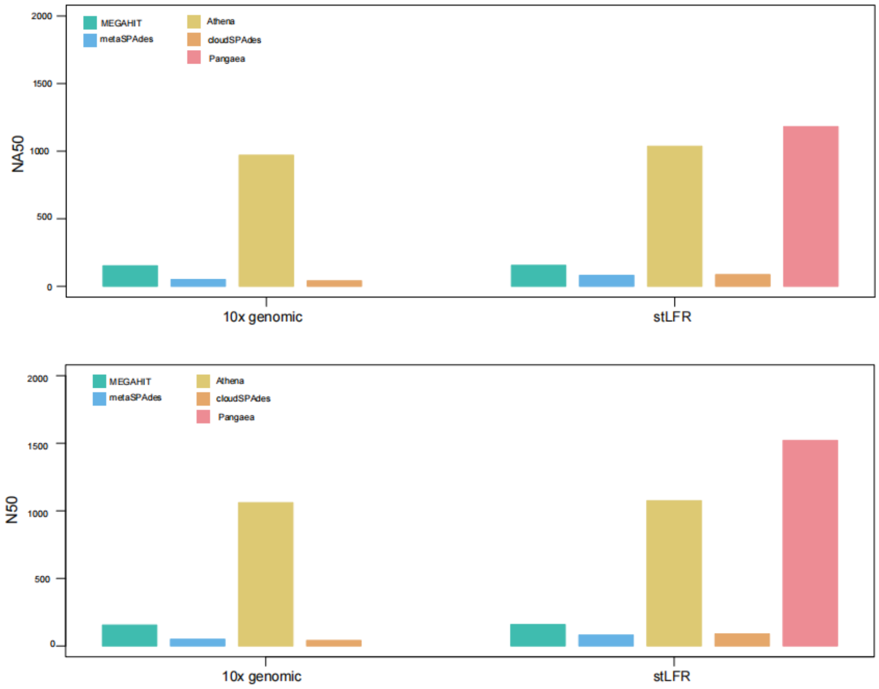

Figure S5: Evaluation of metagenomic assemblers on simulated linked-read data. The top panel

illustrates the NA50 values across five assemblers based on the simulated data. The bottom panel illustrates the N50 values across five assemblers based on the simulated sequencing data.

Indels: What are the sizes of the indels detected? Why were newer tools, such as PopIns2, Pamir, or Novel-X not tried as well?

Response:

Thanks for the comments. In our study, we used FreeBayes, GATK and SAMtools to identify indels shorter than 50 bps. For detecting large structural variants (SVs) exceeding 50 bps, we employed Aquila and LinkedSV. Due to challenges with running Novel-X (Segmental Fault), we opted to include Pamir and PopIns2 to compare their performance with Aquila, Pamir, and PopIns2.

We have included the updated results in the manuscript, specifically in the section spanning from **line 290 to 292**, along with **Figure 5G**.

"Aquila also shows a better performance than Pamir and PopIns2 to detect insertions (Aquila: 0.35; Pamir: 0.07; PopIns2: 0.01) (Figure 5G)."

Figure 5 F and G:

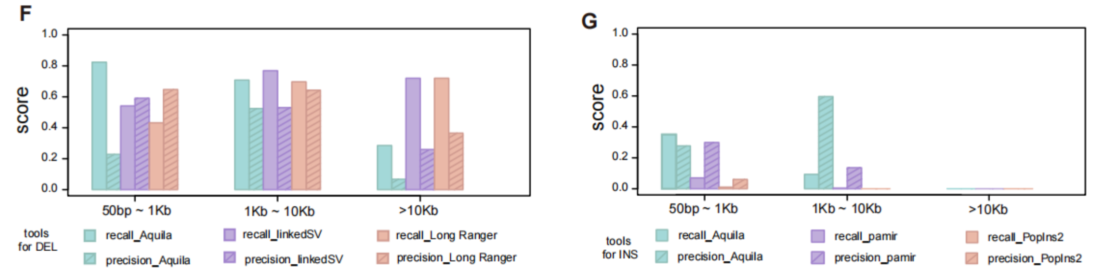

Figure 5. Evaluation of linked-read based detection of variation in the human genome. (F) The performance on detection of deletions using Aquila, LinkedSV and LongRanger. (G) The performance on detection of insertions using Aquila, pamir and PopIns2.

Analysis:

Lines 166-169: Figure 1 panel A1 vs. B1- why do the distribution of estimated fragment sizes from the 10x datasets look so different in metagenomic vs. human samples, when there is reasonable consistency in TELL-Seq and stLFR datasets?

Answer:

Thank you for the comments. In the panels A1 and B1 of Figure 1, we described **the number of fragments per barcode** rather than the **fragment sizes**. We observed, for 10x Genomics, the peak barcode count is around 7 for human genome sequencing and around 12 for metagenome sequencing. This significant difference is attributed to the barcoding method employed. In the case of 10x Genomics, barcoding takes place within water-in-oil droplets, which require a specialized instrument for droplet generation. The number of DNA fragments present in each droplet is influenced by the size of the DNA fragments. Typically, DNA fragments from human genome are longer, resulting in fewer fragments being included in each droplet. On the other hand, microbial DNA fragments are shorter, allowing for more fragments to be accommodated within a droplet. In

contrast, neither TELL-Seq nor stLFR utilizes droplets for barcoding. Instead, the barcoding reactions occur in an open environment and are partitioned using beads alone. Generally, each bead is conjugated with at least one unique barcode sequence on its surface and can capture 1-2 DNA fragments. For stLFR and TELL-Seq, the number of DNA fragments is not dependent on the length of the DNA fragments, resulting in a similar number of fragments per barcode for both human and metagenomic sequencing.

Lines 182-184: Figure 3A- why is LRTK's taxonomic classification quality generally lower than the of the tools? At least in terms of recall, it should perform better as mapping reads to reference genomes should have a lower false negative rate than k-mer-based tools. Also, what is the threshold for having detect a taxon? Is it just any number of reads or is there a minimum bound?

Response:

Thank you for pointing out this problem. The low recall values are because of the stringent threshold we used to filter false positive species. In the original manuscript, we kept microbial genomes with at least 60% of the windows covered by sequencing reads. In the revised manuscript, we have refined the taxonomic classification module based on the simulated dataset to improve the performance of species detection. The new strategy is based on genome coverage and we kept the microbial genomes with a coverage higher than 40% and covered bases of more than 500 kb. Currently, LRTK outperforms other tools in the simulated dataset and ranks second on the ATCC-MSA-1003 dataset. In the revised manuscript, we update related descriptions **from line 208 to line 226:**

Since LRTK would also detect genomic variants at the same time, we primarily focus on species with a relative abundance higher than 0.1%.

Figure 3A:

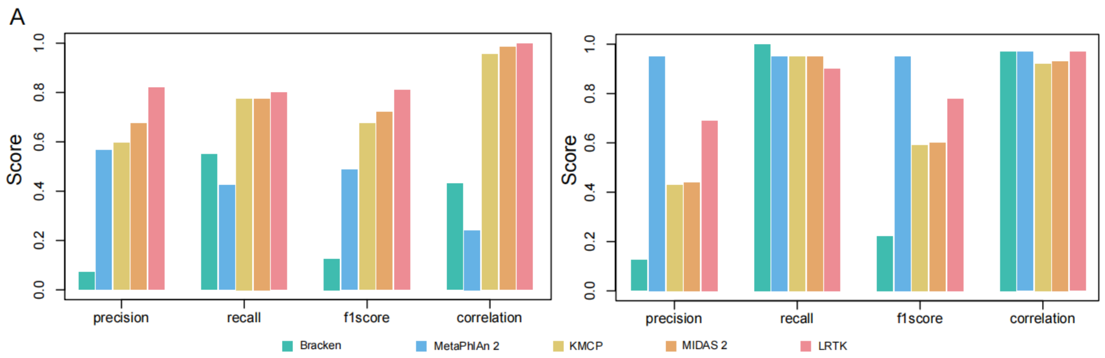

Figure 3. Comparison of linked-read based metagenomic quantification, SNV identification and phasing. (A) Evaluation of tools to quantify taxonomic abundance based on the linked-reads from simulated dataset (left panel) and ATCC-MSA-1003 (right panel).

Lines 187-188: Figure 3B- at least 15% of each caller's set of variants is unique to the variant, while a maximum of 50% is universal. I'd not interpret that as consistency.

Response:

Thank you for your comments. In the revised manuscript, we conducted a comprehensive

investigation of metagenomic single nucleotide variants (SNVs) and revised our conclusions in **Figure 3B.** We performed microbial SNV calling based on stLFR linked-reads from the ATCC-MSA-1003 using three variant calling tools: FreeBayes, inStrain, and SAMtools. For FreeBayes and SAMtools, we further removed the SNVs if the total depths were less than 6 , the number of reads supporting alternative allele was less than 2, and SNV qualities were below 15. We did not perform further quality control for the SNVs from inStrain as it did not generate SNV quality scores.

We found that approximately 21% of the total SNVs were jointly detected by all three tools. These SNV, account for around 76% and 80% of the total SNVs detected by FreeBayes and SAMtools, respectively. However, in the special case of inStrain, more than 50% of the SNVs were uniquely detected, suggesting inStrain is a more sensitive tool to detect microbial SNVs.

We have updated the related results in the revised manuscript **(lines 228-234)**:

"Comparing different SNV callers implemented in LRTK, we discovered that approximately 176,891 SNVs were jointly detected by FreeBayes, SAMtools and inStrain on stLFR linked-reads for ATCC-MSA-1003 (Figure 3B). These SNVs account around 76%, 80% and 21% of the total SNVs detected by FreeBayes and SAMtools and inStrain, respectively (Figure 3B). More than 50% of SNVs from inStrain can not be detected by FreeBayes and SAMtools, suggesting inStrain is the most sensitive tool to detect microbial SNVs."

Figure 3B:

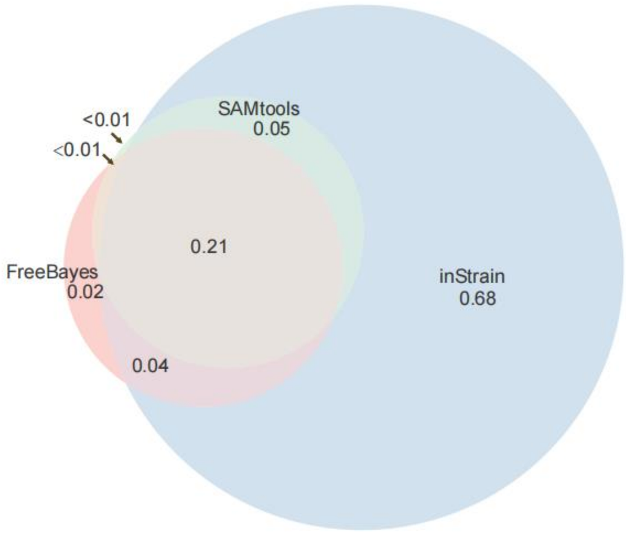

Figure 3. Comparison of linked-read based metagenomic quantification, SNV identification and phasing. (B) Comparing the performance of SAMtools, FreeBayes and inStrain to detect metagenomic SNVs.

Lines 192-193: Are you referring to allelic imbalance as it is popularly used to refer to expression variation between the two haplotypes of a diploid organism? This clearly doesn't apply in the case of bacteria. If this is not what you're referring to, please define and/or cite the applicable definition.

Response:

Thanks for the comments. Here, the term "genome-wide mirrored allele imbalance" refers to the

presence of allele imbalance across a large fraction of the genome. This concept is derived from the notion of mirrored subclonal allelic imbalance observed in cancer genomes. Mirrored subclonal allelic imbalance events are identified when the maternal allele is gained or lost in one tumor region and the paternal allele is gained or lost in a different region [4]. It is important to note that the number of genome copies involved in this phenomenon is not limited to two or three; it can vary and be relatively high. Mirrored subclonal allelic imbalance plays a critical role in tumor evolution and the progression of cancer. Analyzing and visualizing the directional imbalance of alleles in multi-sample settings allows for the inference of these events.

It has been reported that resident populations of gut bacteria exhibit a relatively clonal structure, with a few dominant strains of each species at intermediate or high frequencies [5][6]. In metagenome, we used MAI to describe the dynamic changes of multiple strains under one species. MAI may be identified when the reference allele is gained or lost at one time point while the altered allele (minor) allele is gained or lost at a different time point. Based on multiple time-series metagenomic sequencing data from the same individual, Roodgar et al. tracked the longitudinal trajectories of genetic elements and observed evidence of clonal evolution, where pre-existing single nucleotide variants were swept at an almost genome-wide level [7]. Linked reads provide a means to directly visualize these events, offering valuable evidence in their study. We have updated the related descriptions in the **Methods** section of the revised manuscript from **line 541 to line 577**.

Lines 201-208: It's odd that despite the 10x datasets having the largest estimated fragment size, they have some of the smallest genome fractions, NGA50, and NA50. Why is this? Are they just smaller datasets, on average?

Response:

To mitigate the impact of data size, we subsampled approximately 20 Gb of sequencing data from ATCC-MSA-1003 for each of the three linked-read technologies. Our comparison focused on metagenome assemblies from linked-read assemblers: Athena, cloudSPAdes, and Pangaea. Interestingly, we noted that the assembly from the 10x Genomics dataset exhibited the lowest NA50 and N50 values among the three technologies. This trend was also observed with metaSPAdes. Conversely, there were no discernible variations in the NA50 and N50 values derived from MEGAHIT assemblies. These findings imply that the disparities in assembly performance may not be solely attributed to differences in data size.

We observed that there was a consistent decrease in the number of fragments per barcode ($N_{F/P}$) for the stLFR and TELL-Seq technologies. For example, the $N_{F/P}$ was 1.48 for stLFR and it means that the related reads for each barcode may come from the same fragment. The lower numbers indicated that stLFR and TELL-Seq have superior performance in deconvolving linked-reads from different species than 10x Genomics. This may be the reason for the superior assembly performances on stLFR and TELL-Seq linked-reads than the 10x Genomics linked-reads.

Miscellaneous:

UHGG: Please mention the fact that the UHGG is the default database, as well as whether or not the user will be able to supply their own databases.

Response:

Besides the default UHGG dataset, LRTK can also allow users to provide their customized reference genomes by utilizing the "--database" parameter in the align function.

We also updated related descriptions from **line 462 to line 466** in the revised manuscript:

"For metagenomic sequencing data, we developed a tiered alignment approach to align the linked read to microbial genomes using the aforementioned modified EMA. The default reference genomes for the human gut metagenome were downloaded from UHGG. For non-gut samples, we used the GTDB as the reference database, but user could also construct their own custom database."

Line 363: What does `{M}` refer to?

Response:

M is the abbreviation of multi-mapped reads. In the manuscript, we used M to represent the number of multiple aligned reads on each bacterial genome.

Line 369: What does `U` mean here? Is this the number of uniquely aligned reads in one of the windows N that a multi-aligned read aligns to?

Response:

U is the abbreviation of unique mapped reads. In the manuscript, we used U to represent the number of uniquely aligned reads on each bacterial genome.

Lines 371-372: What does 'n% most closely covered windows' refer to?

Response:

In LRTK, following the initial classification from the alignment file, we apply a filtration step based on genome coverage. In the original manuscript, we employed a window-based approach to calculate bacterial genome coverage. We divided the genome into windows and removed the windows with extreme number of reads (> <), and then calculated the average coverage using the remaining windows. The **"n% most closely covered windows"** is equal to the remaining windows.

In the revised manuscript, we have updated the taxonomic classification module and replaced this window-based filtration approach with a total genome coverage approach. We only kept the microbial genomes whose coverage is higher than 40% and covered base is more than 500 kb in the new approach. The related description is updated **from line 462 to line 493** in the revised manuscript.

Lines 399-405: How are SNVs chosen for MAI analysis from the three available SNV callers?

Response:

This analysis is conducted based on the sets of single nucleotide variants (SNVs) identified by one of the three available SNV callers. For each interested species, SNVs are extracted from each sample and merged into a combined SNP set. In this process, one sample is selected as the reference for the interest species. For each species which is present in two samples, the minor allele is determined based on the reference sample. The BAF is calculated as the ratio of the minor allele count to the total allele count. Based on the combined SNP set, BAF values for the interested sample are then compared with the BAFs of the reference sample to identify any mirrored allelic imbalances. In the revised manuscript, we updated related description from **line 539 to line 557**.

Lines 653-656: Which dataset was used for quality evaluation?

Response:

To ensure fair comparisons among the three sequencing technologies, we prepared the same volume of data for 10x Genomics, stLFR and TELL-Seq sequencing. Specifically, for human genome sequencing, we extracted around 110 Gb base from sample NA12878. For metagenome sequencing, we extracted approximately 20 Gb base from sample ATCC-MAS-1003.

Line 665: What do the abbreviations BAF and T stand for?

Response:

"T" is the abbreviation used to represent "time point" in our study. In **Figure 3D**, the labels on the right side correspond to the sample names in dataset D1 **(Table S2)**. In our study, "BAF" stands for "B allele frequency." To provide a more comprehensive understanding, we have included detailed descriptions of BAF in the legend for **Figure 3 (lines 884-885)**:

"(D) SNV based strain phasing in pairwise samples. The right label shows the sample name in D1. BAF is the abbreviation of B allele frequency."

Reviewer #3: The paper titled "LRTK: A Platform-Agnostic Toolkit for Linked-Read Analysis of Both Human Genomes and Metagenomes" by Yang et al. is dedicated to the development of a unified interface for linked-read data processing.
The problem described in the paper indeed exists; each linked-read technology requires complex preprocessing steps that are not straightforward or efficient. The idea of consolidating multiple tools in one place, with some of them modified to handle multiple data types, is commendable. Overall, I am supportive of this paper. My main concern, however, is that the impact of linked-read applications in the paper appears to be exaggerated, and the authors need to provide more context in their presentation. Also, some parts of the paper are vague described. I will elaborate on my concerns in more detail below.

We thank the reviewer for these valuable comments. We have carefully revised the manuscript and added more detailed information for the methods and results.

X) Linked-read sequencing generates reads with high base quality and extrapolative 64 information on long-range DNA connectedness, which has led to significant 65 advancements in human genome and metagenome research[1-3]. - Citations 1-3 do not really tell about advancements in human genome and metagenome research, these are technologies papers. Similar problem can be found in "Despite the limitations that genome specificity…" paragraph. Authors cited and described several algorithms, that are not really genomic studies. E.g. "stLFR[2] has found application in a customized pipeline that has been developed to first convert its raw reads into a 10x-compatible format, after which Long Ranger is applied for downstream analysis." is not an example of genomic study, but a pipeline description.

Response:

We checked our manuscript thoroughly and updated the citations to focus more on papers related to genomic studies, replacing those that were previously centered on technological aspects.

To support the "significant advancements in human genome and metagenome research", we cited four relevant studies on **line 66** in the revised manuscript:
"1. Bergström A, McCarthy S A, Hui R, et al. Insights into human genetic variation and population history from 929 diverse genomes[J]. Science, 2020, 367(6484): eaay5012.
2. Hadi K, Yao X, Behr J M, et al. Distinct classes of complex structural variation uncovered across thousands of cancer genome graphs[J]. Cell, 2020, 183(1): 197-210. e32.
3. Dréau A, Venu V, Avdievich E, et al. Genome-wide recombination map construction from single individuals using linked-read sequencing[J]. Nature Communications, 2019, 10(1): 4309.
4. Roodgar M, Good B H, Garud N R, et al. Longitudinal linked-read sequencing reveals ecological and evolutionary responses of a human gut microbiome during antibiotic treatment[J]. Genome research, 2021, 31(8): 1433-1446."

Additionally, we have corrected the sentence "*Despite the limitations that genome specificity…*" as "Despite the limitations that genome specificity places on research scope, linked-read sequencing has already been successfully applied to many human genomic studies." And we cited four relevant studies on **line 90** in the revised manuscript:

“1. Dréau A, Venu V, Avdievich E, et al. Genome-wide recombination map construction from single individuals using linked-read sequencing[J]. Nature Communications, 2019, 10(1): 4309.
2. Marks P, Garcia S, Barrio A M, et al. Resolving the full spectrum of human genome variation using Linked-Reads[J]. Genome research, 2019, 29(4): 635-645.
3. Viswanathan S R, Ha G, Hoff A M, et al. Structural alterations driving castration-resistant prostate cancer revealed by linked-read genome sequencing[J]. Cell, 2018, 174(2): 433-447. e19.
4. Greer S U, Nadauld L D, Lau B T, et al. Linked read sequencing resolves complex genomic rearrangements in gastric cancer metastases[J]. Genome medicine, 2017, 9(1): 1-17.”

X) Table S1 does not improve the paper, I would say it does completely the opposite. LongRanger is not a toolkit, it should be considered as read alignment tool that outputs some SVs and haplotypes along the way. So LongRanger vs LRTK comparison does not make sense to me. There are other tools that solve metagenome assembly problem, human assembly problem, call certain classes of SVs etc.

Response:

We agree that Long Ranger is primarily a read alignment tool and comparing it directly with LRTK is not justifiable. Following the suggestions, we removed the Table S1 in the revised manuscript.

x) I think incorporating longranger is important, since its performance is reported to be better than EMA for human samples and it is also more popular than EMA. Is it possible and have you tried doing it?

Response:

Thank you for the suggestions. Because Long Ranger is not open source and has redundant functions with LRTK, we have integrated Lariat [8], the alignment algorithm used in Long Ranger, in the latest version of LRTK. Subsequently, we refined LRTK to ensure that Lariat was compatible with sequencing data from the three platforms.

x) I would remove exaggerations such as "myriad" from the text. The scope of linked-reads is pretty limited nowadays. I agree that linked-reads might be useful in metagenomics/transcriptomics and other scenarios that were mentioned in the text, but the number of studies is very limited especially nowadays, and was not really big when 10X platform was on the rise

Response:

We agree that linked-read sequencing is not a mainstream technology nowadays and has been used in a limited number of studies. We have revised our language to avoid overstatement. **In line 90,** we have replaced "a myriad of" with "many human genomic studies" to represent the current scope of research in this area more precisely.

x) "LRTK reconstructs long DNA fragments" - when people talk about long fragment reconstruction, they usually mean moleculo-style reconstruction through assembly. This

reconstruction resemble" barcode deconvolution", described in Danko et al, and Mak et al. So I would stick to this terminology

Response:

We appreciate the comments from the reviewer. The term "barcode deconvolution" may be more appropriate to characterize the methodology to reconstruct long DNA fragments. Similar to QuickDeconvolution and Ariadne, our method also tried to categorize reads into distinct DNA fragments according to their aligned genomic positions. In light of this, we have substituted the phrase "LRTK supports the reconstruction of long DNA fragments" with "LRTK reconstructs long DNA fragments by barcode deconvolution" in the revised manuscript **(line 185)**.

x) it is important to note that, Aquila, LinkedSV and VALOR2 are linked-read specific tools, while FreeBayes, Samtools and GATK are short-read tools. Also, provide target SV length for both groups of tools.

Response:

The comparisons among short-read based tools are also meaningful. Because barcode-aware aligners usually enhance the alignments on complex genomic regions, such as repeat regions that are typically difficult for short-read sequencing to resolve. However, the influence on downstream analysis, such as single nucleotide variant (SNV) calling remains unclear.

The utilization of co-barcoded reads to improve the detection of structural variations (SVs) is one of the key advantages of linked read sequencing. Generally speaking, simple SVs consist of insertions and deletions that are larger than 50 bps. Insertions and deletions (indels) which are shorter than 50 bps are defined as small indels and could be detected by the aforementioned short-read based tools. In theory, the linked read based tools should have better performance to detect indels larger than 50 bps. As a result, LRTK leverages short-read based tools for the detection of small indels and linked-read-specific callers for SVs. To clarify these concepts, we have included the specific target lengths for INDEL categorization from **line 141 to line 145** in the revised manuscript.

"After reads alignment, LRTK offers users an option to select one of the well-known tools for variant calling, including FreeBayes, SAMtools, GATK for SNV and small INDEL (Insertion and Deletion, < 50 bps) calls; and Aquila, LinkedSV and VALOR2 for structural variant calls (SVs) (>50 bps)."

x) There are some minor problems with Github readme. E.g. "*parameters". Also, I don't understand how to use conversion in real life… E.g. 10X Genomics data often comes as a folder with multiple gzipped R1/R2/I1 files. I don't understand how would I use it in that case.

Response:

For the problems on Github readme, we have corrected and updated the documentation about LRTK. We try our best to ensure the documentation's clarity and accuracy, thus facilitating the proper application of LRTK and supporting its users.

The conversion command (preprocess operation) is a critical step in preparing sequencing

data for accurate alignment with barcode-aware aligners such as EMA. This process involves attaching barcodes to reads, correcting errors within the barcodes, and sorting the reads by barcode, which are also used in Long Ranger. Additionally, LRTK is also equipped with the functions to handle stLFR and TELL-Seq sequencing technologies. In practical scenarios, as mentioned by the reviewer, we often encounter many samples, each containing multiple gzipped files - this is commonly observed in human sequencing data. According to our experience, there are usually two strategies to solve this problem. The first one is to merge multiple gzipped files into a single pair of gzipped files (R1/R2) and then submit them to process with tools like LRTK. This is usually carried out on high-performance computing nodes equipped with large memory and substantial CPUs. The second one is to process each pair of gzip files (R1/R2) independently with the conversion command and alignment command in LRTK, followed by merging the alignment files for each sample. This approach is usually implemented on a computing cluster with substantial computing nodes, each with limited memory and CPUs.

x) Please cite or explain why this is happening (not only when) - "A known concern with stLFR linked-read sequencing is the loss of barcode specificity during analysis."

Response:

In our study, the phrase “loss of barcode specificity”, implies that certain barcode information was overlooked during the data analysis process. In some studies, the TELL-seq 18 bps barcodes and stLFR 30 bps barcodes were usually converted into a 10x-compatible data format (16 bps), which is a necessary step for compatibility with the Supernova assembler and Long Ranger aligner (https://sagescience.com/wp-content/uploads/2020/10/TELL-Seq-Software-Roadmap-User-Guide-2.pdf and https://github.com/BGI-Qingdao/stlfr2supernova_pipeline). For stLFR and TELL-Seq, this step will merge linked-reads with different barcodes to the same barcode from 10x Genomics. The number of original barcodes of TELL-Seq is approximately 2 billion; however, this figure was reduced to about 4.7 million subsequent to the conversion process. For stLFR, as we observed, the type of barcodes decreased by around 50% for the sample NA12878 during the stlfr2supernova process. In the revised manuscript, we added the detailed descriptions **from line 98 to line 104**:

“In addition, the format conversion may induce the loss of barcode specificity as the type of barcodes decrease dramatically. This occurs when the stLFR and TELL-Seq linked-reads are typically converted into a 10x-compatible format to run Long Ranger and Supernova (https://github.com/BGI-Qingdao/stlfr2supernova_pipeline and https://sagescience.com/wp-content/uploads/2020/10/TELL-Seq-Software-Roadmap-User-Guide-

x) I don't understand what is "Length-weighted average (μFL) and unweighted average (WμFL) of DNA 688 fragment lengths." from the figure. One of them is just an average and what about second? Figure looks confusing

Response:

Sorry for the unclear description. In our study, we calculated the average length of DNA

fragments, denoting it as μFL (average Fragment Length). Additionally, we computed the N50 value of all DNA fragments, which we refer to as WμFL (Weighted average Fragment Length). The N50 metric is similar to the average length, yet it places more emphasis on the longer contigs within the dataset. For a comprehensive understanding, we cited a paper by Zhang L, *et al* to describe the calculation in detail. We also included a brief explanation of this metric in the legend for **Figure S2D** in the revised manuscript **(lines 910-913)**:

"(D) Length-weighted average (μFL) and unweighted average (WμFL) of DNA fragment lengths. Here, μFL was calculated as the mean DNA fragment length while WμFL was calculated as the N50 value of all the DNA fragments."

x) LRTK supports reconstruction of long DNA fragments - this section describes something else. More about statistics and data QC

Response:

Thanks for the comments. Currently, the long DNA fragments mainly serve as a measure of the quality of linked-read sequencing libraries. In our previous study, we have shown the statistics of the reconstructed long DNA fragments (E.g. C_F, C_R et al.) can significantly affect human genome assembly [1], structural variants call [2] and metagenome assembly [3]. Also, these statistics are also critical indicators to evaluate the quality of the library and provide essential insights for its improvement. For example, a low C_F suggests the need for increased DNA input, while the same low C_R indicates a requirement for generating additional reads. Once we obtain results from data analysis, it is crucial to discern whether any issues arise from the library's quality or from the data analysis tools themselves, such as those used for structural variant calls and phasing, which heavily rely on long DNA fragments. We add a paragraph in the discussion to further discuss the long fragment in the revised manuscript **(from line 373 to line 385)**.

x) LRTK promotes metagenome assembly using barcode specificity - please remove supernova, it was never a metagenomic assembler. Check cloudSPAdes instead

Response:

We removed the Supernova and evaluated the assembly performance of cloudSPAdes on ATCC-MSA-1003. In the revised manuscript, we substituted 'supernova' with 'cloudSPAdes' in **Figure 4A** and updated related descriptions to reflect this change **(from line 244 to line 255)**.

x) "The superior assembly performance we have observed" - superior compared to what? If so, some short-read benchmark should be included.

Response:

Sorry for the confusion. The "superior" refers to the performance of metagenome assemblers for linked-reads that perform better than that for short reads. In the revised manuscript, we incorporated MEGAHIT and metaSPAdes and compared them with the linked-reads based assemblers. We have also revised **Figure 4A** and manuscripts from **line 244 to line 264**.

x) "LRTK improves human genome variant phasing using long range information" - What dataset is this? What callset was used for ground truth? Briefly describe how comparisons were done?

Response:

Thanks for the comments. In the original manuscript, the title may be misleading. In the revised manuscripts, we changed the subtitle to “LRTK provides best practice for human genome variants detection and phasing”.

We compared the phasing performance between HapCUT2 and WhatsHap using the small variants from NA12878. But we did not include ground truth datasets for comparison. To perform fair comparison, the small variants (SNVs and INDELs) were identified using the same variant calling tool, for example, FreeBayes, across the three platforms. Subsequently, we run HapCUT2 with the parameter “--nf 1” and run WhatsHap with the parameter “--reference GRCH38” on the detected variants. The tool PhaseME [9] was then used to calculate the phased block size and phased rate to assess the phasing quality. We also updated related descriptions in the revised manuscripts from **line 528 to line 529.**

x) Figures 5F-G together are very confusing. First I don't expect tools like LinkedSV to have high recall (around 1.0) and low precision. Also, figure G is kind of subset of figure F, but results are completely different. Also use explicit notation. E.g. 50-1kbp and 1-10kbp mean completely different things.

Response:

We deeply thank the reviewer for the insightful observations. Following the suggestions provided, we have thoroughly re-examined our benchmarking results and are prepared to address the posed questions one by one.

Regarding the first inquiry about the high recall rate, we revisited the parameters and outputs from LinkedSV. The analysis was performed using the command '-v hg38 -t 64 --germline_mode', and LinkedSV typically generates three SV-related files: *small_deletions.bedpe, *large_cnv.bedpe, and *large_svcalls.bedpe. Upon reviewing the *small_deletions.bedpe file, we observed that the recall rate for deletions stands at 0.577, with a precision of 0.576. Breaking it down by size, we found that for small SVs (50 bps to 1 kb), the recall is 0.54 and precision is 0.59; for medium SVs (1kb ~ 10 kb), the recall is 0.77 with a precision of 0.53; and for large SVs (larger than 10 kb), the rates are 0.71 for recall and 0.25 for precision. Previously, we aggregated the identified deletions from all three files, which resulted in a recall rate of 0.99 and a precision of 0.21 for SVs larger than 10 kb. We have since amended **Figure 5F** and the related descriptions in the revised manuscript **(lines: 287-292)**:

“As the recall values illustrated in Figure 5F, Aquila has a higher recall value for 50 bps – 1 kb deletions (Aquila: 0.82; LinkedSV: 0.54; Long Ranger: 0.43). In contrast, LinkedSV and Long Ranger performed better in detecting deletions longer than 1 kb (Aquila: 0.29; LinkedSV: 0.71; Long Ranger: 0.72). Aquila also shows a better performance than Pamir and PopIns2 to detect insertions (Aquila: 0.35; Pamir: 0.07; PopIns2: 0.01) (Figure 5G).”

For the second question concerning **Figure 5F and 5G**, we utilized these figures to demonstrate the detection performance for deletions and insertions, respectively. The distinction between deletions and insertions is notably obvious, given that deletions can often be inferred from the reference genome, whereas insertions typically consist of novel sequences.

Lastly, regarding the third question about SV size, we acknowledge the previous descriptions were unclear and have made corrections in the revised manuscript. In **Figure 5F and 5G**, the phrase '50-1kbp' has been updated to '50 bps to 1 kb', and '1-10 kb' has been changed to '1 kb to 10 kb' for clarity.

Figure 5 F and G:

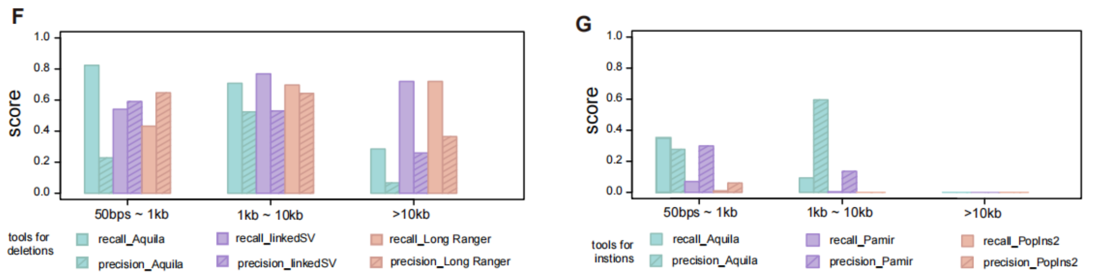

Figure 5. Evaluation of linked-read based detection of variation in the human genome. (F) The performance on detection of deletions using Aquila, LinkedSV and LongRanger. (G) The performance on detection of insertions using Aquila, Pamir and PopIns2.

x) We curated one benchmarking dataset and two real datasets to demonstrate the 307 performance of LRTK - what do you mean by "curation" here

Response:

Sorry for this inappropriate word. We have replaced the word “curated” with “included” in the revised manuscript **line 399.**

x) Why don't you use Tell-Seq barcode whitelist mentioned here - https://sagescience.com/wp-content/uploads/2020/10/TELL-Seq-Software-Roadmap-User-Guide-2.pdf

Response:

We agreed that the barcode whitelist provided in the user guide is also frequently utilized. According to the description in user guide, this whitelist comprises approximately 4 million barcodes, derived from the original 2 billion barcodes. It is commonly employed as a substitute for the default 10x Genomics whitelist used in Long Ranger and Supernova software packages. However, due to its significantly reduced size compared to the comprehensive whitelist, it has the potential to overlook critical data. Consequently, we opted for the original barcode datasets and adhered to the processing protocol delineated in Chen's paper [10] to ensure a thorough analysis.

x) Tiered alignment approach is vaguely introduced. It is not clear what "n% most closely covered windows." mean, or how do we select a subset of reference genomes for the second phase

Response:

In the revised manuscript, we have provided a detailed description about our Tiered Alignment methodology **(lines 467 to line 499)**.

In LRTK, following the initial classification from the alignment file, we apply a filtration step based on genome coverage. In the original manuscript, we employed a window-based approach to calculate bacterial genome coverage. We divided the genome into windows and removed the windows with extreme number of reads (> <), and then calculated the average coverage using the remaining windows. The **“n% most closely covered windows”** represents the remaining windows.

In the revised manuscript, we have updated the taxonomic classification module and replaced this window-based filtration approach with a total genome coverage approach. We only kept the microbial genomes whose coverage is higher than 40% and covered base is more than 500 kb in the new approach.

References

1. Zhang L, Zhou X, Weng Z, Sidow A. Assessment of human diploid genome assembly with 10x Linked-Reads data. *Gigascience*. Gigascience; 2019; doi: 10.1093/GIGASCIENCE/GIZ141.

2. Zhang L, Zhou X, Weng Z, Sidow A. De novo diploid genome assembly for genome-wide structural variant detection. *NAR Genomics Bioinforma*. Oxford Academic; 2020; doi: 10.1093/NARGAB/LQZ018.

3. Zhang L, Fang X, Liao H, Zhang Z, Zhou X, Han L, et al.. A comprehensive investigation of metagenome assembly by linked-read sequencing. *Microbiome*. BioMed Central Ltd; 2020; doi: 10.1186/S40168-020-00929-3/FIGURES/4.

4. Jamal-Hanjani M, Wilson GA, McGranahan N, Birkbak NJ, Watkins TBK, Veeriah S, et al.. Tracking the Evolution of Non-Small-Cell Lung Cancer. *N Engl J Med*. N Engl J Med; 2017; doi: 10.1056/NEJMOA1616288.

5. Vasquez KS, Willis L, Cira NJ, Ng KM, Pedro MF, Aranda-Díaz A, et al.. Quantifying rapid bacterial evolution and transmission within the mouse intestine. *Cell Host Microbe*. Cell Press; 2021; doi: 10.1016/j.chom.2021.08.003.

6. Truong DT, Tett A, Pasolli E, Huttenhower C, Segata N. Microbial strain-level population structure & genetic diversity from metagenomes. *Genome Res*. Cold Spring Harbor Laboratory Press; 2017; doi: 10.1101/GR.216242.116/-/DC1.

7. Roodgar M, Good BH, Garud NR, Martis S, Avula M, Zhou W, et al.. Longitudinal linked-read sequencing reveals ecological and evolutionary responses of a human gut microbiome during antibiotic treatment. *Genome Res*. Cold Spring Harbor Laboratory Press; 2021; doi: 10.1101/GR.265058.120.

8. Bishara A, Liu Y, Weng Z, Kashef-Haghighi D, Newburger DE, West R, et al.. Read clouds uncover variation in complex regions of the human genome. *Genome Res*. Genome Res; 2015; doi: 10.1101/GR.191189.115.

9. Majidian S, Sedlazeck FJ. PhaseME: Automatic rapid assessment of phasing quality and phasing improvement. *Gigascience*. Oxford University Press; 2020; doi: 10.1093/gigascience/giaa078.

10. Chen Z, Pham L, Wu TC, Mo G, Xia Y, Chan PL, et al.. Ultralow-input single-tube linked-read

library method enables short-read second-generation sequencing systems to routinely generate highly accurate and economical long-range sequencing information. *Genome Res*. Cold Spring Harbor Laboratory Press; 2020; doi: 10.1101/gr.260380.119.

Manuscript

Click here to access/download;Manuscript;LRTK.revised.clean.final.track_c

LRTK: A platform agnostic toolkit for linked-read analysis of both human ~~genomes~~genome and ~~metagenomes~~metagenome

Chao Yang[1†], Zhenmiao Zhang[1†], Yufen Huang[2,3], Xuefeng Xie[4], Herui Liao[5], Jin Xiao[1], Werner Pieter Veldsman[1], Kejing Yin[1], Xiaodong ~~Fang[2,3]~~Fang[3,4*], Lu Zhang[1,6*]

[1]Department of Computer Science, Hong Kong Baptist University, Hong Kong SAR, Hong Kong

[2]BGI Research, Shenzhen 518083, China

[3]BGI Genomics, Shenzhen 518083, China

[4]BGI Research, Sanya 572025, China

[5]Department of Electrical Engineering, City University of Hong Kong, Hong Kong SAR, Hong Kong

[6]Institute for Research and Continuing Education, Hong Kong Baptist University, China

[†]These authors contributed equally to this work.

*To whom correspondence should be addressed: E-mail:

fangxd@genomics.cn ,

ericluzhang@hkbu.edu.hk.

Style Definition: Normal: Font: (Default) SimSun, (Asian) SimSun, 12 pt, Left, Widow/Orphan control
Style Definition: Heading 1
Style Definition: Heading 2
Style Definition: Heading 3
Style Definition: Comment Text
Style Definition: Body Text
Style Definition: Balloon Text
Style Definition: Footer
Style Definition: Header
Style Definition: Title
Style Definition: Abstract-Text
Style Definition: List Paragraph
Style Definition: p
Style Definition: Author-Affiliation
Style Definition: corrs-au

Abstract

Linked-read sequencing technologies generate high~~-~~-base quality short-reads that contain extrapolative information on long-range DNA connectedness. These advantages of linked-read technologies are well~~-~~-known and ~~has~~have been demonstrated in many human genomic and metagenomic studies. However, existing linked-read analysis pipelines (e.g., Long Ranger) were primarily developed to process sequencing data from the human genome and are not suited for analyzing metagenomic sequencing data. Moreover, linked-read analysis pipelines are typically limited to one specific sequencing platform. To address these limitations, we present the Linked-Read ToolKit (LRTK), a unified and versatile toolkit for platform agnostic processing of linked-read sequencing data from both human ~~genomes~~genome and ~~metagenomes~~metagenome. LRTK provides functions to perform linked-read simulation, barcode sequencing error correction, ~~read cloud assembly,~~ barcode-aware read alignment and metagenome assembly, reconstruction of long DNA fragments, taxonomic classification and quantification, as well as barcode-assisted genomic variant calling and phasing. LRTK has the ability to process multiple samples automatically, and provides ~~the user~~users with the option to generate reproducible reports during processing of raw sequencing data and at multiple checkpoints throughout downstream analysis. We applied LRTK on ~~two benchmarking and three real~~ linked-~~read data sets~~reads from simulation, mock community and real datasets for both ~~the~~ human genome and metagenome. We ~~showcase~~showcased LRTK's ability to

Formatted: Font: 12 pt

generate comparative performance results from ~~the~~ preceding benchmark ~~study~~studies and to report these results in publication-ready HTML document plots. LRTK provides comprehensive and flexible modules along with an easy-to-use Python-based workflow for processing linked-read sequencing datasets, thereby filling the current gap in the field caused by platform-centric genome-specific linked-read data analysis tools.

~~Key words~~Keywords: linked-read sequencing, 10x Genomics, TELL-Seq, stLFR, metagenome, human genome

Introduction

Linked-read sequencing generates reads with high base quality and extrapolative information on long-range DNA connectedness, which has led to significant advancements in human genome and metagenome research[1–3]. It circumvents the typical lack of long-range DNA information in short-read sequencing, and the high error rates and large initial DNA load requirements of long-read sequencing (e.g., Oxford Nanopore and Pacific Bioscience). These advantages of linked-read sequencing are invaluable when dealing with challenging cases of low-input clinical samples, such as cancer tissues or infectious disease samples. Linked-read technology furthermore promotes haplotype construction and the detection of complex structural variations[4], and its relatively low cost enables the application in large cohort studies.

Linked-read sequencing platforms, such as 10x Genomics linked-read (10x Genomics; now discontinued) and the newly developed single-tube long fragment read (stLFR)[2] and transposase enzyme linked long-read sequencing (TELL-Seq)[3], hold much promise in the metagenomics area. The hidden long-range information they provide enables local assembly of co-barcoded reads and thus significantly increases the number of high quality MAGs[5]. In longitudinal sequencing data sets[6–8], the barcodes associated with linked-read promote the phasing of variants and refine the identification of intra-host evolution of gut microbiota. In some

~~complex environments, such as soil, linked-read sequencing has been shown to aid in the investigation of soil microbe genomes[9]. However, existing linked-read pipelines are mainly designed for use with the human genome, which points to an urgent need for appropriate metagenome analysis toolkits **(Table S1)**.~~

~~Despite the limitations that genome specificity places on research scope, linked-read sequencing has already been successfully applied to a myriad of genomic studies. For example, Long Ranger[10] performs barcode-aware read alignment and implements modules for variant calling and phasing using 10x Genomics linked-read. Tell-Sort[3] is a Docker-based pipeline to process raw TELL-Seq reads, and detect and phase genomic variants. stLFR[2] has found application in a customized pipeline that has been developed to first convert its raw reads into a 10x-compatible format, after which Long Ranger is applied for downstream analysis. The former pipeline however typically requires a lot of random-access memory and its data format conversion process is time consuming. Beyond the preceding examples that validate the inherent usefulness of linked-read technology, our search of the literature furthermore revealed a lack of unified and open-source toolkits that are compatible with the different linked-read platforms.~~

Linked-read sequencing generates short-reads with high base quality and extrapolative information on long-range DNA connectedness, which has led to significant advancements in human genome and metagenome research [1–4]. It circumvents the typical lack of long-range DNA information in short-read sequencing, and the high error rates and large initial DNA load requirements of long-

read sequencing (e.g., Oxford Nanopore and Pacific Bioscience). These advantages of linked-read sequencing are invaluable when dealing with challenging cases of low-input clinical samples, such as cancer tissues or infectious disease samples. Linked-read technology furthermore promotes haplotype construction and the detection of complex structural variations [5], and its relatively low cost enables the application in large cohort studies.

Linked-read sequencing platforms, such as 10x Genomics linked-read (10x Genomics; now discontinued) and the newly developed single-tube long fragment read (stLFR) [6] and transposase enzyme linked long-read sequencing (TELL-Seq) [7], hold much promise in the metagenomics area. The hidden long-range information they provide enables local assembly of co-barcoded reads and thus significantly increases the number of high-quality metagenome-assembled genomes (MAGs) [8]. In longitudinal sequencing datasets [3,9,10], the barcodes associated with linked-read promote the phasing of genomic variants and refine the identification of intra-host evolution of gut microbiota. In some complex environments, such as soil, linked-read sequencing has been shown to aid in the investigation of the involved microbial genomes [11,12]. However, existing linked-read pipelines are mainly designed for use with the human genome, which points to an urgent need for appropriate metagenome analysis toolkits.

Despite the limitations that genome specificity places on research scope, linked-read sequencing has already been successfully applied to many human genomic studies [2,13–15]. Some toolkits have been developed to facilitate the investigation of

the human genome. For example, Long Ranger [16] performs barcode-aware read alignment and implements modules for genomic variant calling and phasing using 10x Genomics linked-reads. Tell-Sort [7] is a Docker-based pipeline to process TELL-Seq linked-reads, for genomic variants detection and phasing. stLFR has found application in a customized pipeline that has been developed to first convert its raw reads into a 10x-compatible format, after which Long Ranger is applied for downstream analysis. This pipeline, however, typically requires a lot of random-access memory and its data format conversion procedure is time-consuming. In addition, the format conversion may induce the loss of barcode specificity as the type of barcodes decreases dramatically. This occurs when the stLFR and TELL-Seq linked-reads are typically converted into a 10x-compatible format to run Long Ranger and Supernova (https://github.com/BGI-Qingdao/stlfr2supernova_pipeline and https://sagescience.com/wp-content/uploads/2020/10/TELL-Seq-Software-Roadmap-User-Guide-2.pdf). Beyond the preceding examples that validate the inherent usefulness of linked-read technologies, our search of the literature furthermore revealed a lack of unified and open-source toolkits that are compatible with the different linked-read platforms.

To this end, we present Linked-Read ToolKit (LRTK), a unified and versatile toolkit to analyze both ~~metagenome~~metagenomic and human genome linked-read sequencing data derived from any of the three major linked-read sequencing platforms. LRTK delivers a suite of utilities to simulate linked-read sequencing data, barcode sequencing error correction, ~~read cloud assembly,~~ barcode-aware alignment~~,~~

and metagenome assembly, reconstruction of long DNA fragments, and genomic variant detection and phasing. LRTK is open-source, automatically produces HTML reports to summarize quality statistics as part of its pipeline, and generates publication-ready visualizations. We applied LRTK ~~on benchmarking metagenome~~to linked-reads from simulation, mock community and ~~human genome sequencing data (ATCC MSA-1003 and NA12878)~~real datasets to evaluate the performance of ~~10x Genomics, stLFR and TELL-Seq linked-read sequencing~~different technologies~~. We also applied LRTK on three real data sets to~~ and demonstrate its potential applications. Our results show that LRTK performs favorably on human ~~genomic~~genome linked-read sequencing data when compared to the pipelines designed specifically for a single platform~~ technologies,~~ and that it adequately allows for data analysis of metagenomic sequencing data.

Formatted: Font color: Black

Results

Overview of LRTK

~~We developed a comprehensive workflow (which we refer to as LRTK) that takes raw linked reads from 10x Genomics, stLFR, or TELL-Seq technologies, and processes these inputs in a multi-step checkpointed pipeline that ends with the generation of user-friendly reports. LRTK consists of two main sections for metagenomic and human genome sequencing data~~ ~~**(Figure 1)**~~~~. For metagenomic sequencing, LRTK includes the representative genomes from the UHGG[11] project as its default microbial reference genomes. LRTK uses a modified barcode-aware aligner based on EMA[12] to map~~

reads to the reference genomes. To reduce mapping errors, LRTK employs a tiered alignment approach to align the sequencing reads. First, it identifies candidate genomes from the alignment files, and extracts the related genomes from the reference genome database. Second, the reads are aligned to the selected reference genomes to provide correct metagenomic read assignments. If the single nucleotide variant (SNV) calling function is enabled, LRTK also performs variation calling and phasing for the identified species.

In the human genome section, LRTK directly maps reads to the human genome using the modified EMA[12] approach and marks the duplicates for each barcode. LRTK reconstructs long DNA fragments through greedy extension based on the read alignment coordinates if the co-barcoded reads are within a specified distance[13]. After mapping, LRTK provides user a choice of several well-known tools for variant detection, including FreeBayes[14], SAMtools[15], GATK[16] for calling small variants; and Aquila[17], LinkedSV[18] and VALOR2[19] for calling large variants. For variant phasing, LRTK utilizes HapCUT2[20] and WhatsHap[21] to call phasing blocks **(Table S2)**. Further details are available in the **Method** section.

We developed Linked-Read ToolKit (which we refer to as LRTK) that takes raw linked-reads from 10x Genomics, stLFR, or TELL-Seq technologies, and analyzes these inputs in a multi-step checkpointed pipeline that ends with the generation of user-friendly reports. LRTK consists of two main sections to process metagenomic and human genome linked-read sequencing data from mainstream technologies **(Figure 1)**. For metagenomic sequencing, LRTK includes the representative human gut microbial

genomes from the UHGG [17] project as its default reference genomes. We modified EMA [18] to perform barcode-aware alignment to be compatible with different platforms. To further reduce spurious mapping errors, LRTK eliminates the genomes if their coverage is below 40%. It performs genomic variation calling and phasing for these candidate genomes if the single nucleotide variant (SNV) calling function is enabled. In addition, LRTK is equipped with the functions to perform barcode-aware metagenome assembly and reconstruct MAGs from the metagenomic linked-read sequencing data.

In the human genome processing section, LRTK directly aligns linked-reads to the human reference genome using the same modified EMA followed by marking PCR duplicates for each barcode. LRTK reconstructs long DNA fragments through greedy extension based on the alignment coordinates of co-barcoded linked-reads [19]. After reads alignment, LRTK offers users an option to select one of the well-known tools for variant calling, including FreeBayes [20], SAMtools [21], GATK [22] for SNV and small INDEL (Insertion and Deletion, < 50 bps) calls; and Aquila [23], LinkedSV [24] and VALOR2 [25] for structural variant calls (SVs) (>50 bps). For variant phasing, LRTK utilizes HapCUT2 [26] and WhatsHap [27] to explore phasing blocks for SNVs and small INDELs **(Table S1)**. Further details are available in the **Methods** section.

Data Description

We incorporated one simulated dataset (S1), one dataset from a mock community (B1), and two real datasets from human gut microbiomes (D1 and D2) to evaluate

metagenomic sequencing analysis module of LRTK **(Table S2)**. For dataset S1**,** we simulated 11.9 Gb and 9.2 Gb linked-reads from 10x Genomics and stLFR for 40 complete bacterial genomes from NCBI RefSeq database, with lognormal abundance distribution (**Methods**), respectively **(Table S3)**. The dataset B1 was generated from ATCC-MSA-1003, containing 20 bacterial species with abundances varying from 0.02% to 18% **(Table S4).** The sequencing data volumes are 37.7 Gb, 111 Gb and 55 Gb for 10x Genomics, stLFR and TELL-Seq linked-reads, respectively. For the two real metagenomic datasets, D1 consists of 16 longitudinal human gut metagenomic sequencing datasets from a single individual, sequenced with an average of 24 Gb linked-reads on 10x Genomics platform [3]. The other dataset D2 contains around 99 Gb stLFR linked-reads from human gut metagenome[28]. For the human genome analysis section, we collected the linked-read sequencing datasets from NA12878, NA24143, NA24149 and NA24385 (**Table S2).** In addition, we performed linked-reads down-sampling to ensure fair comparisons among the three sequencing technologies. We down-sampled around 20 Gb metagenomic linked reads from ATCC-MSA-1003 for each platform. Similarly, around 110 Gb (~35X) linked reads from NA12878 were extracted for the three technologies **(Table S3)**.

LRTK ~~support~~supports multiple linked-read sequencing technologies

LRTK can handle linked-~~read data~~reads from various sequencing technologies, including but not limited to 10x Genomics, stLFR, or TELL-Seq. Initially, LRTK

Formatted: Indent: First line: 0"

converts the raw linked-~~read from 10x Genomics, stLFR and TELL-Seq~~reads into a unified FASTQ format, which contains a new field “BX:Z:” to ~~store~~include 16 ~~bp~~bps (10x Genomics linked-read), 18 ~~bp~~bps (TELL-Seq) and 30 ~~bp~~bps (stLFR) barcode sequences **(Figure S1)**.

~~Because barcode sequences are typically located at the beginning or end of linked-reads, LRTK includes functions to correct potential sequencing errors. For 10x Genomics linked-read, after correction, there are approximately 94.8% of barcode sequences on whitelist for NA12878 and 94.1% for ATCC-MSA-1003, respectively. The performance is comparable to the results obtained from Long Ranger, which retains 94.4% of barcode sequences for NA12878 and 93.6% for ATCC-MSA-1003. The retention rates were slightly lower for stLFR linked-read, with 85.4% for NA12878 and 90.7% for ATCC-MSA-1003. A known concern with stLFR linked-read sequencing is the loss of barcode specificity during analysis. This occurs when the data is typically converted into a 10x-compatible format to run Long Ranger or directly aligned using BWA[22] without considering the barcode information[3]. We made modifications to EMA[12] **(Methods)** to accommodate barcodes with varying lengths, including but not limited to stLFR. Our adapted approach proved to be more effective at preserving barcode specificity than the 10x-compatible format conversion approach.~~

~~LRTK supports reconstruction of~~Sequencing errors are often enriched at the start or end of linked-reads, where barcode sequences are typically found. LRTK includes functions to correct potential sequencing errors in barcodes. After error correction, there are approximately 94.8% and 94.1% of barcode sequences on the whitelist of 10x

Genomics linked-reads for NA12878 and ATCC-MSA-1003 **(Table S2)**, respectively. The performance is comparable to the results obtained from Long Ranger (94.4% for NA12878 and 93.6% for ATCC-MSA-1003). The corresponding rates are slightly lower for stLFR linked-read (85.4% for NA12878 and 90.7% for ATCC-MSA-1003) compared to 10x Genomics linked-read. As there is no whitelist for TELL-Seq, we could not perform the analysis for NA12878 and ATCC-MSA-1003 TELL-Seq linked-read sequencing data.

LRTK reconstructs long DNA fragments by barcode deconvolution

~~The quality of DNA library may significantly affect assembly performance and variant calling[23]. To evaluate the quality of linked-read sequencing libraries, we reconstructed the long DNA fragments for both human genome and metagenome sequencing data. We also calculated several key statistics to comprehensively compare the fragment properties across different linked-read sequencing technologies. The statistics include average coverage of short reads per fragment (C_R), average physical coverage of the genome by long DNA fragments (C_F), number of fragments per partition ($N_{F/P}$), fragment length (μ_{FL}), and unweighted and length-weighted DNA fragment length (μ_{FL} and $W\mu_{FL}$) **(Figure S2)**. On human genome sequencing data (B2, NA12878), LRTK detected approximately 7.36, 1.22 and 3.09 fragments per barcode and achieved average fragment lengths of 50.19, 62.28 and 80.04 kb for 10x Genomics linked-read, stLFR and TELL-Seq, respectively **(Figure 2B)**. On the metagenome~~

sequencing data (ATCC-MSA-1003), stLFR linked-read yielded the lowest $N_{F/P}$ ($N_{F/P}$ =1.54), while TELL-Seq linked-read yielded a slightly higher number ($N_{F/P}$ =4.26). Both numbers were much lower than that obtained from 10x Genomics linked-read ($N_{F/P}$ =16.61) **(Figure 2A)**, indicating that stLFR and TELL-Seq have superior performance in terms of the barcode specificity.

LRTK supportsThe quality of DNA sequencing library may significantly affect the performance of metagenome assembly [29], human genome assembly [19] and structural variant calling [30]. To evaluate the quality of linked-read sequencing libraries, we reconstructed the input long DNA fragments for both human genome and metagenomic sequencing data based on co-barcoded read alignments (**Methods**). We also calculated several key statistics to comprehensively compare the libraries from different linked-read sequencing technologies [19]. The statistics include average coverage of short-reads per fragment (C_R), average physical coverage of the genome by long DNA fragments (C_F), number of fragments per partition/beads ($N_{F/P}$), unweighted and length-weighted average DNA fragment length (μ_{FL} and $W\mu_{FL}$) **(Figure S2)**.

For NA12878 (**Table S3**), LRTK detected approximately 7.34, 1.95 and 3.60 fragments per barcode and achieved μ_{FL} of 45.9 kb, 16.46 kb and 54.78 kb for the libraries from 10x Genomics, stLFR and TELL-Seq, respectively **(Figure 2B)**. For ATCC-MSA-1003 (**Table S3**), stLFR linked-reads yielded the lowest $N_{F/P}$ ($N_{F/P}$ =1.48), while TELL-Seq linked-reads yielded a slightly higher number ($N_{F/P}$ =4.59). Both values were much lower than that obtained from 10x Genomics ($N_{F/P}$ =13.6) **(Figure**

2A), indicating that stLFR and TELL-Seq have superior performance in deconvolving linked-reads from different species.

LRTK enables metagenome taxonomic ~~classification~~quantification and genomic variant detection using barcode-aware alignment

~~Previous studies have amply demonstrated the huge potential of linked-reads in metagenomics[5–9]. Here, we also evaluated the performance of linked-reads in detecting taxonomic abundance and metagenomic variants. Based on standardized microbial genome databases such as GTDB[24] and UHGG[11], we developed a reference genome based computational framework to identify species and SNVs from metagenomic sequencing data. Using the ATCC-MSA-1003 benchmarking data[23], we observed that LRTK had a comparable performance with *k-mer* based tools such as KMCP[25] and marker-gene based tools, MIDAS2[26] **(Figure 3A)**. For high abundance species, LRTK could accurately identify SNVs from the alignment files and use barcode information to estimate genetic linkages and infer potential haplotypes. Comparison of metagenome SNV calling tools in the LRTK pipeline showed high consistency among FreeBayes[14], inStrain[27] and SAMtools[15] **(Figure 3B)**. We applied LRTK on a real longitudinal linked-read metagenomic data set (D1)[6] to evaluate LRTK's performance in practice. LRTK revealed the taxonomical composition and genomic variations for each sample. Using multiple related samples, LRTK also identified a genome-wide mirrored allele~~

~~imbalance (MAI) for species *Alistipes finegoldii*, which may suggest intra-host strain evolution over time **(Figure 3C and D)**.~~

Previous studies have amply demonstrated the huge potential of linked-reads in metagenomic researches [3,8,9,11]. Here, we evaluated the performance of linked-reads in detecting taxonomic abundance and genomic variants using LRTK. In LRTK, we developed a computational pipeline to detect and quantify microbes using microbial reference genomes. LRTK can better identify the involved microbes based on their genome coverage (F1=0.81) than the existing k-mer based tools: Bracken [31] (F1=0.13), and KMCP [32] (F1=0.67); and marker gene based tools: MetaPhlAn2 [33] (F1=0.49), and MIDAS 2 [34] (F1=0.72), on the simulated stLFR linked-reads (**Figure 3A**). For the stLFR linked-reads from ATCC-MSA-1003, LRTK also demonstrated a superior performance (F1=0.78) than Bracken (F1=0.22), KMCP (F1=0.59) and MIDAS 2 (F1=0.6), but was inferior to MetaPhlAn 2 (F1: 0.95). We also evaluated the LRTK performance of taxonomic quantification by comparing the benchmark microbial abundance (from simulation and ATCC-MSA-1003) and the predicted values using Spearman correlation coefficient (SCC). For the simulated dataset, LRTK (SCC=0.99), MIDAS 2 (SCC=0.98), and KMCP (SCC=0.95) exhibited superior performance. For stLFR linked-reads in ATCC-MSA-1003, LRTK (SCC=0.97), Bracken (SCC=0.97), and MetaPhlAn 2 (SCC=0.97) were the top performers. Comparable findings were also observed in the linked-reads data generated from other technologies in simulation **(Figure S3A)** and ATCC-MSA-1003 **(Figure S3B and S3C).** In addition, LRTK also enables the identification of

microbial SNVs and could reconstruct their potential haplotypes based on co-barcoded linked-reads. Comparing different SNV callers implemented in LRTK, we discovered that approximately 176,891 SNVs were jointly detected by FreeBayes [17], SAMtools [18] and inStrain [35] on stLFR linked-reads for ATCC-MSA-1003 **(Figure 3B).** These SNVs account around 76%, 80% and 21% of the total SNVs detected by FreeBayes and SAMtools and inStrain, respectively **(Figure 3B)**. More than 50% of SNVs from inStrain can not be detected by FreeBayes and SAMtools, suggesting inStrain is the most sensitive tool to detect microbial SNVs. We further applied LRTK to explore the taxonomic composition and genomic variants for each sample from a longitudinal linked-read metagenomic dataset **(D1, Table S2)** [3]. Using multiple related samples, LRTK identified a genome-wide mirrored allele imbalance **(Methods)** for species Alistipes finegoldii: the minor alleles of some SNVs at some timepoints became the major alleles at another timepoints, which may suggest intra-host strain evolution over time **(Figure 3C and D)**.

LRTK promotes metagenome assembly using linked-reads with high barcode specificity

~~*De novo* assembly approaches have reconstructed numerous novel microorganisms from metagenomic sequencing data. Linked-read sequencing enhances metagenome assembly performance, especially for low abundance species[28]. Using benchmarking metagenomic sequencing data (ATCC-MSA-1003), we compared the performance of three linked-read based assemblers: Athena[28],~~

~~Supernova[29] and Pangaea[30]. Among them, Pangaea achieved the best reconstructed genome fractions, NGA50, and NA50, for both stLFR and TELL-Seq sequencing data **(Figure 4A)**. With Pangaea, LRTK achieves NA50 values of 1.8 Mb and 1.2 Mb for stLFR and TELL-Seq sequencing data, respectively. On 10x Genomics sequencing data, Athena exhibited superior assembly performance, with a NGA50 of 245 Kb. We applied LRTK on a dataset of human gut microbiomes (D2)[30] for illustrative purposes. Two of the assembled contigs were circularized genomes that showed near perfect collinearity with the closest reference genomes **(Figure 4B)**. LRTK could also automatically clusters the contigs into bins after assembly. LRTK recovered 24 near-complete, 7 high quality and 52 medium quality bins, respectively **(Figure 4C-E)**. The superior assembly performance we have observed affirms the effectiveness of linked-read sequencing technologies in metagenome assembly. Interestingly, the assembled metagenomes, due to their high quality, have the potential to serve as reference genomes. This characteristic makes LRTK valuable to longitudinal studies relying on linked-read metagenomic sequencing data.~~

In our previous study, we have shown metagenome assembly on linked-reads could improve assembly length and the number of near-complete MAGs[28]. We compared the performance of two well-known short-read assemblers: MEGAHIT [36] and metaSPAdes [37], and three linked-read assemblers: Athena [38], CloudSPAdes [39] and Pangaea [28] using the linked-reads from ATCC-MSA-1003 and simulation **(Table S3).** Among them, LRTK (Pangaea module) achieves the highest NA50 values

for stLFR (NA50=372kb) and TELL-Seq (NA50=339kb) linked-reads, respectively **(Figure 4A)**. As Pangaea is not compatible with 10x Genomics linked-reads, Athena becomes the best tool on 10x Genomics in terms for NA50 (Athena:146 kb; cloudSPAdes: 45 kb; metaSPAdes: 17 kb and MEGAHIT:79 kb). We also examined the assembly quality of each species in ATCC-MSA-1003, and observed that Pangaea always obtained the highest NA50 and N50 values for stLFR and TELL-Seq linked-reads while the assembly length is comparable **(Figure S4A)**. For simulated linked-reads, Pangaea and Athena also show superior performances than the other metagenome assemblers (**Figure S4B and Figure S5**). We applied LRTK to a human gut metagenomic dataset (D2) [28] and found two contigs were circularized, which showed near perfect collinearity with the closest reference genomes **(Figure 4B)**. LRTK could also automatically perform contig binning using MetaBAT 2 [40] after metagenome assembly. In the D2 data set, LRTK recovers 24 near-complete, 7 high-quality and 52 medium-quality bins for D2 **(Methods, Figure 4C-E)**. The superior assembly performance we have observed affirms the efficacy of linked-read sequencing technologies on metagenome assembly.

LRTK provides best practices for human ~~genome~~genomic variant detection and phasing ~~using long-range information~~

~~Previous studies have shown that the high base accuracy and long DNA fragments arising from linked-read sequencing benefits phasing of human genomic variants[31]. Here, we benchmarked the computational tools for variant detection and~~

phasing tasks, and constructed a best practice to apply linked-reads on human genome studies. To ensure fair comparisons, LRTK used the modified version of the barcode-aware alignment tool, EMA[12], to map linked reads from different sequencing technologies to the human genome (GRCH38). We first benchmarked the commonly used tools FreeBayes[14], GATK[16] and SAMtools[15] to detect SNV and small insertions and deletions (INDEL). Among them, GATK achieved the best F1 score in detecting SNVs, followed by SAMtools and FreeBayes **(Figure 5A)**. For INDEL calling, all three tools showed similar precision levels of around 0.5 while GATK had a better recall rate **(Figure 5B)**. We compared the linked-read phasing tools, Whatshap[21] and HapCUT2[20], in combination with the aforementioned variant detection tools. We observed that HapCUT2 **(Figure 5C and D)** achieves longer phasing blocks and higher phased heterozygous SNV rate compared to WhatsHap[21]. Since most structural variation (SV) detection tools were developed to be used with 10x Genomics sequencing data and may therefore have uncertain performance loss when used with other sequencing technologies, we only benchmarked the performance of SV calling tools using 10x Genomics sequencing data. As illustrated in **Figure 5F and G**, Aquila had higher recall rates for 50 bp – 1 Kb deletions and insertions, while LinkedSV[18] performed better in detecting deletions longer than 1 Kb. We also applied LRTK on a family trio's genomic sequencing dataset (D3: NA24143, NA24149 and NA24385) to evaluate its actual performance. LRTK demonstrated excellent phasing performance and improved identity-by-descent (IBD) segments detection for pairwise samples **(Figure 5E)**.

LRTK provides flexible commands to process sequencing data

A primary advantage of LRTK is its flexible, user-defined settings for different tasks. Users have a choice to run each LRTK module independently and generate separate results for each module. For instance, the MKFQ function could be independently used to simulate sequencing reads from the stLFR platform. For some functions, LRTK provides multiple tools for users to choose from. For metagenome SNVs detection, LRTK offers the user a choice between SAMtools[15], FreeBayes[14], and inStrain[27]. To accommodate user-specific requirements on LRTK command, LRTK also allows users to set different parameters and save the results for further comparison.

LRTK provides automated analysis and user-friendly reports

Previous studies have shown that genomic variant detection [41] and phasing could benefit from the high base quality and long DNA fragments provided by linked-read[42]. Here, we benchmarked the computational tools for human genomic variant detection and phasing, and demonstrated a best practice guideline for human genome linked-read analysis using LRTK. We applied the modified version of EMA to align linked-reads from different sequencing technologies to the human reference genome (GRCh38). We first benchmarked the commonly used tools FreeBayes [20], GATK [22] and SAMtools [21] to detect SNVs and small INDELs. Among them, GATK (F1=0.90) achieves the best average F1 score across the three technologies in detecting SNVs, followed by SAMtools (F1=0.89) and FreeBayes (F1=0.87) **(Figure**

5A). For small INDEL calling, SAMtools demonstrated the best average F1 score (SAMtools: F1= 0.71; GATK: F1= 0.65; FreeBayes: F1 = 0.59) while GATK has a better recall value (SAMtools: average recall = 0.87; GATK: average recall = 0.91; FreeBayes: average recall = 0.79) (**Figure 5B)**. We then compared the linked-read phasing tools, HapCUT2 [26] and WhatsHap [27], for genomic variant phasing. We observed that HapCUT2 **(Figure 5C and D)** achieved longer average length of phasing blocks (HapCUT2: 23.2 Mb and WhatsHap: 0.4 Mb) and a higher average phased heterozygous SNV rate compared to WhatsHap (HapCUT2: 0.99 and WhatsHap: 0.63). We only evaluated the performance of SV detection tools on 10x Genomics linked-reads of NA24385 because some of the tools do not support stLFR and TELL-Seq. As the recall values illustrated in **Figure 5F**, Aquila has a higher recall value for 50 bps – 1 kb deletions (Aquila: 0.82; LinkedSV: 0.54; Long Ranger: 0.43). LinkedSV [24] and Long Ranger perform better in detecting deletions longer than 1 kb (Aquila: 0.29; LinkedSV: 0.71; Long Ranger: 0.72). Aquila also shows a better performance than Pamir [43] and PopIns2 [44] to detect insertions (Aquila: 0.35; Pamir: 0.07; PopIns2: 0.01) (**Figure 5G**). We also applied LRTK to linked-read from a trio (D3; Father: NA24149; Mother: NA24143; and Son:NA24385) and found it demonstrated excellent variant phasing performance and identity-by-descent segment detection in pairwise samples **(Figure 5E)**.

LRTK provides flexible commands to process sequencing data

A primary advantage of LRTK is its flexible, user-defined settings for different tasks. Users have a choice to run each LRTK module independently and generate separate results for each module. For instance, the MKFQ function could be independently used to simulate linked- reads from 10x Genomics and stLFR platforms. For some functions, LRTK provides multiple tools for users to choose. For microbial SNVs detection, LRTK offers users a choice between SAMtools [21], FreeBayes [20], and inStrain [35]. To accommodate user-specific requirements on LRTK command, LRTK also allows users to set different parameters and save the results for further comparison.

LRTK provides automated analysis and user-friendly reports

LRTK provides an automated analysis pipeline, starting from raw ~~sequencing~~ linked-reads to performing diverse data analysis and generating publication-ready visualizations. Specifically, LRTK ~~produces~~investigates different types of data features, calculates their corresponding statistical indicators, and presents them together in a HTML report. Taking the aforementioned longitudinal linked-read sequencing dataset D1 as an example, LRTK generates a systematic summary ~~for~~of the input ~~data~~sequencing libraries and analysis ~~results~~outcomes of each step. Firstly, LRTK produces summary ~~statistics for~~of the FASTQ files obtained from the read quality control ~~(QC)~~ tools **(Figure ~~S3A~~S6A-B)**. After aligning reads to the reference genomes, LRTK calculates the key parameters for ~~library preparation~~the sequencing

Formatted: Indent: First line: 0"

Formatted: Font color: Black

Formatted: Font color: Black

libraries and reconstructed long DNA fragments **(Figure ~~S3C~~S6C-D)**. For ~~detected~~microbial species and genomic variants, LRTK generates basic statistics and presents them using concise distribution plots **(Figure ~~S3E~~S6E-F)**. For downstream analysis, LRTK conducts principal components analysis ~~(PCA) analysis~~ on the relative abundance profiles from multiple samples and performs clustering analysis on allele ~~frequency to make comparisons across multiple samples~~ frequencies of microbial SNVs **(Figure ~~S3G~~S6G)**. Similar reports could be produced for ~~users employing LRTK to study the~~ human genome analysis using LRTK **(Figure ~~S4~~S7)**.

Evaluation of the computational resources required for LRTK

We would focus on evaluating computational resources required by LRTK for linked-read preprocessing and alignment. The computational resources needed for genomic variants calling and phasing depend on the chosen software, while metagenome sequencing data typically demand fewer computational resources due to the lower volume of required sequencing reads. We extracted approximately the same data volume (around 35X, 110G bases) of linked-reads from NA12878 for 10x Genomics, stLFR and TELL-Seq and aligned these reads to the human reference genomes. As shown in **Figure S8**, LRTK required around 26.3, 37.6 and 19.8 hours to align reads from the three platforms with a maximum memory usage of around 74Gb using 64 threads. In comparison to Long Range, LRTK reduced memory requirements (maximum memory for Long Ranger: >100 Gb) at the expense of increased running

Formatted: Font color: Black

Formatted: Font color: Black

Formatted: Font color: Black

Formatted: Font color: Black

Formatted: Font: Times New Roman

time. The experiments were carried out on the computational nodes with Xeon Gold 6330 @ 2.0GHz (2S/28C) / 1T RAM / 900GB SWAP.

Discussion

Seeing that multiple linked-read sequencing technologies are extensively utilized in scientific studies, a platform agnostic linked-read processing tool would be an intuitive solution to ensure reproducibility and robustness. Unfortunately, a cross-platform software solution is currently unavailable to the research community. Accordingly, we introduce LRTK, a unified and versatile computational framework to efficiently process sequencing data from 10x Genomics, stLFR and TELL-Seq technologies. LRTK includes separately invokable commands to perform linked-read simulation, barcode sequencing error correction, barcode-aware alignment~~, read cloud based~~ and metagenome assembly, reconstruction of long DNA fragments and other barcode-assisted genomic variant calling and phasing. LRTK also provides automated and complete analysis, from raw data QC through advanced downstream analysis to generation of publication-ready visualization. LRTK is also open source, allows easy integration with other scientific pipelines.

~~One of the core advantages of LRTK is that it provides solutions for both metagenomic analysis and human genome analysis. Linked-read sequencing technologies offer highly accurate reads and inferable long-range information, which confers distinct advantages in genome research, such as superior phasing ability[31]. Phasing is valuable in exploring intra-host evolution of microbiota where strains~~

under the same species may have some variants. LRTK follows a reference genome based approach to detect species' abundance and to identify metagenome variants. It also infers the mirrored allele imbalance from multiple related samples. In addition, we establish a best practice to perform variation calling, phasing, and detection of IBD segments for human genome.

Finally, it is worth mentioning that LRTK has the potential to be extended to handle other types of linked-read sequencing technologies. For example, in 2017, Illumina introduced the bead-based barcode partitioning in a single tube to phase human genomes. It further proposed the complete long-read technology for complex genomes in 2022[32]. Additionally, Meier J I, *et al.* developed Haplotype tagging to investigate the butterfly species[33]. Redin D, *et al.* recently introduced a novel library preparation method for high throughput barcoding of short reads[34]. The new single cell metagenomic sequencing technologies employs highly accurate barcoded reads and provides inferable long range information, which could potentially be used in combination with current linked-read technology in future studies[35]. We are actively developing LRTK to incorporate these technologies.

Short-read sequencing has led to significant discoveries in large-scale population sequencing studies within the human genome and metagenome fields [45,46]. However, the limited sequencing length poses challenges for tasks like complex structural variant detection in the human genome and ribosomal RNAs assembly from metagenomic data. Long-read sequencing, such as single-molecule real-time sequencing by Pacific Biosciences (PacBio), nanopore sequencing by Oxford

Nanopore Technologies (ONT), are gaining attentions for their improved performance in addressing these challenges. Despite their advantages, long-read sequencing can be costly for large-cohort studies. Linked-read sequencing technologies, offer a cost-effective solution for large population studies by attaching barcodes to short-reads to establish long-range DNA connectedness. Previous researches have shown 10x Genomics linked-reads have facilitated the discovery of complex structural variants, such as chromothripsis [5], large rearrangements [47] and tandem duplications [14], in cancer studies. Additionally, 10x Genomics linked-reads have enhanced metagenome assembly contiguity [38] and enabled haplotype construction from time-series metagenomic data [3]. The newly emerging stLFR and TELL-Seq have further improved barcode specificity aiming for one fragment per barcode. These advancements have shown superior performance in distinguishing linked-reads from different species [28]. Our study benchmarks tools developed for linked-read sequencing and integrates them into LRTK to support academic applications. Furthermore, LRTK is the only tool that can accept linked-reads from all three platforms and avoid loss of barcode specificity.

Our finding revealed that in practice, the $N_{F/P}$ values for stLFR and TELL-Seq were much lower than those obtained from 10x Genomics. According to Bishara, Alex, et al. [38], assembly using 10x Genomics linked-reads often struggles with high-copy genomic repeat regions. The long-fragment barcoding approaches adopted by stLFR and TELL-seq has notably reduced the number of long fragments per barcode. This reduction could improve the assembly of microbial genomes containing

high-copy repeats. In our previous study, we demonstrated that the characteristics of the reconstructed long DNA fragments (E.g. C_R, C_F et al.) can significantly affect human genome assembly [19] and structural variant calling [30]. The improved stLFR and TELL-Seq may also further refine the structural variants calling and variant phasing on human genome. We anticipate that future linked-read sequencing technologies will improve both DNA-extraction techniques and long-fragment barcoding approaches to achieve one fragment per barcode.

Finally, it is worth mentioning that LRTK has the potential to be extended to handle other types of linked-read sequencing technologies. For example, in 2017, Illumina introduced the bead-based barcode partitioning in a single tube to phase human genomes. It further proposed the complete long-read technology for complex genomes in 2022 [48]. Additionally, Meier J I, *et al.* developed Haplotype tagging to investigate the butterfly species [49]. Redin D, *et al.* recently introduced a novel library preparation method for high throughput barcoding of short reads [50]. The new single-cell metagenomic sequencing technologies employ highly accurate barcoded reads and provide inferable long-range information, which could potentially be used in combination with current linked-read technology in future studies [51]. We are actively developing LRTK to incorporate these technologies.

Methods

Data collection

~~We collected one benchmarking dataset and two real datasets to demonstrate the~~

performance of LRTK in processing metagenomic sequencing data **(Table S3)**. The benchmarking datasets (B1: ATCC-MSA-1003) were obtained from the NCBI with the following accession numbers: SRR12283286 for 10x Genomics, and PRJNA875547 for stLFR and TELL-Seq sequencing technologies. The ATCC-MSA-1003 mock community contains 20 bacteria species in staggered abundances (5 species at 18%, 1.8%, 0.18% and 0.02% abundance levels, respectively). The detailed composition of each bacterial species as well as the corresponding reference sequence could be acquired from the website: https://www.atcc.org/products/msa-1003. The first real metagenomic dataset (D1), consisting of longitudinal 10x Genomics linked-read sequencing data, was downloaded from the NCBI under accession number SRP323279. We used only a subset of samples containing the barcode information in the read ID **(Table S3)**. Another real metagenomic dataset (D2) containing deep stLFR sequencing data, was downloaded from the China National GeneBank (CNGB) under project CNP0003432. For the human genome section, we obtained the linked-read of NA12878 for 10x Genomics, stLFR and TELL-Seq and used them as the benchmarking dataset B2. We also downloaded the 10x Genomics linked-read sequencing data for a family trio (NA24143, NA24149 and NA24385) and used them as the real data set D3 **(Table S3)**.

We included one simulated metagenomic dataset (S1), one mock microbial community (B1) and two human gut metagenomic sequencing datasets (D1, D2) to evaluate the performance of the metagenomic data analysis section of LRTK **(Table**

S2). For S1, we simulated 11.8 Gb 10x Genomics and 9.2 Gb stLFR linked-reads using LRTK-SIM [29] for 40 complete bacterial genomes extracted from the NCBI RefSeq database (December, 2023) with the same abundances (Log Normal Distribution) **(Table S5)**. LRTK-SIM allows flexible parameters, such as C_F, C_R, $N_{F/P}$, μ_{FL} and $W\mu_{FL}$, to simulate linked-read data. We set the parameters, "C_F =500, C_R =0.2, $N_{F/P}$ =16, μ_{FL} =20" to simulate 10x Genomics linked-reads while "C_F =500, C_R =0.2, $N_{F/P}$ =1, μ_{FL} =20" to simulate stLFR linked-reads. The dataset B1 contains linked-reads from a mock microbial community ATCC-MSA-1003, from three different platforms (SRR12283286 for 10x Genomics, and PRJNA875547 for stLFR and TELL-Seq). The ATCC-MSA-1003 mock community is composed of 20 bacterial species represented at staggered abundances—specifically, five species at 18%, 1.8%, 0.18%, and 0.02% abundance levels, respectively. The complete descriptions of the mock metagenomic sample, including the genome sizes, individual bacterial species, and their corresponding reference sequence accessions, have been included in **Table S4.** The D1, contains 16 longitudinal human gut metagenomic 10x Genomics linked-read sequencing datasets from one single individual (accession number: SRP323279) **(Table S2)**. The D2 was downloaded from the China National GeneBank (CNGB) under project CNP0003432. It contains around 99 Gb stLFR human gut metagenomic sequencing data. In the human genome section, we collected the available linked-read sequencing data for the three technologies from NA12878, NA24143, NA24149 and NA24385. The detailed information has been included in

Table S2. For ATCC-MSA-1003 and NA12878, we further performed linked-read down-sampling at the barcode level by using in-house scripts.

Data preprocessing

We utilized LRTK to convert the raw linked-read from 10x Genomics, stLFR and TELL-Seq into a unified FASTQ format **(Figure S1)** and correct potential sequencing errors in barcodes. For the 10x Genomics and stLFR linked-read, the barcodes are aligned to their respective barcode whitelists using the BWA aln command. Aligned barcodes containing fewer than 2 mismatches are then corrected as the corresponding barcodes in the whitelist. Due to the lack of a barcode whitelist for TELL-Seq, LRTK adopts the approach described by Chen *et al.*[3] to correct barcode errors. LRTK counts the supporting read for each barcode, compares barcodes with one supporting read to those with multiple supporting reads, and corrects potential sequencing errors for barcodes with one mismatch to those with multiple supporting reads. The linked-read in the unified FASTQ file are then subjected to fastp[36] to remove adapter sequences and low-quality reads. For metagenomic sequencing data, the sequencing reads are additionally aligned to the human genome and only unmapped microbial reads are retained. LRTK also provides support for simulating linked-read from 10x Genomics and stLFR platforms through modification of LRTK-SIM[23].

LRTK converts the raw linked-reads from 10x Genomics, stLFR and TELL-Seq into a unified FASTQ format **(Figure S1)** and corrects potential sequencing errors in barcodes. For 10x Genomics and stLFR linked-read, the barcodes are aligned to their

respective barcode whitelists using the “BWA aln” command. The barcodes with fewer than 2 mismatches in alignments are then corrected as the corresponding barcodes in the whitelist. LRTK adopts the approach described by Chen *et al.* [7] to correct barcode errors for TELL-Seq due to the lack of barcode whitelist. In general, LRTK tallies the supporting reads for each barcode derived from TELL-Seq linked-reads and distinguishes between barcodes with a single supporting read and those with multiple supporting reads. It then corrects possible sequencing errors in barcodes that initially had one mismatch by comparing them to those with multiple supporting reads. The linked-reads in the unified FASTQ file are then provided to fastp [52] to remove adapter sequences and low-quality reads. For metagenomic sequencing data, the sequencing reads are aligned to the human genome first and only unmapped reads are used for subsequent analysis.

Metagenome assembly and contig binning

~~We evaluated the performance of three different metagenome assemblers: Athena[28], Pangaea[30] and Supernova[29], and observed superior performance of Pangaea on stLFR and TELL-Seq sequencing data. Therefore, for LRTK, we chose Pangaea as the default assembler to assemble linked metagenomic sequencing data. After the initial assembly, we extracted the contigs that were at least 1Mb in length and checked their circularization. The uncircularized contigs were grouped using MetaBAT2[37].~~

We evaluated the performance of five metagenome assemblers on linked-reads: Athena [38], Pangaea [28], cloudSPAdes [39], MEGAHIT [36] and metaSPAdes [37], and observed superior performance of Pangaea on stLFR and TELL-Seq sequencing data. Therefore, for LRTK, we chose Pangaea as the default assembler to assemble linked-reads metagenomic sequencing data for LRTK. After the initial assembly, LRTK extracts the circular contigs that are at least 1Mb in length. The uncircularized contigs are grouped into MAGs using MetaBAT 2 [40]. According to standard criteria of the minimum information about MAGs [53], they could be classified into near-complete (completeness > 90%, contamination < 5%, and could be detected 5S, 16S, and 23S rRNAs, and at least 18 tRNAs), high-quality (completeness > 90%, and contamination < 5%), medium-quality (completeness ≥ 50%, and contamination < 10%), and low-quality (the other MAGs).

Barcode-aware read alignment

~~For human genome sequencing data, we utilized EMA, a barcode-aware alignment approach, to map high-quality reads to the human reference genome[38]. We modified EMA to be compatible with the barcodes from stLFR (30 bp) and TELL-Seq (18 bp). LRTK marks PCR duplicates for each barcode using the "~~For human genome sequencing data, LRTK utilizes EMA, a barcode-aware alignment approach, to map high-quality 10x Genomics linked-reads to the human reference genome [54]. We further modified EMA to be compatible with the barcodes from stLFR (30 bps) and TELL-Seq (18 bps). LRTK marks PCR duplicates for each barcode using the

Formatted: Indent: First line: 0"

"BARCODE_TAG" parameter in Picard (https://broadinstitute.github.io/picard/). The alignment files are then sorted according to the genomic coordinates of alignments for further analysis.

~~For metagenomic sequencing data, we developed a tiered alignment approach to align the linked read to microbial genome using modified EMA. The default reference genomes for the human gut metagenome were downloaded from UHGG[11]. After the first alignment, we partitioned the reference genomes into 1-kb windows and calculated the number of mapped reads for each window. The mapped reads are categorized into two types: unique mapped reads (U) and reads with multiple alignments (M). Thus, we can use the following formulas to determine the total read count of the window (RC(W)), unique mapped read count ($RC(U)$) and multiply mapped read count ($RC(M)$), where l is the window size.~~

For metagenomic sequencing data, we developed a tiered alignment approach to align the linked-reads to microbial genomes using the aforementioned modified EMA. The default reference genomes for the human gut metagenome were downloaded from UHGG [17]. For non-gut samples, we used the GTDB [55] as the reference database, but users could also incorporate their own custom database.

In the first round of alignment, linked-reads were directly mapped to the 4,724 representative genomes in UHGG (by default). For each genome, LRTK calculates the coverage rate and covered base, and then extracts genomes with "coverage rate > 40% and covered base > 500 kb". These genomes will be used as the candidate reference genomes for the second round of alignment.

In the second round of alignment, linked reads are only mapped to those candidate reference genomes to reduce multiple alignment errors. For each candidate genome, LRTK partitions it into 10 kb windows and calculated the number of mapped reads on each window. The mapped reads are categorized into two types: unique-mapped reads (U) and multi-mapped reads (M), which will be processed separately. LRTK uses the formulas below to determine the total read count of a window (RC(W)), unique-mapped read count ($RC(U)$) and multi-mapped read count ($RC(M)$), where l is the window size.

$$RC(W) = RC(U) + RC(M)$$

$$RC(U) = \frac{U}{l}$$

$$RC(M) = \sum_{i=1}^{M} \omega_i * \{M\}/l$$

~~In the case of read with multiple alignments, a~~A coefficient ω is introduced~~.~~ for multi-mapped reads. When a ~~read~~multi-mapped in M ~~has alignments with~~ is aligned to N different ~~windows~~genomes, ω is calculated using the following formula:

$$\omega = U / \sum_{i=1}^{N} RC(U)$$

~~To ensure accurate estimation~~LRTK removes the windows with an extreme number of ~~genome abundance, we calculate it~~ reads (> <), and calculates the average depth using the remaining windows as the ~~mean~~depth of the ~~n% most closely covered~~

windows. After initial classifyingcorresponding genome. The relative abundance is then calculated by aggregating the average depth of microbialall identified genomes, we extract.

In the experiments, we further evaluated the performance (F1 score, precision and recall) of LRTK, KMCP [32], and MIDAS 2 [34] to identify microbes and quantify their respectiveabundances. The reference databases were prepared using the genome sequences and realign the reads to the new subset reference genome sequence Finally, we recalculate the relative abundance by using the most closely covered windows againfrom the GTDB database for the three tools. We also compared LRTK with the widely used taxonomic classification tools: Bracken [31] and MetaPhlAn 2 [33] using their default databases.

Reconstruction of long DNA fragments

We developed a computational strategy to reconstruct long DNA fragments from linked-read sequencing data. Initially LRTK extracts unique-mapped co-barcoded paired--end short-reads that share the same barcode infrom the alignment file. The uniquely aligned paired-end reads are collected to evaluate the distribution of insert sizes (with a mean of μ_{PE} and a standard deviation of σ_{PE}). Alignments are removed if the distance between two reads in a pairpaired-end read (R1 and R2) exceedsexceed a certain threshold, that is,

$$Dis(\mathrm{R1}, \mathrm{R2}) > \mu_{PE} + 3 * \sigma_{PE}.$$

Formatted: Indent: First line: 0"

Formatted: Font color: Black

Formatted: Font color: Black

Formatted: Font color: Black

Formatted: Font color: Black

The remaining paired-end reads are used as seeds and extended to both directions to connect with other seeds sharing the same barcode until no more eligible seeds can be found within a specified distance. (200 kb by default). All of these co-barcoded reads are considered to be derived from the same long DNA fragment and are collected as a read cloud.

Identify and phase genomic variants

LRTK supports multiple popular variant detection tools and adheres to best practices for linked-read sequencing. For human genomic sequencing data, LRTK provides FreeBayes[14], SAMtools[15] and GATK[16] to call SNVs and INDELs. Freebayes is a Bayesian genetic variant caller designed to find SNPs, indels, multinucleotide polymorphisms, and complex events. It is straightforward, easy to use and timesaving. Similar to Freebaye, GATRK also mainly used the Bayesian model to detect variants. however, GATK enhance the ability to identify small insertions and deletions by adopting some distinct methods, such as read realignment, base recalibration. These steps are useful but also largely increased the running time of GATK. By contrast, SAMTOOLS utilized the HMM model to rapidly detect small variants and performs well in many studies. The available phasing tools include HapCUT2[20] and WhatsHap[21]. Having obtained the phased SNVs, we then use hap-ibd[39] to detect pairwise identity-by-descent segments across multiple samples. By default, large structural

variations (SVs) are identified and phased using Aquila[17]. However, Users have the flexibility to select other tools such as LinkedSV[18] and VALOR2[19] for SV analysis. LinkedSV leverages barcode overlapping, read depth, paired-end signals and local assembly to detect deletions. However, current LinkedSV does not support the detection of insertions. By contrast, the assembly-based tool, Aquila, could detect insertions and deletions at the same time and may be an alternative solution.

For metagenome sequencing data, LRTK offers three metagenome SNV callers: FreeBayes[14], inStrain[27] and SAMtools[15]. The SNV phasing is performed on high abundance species using WhatsHap[21] with the inferred ploidy. Because HapCUT2 mainly works on diploid model (human genome) while WhatsHap adopts a novel clustering and threading method to perform accurate polyploid phasing, which may be effective for microbial variants. LRTK also selects SNVs located on certain high abundance species and compares the SNV profiles across multiple samples. Based on the allele frequency of SNVs, we used an unsupervised clustering method to detect potential minor allele imbalance (MAI) events for pairwise samples. In brief, one sample is chosen as the reference for all the contigs. The minor allele frequency of heterozygous SNVs present in others samples are then compared to the reference sample. We used the k-means clustering method to separate the SNVs into distinct groups using the minor allele frequency matrix. The number of clusters were determined by using the Calinski-Harabasz index. The number of clusters may be used to determine the number of strains under the same species.

Identify and phase genomic variants

LRTK supports multiple popular variant detection tools and adheres to best practices for linked-read sequencing. For human genome sequencing data, LRTK provides FreeBayes [20], SAMtools [21] and GATK [22] to call SNVs and small INDELs. FreeBayes is a Bayesian genetic variant detection tool designed to identify SNPs, INDELs, multinucleotide polymorphisms, and more complex events. It is straightforward, easy to use and timesaving. GATK, while also employing a Bayesian framework, enhances its detection capabilities for insertions and deletions through specialized techniques such as read realignment and base recalibration. Although these steps add value, they also increase GATK's computational runtime. In contrast, SAMtools uses a hidden Markov model for the identification of small variants and has demonstrated robust performance across various studies. The available phasing tools include HapCUT2 [26] and WhatsHap [27]. HapCUT2 demonstrated excellent performance to phase heterozygous SNVs within a diploid context, such as the human genome. WhatsHap, however, introduces an innovative clustering and threading approach that delivers precise phasing in polyploid genomes. Having obtained the phased SNVs, we used PhaseME [56] to calculate the phasing block and phasing rate to assess the phasing quality. Subsequently, we used hap-ibd [57] to detect pairwise identity-by-descent segments across multiple samples. By default, large SVs were identified using Aquila[23]. However, Users have the flexibility to select other tools such as LinkedSV [24] and VALOR2 [25] for SV analysis. LinkedSV leverages barcode overlapping, read depth, paired-end signals and local assembly to detect

deletions, although it currently lacks support for insertion detection. In contrast, the assembly-based tool Aquila is capable of detecting both insertions and deletions, offering a comprehensive solution.

For metagenome sequencing data, LRTK offers three metagenome SNV callers: FreeBayes [20], inStrain [35] and SAMtools [21]. The SNV phasing was performed on high abundance species using WhatsHap [27] with the inferred ploidy. LRTK also selected SNVs located on certain high abundance species and compared the SNV profiles across multiple samples. Based on the allele frequency of SNVs, we used an unsupervised clustering method to detect potential mirrored allele imbalance (MAI) events for pairwise samples. MAI is derived from the notion of mirrored subclonal allelic imbalance observed in cancer genomes, where the maternal allele is gained or lost in one tumor region and the paternal allele is gained or lost in a different region [58]. In metagenome, MAI means that the reference allele is gained or lost at one time point while the altered allele (minor) allele is gained or lost at a different time point. To detect MAI, one sample was chosen as the reference for all the contigs. For each species which was present in at least two sample, the minor allele was determined based on the reference sample. The BAF was calculated as the ratio of the minor allele count to the total allele count. For each interested species, BAFs of SNPs (minimum supported read number exceeding 2) were extracted from each sample and merged into a combined SNP set. Based on the combined SNP set, BAF values for the interested sample were then compared with the BAFs of the reference sample. We used the k-means clustering method to separate the SNVs into distinct groups using

the minor allele frequency matrix. The number of clusters were determined by using the Calinski-Harabasz index.

Downstream analysis and HTML-based visualization

The human genome analysis report begins with generating the FASTQ quality control statistics during the preprocessing step. ~~For the~~The barcode~~ ~~-aware alignment step~~, it~~ presents the distribution of several key statistics about the DNA sequencing library, including the number of fragments per barcode, fragment length and average read coverage per fragment. It also includes information about the sequencing coverage and insert size. The variant calling step summarizes the number of SNVs, small INDELs and large SVs and illustrates their distributions.

Similarly, the metagenome analysis report~~ firstly~~ illustrates the QC results for preprocessing, alignment and variant calling steps. Additionally, it includes an optional report about the automatic analysis of multiple related samples. The report shows the distribution of high abundant species across multiple samples, and uses a principal component analysis ~~(PCA) ~~plot to visualize the divergence across them. The report also depicts the distribution of SNVs of these high abundant species, and the minor allele frequency distribution in pairwise samples.

Formatted: Indent: First line: 2 ch

Code Availability and Requirements

Source code is available at https://github.com/ericcombiolab/LRTK. LRTK can be downloaded as a packaged conda environment (https://anaconda.org/bioconda/lrtk).

Project name: Linked Read ToolKits project

Project home page: https://github.com/ericcombiolab/LRTK

Operating system(s): Linux and macOS

Programming language: C and ~~python~~Python

Other requirements: Conda, Python 3.6 or higher

License: MIT

RRID: SCR_023945

Data Availability

~~We curated two benchmarking datasets and three real datasets to evaluate the performance of LRTK in processing linked-read sequencing data.~~

We included one simulated metagenomic dataset (S1), one mock microbial community (B1) and two human gut metagenomic sequencing datasets (D1, D2) in the metagenome section and the human genome sequencing data for four samples (D3) in the human genome section **(Table S2).**

B1: The ~~benchmarking dataset~~mock microbial community B1 contains the 10x Genomics, stLFR and TELL-Seq sequencing data for ATCC MSA-1003 mock community. These datasets were obtained from the NCBI with the following accession numbers: SRR12283286 for 10x Genomics, and PRJNA875547 for stLFR and TELL-Seq sequencing technologies.

~~**B2:** The benchmarking dataset B2 contains the 10x Genomics, stLFR and TELL-Seq sequencing data for human sample NA12878. The linked-read of for 10x Genomics and stLFR were downloaded from GIAB while the raw TELL-Seq data was downloaded with the accession number SRR10584152 (PRJNA591637).~~

D1: The first real metagenomic dataset (D1), consisting of longitudinal 10x Genomics linked-read sequencing data, was downloaded from the NCBI under accession number SRP323279. ~~We used only a subset of samples containing the barcode information in the read ID.~~

D2: The second real metagenomic dataset (D2), containing deep stLFR sequencing data, was downloaded from the China National GeneBank (CNGB) under project CNP0003432.~~-~~

D3: ~~We also downloaded~~The human genome dataset D3 contains the linked-read sequencing data for ~~a family trio (~~NA12878, NA24143, NA24149 and NA24385~~) and used them as the real data set D3 (Table S3).~~. The 10x Genomics and stLFR sequencing data was downloaded ~~from GIAB.~~following the links in **Table S2**. For TELL-Seq sequencing data, we only obtained the raw TELL-Seq data from NA12878 and NA24385 from SRA database ~~(PRJNA591637: including SRR10689414,~~

Formatted: Font color: Black

Formatted: Font color: Black

Formatted: Font: Not Bold

Formatted: Font color: Black

Formatted: Normal (Web)

Formatted: Font: Not Bold, Font color: Black

Formatted: Font: SimSun, Not Bold

Formatted: Font color: Black

Formatted: Font color: Black

Formatted: Font color: Black

Formatted: Font color: Black

Formatted: Font color: Black

~~SRR10689415, SRR10689416, SRR10689417).~~under the accession SRX7264479 and SRX7264481, respectively.

Abbreviations

stLFR: single-tube long fragment read

TELL-Seq: transposase enzyme linked long-read sequencing

SNV: single nucleotide variant

INDEL: small insertion and deletion

SV: structural variation

CRediT authorship contribution statement:

Chao Yang: Writing - original draft, and preparing figures and tables, preparing source codes; **Zhenmiao Zhang:** preparing source codes; **Xuefeng Xie:** Consolidating resources; **Yufen Huang:** Consolidating resources; **Herui Liao:** preparing source codes; **Werner P Veldsman:** Revising - original draft, and visualizations; **Jin Xiao:** Revising - original draft, and visualizations; **Kejing Yin:** Supervision; **Xiaodong Fang:** Supervision; **Lu Zhang:** Project administer, Writing – review & editing, Supervision, and funding acquisition.

Declaration of Competing Interest:

The authors declare that they have no known competing financial interests

Acknowledgments:

This research was partially supported by the open project of BGI-Shenzhen, Shenzhen 518000, China (BGIRSZ20220012), the Hong Kong Research Grant Council Early Career Scheme (HKBU 22201419), HKBU Start-up Grant Tier 2 (RC-SGT2/19-20/SCI/007), HKBU IRCMS (No. IRCMS/19-20/D02), the Guangdong Basic and Applied Basic Research Foundation (No. 2021A1515012226), the Science Technology and Innovation Committee of Shenzhen Municipality, China (SGDX20190919142801722) and Shenzhen Science and Technology Innovation Commission (SZSTI) - Shenzhen Virtual University Park (SZVUP) Special Fund Project (No. 2021Szvup135).

Additional Files

Figure S1. LRTK text file specification.

Figure S2. Ideogrammatic definitions of C_R, C_F, $N_{F/P}$, μ_{FL}, and $W\mu_{FL}$ metrics.

Figure S3. Demo reports for metagenomics sequencingEvaluation of taxonomic quantification performance for 10x Genomics and TELL-Seq linked-reads.

Figure S4. Evaluation of assembly performance at species level.

Figure S5. Evaluation of metagenomic assemblers on simulated linked-read data.

Figure S6. Demo reports for metagenomics sequencing.

Figure S7. Demo reports for human genome sequencing.

Figure S8. Computational requirements for linked-reads preprocessing and alignment between LRTK and Long Ranger on NA12878.

Table S1. ~~Comparison of LRTK with other published linked-read pipelines.~~

~~Table S2.~~ Bioinformatics tools included in LRTK.

Table ~~S3~~S2. Linked-read sequencing data sources.

Table S3. Data descriptions for the simulated and down-sampled linked-reads from ATCC-MSA-1003 and NA12878.

Table S4. Reference genomes for the ATCC-MSA-1003 mock sample.

Table S5. Reference genomes for the simulated dataset.

Formatted: Line spacing: single

Formatted: Font: 16 pt

References

~~1. Eisenstein M. Startups use short-read data to expand long-read sequencing market. *Nat Biotechnol*. Nat Biotechnol; 2015; doi: 10.1038/NBT0515-433.~~

~~2~~1. Bergström A, McCarthy SA, Hui R, Almarri MA, Ayub Q, Danecek P, et al.. Insights into human genetic variation and population history from 929 diverse genomes. *Science (80-)*. American Association for the Advancement of Science; 2020; doi: 10.1126/SCIENCE.AAY5012/SUPPL_FILE/AAY5012-BERGSTROM-SM.PDF.

2. Dréau A, Venu V, Avdievich E, Gaspar L, Jones FC. Genome-wide recombination map construction from single individuals using linked-read sequencing. *Nat Commun 2019 101*. Nature Publishing Group; 2019; doi: 10.1038/s41467-019-12210-9.

3. Roodgar M, Good BH, Garud NR, Martis S, Avula M, Zhou W, et al.. Longitudinal linked-read sequencing reveals ecological and evolutionary responses of a human gut microbiome during antibiotic treatment. *Genome Res*. Cold Spring Harbor Laboratory Press; 2021; doi: 10.1101/GR.265058.120.

4. Hadi K, Yao X, Behr JM, Deshpande A, Xanthopoulakis C, Tian H, et al.. Distinct Classes of Complex Structural Variation Uncovered across Thousands of Cancer Genome Graphs. *Cell*. Cell Press; 2020; doi: 10.1016/J.CELL.2020.08.006.

5. Spies N, Weng Z, Bishara A, McDaniel J, Catoe D, Zook JM, et al.. Genome-wide reconstruction of complex structural variants using read clouds. *Nat Methods 2017 149*. Nature Publishing Group; 2017; doi: 10.1038/nmeth.4366.

6. Wang O, Chin R, Cheng X, Yan Wu MK, Mao Q, Tang J, et al.. Efficient and unique co-barcoding of second-generation sequencing reads from long DNA molecules enabling cost effective and accurate sequencing, haplotyping, and de novo assembly. *Genome Res*. Cold Spring Harbor Laboratory Press; 2019; doi: 10.1101/GR.245126.118.

~~3~~7. Chen Z, Pham L, Wu TC, Mo G, Xia Y, Chan PL, et al.. Ultralow-input single-tube linked-read library method enables short-read second-generation sequencing systems to routinely generate highly accurate and economical long-range sequencing information. *Genome Res*. Cold Spring Harbor Laboratory Press; 2020; doi: 10.1101/gr.260380.119.

~~4~~8~~. Spies N, Weng Z, Bishara A, McDaniel J, Catoe D, Zook JM, et al.. Genome-wide reconstruction of complex structural variants using read clouds. *Nat Methods 2017 149*. Nature Publishing Group; 2017; doi: 10.1038/nmeth.4366.~~

~~5~~. Siranosian BA, Brooks EF, Andermann T, Rezvani AR, Banaei N, Tang H, et al.. Rare transmission of commensal and pathogenic bacteria in the gut microbiome of hospitalized adults. *Nat Commun 2022 131*. Nature Publishing Group; 2022; doi: 10.1038/s41467-022-28048-7.

~~6~~9~~. Roodgar M, Good BH, Garud NR, Martis S, Avula M, Zhou W, et al.. Longitudinal linked-read sequencing reveals ecological and evolutionary responses of a human gut microbiome during antibiotic treatment. *Genome Res*. Cold Spring Harbor Laboratory Press; 2021; doi: 10.1101/GR.265058.120/-/DC1.~~

~~7~~. Huang Y, Jiang P, Liang Z, Chen R, Yue Z, Xie X, et al.. Assembly and analytical validation of a metagenomic reference catalog of human gut microbiota based on co-barcoding sequencing. *Front Microbiol*. Frontiers; 2023; doi: 10.3389/FMICB.2023.1145315.

~~8~~10. Davila Aleman FD. Microbiome and aging: A study of microbial evolution and community structure across model organisms. Abtract and Metadata. 2022;

~~9~~11. Tracanna V, Ossowicki A, Petrus MLC, Overduin S, Terlouw BR, Lund G, et al.. Dissecting Disease-Suppressive Rhizosphere Microbiomes by Functional Amplicon Sequencing and 10× Metagenomics. *mSystems*. American Society for Microbiology; 2021; doi: 10.1128/MSYSTEMS.01116-20/SUPPL_FILE/MSYSTEMS.0116-20-S0001.PDF.

~~10.~~12. Tolstoganov I, Pevzner PA, Korobeynikov A. SpLitteR: Diploid genome assembly using linked TELL-Seq reads and assembly graphs. *bioRxiv*. Cold Spring Harbor Laboratory; 2022; doi: 10.1101/2022.12.08.519233.

13. Marks P, Garcia S, Barrio AM, Belhocine K, Bernate J, Bharadwaj R, et al.. Resolving the full spectrum of human genome variation using Linked-Reads. *Genome Res*. 2019; doi: 10.1101/gr.234443.118.

14. Viswanathan SR, Ha G, Hoff AM, Wala JA, Carrot-Zhang J, Whelan CW, et al.. Structural Alterations Driving Castration-Resistant Prostate Cancer Revealed by Linked-Read Genome Sequencing. *Cell*. Cell Press; 2018; doi: 10.1016/J.CELL.2018.05.036.

15. Greer SU, Nadauld LD, Lau BT, Chen J, Wood-Bouwens C, Ford JM, et al.. Linked read sequencing resolves complex genomic rearrangements in gastric cancer metastases. *Genome Med*. Genome Medicine; 2017; doi: 10.1186/s13073-017-0447-8.

16. Zheng GXY, Lau BT, Schnall-Levin M, Jarosz M, Bell JM, Hindson CM, et al.. Haplotyping germline and cancer genomes with high-throughput linked-read sequencing. *Nat Biotechnol 2016 343*. Nature Publishing Group; 2016; doi: 10.1038/nbt.3432.

~~11~~17. A A, S N, M B, F S, M B, ZJ S, et al.. A unified catalog of 204,938 reference genomes from the human gut microbiome. *Nat Biotechnol*. Nat Biotechnol; 2021; doi: 10.1038/S41587-020-0603-3.

~~12~~18. Shajii A, Numanagić I, Whelan C, Berger B. Statistical Binning for Barcoded Reads Improves Downstream Analyses. *Cell Syst*. Cell Press; 2018; doi: 10.1016/J.CELS.2018.07.005/ATTACHMENT/8C829484-1F22-47F4-A513-B90845BD41D7/MMC1.PDF.

~~13~~19. Zhang L, Zhou X, Weng Z, Sidow A. Assessment of human diploid genome assembly with 10x Linked-Reads data. *Gigascience*. Gigascience; 2019; doi: 10.1093/GIGASCIENCE/GIZ141.

~~14~~20. Garrison E, Marth G. Haplotype-based variant detection from short-read sequencing. 2012; doi: 10.48550/arxiv.1207.3907.

~~15~~21. Li H, Handsaker B, Wysoker A, Fennell T, Ruan J, Homer N, et al.. The Sequence Alignment/Map format and SAMtools. *Bioinformatics*. Oxford Academic; 2009; doi: 10.1093/BIOINFORMATICS/BTP352.

1622. McKenna A, Hanna M, Banks E, Sivachenko A, Cibulskis K, Kernytsky A, et al.. The Genome Analysis Toolkit: A MapReduce framework for analyzing next-generation DNA sequencing data. *Genome Res*. Cold Spring Harbor Laboratory Press; 2010; doi: 10.1101/GR.107524.110.

1723. Zhou X, Zhang L, Weng Z, Dill DL, Sidow A. Aquila enables reference-assisted diploid personal genome assembly and comprehensive variant detection based on linked reads. *Nat Commun 2021 121*. Nature Publishing Group; 2021; doi: 10.1038/s41467-021-21395-x.

1824. Fang L, Kao C, Gonzalez M V., Mafra FA, Pellegrino da Silva R, Li M, et al.. LinkedSV for detection of mosaic structural variants from linked-read exome and genome sequencing data. *Nat Commun 2019 101*. Nature Publishing Group; 2019; doi: 10.1038/s41467-019-13397-7.

1925. Karaoğlanoğlu F, Ricketts C, Ebren E, Rasekh ME, Hajirasouliha I, Alkan C. VALOR2: characterization of large-scale structural variants using linked-reads. *Genome Biol*. BioMed Central Ltd.; 2020; doi: 10.1186/S13059-020-01975-8/TABLES/2.

2026. Edge P, Bafna V, Bansal V. HapCUT2: robust and accurate haplotype assembly for diverse sequencing technologies. *Genome Res*. Genome Res; 2017; doi: 10.1101/GR.213462.116.

2127. Patterson MD, Marschall T, Pisanti N, Van Iersel L, Stougie L, Klau GW, et al.. WhatsHap: Weighted Haplotype Assembly for Future-Generation Sequencing Reads.

https://home.liebertpub.com/cmb. Mary Ann Liebert, Inc. 140 Huguenot Street, 3rd Floor New Rochelle, NY 10801 USA ; 2015; doi: 10.1089/CMB.2014.0157.

~~22. Li H, Durbin R. Fast and accurate short read alignment with Burrows–Wheeler transform. *Bioinformatics*. Oxford Academic; 2009; doi: 10.1093/BIOINFORMATICS/BTP324.~~

~~23~~28. Zhang L, Kong Baptist H, Zhang Z, Kong H, Xiao J, Wang H, et al.. Exploring high-quality microbial genomes by assembling short-reads with long-range connectivity. 2023; doi: 10.21203/RS.3.RS-3280231/V1.

29. Zhang L, Fang X, Liao H, Zhang Z, Zhou X, Han L, et al.. A comprehensive investigation of metagenome assembly by linked-read sequencing. *Microbiome*. BioMed Central Ltd; 2020; doi: 10.1186/S40168-020-00929-3/FIGURES/4.

~~24. DH P, M C, C R, AJ M, PA C, P H. GTDB: an ongoing census of bacterial and archaeal diversity through a phylogenetically consistent, rank normalized and complete genome-based taxonomy. *Nucleic Acids Res*. Nucleic Acids Res; 2021; doi: 10.1093/NAR/GKAB776.~~

~~25~~30. Zhang L, Zhou X, Weng Z, Sidow A. De novo diploid genome assembly for genome-wide structural variant detection. *NAR Genomics Bioinforma*. Oxford Academic; 2020; doi: 10.1093/NARGAB/LQZ018.

31. Lu J, Breitwieser FP, Thielen P, Salzberg SL. Bracken: estimating species abundance in metagenomics data. *PeerJ Comput Sci*. PeerJ Inc.; 2017; doi: 10.7717/PEERJ-CS.104.

32. Shen W, Xiang H, Huang T, Tang H, Peng M, Cai D, et al.. KMCP: accurate metagenomic profiling of both prokaryotic and viral populations by pseudo-mapping. *Bioinformatics*. Oxford Academic; 2023; doi: 10.1093/BIOINFORMATICS/BTAC845.

~~26~~33. Segata N, Waldron L, Ballarini A, Narasimhan V, Jousson O, Huttenhower C. Metagenomic microbial community profiling using unique clade-specific marker genes. *Nat Methods*. 2012; doi: 10.1038/nmeth.2066.

34. Zhao C, Dimitrov B, Goldman M, Nayfach S, Pollard KS. MIDAS2: Metagenomic Intra-species Diversity Analysis System. *Bioinformatics*. Oxford Academic; 2023; doi: 10.1093/BIOINFORMATICS/BTAC713.

~~27~~35. Olm MR, Crits-Christoph A, Bouma-Gregson K, Firek BA, Morowitz MJ, Banfield JF. inStrain profiles population microdiversity from metagenomic data and sensitively detects shared microbial strains. *Nat Biotechnol 2021 396*. Nature Publishing Group; 2021; doi: 10.1038/s41587-020-00797-0.

~~28~~36. D L, CM L, R L, K S, TW L. MEGAHIT: an ultra-fast single-node solution for large and complex metagenomics assembly via succinct de Bruijn graph. *Bioinformatics*. Bioinformatics; 2015; doi: 10.1093/BIOINFORMATICS/BTV033.

37. Nurk S, Meleshko D, Korobeynikov A, Pevzner PA. metaSPAdes: a new versatile metagenomic assembler. *Genome Res*. Cold Spring Harbor Laboratory Press; 2017; doi: 10.1101/GR.213959.116.

38. A B, EL M, M K, AE P, Z W, A S, et al.. High-quality genome sequences of uncultured microbes by assembly of read clouds. *Nat Biotechnol*. Nat Biotechnol; 2018; doi: 10.1038/NBT.4266.

29. Visendi P. De Novo Assembly of Linked Reads Using Supernova 2.0. *Methods Mol Biol*. Humana Press Inc.; 2022; doi: 10.1007/978-1-0716-2067-0_12/COVER.

30. Zhang 39. I T, A B, Z, Wang H, Yang C, Huang Y, Yue Z, Chen Y, et al.. Exploring high-quality microbial genomes byPA P. cloudSPAdes: assembly of linked synthetic long reads with high barcode specificity using deep learning. *bioRxiv*. Cold Spring Harbor Laboratory; 2022de Bruijn graphs. *Bioinformatics*. Bioinformatics; 2019; doi: 10.1093/BIOINFORMATICS/BTZ349.

40. Kang DD, Li F, Kirton E, Thomas A, Egan R, An H, et al.. MetaBAT 2: An adaptive binning algorithm for robust and efficient genome reconstruction from metagenome assemblies. *PeerJ*. PeerJ Inc.; 2019; doi: 10.1101/2022.09.07.5069637717/PEERJ.7359/SUPP-3.

3141. Zhou X, Batzoglou S, Sidow A, Zhang L. HAPDeNovo: A haplotype-based approach for filtering and phasing de novo mutations in linked read sequencing data. *BMC Genomics*. BioMed Central Ltd.; 2018; doi: 10.1186/S12864-018-4867-7/TABLES/3.

42. Chaisson MJP, Sanders AD, Zhao X, Malhotra A, Porubsky D, Rausch T, et al.. Multi-platform discovery of haplotype-resolved structural variation in human genomes. *Nat Commun 2019 101*. Nature Publishing Group; 2019; doi: 10.1038/s41467-018-08148-z.

3243. Kavak P, Lin YY, Numanagić I, Asghari H, Güngör T, Alkan C, et al.. Discovery and genotyping of novel sequence insertions in many sequenced individuals. *Bioinformatics*. Oxford Academic; 2017; doi: 10.1093/BIOINFORMATICS/BTX254.

44. Krannich T, Timothy W, Niehus S, Holley G, Halldorsson B V., Kehr B. Population-scale detection of non-reference sequence variants using colored de Bruijn graphs. *Bioinformatics*. Oxford Academic; 2022; doi: 10.1093/BIOINFORMATICS/BTAB749.

45. Altshuler DL, Durbin RM, Abecasis GR, Bentley DR, Chakravarti A, Clark AG, et al.. A map of human genome variation from population-scale sequencing. *Nature*. 2010; doi: 10.1038/nature09534.

46. E P, F A, S M, M Z, N K, F A, et al.. Extensive Unexplored Human Microbiome Diversity Revealed by Over 150,000 Genomes from Metagenomes Spanning Age, Geography, and Lifestyle. *Cell*. Cell; 2019; doi: 10.1016/J.CELL.2019.01.001.

47. Xia LC, Bell JM, Wood-Bouwens C, Chen JJ, Zhang NR, Ji HP. Identification of large rearrangements in cancer genomes with barcode linked reads. *Nucleic Acids Res*. Oxford Academic; 2018; doi: 10.1093/NAR/GKX1193.

48. Zhang F, Christiansen L, Thomas J, Pokholok D, Jackson R, Morrell N, et al.. Haplotype phasing of whole human genomes using bead-based barcode partitioning in a single tube. *Nat Biotechnol 2017 359*. Nature Publishing Group; 2017; doi: 10.1038/nbt.3897.

3349. Meier JI, Salazar PA, Kučka M, Davies RW, Dréau A, Aldás I, et al.. Haplotype tagging reveals parallel formation of hybrid races in two butterfly species. *Proc Natl Acad Sci U S A*. National Academy of Sciences; 2021; doi: 10.1073/PNAS.2015005118/SUPPL_FILE/PNAS.2015005118.SD06.XLSX.

3450. Redin D, Frick T, Aghelpasand H, Käller M, Borgström E, Olsen RA, et al.. High throughput barcoding method for genome-scale phasing. *Sci Reports 2019 91*. Nature Publishing Group; 2019; doi: 10.1038/s41598-019-54446-x.

3551. Zheng W, Zhao S, Yin Y, Zhang H, Needham DM, Evans ED, et al.. High-throughput, single-microbe genomics with strain resolution, applied to a human gut microbiome. *Science (80-)*. American Association for the Advancement of Science; 2022; doi: 10.1126/SCIENCE.ABM1483/SUPPL_FILE/SCIENCE.ABM1483_MOVIES_S1_TO_S10.ZIP.

3652. Chen S, Zhou Y, Chen Y, Gu J. fastp: an ultra-fast all-in-one FASTQ preprocessor. *Bioinformatics*. Oxford Academic; 2018; doi: 10.1093/BIOINFORMATICS/BTY560.

37. DD K, F L, E K, A T, R E, H A, et al.. MetaBAT 2: an adaptive binning algorithm for robust and efficient genome reconstruction from metagenome assemblies. *PeerJ*. PeerJ; 2019; doi: 10.7717/PEERJ.7359.

3853. Bowers RM, Kyrpides NC, Stepanauskas R, Harmon-Smith M, Doud D, Reddy TBK, et al.. Minimum information about a single amplified genome (MISAG) and a

metagenome-assembled genome (MIMAG) of bacteria and archaea. *Nat Biotechnol 2017 358*. Nature Publishing Group; 2017; doi: 10.1038/nbt.3893.

54. Schneider VA, Graves-Lindsay T, Howe K, Bouk N, Chen HC, Kitts PA, et al.. Evaluation of GRCh38 and de novo haploid genome assemblies demonstrates the enduring quality of the reference assembly. *Genome Res*. Cold Spring Harbor Laboratory Press; 2017; doi: 10.1101/GR.213611.116.

~~39~~55. Parks DH, Chuvochina M, Rinke C, Mussig AJ, Chaumeil P-A, Hugenholtz P. GTDB: an ongoing census of bacterial and archaeal diversity through a phylogenetically consistent, rank normalized and complete genome-based taxonomy. *Nucleic Acids Res*. Oxford University Press (OUP); 2021; doi: 10.1093/NAR/GKAB776.

56. Majidian S, Sedlazeck FJ. PhaseME: Automatic rapid assessment of phasing quality and phasing improvement. *Gigascience*. Oxford University Press; 2020; doi: 10.1093/gigascience/giaa078.

57. Zhou Y, Browning SR, Browning BL. A Fast and Simple Method for Detecting Identity-by-Descent Segments in Large-Scale Data. *Am J Hum Genet*. Cell Press; 2020; doi: 10.1016/J.AJHG.2020.02.010.

58. Jamal-Hanjani M, Wilson GA, McGranahan N, Birkbak NJ, Watkins TBK, Veeriah S, et al.. Tracking the Evolution of Non-Small-Cell Lung Cancer. *N Engl J Med*. N Engl J Med; 2017; doi: 10.1056/NEJMOA1616288.

59. Bishara A, Liu Y, Weng Z, Kashef-Haghighi D, Newburger DE, West R, et al.. Read clouds uncover variation in complex regions of the human genome. *Genome Res*. Genome Res; 2015; doi: 10.1101/GR.191189.115.

Figures and Tables

Figure 1. Overview of LRTK. The LRTK workflow includes a metagenomic section (left panel) and human genomic section (right panel). The metagenomic section implements barcode correction, ~~read cloud assembly,~~ barcode-aware alignment and metagenome assembly, long DNA fragment reconstruction， taxonomic classification and quantification, as well as SNV detection and phasing. The human genomic section implements barcode correction, barcode-aware alignment, long DNA fragment reconstruction, and detection and phasing of SNVs, INDELs and SVs.

Figure 2. ~~Quality~~Distribution of quality metrics for different linked-read sequencing platforms. (A) ~~Quality metrics under~~ metagenome sequencing, **(B)** human genome sequencing. The left panel displays the distribution of the number of long DNA fragments per barcode ~~for 10x Genomics, stLFR and INDEL in metagenomic sequencing data, respectively.~~. The middle panel shows the ~~DNA~~ length distributions of reconstructed long DNA fragments. The right panel shows the ~~average~~ distribution of short-read coverage ~~per fragment. **(B)** Quality metrics under human genome sequencing. The left panel shows the number~~ of fragments ~~per barcode, while~~

Formatted: Font: Bold

~~the middle panel shows the DNA length of reconstructed fragments. The right panel shows the average read coverage per fragment. ~~.

Figure 3. Comparison of linked-read based metagenomic quantification, SNV identification and phasing. (A) Evaluation of tools to quantify taxonomic abundance based on the linked-~~read metagenomic sequencing data.~~reads from simulated dataset (left panel) and ATCC-MSA-1003 (right panel). **(B)** ~~Comparison~~Comparing the performance of ~~tools~~SAMtools, FreeBayes and inStrain to detect metagenomic SNVs. **(C)** Dynamic changes of taxonomic abundance and allele frequency. **(D)** SNV based strain phasing in pairwise samples. The right label shows the sample name in D1. BAF is the abbreviation of B allele frequency.

Figure 4. Evaluation of metagenome assemblies on linked-read ~~based metagenomic assembly.~~sequencing. (A) Evaluation of the assembly performance for MEGAHIT, metaSPAdes, Athena, ~~Supernova~~cloudSPAdes and Pangaea ~~using~~on 10x Genomics, stLFR and TELL-Seq ~~sequencing.~~linked-read sequencing data from ATCC-MSA-1003. Pangaea does not support 10x Genomics linked-reads. The left panel demonstrates the calculated NA50 values while the right panel shows the calculated N50 values. **(B)** Illustration of the two assembled circular contigs. **(C)** The distribution of completeness and contamination for reconstructed bins. **(D)** The number of contigs in each contig group. **(E)** The number of detected tRNAs in each contig group.

Figure 5. Evaluation of linked-read based detection of variation in the human genome. (A, B) Performance metrics on the detection of SNVs and INDELs using

Formatted: Font: Not Bold

Formatted: Font: Times New Roman, Font color: Custom Color(RGB(35,31,32))

FreeBayes, GATK and SAMtools for 10x Genomics, stLFR and TELL-Seq. **(C, D)** The performance on phasing of small variants using HapCUT2 and WhatsHap for 10x Genomics, stLFR and TELL-Seq. **(E)** Illustration of the ~~improved~~ performance on IBD detection. **(F~~, G~~)** The performance on detection of ~~large~~ deletions ~~**(F)** and insertions **(G)**~~ using Aquila, LinkedSV and LongRanger. **(G)** The performance on detection of insertions using Aquila, Pamir and PopIns2.

~~**Supplementary**~~ **Figure ~~1~~S1: LRTK text file specification. (A)** An example of the unified FASTQ format. Each read contains a barcode field “BX:Z:barcode” after the read names. The lengths of barcodes are 16 ~~bp~~bps, 30 ~~bp~~bps and 18 ~~bp~~bps for 10x Genomics linked-read, stLFR and TELL-Seq, respectively. **(B)** An example of the BAM file generated by LRTK. The barcode information is stored in the “BX:Z:barcode” field.

~~**Supplementary**~~ **Figure ~~2~~S2. Ideogrammatic definitions of C_R, C_F, $N_{F/P}$, μ_{FL}, and $W\mu_{FL}$ metrics.** (**A**) C_R: Average depth of short reads per fragment. (**B**) C_F: Average physical depth of the genome by long DNA fragments. (**C**) $N_{F/P}$: Number of fragments per barcode. (**D**) Length-weighted average (μ_{FL}) and unweighted average ($W\mu_{FL}$) of DNA fragment lengths. Here, μFL was calculated as the mean DNA fragment length while WμFL was calculated as the N50 value of all the DNA fragments.

~~**Supplementary Figure 3.**~~**Figure S3. Evaluation of taxonomic quantification performance for 10x Genomics and TELL-Seq linked-reads.** (**A**) Evaluation of taxonomic quantification across LRTK, Bracken, KMCP, MetaPhlAn 2 and MIDAS 2

using the simulated 10x Genomics linked-reads. (**B**) Evaluation of taxonomic quantification across LRTK, Bracken, KMCP, MetaPhlAn 2 and MIDAS 2 using the 10x Genomics linked-reads from ATCC-MSA-1003. (**C**) Evaluation of taxonomic quantification across LRTK, Bracken, KMCP, MetaPhlAn 2 and MIDAS 2 using the TELL-Seq linked-reads from ATCC-MSA-1003.

Figure S4: Evaluation of assembly performance at species level. The left panel demonstrates the average assembled genome fraction, NA50 and N50 for ATCC-MSA-1003. The right panel demonstrates the average assembled genome fraction, NA50 and N50 from the simulated dataset.

Figure S5: Evaluation of metagenomic assemblers on simulated linked-read data. The top panel illustrates the NA50 values across five assemblers based on the simulated data. The bottom panel illustrates the N50 values across five assemblers based on the simulated sequencing data.

Figure S6. Demo reports for metagenomics sequencing. (**A**) Per base sequencing quality scores along the reads. (**B**) Per Base GC content along the reads. (**C**) The number of fragments per barcode. (**D**) Fragment length distribution. (**E**). The abundance of the top 10 most abundant species. (**F**) The number of SNVs per species. (**G**) A principal component analysis using sample data from different groups.

~~Supplementary~~ Figure ~~4~~S7: Demo reports for human genome sequencing. (**A**) Per base sequencing quality scores along the reads. (**B**) Per Base GC content along the reads. (**C**) The number of fragments per barcode. (**D**) Fragment length distribution. (**E**) Sequencing depth frequency. (**F**) The distribution of sequencing depth along a

whole genome. (**G**) The distribution of inferred insert sizes. (**H**) The distribution of detected deletions. (**I**) The distribution of detected insertions.

Figure S8: Computational requirements for linked-reads preprocessing and alignment between LRTK and Long Ranger on NA12878. (A) linked-reads preprocessing; **(B)** linked-reads alignment.

Formatted: Line spacing: single

Formatted: Font: Times New Roman, Bold

Table S1~~: Comparison of LRTK with other published linked-read pipelines.~~

	~~**Items**~~	~~**Long Ranger**~~	~~**LRTK**~~
~~**Input**~~	~~10x Genomics~~	~~Y~~	~~Y~~
	~~stLFR~~	~~N~~	~~Y~~
	~~TELL-Seq~~	~~N~~	~~Y~~
~~**Modules**~~	~~Linked-read preprocessing~~	~~Y~~	~~Y~~
	~~metagenome quantification~~	~~N~~	~~Y~~
	~~metagenome SNV detection~~	~~N~~	~~Y~~
	~~metagenome phasing~~	~~N~~	~~Y~~
	~~metagenome assembly~~	~~N~~	~~Y~~
	~~metagenome downstream analysis~~	~~N~~	~~Y~~
	~~human genome alignment~~	~~Y~~	~~Y~~
	~~human genome SNV detection~~	~~Y~~	~~Y~~
	~~human genome SV detection~~	~~Y~~	~~Y~~
	~~human genome phasing~~	~~Y~~	~~Y~~
	~~human genome downstream analysis~~	~~N~~	~~Y~~

~~**Table S2**~~**. Bioinformatics tools included in LRTK.**

**Categories**	**Tools**
Linked read simulation	~~LRTK-SIM[23]~~LRTK-SIM [29]
Read preprocessing	~~fastp[36]~~fastp [52]
~~Barcode mapping and correction~~	~~BWA[22]~~
~~Read-cloud~~Barcode-aware metagenome assembly	~~Pangaea[30]~~Pangaea [28]

Formatted: Font: Not Bold

Formatted: Font: Not Bold

Formatted: Font: 10.5 pt, Not Bold

Formatted: Font: 10.5 pt

Formatted: Font: 10.5 pt

Formatted: Font: 10.5 pt, Not Bold

Formatted: Font: 10.5 pt

Formatted: Font: 10.5 pt, Not Bold

Formatted: Font: 10.5 pt

Barcode-aware read alignment	~~Modified EMA[12]~~ Modified EMA [18] , lariat [59]
SNV detection	~~FreeBayes[14], GATK[16], inStrain[27], SAMtools[15]~~FreeBayes [20], GATK [22], inStrain [35], SAMtools [21]
~~Linked-read-based~~Barcode-assited SV detection	~~Aquila[17], LinkedSV[18], VALOR2[19],~~ Aquila [23], LinkedSV [24]
~~Linked-read-based~~Barcode-assited phasing	~~HapCUT2[20], WhatsHap[21]~~HapCUT2 [26], WhatsHap [27]

Table ~~S3~~S2. Linked-read sequencing data sources.

Cohort	Sample	Sequencing Technologies	Data sources	Size
B1	ATCC-MSA-1003	10x Genomics	SRR12283286	37.7G
		stLFR	PRJNA875547	111G
		TELL-Seq	PRJNA875547	55G
D1	~~MPS_stool_lyme_10~~T10	10x Genomics	SRR14763277	14.8G
	~~MPS_stool_lyme_8~~T8		SRR14763279	6.9G
	~~MPS_stool_lyme_6~~T6		SRR14763281	8.2G
	~~MPS_stool_lyme_5~~T5		SRR14763282	40.6G
	~~MPS_stool_lyme_4~~T4		SRR14763283	39.5G
	~~MPS_stool_lyme_3~~T3		SRR14763284	4.1G
	~~MPS_stool_lyme_18~~T18		SRR14763286	10.4G
	~~MPS_stool_lyme_17~~T17		SRR14763287	18G
	~~MPS_stool_lyme_16~~T16		SRR14763288	20.4G
	~~MPS_stool_lyme_15~~T15		SRR14763289	8.4G
	~~MPS_stool_lyme_14~~T14		SRR14763290	38.5G
	~~MPS_stool_lyme_13~~T13		SRR14763291	10.3G
	~~MPS_stool_lyme_12~~T12		SRR14763292	11.6G

Formatted: Font: 10.5 pt, Not Bold
Formatted: Font: 10.5 pt
Formatted: Font: 10.5 pt
Formatted: Font: 10.5 pt, Not Bold
Formatted: Font: 10.5 pt
Formatted: Font: 10.5 pt
Formatted: Font: 10.5 pt, Not Bold
Formatted: Font: 10.5 pt
Formatted: Font: 10.5 pt
Formatted: Font: 10.5 pt, Not Bold
Formatted: Font: 10.5 pt
Formatted: Font: 10.5 pt
Inserted Cells
Formatted: Font: 12 pt
Formatted: Font: 12 pt
Formatted: Font: 12 pt
Formatted: Font: 12 pt
Formatted: Font: Bold
Formatted: Font: Bold

	~~MPS_stool_lyme_11~~T11		SRR14763293	75G
	~~MPS_stool_lyme_2~~T2		SRR14763294	38.1G
	~~MPS_stool_lyme_1~~T1		SRR14763295	38.6G
D2	S1	stLFR	CNP0003432 (CNR0585102)	98.97 G
B2	NA12878	10x Genomics	https://ftp-trace.ncbi.nlm.nih.gov/giab/ftp/data/NA12878/10Xgenomics_ChromiumGenome_LongRanger2.0_06202016/NA12878.fastqs/	142.1 G
		stLFR	https://ftp-trace.ncbi.nlm.nih.gov/giab/ftp/data/NA12878/stLFR/	251 G
		TELL-Seq	https://www.ncbi.nlm.nih.gov/sra/SRX7264479	100.4G
D3	NA24385	10x Genomics	https://ftp-trace.ncbi.nlm.nih.gov/giab/ftp/data/AshkenazimTrio/HG002_NA24385_son/10Xgenomics_ChromiumGenome/NA24385.fastqs/	160.3 G
		stLFR	https://ftp-trace.ncbi.nlm.nih.gov/giab/ftp/data/AshkenazimTrio/HG002_NA24385_son/stLFR/	227 G
		TELL-Seq	https://www.ncbi.nlm.nih.gov/sra/SRX7264481	109.5 G
	NA24143	10x Genomics	https://ftp-trace.ncbi.nlm.nih.gov/giab/ftp/data/AshkenazimTri	83.3 G

			o/HG004_NA24143_mother/10Xgenomics_ChromiumGenome/NA24143.fastqs/	
		stLFR	https://ftp-trace.ncbi.nlm.nih.gov/giab/ftp/data/AshkenazimTrio/HG004_NA24143_mother/stLFR/	222 G
		TELL-Seq	NA	NA
	NA24149	10x Genomics	https://ftp-trace.ncbi.nlm.nih.gov/giab/ftp/data/AshkenazimTrio/HG003_NA24149_father/10Xgenomics_ChromiumGenome/NA24149.fastqs/	78.3 G
		stLFR	https://ftp-trace.ncbi.nlm.nih.gov/giab/ftp/data/AshkenazimTrio/HG003_NA24149_father/stLFR/	248 G
		TELL-Seq	NA	NA

Table S3. Data descriptions for the simulated and down-sampled linked-reads from ATCC-MSA-1003 and NA12878.

	Simulated dataset	
**Statistics**	**10x Genomics**	**stLFR**
Total Reads (Mb)	134.1	92.17
Total Bases (Gb)	11.87	9.22
Q20 Bases (Gb)	11.07	8.57

Formatted: Font: Times New Roman

Formatted: Font: Times New Roman

Q30 Bases (Gb)	10.41	8.02	
GC content	54.87%	50.63%	
reads passed filters	99.99%	99.99%	
reads with low quality	0	0	
reads with too many N	< 0.01%	< 0.01%	
reads too short	< 0.01%	< 0.01%	
Total number of barcodes	228,824	1,887,428	
Mean number of read pairs per barcode	293.02	24.42	
Median number of read pairs per barcode	284	17	
	**ATCC1003**		
**Statistics**	**10x Genomics**	**stLFR**	**TELL-Seq**
Total Reads (Mb)	151.85	201.2	156.34
Total Bases (Gb)	19.27	20.12	20.25
Q20 Bases (Gb)	19.17	19.12	19.61
Q30 Bases (Gb)	18.9	17.07	18.69
GC content	53.14%	48.75%	46.20%
reads passed filters	99.59%	94.71%	99.99%
reads with low quality	0	0.0348	0
reads with too many N	< 0.01%	< 0.01%	< 0.01%
reads too short	< 0.01%	0.0186	< 0.01%
Total number of barcodes	710,283	8,894,042	3,652,328
Mean number of read pairs per barcode	106.89	12.0864	22.81
Median number of read pairs per barcode	26	6	3
	**NA12878**		
**Statistics**	**10x Genomics**	**stLFR**	**TELL-Seq**
Total Reads (Mb)	794.35	1,097.29	754.79
Total Bases (Gb)	108.85	109.73	110.2
Q20 Bases (Gb)	102.84	104.02	105.27
Q30 Bases (Gb)	96.57	92.3	99.73
GC content	41.50%	41.91%	45.00%
reads passed filters	99.99%	95.73%	94.15%
reads with low quality	< 0.01%	3.09%	0.43%
reads with too many N	< 0.01%	< 0.01%	< 0.01%
reads too short	< 0.01%	1.18%	5.41%
Total number of barcodes	2,331,331	15,826,079	16,909,555
Mean number of read pairs per barcode	170.36	35.61966	23.42
Median number of read pairs per barcode	7	2	1

Table S4. Reference genomes for the ATCC-MSA-1003 mock sample.

**Organism name**	**Relative abundance**	**Reference genome**	**Genome size (bp)**	**ATCC links**
Acinetobacter_baumannii	0.18%	ATCC 17978	4,075,779	https://genomes.atcc.org/genomes/e1d18ea4273549a0
Bacillus_pacificus	1.80%	ATCC 10987	5,442,819	https://genomes.atcc.org/genomes/687931d9b06b4cb4
Bifidobacterium_adolescentis	0.02%	ATCC 15703	2,089,630	https://genomes.atcc.org/genomes/90eb97d11e4b445f
Cereibacter_sphaeroides	18.00%	ATCC 17029	2,089,630	https://genomes.atcc.org/genomes/3a2ecd8337b14710
Clostridium_beijerinckii	1.80%	ATCC 35702	6,007,460	https://genomes.atcc.org/genomes/3210fc7fdeb14ad2
Cutibacterium_acnes	0.18%	ATCC 11828	2,497,484	https://genomes.atcc.org/genomes/070cc66203ff4f5a
Deinococcus_radiodurans	0.02%	ATCC BAA-816	3,280,465	https://genomes.atcc.org/genomes/2c9f475933234e78
Enterococcus_faecalis	0.02%	ATCC 47077	2,738,556	https://genomes.atcc.org/genomes/d8b30e7d0fd74a12
Escherichia_coli	18.00%	ATCC 700926	4,642,497	https://genomes.atcc.org/genomes/8f5bf4f0d4a04c50
Helicobacter_pylori	0.18%	ATCC 700392	1,667,794	https://genomes.atcc.org/genomes/9038b5a9e94245e8
Lactobacillus_gasseri	0.18%	ATCC 33323	1,854,273	https://genomes.atcc.org/genomes/b926c8c7fe4440b5

Neisseria_meningitidis	0.18%	ATCC BAA-335	2,243,896	https://genomes.atcc.org/genomes/261b0e41db924d0f
Phocaeicola_vulgatus	0.02%	ATCC 8482	5,163,177	https://genomes.atcc.org/genomes/b7dcc8d57632456b
Porphyromonas_gingivalis	18.00%	ATCC 33277	2,399,479	https://genomes.atcc.org/genomes/0472781aae86424a
Pseudomonas_paraeruginosa	1.80%	ATCC 9027	6,374,461	https://genomes.atcc.org/genomes/c1a92e4fc09a4ed2
Schaalia_odontolytica	0.02%	ATCC 17982	2,396,044	https://genomes.atcc.org/genomes/6823ab7475dd4769
Staphylococcus_aureus	1.80%	ATCC BAA-1556	2,923,627	https://genomes.atcc.org/genomes/63b4b3239efa42df
Staphylococcus_epidermidis	18.00%	ATCC 12228	2,575,951	https://genomes.atcc.org/genomes/d1ef0271f5b14846
Streptococcus_agalactiae	1.80%	ATCC BAA-611	2,159,783	https://genomes.atcc.org/genomes/e74f122703624e54
Streptococcus_mutans	18.00%	ATCC 700610	2,031,444	https://genomes.atcc.org/genomes/d0622592e00d4ee0

Table S5. Reference genomes for the simulated dataset.

**Organism name**	**Relative abundance**	**Reference genome**	**Genome size (bp)**
Thermoanaerobacter pseudethanolicus ATCC 33223	0.003055997	NC_010321.1	2,362,816
Desulfitobacterium hafniense DCB-2	0.00607231	NC_011830.1	5,279,134

Desulfofarcimen acetoxidans DSM 771	0.004800511	NC_013216.1	4,545,624
Xylanimonas cellulosilytica DSM 15894	0.039646357	NC_013530.1	3,831,380
Sulfurimonas autotrophica DSM 16294	0.088701172	NC_014506.1	2,153,198
Corynebacterium resistens DSM 45100	0.002236753	NC_015673.1	2,601,311
Bartonella clarridgeiae 73	0.020130366	NC_014932.1	1,522,743
Isoalcanivorax pacificus W11-5	0.015516627	NZ_CP004387.1	4,168,427
Pseudobdellovibrio exovorus JSS	0.003212947	NC_020813.1	2,657,893
Psychromicrobium lacuslunae	0.010557049	NZ_CP011005.1	3,599,434
Helicobacter typhlonius	0.020004482	NZ_LN907858.1	1,920,832
Wolbachia endosymbiont of Folsomia candida	0.05823542	NZ_CP015510.2	1,801,626
Sporosarcina ureae	0.017837164	NZ_CP015108.1	3,362,333
Sedimentisphaera salicampi	0.003382715	NZ_CP021023.1	3,192,293
Fuscovulum blasticum	0.027337676	NZ_CP020470.1	3,706,095
Dongshaea marina	0.030624348	NZ_CP028897.1	5,112,152
Runella rosea	0.006440272	NZ_CP030850.1	7,162,093
Desulfovibrio ferrophilus	0.010084579	NZ_AP017378.1	3,720,107
Bradyrhizobium guangzhouense	0.018027951	NZ_CP030053.1	8,138,177
Haematobacter massiliensis	0.004961852	NZ_CP035510.1	4,232,912
Cellulomonas shaoxiangyii	0.09751048	NZ_CP039291.1	3,909,366

Microlunatus elymi	0.011982578	NZ_CP041692.1	5,121,194
Kushneria phosphatilytica	0.033312055	NZ_CP043420.1	3,624,619
Lactiplantibacillus plantarum	0.007816798	NZ_CP028221.1	3,223,643
Mycolicibacterium tokaiense	0.013904496	NZ_AP022600.1	6,328,149
Mycolicibacillus koreensis	0.008194722	NZ_AP022594.1	4,155,701
Lactococcus garvieae	0.00591555	NZ_CP065637.1	2,084,337
Rhizorhabdus wittichii	0.036707087	NZ_CP059319.1	6,073,182
Parabacteroides goldsteinii	0.006468574	NZ_CP081906.1	7,053,599
Pseudomonas savastanoi	0.007014885	NZ_CP076652.1	5,999,881
Escherichia coli str. K-12 substr. MG1655	0.175270285	NZ_CP097882.1	4,675,188
Salinimicrobium tongyeongense	0.013594968	NZ_CP069620.1	3,509,958
Iamia majanohamensis	0.005263885	NZ_CP116942.1	4,576,919
Ligilactobacillus faecis	0.012418841	NZ_CP123639.1	2,382,213
Borreliella kurtenbachii	0.014520015	NZ_CP124058.1	1,015,378
Staphylococcus schweitzeri	0.004400918	NZ_LR134304.1	2,784,939
Cardiobacterium hominis	0.00908479	NZ_LR134365.1	2,670,755
Slackia heliotrinireducens	0.005737567	NZ_LR134379.1	3,166,158
Wolbachia endosymbiont (group A) of Icerya purchasi	0.007627304	NZ_OX366357.1	1,372,034
Wolbachia endosymbiont (group A) of Hylaeus communis	0.132387656	NZ_OX366396.1	1,526,905

Figure1

Click here to access/download;Figure;Figure1.pdf

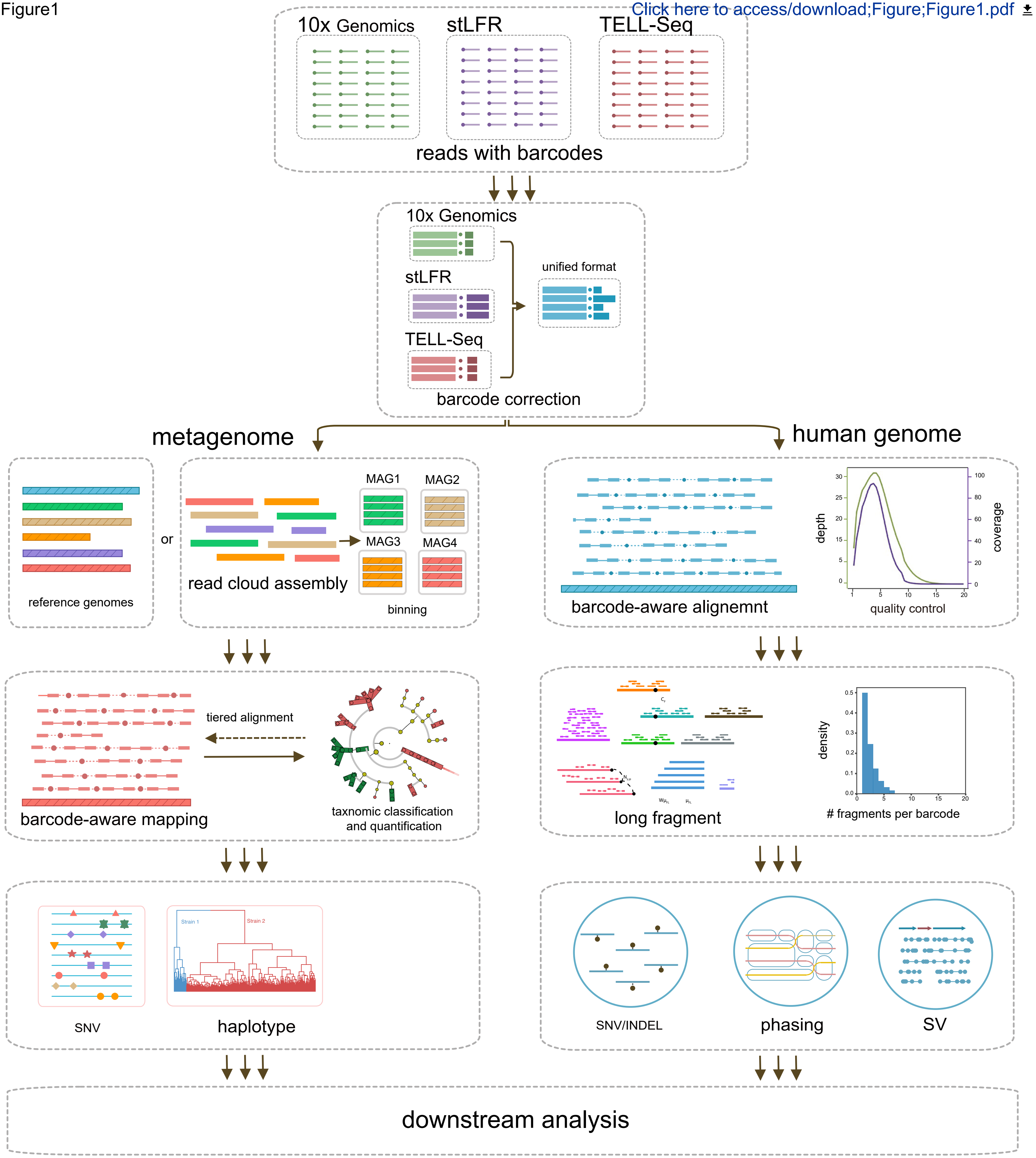

Figure2

Click here to access/download;Figure;Figure2.pdf

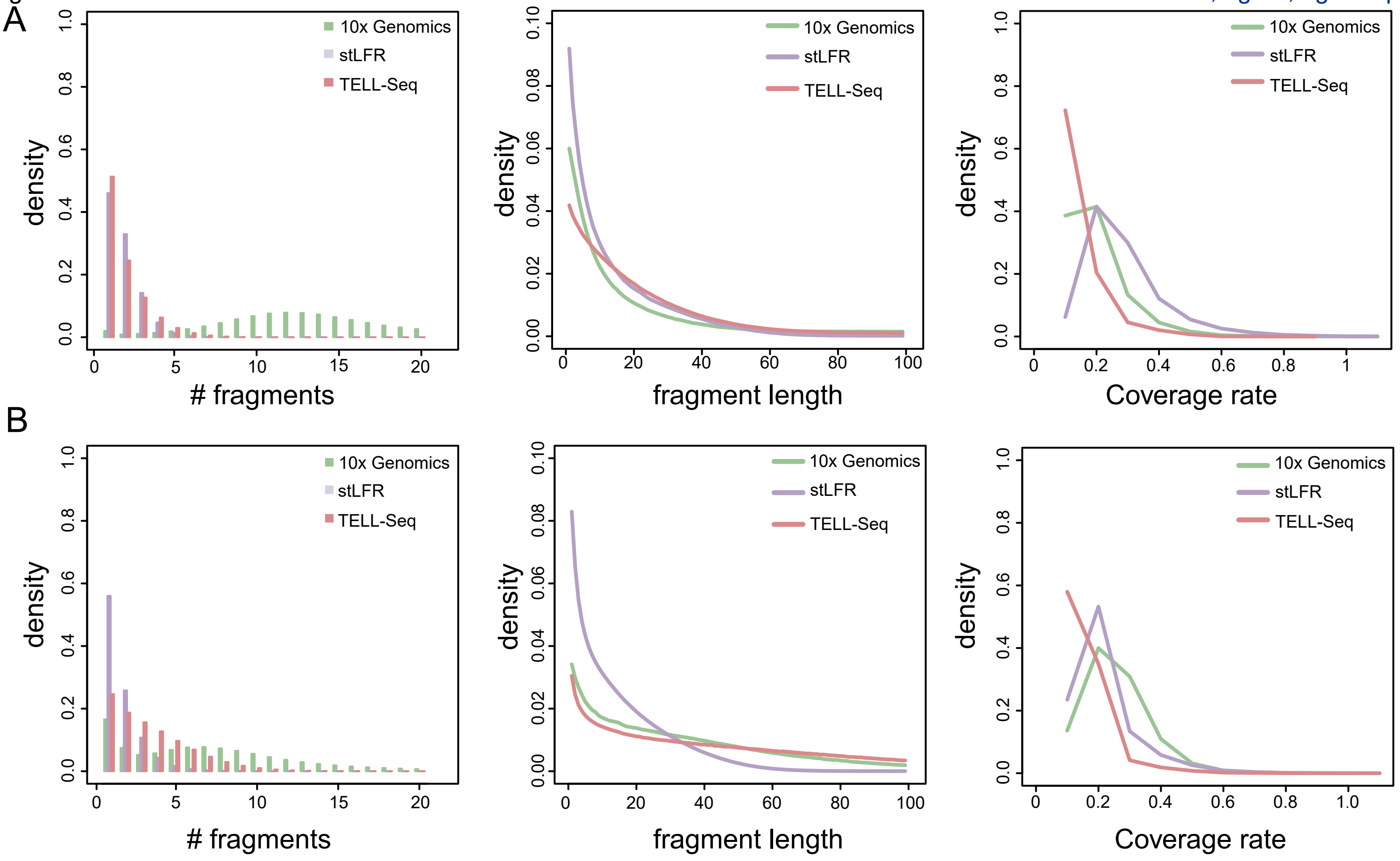

Figure3

Click here to access/download;Figure;Figure3.pdf

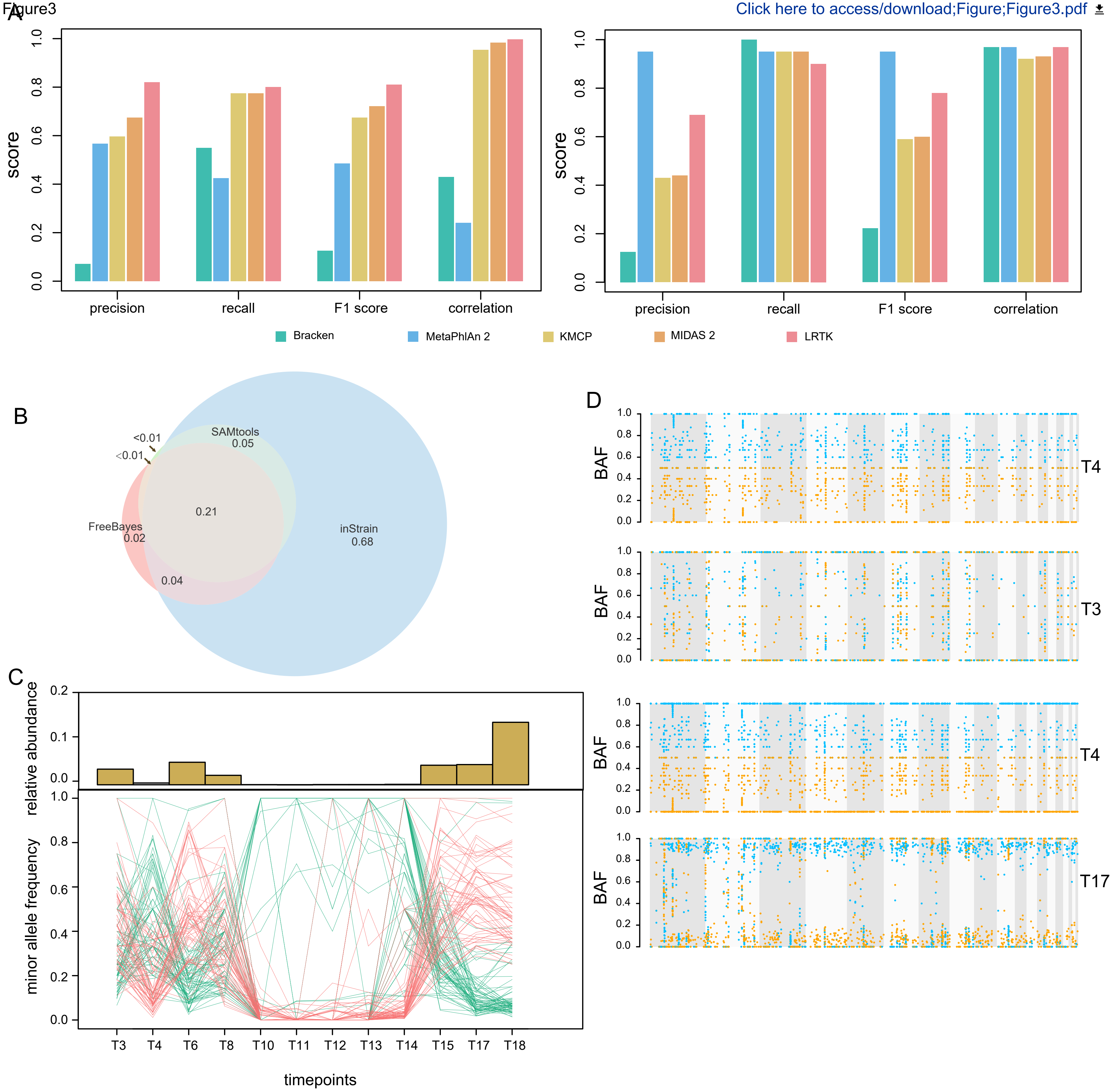

Figure4

Click here to access/download;Figure;Figure4.pdf

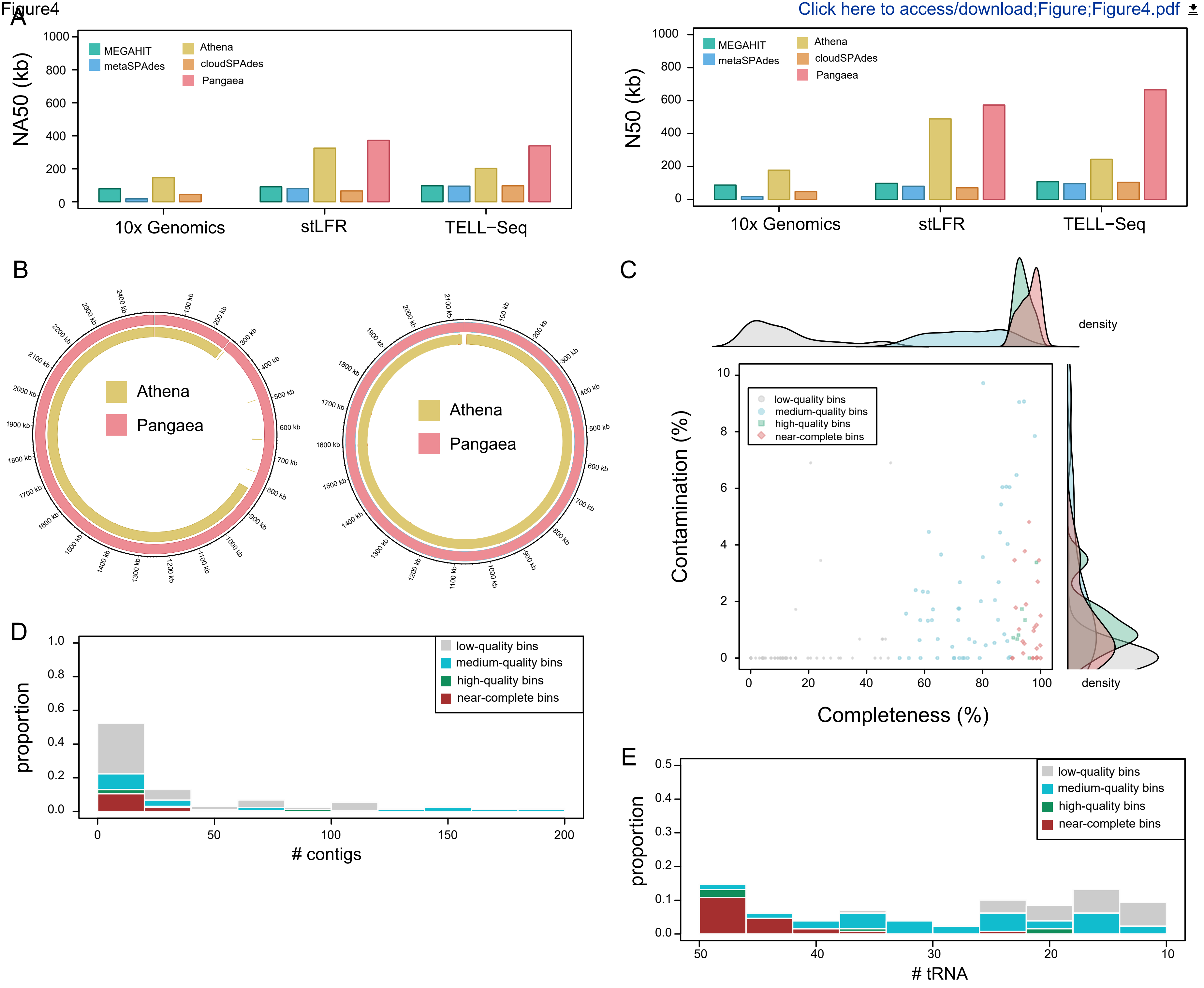

Figure5

Click here to access/download;Figure;Figure5.pdf

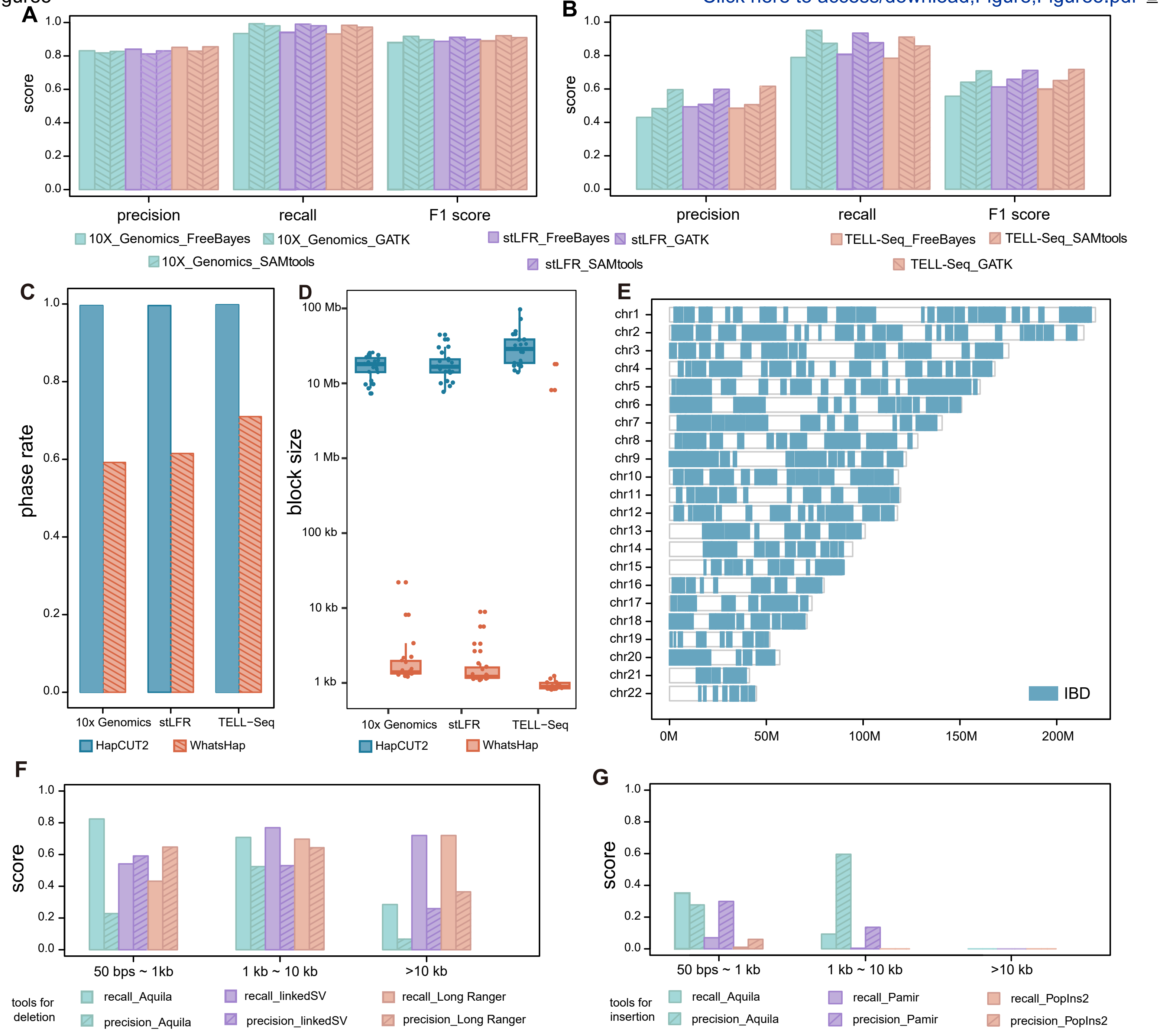

FigureS1

Click here to access/download
Supplementary Material
FigureS1.pdf

FigureS2

Click here to access/download

Supplementary Material

FigureS2.pdf

FigureS3

Click here to access/download
Supplementary Material
FigureS3.pdf

FigureS4

Click here to access/download
Supplementary Material
FigureS4.pdf

FigureS5

Click here to access/download
Supplementary Material
FigureS5.pdf

FigureS6

Click here to access/download
Supplementary Material
FigureS6.pdf

FigureS7

Click here to access/download
Supplementary Material
FigureS7.pdf

FigureS8

Click here to access/download
Supplementary Material
FigureS8.pdf

Click here to access/download
Supplementary Material
SupplementaryTables.xlsx
