# Supplementary material for: LRTK: a platform agnostic toolkit for linked-read analysis of both human genome and metagenome
Source: Gigascience. 2024 Jun 13;13:giae028. doi: 10.1093/gigascience/giae028 (PMC11170215; doi:10.1093/gigascience/giae028)
Supplement: giae028_GIGA-D-23-00278_Revision_2 [file giae028_giga-d-23-00278_revision_2.pdf]

GigaScience

LRTK: A platform agnostic toolkit for linked-read analysis of both human genome and metagenome

--Manuscript Draft--

**Manuscript Number:**	GIGA-D-23-00278R2
**Full Title:**	LRTK: A platform agnostic toolkit for linked-read analysis of both human genome and metagenome
**Article Type:**	Technical Note
**Funding Information:**	

Funder	Recipient
open project of BGI-Shenzhen (BGIRSZ20220012)	Dr. Lu Zhang
Hong Kong Research Grant Council Early Career Scheme (HKBU 22201419)	Dr. Lu Zhang
HKBU Start-up Grant Tier 2 (RC-SGT2/19-20/SCI/007)	Dr. Lu Zhang
HKBU IRCMS (No. IRCMS/19-20/D02)	Dr. Lu Zhang
Basic and Applied Basic Research Foundation of Guangdong Province (No. 2021A1515012226)	Dr. Lu Zhang
Science Technology and Innovation Committee of Shenzhen Municipality (SGDX20190919142801722)	Dr. Xiaodong Fang
Young Collaborative Research Grant (C2004-23Y)	Dr. Lu Zhang
Infectious and Tropical Diseases Research Center, Health Research Institute, Ahvaz Jundishapur University of Medical Sciences (11221026)	Dr. Lu Zhang

Abstract:

Background: Linked-read sequencing technologies generate high-base quality short-reads that contain extrapolative information on long-range DNA connectedness. These advantages of linked-read technologies are well-known and have been demonstrated in many human genomic and metagenomic studies. However, existing linked-read analysis pipelines (e.g., Long Ranger) were primarily developed to process sequencing data from the human genome and are not suited for analyzing metagenomic sequencing data. Moreover, linked-read analysis pipelines are typically limited to one specific sequencing platform.

Findings: To address these limitations, we present the Linked-Read ToolKit (LRTK), a unified and versatile toolkit for platform agnostic processing of linked-read sequencing data from both human genome and metagenome. LRTK provides functions to perform linked-read simulation, barcode sequencing error correction, barcode-aware read alignment and metagenome assembly, reconstruction of long DNA fragments, taxonomic classification and quantification, as well as barcode-assisted genomic variant calling and phasing. LRTK has the ability to process multiple samples automatically, and provides users with the option to generate reproducible reports during processing of raw sequencing data and at multiple checkpoints throughout downstream analysis. We applied LRTK on linked-reads from simulation, mock community and real datasets for both human genome and metagenome. We showcased LRTK's ability to generate comparative performance results from preceding benchmark studies and to report these results in publication-ready HTML document plots.

Conclusions: LRTK provides comprehensive and flexible modules along with an easy-to-use Python-based workflow for processing linked-read sequencing datasets, thereby filling the current gap in the field caused by platform-centric genome-specific linked-read data analysis tools.

Corresponding Author: Lu Zhang
Hong Kong Baptist University
Hong Kong, Please select a state CHINA

Powered by Editorial Manager® and ProduXion Manager® from Aries Systems Corporation

**Corresponding Author Secondary Information:**	
**Corresponding Author's Institution:**	Hong Kong Baptist University
**Corresponding Author's Secondary Institution:**	
**First Author:**	Lu Zhang
**First Author Secondary Information:**	
**Order of Authors:**	Lu Zhang
	Chao Yang
	Zhenmiao Zhang
	Yufen Huang
	Xuefeng Xie
	Herui Liao
	Jin Xiao
	Werner Pieter Veldsman
	Kejing Yin
	Xiaodong Fang
**Order of Authors Secondary Information:**	
**Response to Reviewers:**	Dear editors and reviewers,<br>Many thanks indeed for all your hard work on our manuscript, and thanks a lot for the constructive and valuable comments that providing us insights and inspirations. We have made careful revisions on the manuscript and supplementary data. All the comments from editors and reviewers have been addressed point by point as shown below, and corresponding corrections have been made in the revised manuscript. Kindly let us know if there's any further explanation needed.<br><br>Sincerely,<br>Lu Zhang<br>Department of Computer Science, Faculty of Science,<br>Hong Kong Baptist University, Hong Kong<br>E-mail: ericluzhang@hkbu.edu.hk<br>Phone: +852 3411 5880<br><br>Response to Editor and Reviewers<br>GIGA-D-23-00278R1<br>LRTK: A platform agnostic toolkit for linked-read analysis of both human genome and metagenome<br>Lu Zhang; Chao Yang; Zhenmiao Zhang; Yufen Huang; Xuefeng Xie; Herui Liao; Jin Xiao; Werner Pieter Veldsman; Kejing Yin; Xiaodong Fang<br>GigaScience<br><br>Dear Dr. Zhang,<br><br>Your revised manuscript "LRTK: A platform agnostic toolkit for linked-read analysis of both human genome and metagenome" (GIGA-D-23-00278R1) has been assessed again by reviewer #2. I am pleased to inform you that it is acceptable for publication in GigaScience, in principle, once you have carried out some minor follow-up revisions suggested by the reviewer (see the comments below).<br>Responses to Editor<br>Please also address the following editorial points in the revised manuscript:<br>1) Please structure your abstract (Background", "Findings", "Conclusions").<br>Response:<br>We have reorganized the abstract to align with the required structure.<br>Background: Linked-read sequencing technologies generate high-base quality short-

reads that contain extrapolative information on long-range DNA connectedness. These advantages of linked-read technologies are well-known and have been demonstrated in many human genomic and metagenomic studies. However, existing linked-read analysis pipelines (e.g., Long Ranger) were primarily developed to process sequencing data from the human genome and are not suited for analyzing metagenomic sequencing data. Moreover, linked-read analysis pipelines are typically limited to one specific sequencing platform.
Findings: To address these limitations, we present the Linked-Read ToolKit (LRTK), a unified and versatile toolkit for platform agnostic processing of linked-read sequencing data from both human genome and metagenome. LRTK provides functions to perform linked-read simulation, barcode sequencing error correction, barcode-aware read alignment and metagenome assembly, reconstruction of long DNA fragments, taxonomic classification and quantification, as well as barcode-assisted genomic variant calling and phasing. LRTK has the ability to process multiple samples automatically, and provides users with the option to generate reproducible reports during processing of raw sequencing data and at multiple checkpoints throughout downstream analysis. We applied LRTK on linked-reads from simulation, mock community and real datasets for both human genome and metagenome. We showcased LRTK's ability to generate comparative performance results from preceding benchmark studies and to report these results in publication-ready HTML document plots.
Conclusions: LRTK provides comprehensive and flexible modules along with an easy-to-use Python-based workflow for processing linked-read sequencing datasets, thereby filling the current gap in the field caused by platform-centric genome-specific linked-read data analysis tools.

2) Pleas add ORCIDs to the title page, where available. We have the following on our files: ORCID iDs: Lu Zhang [0000-0002-2794-7371]; Chao Yang [0000-0001-6518-4574]; Zhenmiao Zhang [0000-0003-3748-1664]; Yufen Huang [0000-0001-5939-1091]; Xuefeng Xie [0000-0001-6876-673X]; Herui Liao [0000-0001-8871-3483]; Jin Xiao [0000-0001-5059-1492]; Werner Pieter Veldsman [0000-0001-9837-8332]; Kejing Yin [0000-0003-4146-3338]; Xiaodong Fang [0000-0001-7061-3337]
Response:
We have included the ORCID of each author on the title page in the revised manuscript.
ORCID iDs
Chao Yang https://orcid.org/0000-0001-6518-4574
Zhenmiao Zhang https://orcid.org/0000-0003-3748-1664
Yufen Huang https://orcid.org/0000-0001-5939-1091
Xuefeng Xie https://orcid.org/0000-0001-6876-673X
Herui Liao https://orcid.org/0000-0001-8871-3483
Jin Xiao https://orcid.org/0000-0001-5059-1492
Werner Pieter Veldsman https://orcid.org/0000-0001-9837-8332
Kejing Yin https://orcid.org/0000-0003-4146-3338
Xiaodong Fang https://orcid.org/0000-0001-7061-3337
Lu Zhang https://orcid.org/0000-0002-2794-7371

3) Please apply for a biotools ID identifier, and include it in your manuscript, in the code availability section. (https://bio.tools/)
Response:
We have incorporated the biotools ID for LRTK in the code availability section.
"Biotools ID: biotools:lrtk "

4) Our data curators will contact you shortly to prepare the release of supporting data via our database GigaDB, if applicable. If a GigaDB set is prepared, please cite this from the data availability section.
Response:
We will include GigaDB set in our manuscript once it is ready.

Responses to Reviewer2:
Reviewer reports:

Reviewer #2: The authors have responded to all of the referees' comments and suggestions thoroughly. I have a few remaining suggestions and questions.

Powered by Editorial Manager® and ProduXion Manager® from Aries Systems Corporation

Documentation:

The purpose of multiple tool options:
By 'Its straightforward, easy to use and timesaving.', do you mean that FreeBayes is more computationally efficient than GATK?
Response:
In our study, FreeBayes took less running time than GATK to detect SNVs and INDELs from the alignment files. For NA12878, FreeBayes and GATK spend ~4 hours and 25 hours using 64 threads. This observation is consistent with a previous study [1]. We revised the manuscript as (from line 547 to line 549):
FreeBayes is a Bayesian genetic variant detection tool designed to identify SNVs, INDELs, multinucleotide polymorphisms, and more complex events, which is an easy-to-use and time-saving tool.

Provenance of ATCC-MSA-1003:
The sentence 'The sequencing data volumes are 37.7 Gb, 111 Gb and 55 Gb for...' contains information that already exists in Table S3 and does not need to be repeated.
Response:
Thank you for the comment and we have removed the descriptions from the manuscript.

Table S2: There is no 10x download link for NA24385, NA24143 or NA24149.
Response:
We have included 10x Genomics data download links for NA24385, NA24143 and NA24149 in Table S2.

NA24385
https://ftp-trace.ncbi.nlm.nih.gov/giab/ftp/data/AshkenazimTrio/HG002_NA24385_son/10Xgenomics_ChromiumGenome/NA24385.fastqs/
NA24143
https://ftp-trace.ncbi.nlm.nih.gov/giab/ftp/data/AshkenazimTrio/HG004_NA24143_mother/10Xgenomics_ChromiumGenome/NA24143.fastqs/
NA24149
https://ftp-trace.ncbi.nlm.nih.gov/giab/ftp/data/AshkenazimTrio/HG003_NA24149_father/10Xgenomics_ChromiumGenome/NA24149.fastqs/

Analysis:
Lines 166-169:
The paragraph starting 'We observed, for 10x Genomics, the peak barcode count is around 7...' should be included in the main text to explain the differences in the number of fragments per barcode observed when comparing Figure 2 A1 vs. B1.
Response:
Thank you for the suggestions. We have updated the related results in the revised manuscript from line 383 to line 401:

For human genome sequencing, the average NF/P values were approximately 7 for 10x Genomics linked-reads and were declined to around 2 and 4 for stLFR and TELL-Seq linked-reads, respectively (Figure 2B). For metagenomic sequencing, the average values were around 14 for 10x Genomics linked-reads, 1 for stLFR and 5 for TELL-Seq linked-reads (Figure 2A). These differences may be attributed to the barcoding approaches employed. In the case of 10x Genomics, barcoding takes place within water-in-oil droplets, which require a specialized instrument for droplet generation. The number of DNA fragments present in each droplet is influenced by the size of the DNA fragments. The DNA fragments from the human genome are long, resulting in fewer fragments being included in each droplet. The microbial DNA fragments are relatively shorter, allowing for more fragments to be accommodated within a droplet. In contrast, neither TELL-Seq nor stLFR utilizes droplets for barcoding. Instead, the barcoding reactions occur in an open environment and are partitioned using beads alone. Generally, each bead is conjugated with at least one unique barcode sequence on its surface and can capture 1-2 DNA fragments. For stLFR and TELL-Seq, the number of DNA fragments is not dependent on the length of the DNA fragments, resulting in a

Powered by Editorial Manager® and ProduXion Manager® from Aries Systems Corporation

	similar number of fragments per barcode for both human and metagenomic sequencing.<br><br>Lines 187-188:<br>'For FreeBayes and SAMtools, we further removed the SNVs if the total depths were less than 6, the number of reads supporting alternative allele was less than 2, and SNV qualities were below 15.'- was this included in the manuscript?<br>Response:<br>Thank you for the comments. We have included the descriptions in the Methods section from line 570 to line 574.<br><br>For FreeBayes and SAMtools, LRTK further removes the SNVs if the total depths are less than 6, the number of reads supporting alternative allele is less than 2, and SNV qualities are below 15. LRTK does not perform quality control for the SNVs called by inStrain as it does not output SNV quality scores.<br><br>'inStrain is the most sensitive tool to detect SNVs.'- Is sensitive the right word to use? Sensitive implies that most of SNVs detected are true positives. Is that necessarily true for inStrain?<br>Response:<br>Sorry for the confusion. We revised the sentence as “inStrain is able to detect many unique microbial SNVs” from line 243 in the revised manuscript.<br><br>Lines 192-193:<br>It seems that MAI implies a somatic copy number change in the maternal and paternal alleles of two haplotypes. Are you also implying that each sample should be treated as a diploid organism, and any time that only the reference or alternate allele appears alone in the sample, that it counts as a gain or loss? I'm still not sure why this term (especially with the word 'imbalance') is being used when it seems to be a cancer-specific phenomenon, and relative abundance change, or strain frequency change, or some other phrase is more applicable for metagenomics.<br>Response:<br>Thanks for the comment, we believe “strain frequency change” is more appropriate here. We have replaced “mirrored allelic imbalance / MAI” to “strain frequency change” in the revised manuscript.<br><br>Lines 201-208:<br>The line 'We observed that there was a consistent decrease in the number of fragments per barcode (N_{F/P}) for the stLFR and TELL-Seq technologies.' should be added to the main text.<br>Response:<br>Thanks for the comments. We have added the descriptions in the Discussion section from line 382 to line 383.<br><br>Our findings revealed that there was a consistent decrease of NF/P for stLFR and TELL-Seq technologies than those obtained from 10x Genomics.<br><br>Miscellaneous:<br><br>Lines 399-405:<br>It seems like you're using some kind of unsupervised clustering method to infer strains i.e.: SNVs with allele frequencies that seem to be correlated. There are many methods to infer bacterial strains- why weren't any of those used?<br>Response:<br>We agree that there are many microbial strain inference tools, such as StrainFinder, STRONG, MetaMaps. However, these tools do not consider the long-range connectedness provided by linked-reads. The challenge of developing a strain inference method tailored to linked-read sequencing is still unsolved. In LRTK, we employed an unsupervised clustering method to group SNVs with similar allele frequencies. The SNVs from the same cluster were considered to be from the same strain. This strategy has been successfully applied by Roodgar M, et al. on longitudinal 10x Genomics linked-read sequencing dataset [2].<br><br>Line 665:

	Does the 'B' in BAF (B allele frequency) refer to the alternate and/or minor allele? Please standardize your terminology.<br>Response:<br>Thank you for the comment. We have replaced “B allele frequency” with “minor allele frequency” in the revised manuscript.<br><br>Reference<br>1. Murillo GH, You N, Su X, Cui W, Reilly MP, Li M, et al.. MultiGeMS: detection of SNVs from multiple samples using model selection on high-throughput sequencing data. Bioinformatics. Oxford Academic; 2016; doi: 10.1093/BIOINFORMATICS/BTV753.<br>2. Roodgar M, Good BH, Garud NR, Martis S, Avula M, Zhou W, et al.. Longitudinal linked-read sequencing reveals ecological and evolutionary responses of a human gut microbiome during antibiotic treatment. Genome Res. Cold Spring Harbor Laboratory Press; 2021; doi: 10.1101/GR.265058.120/-/DC1.
**Additional Information:**	
**Question**	**Response**
Are you submitting this manuscript to a special series or article collection?	No
**Experimental design and statistics**<br><br>Full details of the experimental design and statistical methods used should be given in the Methods section, as detailed in our Minimum Standards Reporting Checklist. Information essential to interpreting the data presented should be made available in the figure legends.<br><br>Have you included all the information requested in your manuscript?	Yes
**Resources**<br><br>A description of all resources used, including antibodies, cell lines, animals and software tools, with enough information to allow them to be uniquely identified, should be included in the Methods section. Authors are strongly encouraged to cite Research Resource Identifiers (RRIDs) for antibodies, model organisms and tools, where possible.<br><br>Have you included the information requested as detailed in our Minimum Standards Reporting Checklist?	Yes

Powered by Editorial Manager® and ProduXion Manager® from Aries Systems Corporation

**Availability of data and materials**<br><br>All datasets and code on which the conclusions of the paper rely must be either included in your submission or deposited in publicly available repositories (where available and ethically appropriate), referencing such data using a unique identifier in the references and in the "Availability of Data and Materials" section of your manuscript.<br><br>Have you have met the above requirement as detailed in our Minimum Standards Reporting Checklist?	Yes

Manuscript
Click here to access/download;Manuscript;LRTK_for_production.docx

LRTK: A platform agnostic toolkit for linked-read analysis of both human genome and metagenome

Chao Yang[1†], Zhenmiao Zhang[1†], Yufen Huang[2,3], Xuefeng Xie[4], Herui Liao[5], Jin Xiao[1], Werner Pieter Veldsman[1], Kejing Yin[1], Xiaodong Fang[3,4*], Lu Zhang[1,6*]

[1]Department of Computer Science, Hong Kong Baptist University, Hong Kong SAR, Hong Kong

[2]BGI Research, Shenzhen 518083, China

[3]BGI Genomics, Shenzhen 518083, China

[4]BGI Research, Sanya 572025, China

[5]Department of Electrical Engineering, City University of Hong Kong, Hong Kong SAR, Hong Kong

[6]Institute for Research and Continuing Education, Hong Kong Baptist University, China

[†]These authors contributed equally to this work.

*To whom correspondence should be addressed: E-mail:

fangxd@genomics.cn ,

ericluzhang@hkbu.edu.hk.

ORCID iDs

Chao Yang https://orcid.org/0000-0001-6518-4574

Zhenmiao Zhang https://orcid.org/0000-0003-3748-1664

Yufen Huang https://orcid.org/0000-0001-5939-1091

Xuefeng Xie https://orcid.org/0000-0001-6876-673X

Herui Liao https://orcid.org/0000-0001-8871-3483

Jin Xiao https://orcid.org/0000-0001-5059-1492

Werner Pieter Veldsman https://orcid.org/0000-0001-9837-8332

Kejing Yin https://orcid.org/0000-0003-4146-3338

Xiaodong Fang https://orcid.org/0000-0001-7061-3337

Lu Zhang https://orcid.org/0000-0002-2794-7371

Abstract

Background: Linked-read sequencing technologies generate high-base quality short-reads that contain extrapolative information on long-range DNA connectedness. These advantages of linked-read technologies are well-known and have been demonstrated in many human genomic and metagenomic studies. However, existing linked-read analysis pipelines (e.g., Long Ranger) were primarily developed to process sequencing data from the human genome and are not suited for analyzing metagenomic sequencing data. Moreover, linked-read analysis pipelines are typically limited to one specific sequencing platform.

Findings: To address these limitations, we present the Linked-Read ToolKit (LRTK), a unified and versatile toolkit for platform agnostic processing of linked-read sequencing data from both human genome and metagenome. LRTK provides functions to perform linked-read simulation, barcode sequencing error correction, barcode-aware read alignment and metagenome assembly, reconstruction of long DNA fragments, taxonomic classification and quantification, as well as barcode-assisted genomic variant calling and phasing. LRTK has the ability to process multiple samples automatically, and provides users with the option to generate reproducible reports during processing of raw sequencing data and at multiple checkpoints throughout downstream analysis. We applied LRTK on linked-reads from simulation, mock community and real datasets for both human genome and metagenome. We showcased LRTK's ability to generate comparative performance

results from preceding benchmark studies and to report these results in publication-ready HTML document plots.

Conclusions: LRTK provides comprehensive and flexible modules along with an easy-to-use Python-based workflow for processing linked-read sequencing datasets, thereby filling the current gap in the field caused by platform-centric genome-specific linked-read data analysis tools.

Keywords: linked-read sequencing, 10x Genomics, TELL-Seq, stLFR, metagenome, human genome

Introduction

Linked-read sequencing generates short-reads with high base quality and extrapolative information on long-range DNA connectedness, which has led to significant advancements in human genome and metagenome research [1–4]. It circumvents the typical lack of long-range DNA information in short-read sequencing, and the high error rates and large initial DNA load requirements of long-read sequencing (e.g., Oxford Nanopore and Pacific Bioscience). These advantages of linked-read sequencing are invaluable when dealing with challenging cases of low-input clinical samples, such as cancer tissues or infectious disease samples. Linked-read technology furthermore promotes haplotype construction and the detection of complex structural variations [5], and its relatively low cost enables the application in large cohort studies.

Linked-read sequencing platforms, such as 10x Genomics linked-read (10x Genomics; now discontinued) and the newly developed single-tube long fragment read (stLFR) [6] and transposase enzyme-linked long-read sequencing (TELL-Seq) [7], hold much promise in the metagenomics area. The hidden long-range information they provide enables local assembly of co-barcoded reads and thus significantly increases the number of high-quality metagenome-assembled genomes (MAGs) [8]. In longitudinal sequencing datasets [3,9,10], the barcodes associated with linked-read promote the phasing of genomic variants and refine the identification of intra-host evolution of gut microbiota. In some complex environments, such as soil, linked-read sequencing has been shown to aid in the investigation of the involved microbial

genomes [11,12]. However, existing linked-read pipelines are mainly designed for use with the human genome, which points to an urgent need for appropriate metagenome analysis toolkits.

Despite the limitations that genome specificity places on research scope, linked-read sequencing has already been successfully applied to many human genomic studies [2,13–15]. Some toolkits have been developed to facilitate the investigation of the human genome. For example, Long Ranger [16] performs barcode-aware read alignment and implements modules for genomic variant calling and phasing using 10x Genomics linked-reads. Tell-Sort [7] is a Docker-based pipeline to process TELL-Seq linked-reads, for genomic variants detection and phasing. stLFR has found application in a customized pipeline that has been developed to first convert its raw reads into a 10x-compatible format, after which Long Ranger is applied for downstream analysis. This pipeline, however, typically requires a lot of random-access memory and its data format conversion procedure is time-consuming. In addition, the format conversion may induce the loss of barcode specificity as the type of barcodes decreases dramatically. This occurs when the stLFR and TELL-Seq linked-reads are typically converted into a 10x-compatible format to run Long Ranger and Supernova (https://github.com/BGI-Qingdao/stlfr2supernova_pipeline and https://sagescience.com/wp-content/uploads/2020/10/TELL-Seq-Software-Roadmap-User-Guide-2.pdf). Beyond the preceding examples that validate the inherent usefulness of linked-read technologies, our search of the literature furthermore

revealed a lack of unified and open-source toolkits that are compatible with the different linked-read platforms.

To this end, we present Linked-Read ToolKit (LRTK), a unified and versatile toolkit to analyze both metagenomic and human genome linked-read sequencing data derived from any of the three major linked-read sequencing platforms. LRTK delivers a suite of utilities to simulate linked-read sequencing data, barcode sequencing error correction, barcode-aware alignment and metagenome assembly, reconstruction of long DNA fragments, and genomic variant detection and phasing. LRTK is open-source, automatically produces HTML reports to summarize quality statistics as part of its pipeline, and generates publication-ready visualizations. We applied LRTK to linked-reads from simulation, mock community and real datasets to evaluate the performance of different technologies and demonstrate the potential applications of LRTK. Our results show that LRTK performs favorably on human genome linked-read sequencing data when compared to the pipelines designed specifically for a single platform and that it adequately allows for data analysis of metagenomic sequencing data.

Results

Overview of LRTK

We developed Linked-Read ToolKit (which we refer to as LRTK) that takes raw linked-reads from 10x Genomics, stLFR, or TELL-Seq technologies, and analyzes these inputs in a multi-step checkpointed pipeline that ends with the generation of user-friendly

reports. LRTK consists of two main sections to process metagenomic and human genome linked-read sequencing data from mainstream technologies **(Figure 1)**. For metagenomic sequencing, LRTK includes the representative human gut microbial genomes from the UHGG [17] project as its default reference genomes. We modified EMA [18] to perform barcode-aware alignment to be compatible with different platforms. To further reduce spurious mapping errors, LRTK eliminates the genomes if their coverage is below 40%. It performs genomic variation calling and phasing for these candidate genomes if the single nucleotide variant (SNV) calling function is enabled. In addition, LRTK is equipped with the functions to perform barcode-aware metagenome assembly and reconstruct MAGs from the metagenomic linked-read sequencing data.

In the human genome processing section, LRTK directly aligns linked-reads to the human reference genome using the same modified EMA followed by marking PCR duplicates for each barcode. LRTK reconstructs long DNA fragments through greedy extension based on the alignment coordinates of co-barcoded linked-reads [19]. After reads alignment, LRTK offers users an option to select one of the well-known tools for variant calling, including FreeBayes [20], SAMtools [21], GATK [22] for SNV and small INDEL (Insertion and Deletion, < 50 bps) calls; and Aquila [23], LinkedSV [24] and VALOR2 [25] for structural variant calls (SVs) (>50 bps). For variant phasing, LRTK utilizes HapCUT2 [26] and WhatsHap [27] to explore phasing blocks for SNVs and small INDELs **(Table S1)**. Further details are available in the **Methods** section.

Data Description

We incorporated one simulated dataset (S1), one dataset from a mock community (B1), and two real datasets from human gut microbiomes (D1 and D2) to evaluate the metagenomic sequencing analysis module of LRTK **(Table S2)**. For dataset S1**,** we simulated 11.9 Gb and 9.2 Gb linked-reads from 10x Genomics and stLFR for 40 complete bacterial genomes from NCBI RefSeq database, with lognormal abundance distribution (**Methods**), respectively **(Table S3)**. The dataset B1 was generated from ATCC-MSA-1003, containing 20 bacterial species with abundances varying from 0.02% to 18% **(Table S4).** For the two real metagenomic datasets, D1 consists of 16 longitudinal human gut metagenomic sequencing datasets from a single individual, sequenced with an average of 24 Gb linked-reads on 10x Genomics platform [3]. The other dataset D2 contains around 99 Gb stLFR linked-reads from human gut metagenome [28]. For the human genome analysis section, we collected the linked-read sequencing datasets from NA12878, NA24143, NA24149 and NA24385 (**Table S2).** In addition, we performed linked-reads down-sampling to ensure fair comparisons among the three sequencing technologies. We down-sampled around 20 Gb metagenomic linked reads from ATCC-MSA-1003 for each platform. Similarly, around 110 Gb (~35X) linked reads from NA12878 were extracted for the three technologies **(Table S3)**.

LRTK supports multiple linked-read sequencing technologies

LRTK can handle linked-reads from various sequencing technologies, including but not limited to 10x Genomics, stLFR, or TELL-Seq. Initially, LRTK converts the raw linked-reads into a unified FASTQ format, which contains a new field "BX:Z:" to include 16 bps (10x Genomics linked-read), 18 bps (TELL-Seq) and 30 bps (stLFR) barcode sequences **(Figure S1)**.

Sequencing errors are often enriched at the start or end of linked-reads, where barcode sequences are typically found. LRTK includes functions to correct potential sequencing errors in barcodes. After error correction, there are approximately 94.8% and 94.1% of barcode sequences on the whitelist of 10x Genomics linked-reads for NA12878 and ATCC-MSA-1003 **(Table S2)**, respectively. The performance is comparable to the results obtained from Long Ranger (94.4% for NA12878 and 93.6% for ATCC-MSA-1003). The corresponding rates are slightly lower for stLFR linked-read (85.4% for NA12878 and 90.7% for ATCC-MSA-1003) compared to 10x Genomics linked-read. As there is no whitelist for TELL-Seq, we could not perform the analysis for NA12878 and ATCC-MSA-1003 TELL-Seq linked-read sequencing data.

LRTK reconstructs long DNA fragments by barcode deconvolution

The quality of DNA sequencing library may significantly affect the performance of metagenome assembly [29], human genome assembly [19] and structural variant calling

[30]. To evaluate the quality of linked-read sequencing libraries, we reconstructed the input long DNA fragments for both human genome and metagenomic sequencing data based on co-barcoded read alignments (**Methods**). We also calculated several key statistics to comprehensively compare the libraries from different linked-read sequencing technologies [19]. The statistics include average coverage of short-reads per fragment (C_R), average physical coverage of the genome by long DNA fragments (C_F), number of fragments per partition/beads ($N_{F/P}$), unweighted and length-weighted average DNA fragment length (μ_{FL} and $W\mu_{FL}$) **(Figure S2)**.

For NA12878 (**Table S3**), LRTK detected approximately 7.34, 1.95 and 3.60 fragments per barcode and achieved μ_{FL} of 45.9 kb, 16.46 kb and 54.78 kb for the libraries from 10x Genomics, stLFR and TELL-Seq, respectively **(Figure 2B)**. For ATCC-MSA-1003 (**Table S3**), stLFR linked-reads yielded the lowest $N_{F/P}$ ($N_{F/P}$ =1.48), while TELL-Seq linked-reads yielded a slightly higher number ($N_{F/P}$ =4.59). Both values were much lower than that obtained from 10x Genomics ($N_{F/P}$ =13.6) **(Figure 2A)**, indicating that stLFR and TELL-Seq have superior performance in deconvolving linked-reads from different species.

LRTK enables metagenome taxonomic quantification and genomic variant detection using barcode-aware alignment

Previous studies have amply demonstrated the huge potential of linked-reads in metagenomic studies [3,8,9,11]. Here, we evaluated the performance of linked-reads in detecting taxonomic abundance and genomic variants using LRTK. In LRTK, we

developed a computational pipeline to detect and quantify microbes using microbial reference genomes. LRTK can better identify the involved microbes based on their genome coverage (F1=0.81) than the existing k-mer based tools: Bracken [31] (F1=0.13), and KMCP [32] (F1=0.67); and marker gene based tools: MetaPhlAn2 [33] (F1=0.49), and MIDAS 2 [34] (F1=0.72), on the simulated stLFR linked-reads (**Figure 3A**). For the stLFR linked-reads from ATCC-MSA-1003, LRTK also demonstrated a superior performance (F1=0.78) than Bracken (F1=0.22), KMCP (F1=0.59) and MIDAS 2 (F1=0.6), but was inferior to MetaPhlAn 2 (F1: 0.95). We also evaluated the LRTK performance of taxonomic quantification by comparing the benchmark microbial abundance (from simulation and ATCC-MSA-1003) and the predicted values using Spearman correlation coefficient (SCC). For the simulated dataset, LRTK (SCC=0.99), MIDAS 2 (SCC=0.98), and KMCP (SCC=0.95) exhibited superior performance. For stLFR linked-reads in ATCC-MSA-1003, LRTK (SCC=0.97), Bracken (SCC=0.97), and MetaPhlAn 2 (SCC=0.97) were the top performers. Comparable findings were also observed in the linked-reads data generated from other technologies in simulation **(Figure S3A)** and ATCC-MSA-1003 **(Figure S3B and S3C).** In addition, LRTK also enables the identification of microbial SNVs and could reconstruct their potential haplotypes based on co-barcoded linked-reads. Comparing different SNV callers implemented in LRTK, we discovered that approximately 176,891 SNVs were jointly detected by FreeBayes [17], SAMtools [18] and inStrain [35] on stLFR linked-reads for ATCC-MSA-1003 **(Figure 3B).** These SNVs account for around 76%, 80% and 21% of the total SNVs

detected by FreeBayes and SAMtools and inStrain, respectively **(Figure 3B)**. More than 50% of SNVs from inStrain can not be detected by FreeBayes and SAMtools, suggesting inStrain is able to detect many unique microbial SNVs. We further applied LRTK to explore the taxonomic composition and genomic variants for each sample from a longitudinal linked-read metagenomic dataset **(D1, Table S2)** [3]. Using multiple related samples, LRTK identified a strain frequency change **(Methods)** of the species Alistipes finegoldii: the minor alleles of some SNVs in some time points became the major alleles in other time points, which may suggest intra-host strain evolution over time **(Figure 3C and D)**.

LRTK promotes metagenome assembly using linked-reads with high barcode specificity

In our previous study, we have shown metagenome assembly on linked-reads could improve assembly length and the number of near-complete MAGs[28]. We compared the performance of two well-known short-read assemblers: MEGAHIT [36] and metaSPAdes [37], and three linked-read assemblers: Athena [38], CloudSPAdes [39] and Pangaea [28] using the linked-reads from ATCC-MSA-1003 and simulation **(Table S3).** Among them, LRTK (Pangaea module) achieves the highest NA50 values for stLFR (NA50=372kb) and TELL-Seq (NA50=339kb) linked-reads, respectively **(Figure 4A)**. As Pangaea is not compatible with 10x Genomics linked-reads, Athena becomes the best tool on 10x Genomics in terms of NA50 (Athena:146 kb; cloudSPAdes: 45 kb; metaSPAdes: 17 kb and MEGAHIT:79 kb). We also examined

the assembly quality of each species in ATCC-MSA-1003, and observed that Pangaea always obtained the highest NA50 and N50 values for stLFR and TELL-Seq linked-reads while the assembly length is comparable **(Figure S4A)**. For simulated linked-reads, Pangaea and Athena also show superior performances than the other metagenome assemblers (**Figure S4B and Figure S5**). We applied LRTK to a human gut metagenomic dataset (D2) [28] and found two contigs were circularized, which showed near perfect collinearity with the closest reference genomes **(Figure 4B)**. LRTK could also automatically perform contig binning using MetaBAT 2 [40] after metagenome assembly. In the D2 data set, LRTK recovers 24 near-complete, 7 high-quality and 52 medium-quality bins for D2 **(Methods, Figure 4C-E)**. The superior assembly performance we have observed affirms the efficacy of linked-read sequencing technologies on metagenome assembly.

LRTK provides best practices for human genomic variant detection and phasing

Previous studies have shown that genomic variant detection [41] and phasing could benefit from the high base quality and long DNA fragments provided by linked-read[42]. Here, we benchmarked the computational tools for human genomic variant detection and phasing and demonstrated a best practice guideline for human genome linked-read analysis using LRTK. We applied the modified version of EMA to align linked-reads from different sequencing technologies to the human reference genome (GRCh38). We first benchmarked the commonly used tools FreeBayes [20], GATK

[22] and SAMtools [21] to detect SNVs and small INDELs. Among them, GATK (F1=0.90) achieves the best average F1 score across the three technologies in detecting SNVs, followed by SAMtools (F1=0.89) and FreeBayes (F1=0.87) **(Figure 5A)**. For small INDEL calling, SAMtools demonstrated the best average F1 score (SAMtools: F1= 0.71; GATK: F1= 0.65; FreeBayes: F1 = 0.59) while GATK has a better recall value (SAMtools: average recall = 0.87; GATK: average recall = 0.91; FreeBayes: average recall = 0.79) (**Figure 5B)**. We then compared the linked-read phasing tools, HapCUT2 [26] and WhatsHap [27], for genomic variant phasing. We observed that HapCUT2 **(Figure 5C and D)** achieved longer average length of phasing blocks (HapCUT2: 23.2 Mb and WhatsHap: 0.4 Mb) and a higher average phased heterozygous SNV rate compared to WhatsHap (HapCUT2: 0.99 and WhatsHap: 0.63). We only evaluated the performance of SV detection tools on 10x Genomics linked-reads of NA24385 because some of the tools do not support stLFR and TELL-Seq. As the recall values illustrated in **Figure 5F**, Aquila has a higher recall value for 50 bps – 1 kb deletions (Aquila: 0.82; LinkedSV: 0.54; Long Ranger: 0.43). LinkedSV [24] and Long Ranger perform better in detecting deletions longer than 1 kb (Aquila: 0.29; LinkedSV: 0.71; Long Ranger: 0.72). Aquila also shows a better performance than Pamir [43] and PopIns2 [44] in detecting insertions (Aquila: 0.35; Pamir: 0.07; PopIns2: 0.01) (**Figure 5G**). We also applied LRTK to linked-read from a trio (D3; Father: NA24149; Mother: NA24143; and Son: NA24385) and found it demonstrated excellent variant phasing performance and identity-by-descent segment detection in pairwise samples **(Figure 5E)**.

LRTK provides flexible commands to process sequencing data

A primary advantage of LRTK is its flexible, user-defined settings for different tasks. Users have a choice to run each LRTK module independently and generate separate results for each module. For instance, the MKFQ function could be independently used to simulate linked- reads from 10x Genomics and stLFR platforms. For some functions, LRTK provides multiple tools for users to choose from. For microbial SNVs detection, LRTK offers users a choice between SAMtools [21], FreeBayes [20], and inStrain [35]. To accommodate user-specific requirements on LRTK command, LRTK also allows users to set different parameters and save the results for further comparison.

LRTK provides automated analysis and user-friendly reports

LRTK provides an automated analysis pipeline, starting from raw linked-reads to performing diverse data analysis and generating publication-ready visualizations. Specifically, LRTK investigates different types of data features, calculates their corresponding statistical indicators, and presents them together in an HTML report. Taking the aforementioned longitudinal linked-read sequencing dataset D1 as an example, LRTK generates a systematic summary of the input sequencing libraries and analysis outcomes of each step. Firstly, LRTK produces summaries of the FASTQ files obtained from the read quality control tools **(Figure S6A-B)**. After aligning reads to the reference genomes, LRTK calculates the key parameters for the sequencing libraries and reconstructs long DNA fragments **(Figure S6C-D)**. For

microbial species and genomic variants, LRTK generates basic statistics and presents them using concise distribution plots **(Figure S6E-F).** For downstream analysis, LRTK conducts principal components analysis on the relative abundance profiles from multiple samples and performs clustering analysis on allele frequencies of microbial SNVs **(Figure S6G)**. Similar reports could be produced for human genome analysis using LRTK **(Figure S7)**.

Evaluation of the computational resources required for LRTK

We would focus on evaluating computational resources required by LRTK for linked-read preprocessing and alignment. The computational resources needed for genomic variants calling and phasing depend on the chosen software, while metagenome sequencing data typically demand fewer computational resources due to the lower volume of required sequencing reads. We extracted approximately the same data volume (around 35X, 110G bases) of linked-reads from NA12878 for 10x Genomics, stLFR and TELL-Seq and aligned these reads to the human reference genomes. As shown in **Figure S8**, LRTK required around 26.3, 37.6 and 19.8 hours to align reads from the three platforms with a maximum memory usage of around 74Gb using 64 threads. In comparison to Long Range, LRTK reduced memory requirements (maximum memory for Long Ranger: >100 Gb) at the expense of increased running time. The experiments were carried out on the computational nodes with Xeon Gold 6330 @ 2.0GHz (2S/28C) / 1T RAM / 900GB SWAP.

Discussion

Seeing that multiple linked-read sequencing technologies are extensively utilized in scientific studies, a platform agnostic linked-read processing tool would be an intuitive solution to ensure reproducibility and robustness. Unfortunately, a cross-platform software solution is currently unavailable to the research community. Accordingly, we introduce LRTK, a unified and versatile computational framework to efficiently process sequencing data from 10x Genomics, stLFR and TELL-Seq technologies. LRTK includes separately invokable commands to perform linked-read simulation, barcode sequencing error correction, barcode-aware alignment and metagenome assembly, reconstruction of long DNA fragments and other barcode-assisted genomic variant calling and phasing. LRTK also provides automated and complete analysis, from raw data QC through advanced downstream analysis to generation of publication-ready visualization. LRTK is also open source and allows easy integration with other scientific pipelines.

Short-read sequencing has led to significant discoveries in large-scale population sequencing studies within the human genome and metagenome fields [45,46]. However, the limited sequencing length poses challenges for tasks like complex structural variant detection in the human genome and ribosomal RNAs assembly from metagenomic data. Long-read sequencing, such as single-molecule real-time sequencing by Pacific Biosciences (PacBio) and nanopore sequencing by Oxford Nanopore Technologies (ONT), are gaining attention for their improved performance in addressing these challenges. Despite their advantages, long-read sequencing can be

costly for large-cohort studies. Linked-read sequencing technologies offer a cost-effective solution for large population studies by attaching barcodes to short-reads to establish long-range DNA connectedness. Previous studies have shown 10x Genomics linked-reads have facilitated the discovery of complex structural variants, such as chromothripsis [5], large rearrangements [47] and tandem duplications [14], in cancer studies. Additionally, 10x Genomics linked-reads have enhanced metagenome assembly contiguity [38] and enabled haplotype construction from time-series metagenomic data [3]. The newly emerging stLFR and TELL-Seq have further improved barcode specificity aiming for one fragment per barcode. These advancements have shown superior performance in distinguishing linked-reads from different species [28]. Our study benchmarks tools developed for linked-read sequencing and integrates them into LRTK to support academic applications. Furthermore, LRTK is the only tool that can accept linked-reads from all three platforms and avoid loss of barcode specificity.

Our finding revealed that there was a consistent decrease of $N_{F/P}$ for stLFR and TELL-Seq technologies than those obtained from 10x Genomics. For human genome sequencing, the average $N_{F/P}$ values were approximately 7 for 10x Genomics linked-reads and were declined to around 2 and 4 for stLFR and TELL-Seq sequencing, respectively **(Figure 2B)**. For metagenomic sequencing, the average values were around 14 for 10x Genomics linked-reads, 1 for stLFR and 5 for TELL-Seq sequencing **(Figure 2A)**. These differences may be attributed to the barcoding approaches employed. In the case of 10x Genomics, barcoding takes place within

water-in-oil droplets, which require a specialized instrument for droplet generation. The number of DNA fragments present in each droplet is influenced by the size of the DNA fragments. The DNA fragments from human genome are long, resulting in fewer fragments being included in each droplet. The microbial DNA fragments are relatively shorter, allowing for more fragments to be accommodated within a droplet. In contrast, neither TELL-Seq nor stLFR utilizes droplets for barcoding. Instead, the barcoding reactions occur in an open environment and are partitioned using beads alone. Generally, each bead is conjugated with at least one unique barcode sequence on its surface and can capture 1-2 DNA fragments. For stLFR and TELL-Seq, the number of DNA fragments is not dependent on the length of the DNA fragments, resulting in a similar number of fragments per barcode for both human and metagenomic sequencing. According to Bishara, Alex, et al. [38], assembly using 10x Genomics linked-reads often struggles with high-copy genomic repeat regions due to the many long fragments per barcode. The long-fragment barcoding approaches adopted by stLFR and TELL-seq have notably reduced the number of long fragments per barcode, which may improve the assembly performance of microbial genomes containing high-copy repeats. In our previous study, we demonstrated that the characteristics of the reconstructed long DNA fragments (E.g. C_R, C_F et al.) can significantly affect human genome assembly [19] and structural variant calling [30]. The improved stLFR and TELL-Seq may also further refine the structural variants calling and variant phasing using the barcode specificity. We anticipate that future

linked-read sequencing technologies will improve both DNA-extraction techniques and long-fragment barcoding approaches to achieve one fragment per barcode.

Finally, it is worth mentioning that LRTK has the potential to be extended to handle other types of linked-read sequencing technologies. For example, in 2017, Illumina introduced the bead-based barcode partitioning in a single tube to phase human genomes. It further proposed the complete long-read technology for complex genomes in 2022 [48]. Additionally, Meier J I, *et al.* developed Haplotype tagging to investigate the butterfly species [49]. Redin D, *et al.* recently introduced a novel library preparation method for high throughput barcoding of short reads [50]. The new single-cell metagenomic sequencing technologies employ highly accurate barcoded reads and provide inferable long-range information, which could potentially be used in combination with current linked-read technology in future studies [51]. We are actively developing LRTK to incorporate these technologies.

Methods

Data collection

We included one simulated metagenomic dataset (S1), one mock microbial community (B1) and two human gut metagenomic sequencing datasets (D1, D2) to evaluate the performance of the metagenomic data analysis section of LRTK **(Table S2)**. For S1, we simulated 11.8 Gb 10x Genomics and 9.2 Gb stLFR linked-reads using LRTK-SIM [29] for 40 complete bacterial genomes extracted from the NCBI RefSeq database (December 2023) with the same abundances (Log Normal

Distribution) **(Table S5)**. LRTK-SIM allows flexible parameters, such as C_F, C_R, $N_{F/P}$, μ_{FL} and $W\mu_{FL}$, to simulate linked-read data. We set the parameters, "C_F =500, C_R =0.2, $N_{F/P}$ =16, μ_{FL} =20" to simulate 10x Genomics linked-reads while "C_F =500, C_R =0.2, $N_{F/P}$ =1, μ_{FL} =20" to simulate stLFR linked-reads. The dataset B1 contains linked-reads from a mock microbial community ATCC-MSA-1003, from three different platforms (SRR12283286 for 10x Genomics, and PRJNA875547 for stLFR and TELL-Seq). The ATCC-MSA-1003 mock community is composed of 20 bacterial species represented at staggered abundances—specifically, five species at 18%, 1.8%, 0.18%, and 0.02% abundance levels, respectively. The complete descriptions of the mock metagenomic sample, including the genome sizes, individual bacterial species, and their corresponding reference sequence accessions, have been included in **Table S4.** The D1 contains 16 longitudinal human gut metagenomic 10x Genomics linked-read sequencing datasets from one single individual (accession number: SRP323279) **(Table S2)**. The D2 was downloaded from the China National GeneBank (CNGB) under project CNP0003432. It contains around 99 Gb stLFR human gut metagenomic sequencing data. In the human genome section, we collected the available linked-read sequencing data for the three technologies from NA12878, NA24143, NA24149 and NA24385. The detailed information has been included in **Table S2**. For ATCC-MSA-1003 and NA12878, we further performed linked-read down-sampling at the barcode level by using in-house scripts.

Data preprocessing

LRTK converts the raw linked-reads from 10x Genomics, stLFR and TELL-Seq into a unified FASTQ format **(Figure S1)** and corrects potential sequencing errors in barcodes. For 10x Genomics and stLFR linked-read, the barcodes are aligned to their respective barcode whitelists using the “BWA aln” command (BWA, RRID: SCR_010910). The barcodes with fewer than 2 mismatches in alignments are then corrected as the corresponding barcodes in the whitelist. LRTK adopts the approach described by Chen *et al.* [7] to correct barcode errors for TELL-Seq due to the lack of barcode whitelist. In general, LRTK tallies the supporting reads for each barcode derived from TELL-Seq linked-reads and distinguishes between barcodes with a single supporting read and those with multiple supporting reads. It then corrects possible sequencing errors in barcodes that initially had one mismatch by comparing them to those with multiple supporting reads. The linked-reads in the unified FASTQ file are then provided to fastp (fastp, RRID: SCR_016962) [52] to remove adapter sequences and low-quality reads. For metagenomic sequencing data, the sequencing reads are aligned to the human genome first and only unmapped reads are used for subsequent analysis.

Metagenome assembly and contig binning

We evaluated the performance of five metagenome assemblers on linked-reads: Athena (Athena, RRID: SCR_008110) [38], Pangaea [28], cloudSPAdes [39], MEGAHIT (MEGAHIT, RRID: SCR_018551) [36] and metaSPAdes [37], and

observed superior performance of Pangaea on stLFR and TELL-Seq sequencing data. Therefore, for LRTK, we chose Pangaea as the default assembler to assemble linked-reads metagenomic sequencing data for LRTK. After the initial assembly, LRTK extracts the circular contigs that are at least 1 Mb in length. The uncircularized contigs are grouped into MAGs using MetaBAT 2 (MetaBAT, RRID: SCR_019134) [40]. According to standard criteria of the minimum information about MAGs [53], they could be classified into near-complete (completeness > 90%, contamination < 5%, and could be detected 5S, 16S, and 23S rRNAs, and at least 18 tRNAs), high-quality (completeness > 90%, and contamination < 5%), medium-quality (completeness ≥ 50%, and contamination < 10%), and low-quality (the other MAGs).

Barcode-aware read alignment

For human genome sequencing data, LRTK utilizes EMA, a barcode-aware alignment approach, to map high-quality 10x Genomics linked-reads to the human reference genome [54]. We further modified EMA to be compatible with the barcodes from stLFR (30 bps) and TELL-Seq (18 bps). LRTK marks PCR duplicates for each barcode using the “BARCODE_TAG” parameter in Picard (https://broadinstitute.github.io/picard/) (Picard, RRID: SCR_006525). The alignment files are then sorted according to the genomic coordinates of alignments for further analysis.

For metagenomic sequencing data, we developed a tiered alignment approach to align the linked-reads to microbial genomes using the aforementioned modified EMA.

The default reference genomes for the human gut metagenome were downloaded from UHGG [17]. For non-gut samples, we used the GTDB [55] as the reference database, but users could also incorporate their own custom database.

In the first round of alignment, linked-reads were directly mapped to the 4,724 representative genomes in UHGG (by default). For each genome, LRTK calculates the coverage rate and covered base, and then extracts genomes with "coverage rate > 40% and covered base > 500 kb". These genomes will be used as the candidate reference genomes for the second round of alignment.

In the second round of alignment, linked reads are only mapped to those candidate reference genomes to reduce multiple alignment errors. For each candidate genome, LRTK partitions it into 10 kb windows and calculates the number of mapped reads on each window. The mapped reads are categorized into two types: unique-mapped reads (U) and multi-mapped reads (M), which will be processed separately. LRTK uses the formulas below to determine the total read count of a window (RC(W)), unique-mapped read count ($RC(U)$) and multi-mapped read count ($RC(M)$), where l is the window size.

$$RC(W) = RC(U) + RC(M)$$

$$RC(U) = \frac{U}{l}$$

$$RC(M) = \sum_{i=1}^{M} \omega_i * \{M\}/l$$

A coefficient ω is introduced for multi-mapped reads. When a multi-mapped in M is aligned to N different genomes, ω is calculated using the following formula:

$$\omega = U / \sum_{i=1}^{N} RC(U)$$

LRTK removes the windows with an extreme number of reads, and calculates the average depth using the remaining windows as the depth of the corresponding genome. The relative abundance is then calculated by aggregating the average depth of all identified genomes.

In the experiments, we further evaluated the performance (F1 score, precision and recall) of LRTK, KMCP [32], and MIDAS 2 [34] to identify microbes and quantify their abundances. The reference databases were prepared using the genome sequences from the GTDB database for the three tools. We also compared LRTK with the widely used taxonomic classification tools: Bracken [31] and MetaPhlAn 2 (MetaPhlAn, RRID: SCR_004915) [33] using their default databases.

Reconstruction of long DNA fragments

Initially, LRTK extracts unique-mapped co-barcoded paired-end short-reads from the alignment file to evaluate the distribution of insert sizes (with a mean of μ_{PE} and a standard deviation of σ_{PE}). Alignments are removed if the distance between two reads in a paired-end read (R1 and R2) exceeds a certain threshold, that is,

$$Dis(\mathrm{R1}, \mathrm{R2}) > \mu_{PE} + 3 * \sigma_{PE}.$$

The remaining paired-end reads are used as seeds and extended to both directions to connect with other seeds sharing the same barcode until no more eligible seeds can be found within a specified distance (200 kb by default). All of these co-barcoded reads are considered to be derived from the same long DNA fragment.

Identify and phase genomic variants

LRTK supports multiple popular variant detection tools and adheres to best practices for linked-read sequencing. For human genome sequencing data, LRTK provides FreeBayes (FreeBayes, RRID: SCR_010761) [20], SAMtools (SAMtools, RRID: SCR_002105) [21] and GATK (GATK, RRID: SCR_001876) [22] to call SNVs and small INDELs. FreeBayes is a Bayesian genetic variant detection tool designed to identify SNVs, INDELs, multinucleotide polymorphisms, and more complex events, which is an easy-to-use and time-saving tool [56]. GATK, while also employing a Bayesian framework, enhances its detection capabilities for insertions and deletions through specialized techniques such as read realignment and base recalibration. Although these steps add value, they also increase GATK's computational runtime. In contrast, SAMtools uses a hidden Markov model for the identification of small variants and has demonstrated robust performance across various studies. The available phasing tools include HapCUT2 (HapCUT, RRID: SCR_010791) [26] and WhatsHap [27]. HapCUT2 demonstrates excellent performance in phasing

heterozygous SNVs within a diploid context, such as the human genome. WhatsHap, however, introduces an innovative clustering and threading approach that delivers precise phasing in polyploid genomes. Having obtained the phased SNVs, we used PhaseME [57] to calculate the phasing block and phasing rate to assess the phasing quality. Subsequently, we used hap-ibd [58] to detect pairwise identity-by-descent segments across multiple samples. By default, large SVs are identified using Aquila[23]. However, Users have the flexibility to select other tools such as LinkedSV [24] and VALOR2 [25] for SV analysis. LinkedSV leverages barcode overlapping, read depth, paired-end signals and local assembly to detect deletions, although it currently lacks support for insertion detection. In contrast, the assembly-based tool Aquila is capable of detecting both insertions and deletions, offering a comprehensive solution.

For metagenome sequencing data, LRTK offers three metagenomic SNV callers: FreeBayes [20], inStrain [35] and SAMtools [21]. For FreeBayes and SAMtools, LRTK further removes the SNVs if the total depths are less than 6, the number of reads supporting the alternative allele is less than 2, and SNV qualities are below 15. LRTK does not perform quality control for the SNVs called by inStrain as it does not output SNV quality scores. The SNV phasing was performed on high-abundance species using WhatsHap [27] with the inferred ploidy. LRTK also selected SNVs located on certain high abundance species and compared the SNV profiles across multiple samples. Based on the allele frequency of SNVs, we used an unsupervised clustering method to detect potential strain frequency change events for pairwise

samples. To detect strain frequency change, one sample was chosen as the reference for all the contigs. For each species that was present in at least two samples, the minor allele was determined based on the reference sample. The minor allele frequency (MAF) was calculated as the ratio of the minor allele count to the total allele count. For each interested species, MAF of SNVs (minimum supported read number exceeding 2) were extracted from each sample and merged into a combined SNV set. Based on the combined SNV set, BAF values for the interested sample were then compared with the BAFs of the reference sample. We used the k-means clustering method to separate the SNVs into distinct groups using the minor allele frequency matrix. The number of clusters was determined by using the Calinski-Harabasz index.

Downstream analysis and HTML-based visualization

The human genome analysis report begins with generating the FASTQ quality control statistics during the preprocessing step. The barcode-aware alignment step presents the distribution of several key statistics about the DNA sequencing library, including the number of fragments per barcode, fragment length and average read coverage per fragment. It also includes information about the sequencing coverage and insert size. The variant calling step summarizes the number of SNVs, small INDELs and large SVs and illustrates their distributions.

Similarly, the metagenome analysis report illustrates the QC results for preprocessing, alignment and variant calling steps. Additionally, it includes an optional report about the automatic analysis of multiple related samples. The report

shows the distribution of high abundant species across multiple samples, and uses a principal component analysis plot to visualize the divergence across them. The report also depicts the distribution of SNVs of these high abundant species, and the minor allele frequency distribution in pairwise samples.

Code Availability and Requirements

Project name: Linked Read ToolKits project

Project home page: https://github.com/ericcombiolab/LRTK

Packaged conda environment: https://anaconda.org/bioconda/lrtk

Operating system(s): Linux and macOS

Programming language: C and Python (Python Programming Language, RRID:SCR_008394)

Other requirements: Conda (Conda, RRID:SCR_018317), Python 3.6 or higher

License: MIT

RRID: SCR_023945

Biotools ID: biotools:lrtk

Data Availability

We included one simulated metagenomic dataset (S1), one mock microbial community (B1) and two human gut metagenomic sequencing datasets (D1, D2) in

the metagenome section and the human genome sequencing data for four samples (D3) in the human genome section **(Table S2).**

B1: The mock microbial community B1 contains the 10x Genomics, stLFR and TELL-Seq sequencing data for ATCC MSA-1003 mock community. These datasets were obtained from the NCBI with the following accession numbers: SRR12283286 for 10x Genomics, and PRJNA875547 for stLFR and TELL-Seq sequencing technologies.

D1: The first real metagenomic dataset (D1), consisting of longitudinal 10x Genomics linked-read sequencing data, was downloaded from the NCBI under accession number SRP323279.

D2: The second real metagenomic dataset (D2), containing deep stLFR sequencing data, was downloaded from the China National GeneBank (CNGB) under project CNP0003432.

D3: The human genome dataset D3 contains the linked-read sequencing data for NA12878, NA24143, NA24149 and NA24385. The 10x Genomics and stLFR sequencing data was downloaded following the links in **Table S2**. For TELL-Seq sequencing data, we only obtained the raw TELL-Seq data from NA12878 and NA24385 from the SRA database under the accession SRX7264479 and SRX7264481, respectively.

Supporting data is also available via the GigaScience database, GigaDB [60]. An archival copy of the code is available via Software Heritage [61].

Abbreviations

stLFR: single-tube long fragment read

TELL-Seq: transposase enzyme-linked long-read sequencing

SNV: single nucleotide variant

INDEL: small insertion and deletion

SV: structural variation

CRediT authorship contribution statement:

Chao Yang: Writing - original draft, and preparing figures and tables, preparing source codes; **Zhenmiao Zhang:** preparing source codes; **Xuefeng Xie:** Consolidating resources; **Yufen Huang:** Consolidating resources; **Herui Liao:** preparing source codes; **Werner P Veldsman:** Revising - original draft, and visualizations; **Jin Xiao:** Revising - original draft, and visualizations; **Kejing Yin:** Supervision; **Xiaodong Fang:** Supervision; **Lu Zhang:** Project administer, Writing – review & editing, Supervision, and funding acquisition.

Declaration of Competing Interest:

The authors declare that they have no known competing financial interests

Acknowledgments:

This research was partially supported by the open project of BGI-Shenzhen, Shenzhen 518000, China (BGIRSZ20220012), the Hong Kong Research Grant Council Early Career Scheme (HKBU 22201419), Young Collaborative Research Grant (C2004-23Y), Health and Medical Research Fund (11221026), HKBU Start-up Grant Tier 2 (RC-SGT2/19-20/SCI/007), HKBU IRCMS (No. IRCMS/19-20/D02), the Guangdong Basic and Applied Basic Research Foundation (No. 2021A1515012226), the Science Technology and Innovation Committee of Shenzhen Municipality, China (SGDX20190919142801722).

Additional Files

Figure S1. LRTK text file specification.

Figure S2. Ideogrammatic definitions of C_R, C_F, $N_{F/P}$, μ_{FL}, and $W\mu_{FL}$ metrics.

Figure S3. Evaluation of taxonomic quantification performance for 10x Genomics and TELL-Seq linked-reads.

Figure S4. Evaluation of assembly performance at species level.

Figure S5. Evaluation of metagenomic assemblers on simulated linked-read data.

Figure S6. Demo reports for metagenomic sequencing.

Figure S7. Demo reports for human genome sequencing.

Figure S8. Computational requirements for linked-reads preprocessing and alignment between LRTK and Long Ranger on NA12878.

Table S1. Bioinformatics tools included in LRTK.

Table S2. Linked-read sequencing data used in the manuscript.

Table S3. Data descriptions for the simulated and down-sampled linked-reads from ATCC-MSA-1003 and NA12878.

Table S4. Reference genomes for the ATCC-MSA-1003 mock sample.

Table S5. Reference genomes for the simulated dataset.

References

1. Bergström A, McCarthy SA, Hui R, Almarri MA, Ayub Q, Danecek P, et al.. Insights into human genetic variation and population history from 929 diverse genomes. *Science (80-)*. American Association for the Advancement of Science; 2020; doi: 10.1126/SCIENCE.AAY5012/SUPPL_FILE/AAY5012-BERGSTROM-SM.PDF.

2. Dréau A, Venu V, Avdievich E, Gaspar L, Jones FC. Genome-wide recombination map construction from single individuals using linked-read sequencing. *Nat Commun 2019 101*. Nature Publishing Group; 2019; doi: 10.1038/s41467-019-12210-9.

3. Roodgar M, Good BH, Garud NR, Martis S, Avula M, Zhou W, et al.. Longitudinal linked-read sequencing reveals ecological and evolutionary responses of a human gut microbiome during antibiotic treatment. *Genome Res*. Cold Spring Harbor Laboratory Press; 2021; doi: 10.1101/GR.265058.120.

4. Hadi K, Yao X, Behr JM, Deshpande A, Xanthopoulakis C, Tian H, et al.. Distinct Classes of Complex Structural Variation Uncovered across Thousands of Cancer Genome Graphs. *Cell*. Cell Press; 2020; doi: 10.1016/J.CELL.2020.08.006.

5. Spies N, Weng Z, Bishara A, McDaniel J, Catoe D, Zook JM, et al.. Genome-wide reconstruction of complex structural variants using read clouds. *Nat Methods 2017 149*. Nature Publishing Group; 2017; doi: 10.1038/nmeth.4366.

6. Wang O, Chin R, Cheng X, Yan Wu MK, Mao Q, Tang J, et al.. Efficient and unique co-barcoding of second-generation sequencing reads from long DNA molecules enabling cost effective and accurate sequencing, haplotyping, and de novo

assembly. *Genome Res*. Cold Spring Harbor Laboratory Press; 2019; doi: 10.1101/GR.245126.118.

7. Chen Z, Pham L, Wu TC, Mo G, Xia Y, Chan PL, et al.. Ultralow-input single-tube linked-read library method enables short-read second-generation sequencing systems to routinely generate highly accurate and economical long-range sequencing information. *Genome Res*. Cold Spring Harbor Laboratory Press; 2020; doi: 10.1101/gr.260380.119.

8. Siranosian BA, Brooks EF, Andermann T, Rezvani AR, Banaei N, Tang H, et al.. Rare transmission of commensal and pathogenic bacteria in the gut microbiome of hospitalized adults. *Nat Commun 2022 131*. Nature Publishing Group; 2022; doi: 10.1038/s41467-022-28048-7.

9. Huang Y, Jiang P, Liang Z, Chen R, Yue Z, Xie X, et al.. Assembly and analytical validation of a metagenomic reference catalog of human gut microbiota based on co-barcoding sequencing. *Front Microbiol*. Frontiers; 2023; doi: 10.3389/FMICB.2023.1145315.

10. Davila Aleman FD. Microbiome and aging: A study of microbial evolution and community structure across model organisms. Abtract and Metadata. 2022;

11. Tracanna V, Ossowicki A, Petrus MLC, Overduin S, Terlouw BR, Lund G, et al.. Dissecting Disease-Suppressive Rhizosphere Microbiomes by Functional Amplicon Sequencing and 10× Metagenomics. *mSystems*. American Society for Microbiology; 2021; doi: 10.1128/MSYSTEMS.01116-20/SUPPL_FILE/MSYSTEMS.0116-20-S0001.PDF.

12. Tolstoganov I, Pevzner PA, Korobeynikov A. SpLitteR: Diploid genome assembly using linked TELL-Seq reads and assembly graphs. *bioRxiv*. Cold Spring Harbor Laboratory; 2022; doi: 10.1101/2022.12.08.519233.

13. Marks P, Garcia S, Barrio AM, Belhocine K, Bernate J, Bharadwaj R, et al.. Resolving the full spectrum of human genome variation using Linked-Reads. *Genome Res*. 2019; doi: 10.1101/gr.234443.118.

14. Viswanathan SR, Ha G, Hoff AM, Wala JA, Carrot-Zhang J, Whelan CW, et al.. Structural Alterations Driving Castration-Resistant Prostate Cancer Revealed by Linked-Read Genome Sequencing. *Cell*. Cell Press; 2018; doi: 10.1016/J.CELL.2018.05.036.

15. Greer SU, Nadauld LD, Lau BT, Chen J, Wood-Bouwens C, Ford JM, et al.. Linked read sequencing resolves complex genomic rearrangements in gastric cancer metastases. *Genome Med*. Genome Medicine; 2017; doi: 10.1186/s13073-017-0447-8.

16. Zheng GXY, Lau BT, Schnall-Levin M, Jarosz M, Bell JM, Hindson CM, et al.. Haplotyping germline and cancer genomes with high-throughput linked-read sequencing. *Nat Biotechnol 2016 343*. Nature Publishing Group; 2016; doi: 10.1038/nbt.3432.

17. Almeida A, Nayfach S, Boland M, Strozzi F, Beracochea M, Shi ZJ, et al.. A unified catalog of 204,938 reference genomes from the human gut microbiome. *Nat Biotechnol 2020 391*. Nature Publishing Group; 2020; doi: 10.1038/s41587-020-0603-3.

18. Shajii A, Numanagić I, Whelan C, Berger B. Statistical Binning for Barcoded Reads Improves Downstream Analyses. *Cell Syst*. Cell Press; 2018; doi: 10.1016/J.CELS.2018.07.005/ATTACHMENT/8C829484-1F22-47F4-A513-B90845BD41D7/MMC1.PDF.

19. Zhang L, Zhou X, Weng Z, Sidow A. Assessment of human diploid genome assembly with 10x Linked-Reads data. *Gigascience*. Gigascience; 2019; doi: 10.1093/GIGASCIENCE/GIZ141.

20. Garrison E, Marth G. Haplotype-based variant detection from short-read sequencing. 2012; doi: 10.48550/arxiv.1207.3907.

21. Li H, Handsaker B, Wysoker A, Fennell T, Ruan J, Homer N, et al.. The Sequence Alignment/Map format and SAMtools. *Bioinformatics*. Oxford Academic; 2009; doi: 10.1093/BIOINFORMATICS/BTP352.

22. McKenna A, Hanna M, Banks E, Sivachenko A, Cibulskis K, Kernytsky A, et al.. The Genome Analysis Toolkit: A MapReduce framework for analyzing next-generation DNA sequencing data. *Genome Res*. Cold Spring Harbor Laboratory Press; 2010; doi: 10.1101/GR.107524.110.

23. Zhou X, Zhang L, Weng Z, Dill DL, Sidow A. Aquila enables reference-assisted diploid personal genome assembly and comprehensive variant detection based on linked reads. *Nat Commun 2021 121*. Nature Publishing Group; 2021; doi: 10.1038/s41467-021-21395-x.

24. Fang L, Kao C, Gonzalez M V., Mafra FA, Pellegrino da Silva R, Li M, et al.. LinkedSV for detection of mosaic structural variants from linked-read exome and

genome sequencing data. *Nat Commun 2019 101*. Nature Publishing Group; 2019; doi: 10.1038/s41467-019-13397-7.

25. Karaoğlanoğlu F, Ricketts C, Ebren E, Rasekh ME, Hajirasouliha I, Alkan C. VALOR2: characterization of large-scale structural variants using linked-reads. *Genome Biol*. BioMed Central Ltd.; 2020; doi: 10.1186/S13059-020-01975-8/TABLES/2.

26. Edge P, Bafna V, Bansal V. HapCUT2: robust and accurate haplotype assembly for diverse sequencing technologies. *Genome Res*. Genome Res; 2017; doi: 10.1101/GR.213462.116.

27. Patterson MD, Marschall T, Pisanti N, Van Iersel L, Stougie L, Klau GW, et al.. WhatsHap: Weighted Haplotype Assembly for Future-Generation Sequencing Reads. *https://home.liebertpub.com/cmb*. Mary Ann Liebert, Inc. 140 Huguenot Street, 3rd Floor New Rochelle, NY 10801 USA ; 2015; doi: 10.1089/CMB.2014.0157.

28. Zhang L, Kong Baptist H, Zhang Z, Kong H, Xiao J, Wang H, et al.. Exploring high-quality microbial genomes by assembling short-reads with long-range connectivity. 2023; doi: 10.21203/RS.3.RS-3280231/V1.

29. Zhang L, Fang X, Liao H, Zhang Z, Zhou X, Han L, et al.. A comprehensive investigation of metagenome assembly by linked-read sequencing. *Microbiome*. BioMed Central Ltd; 2020; doi: 10.1186/S40168-020-00929-3/FIGURES/4.

30. Zhang L, Zhou X, Weng Z, Sidow A. De novo diploid genome assembly for genome-wide structural variant detection. *NAR Genomics Bioinforma*. Oxford Academic; 2020; doi: 10.1093/NARGAB/LQZ018.

31. Lu J, Breitwieser FP, Thielen P, Salzberg SL. Bracken: estimating species abundance in metagenomics data. *PeerJ Comput Sci*. PeerJ Inc.; 2017; doi: 10.7717/PEERJ-CS.104.

32. Shen W, Xiang H, Huang T, Tang H, Peng M, Cai D, et al.. KMCP: accurate metagenomic profiling of both prokaryotic and viral populations by pseudo-mapping. *Bioinformatics*. Oxford Academic; 2023; doi: 10.1093/BIOINFORMATICS/BTAC845.

33. Segata N, Waldron L, Ballarini A, Narasimhan V, Jousson O, Huttenhower C. Metagenomic microbial community profiling using unique clade-specific marker genes. *Nat Methods*. 2012; doi: 10.1038/nmeth.2066.

34. Zhao C, Dimitrov B, Goldman M, Nayfach S, Pollard KS. MIDAS2: Metagenomic Intra-species Diversity Analysis System. *Bioinformatics*. Oxford Academic; 2023; doi: 10.1093/BIOINFORMATICS/BTAC713.

35. Olm MR, Crits-Christoph A, Bouma-Gregson K, Firek BA, Morowitz MJ, Banfield JF. inStrain profiles population microdiversity from metagenomic data and sensitively detects shared microbial strains. *Nat Biotechnol 2021 396*. Nature Publishing Group; 2021; doi: 10.1038/s41587-020-00797-0.

36. Li D, Liu CM, Luo R, Sadakane K, Lam TW. MEGAHIT: An ultra-fast single-node solution for large and complex metagenomics assembly via succinct de Bruijn graph. *Bioinformatics*. 2015; doi: 10.1093/bioinformatics/btv033.

37. Nurk S, Meleshko D, Korobeynikov A, Pevzner PA. metaSPAdes: a new versatile metagenomic assembler. *Genome Res*. Cold Spring Harbor Laboratory Press; 2017; doi: 10.1101/GR.213959.116.

38. Bishara A, Moss EL, Kolmogorov M, Parada AE, Weng Z, Sidow A, et al.. High-quality genome sequences of uncultured microbes by assembly of read clouds. *Nat Biotechnol*. Nat Biotechnol; 2018; doi: 10.1038/NBT.4266.

39. Tolstoganov I, Bankevich A, Chen Z, Pevzner PA. cloudSPAdes: assembly of synthetic long reads using de Bruijn graphs. *Bioinformatics*. Bioinformatics; 2019; doi: 10.1093/BIOINFORMATICS/BTZ349.

40. Kang DD, Li F, Kirton E, Thomas A, Egan R, An H, et al.. MetaBAT 2: An adaptive binning algorithm for robust and efficient genome reconstruction from metagenome assemblies. *PeerJ*. PeerJ Inc.; 2019; doi: 10.7717/PEERJ.7359/SUPP-3.

41. Zhou X, Batzoglou S, Sidow A, Zhang L. HAPDeNovo: A haplotype-based approach for filtering and phasing de novo mutations in linked read sequencing data. *BMC Genomics*. BioMed Central Ltd.; 2018; doi: 10.1186/S12864-018-4867-7/TABLES/3.

42. Chaisson MJP, Sanders AD, Zhao X, Malhotra A, Porubsky D, Rausch T, et al.. Multi-platform discovery of haplotype-resolved structural variation in human genomes. *Nat Commun 2019 101*. Nature Publishing Group; 2019; doi: 10.1038/s41467-018-08148-z.

43. Kavak P, Lin YY, Numanagić I, Asghari H, Güngör T, Alkan C, et al.. Discovery and genotyping of novel sequence insertions in many sequenced individuals. *Bioinformatics*. Oxford Academic; 2017; doi: 10.1093/BIOINFORMATICS/BTX254.

44. Krannich T, Timothy W, Niehus S, Holley G, Halldorsson B V., Kehr B. Population-scale detection of non-reference sequence variants using colored de Bruijn graphs. *Bioinformatics*. Oxford Academic; 2022; doi: 10.1093/BIOINFORMATICS/BTAB749.

45. Altshuler DL, Durbin RM, Abecasis GR, Bentley DR, Chakravarti A, Clark AG, et al.. A map of human genome variation from population-scale sequencing. *Nature*. 2010; doi: 10.1038/nature09534.

46. Pasolli E, Asnicar F, Manara S, Zolfo M, Karcher N, Armanini F, et al.. Extensive Unexplored Human Microbiome Diversity Revealed by Over 150,000 Genomes from Metagenomes Spanning Age, Geography, and Lifestyle. *Cell*. Cell; 2019; doi: 10.1016/J.CELL.2019.01.001.

47. Xia LC, Bell JM, Wood-Bouwens C, Chen JJ, Zhang NR, Ji HP. Identification of large rearrangements in cancer genomes with barcode linked reads. *Nucleic Acids Res*. Oxford Academic; 2018; doi: 10.1093/NAR/GKX1193.

48. Zhang F, Christiansen L, Thomas J, Pokholok D, Jackson R, Morrell N, et al.. Haplotype phasing of whole human genomes using bead-based barcode partitioning in a single tube. *Nat Biotechnol 2017 359*. Nature Publishing Group; 2017; doi: 10.1038/nbt.3897.

49. Meier JI, Salazar PA, Kučka M, Davies RW, Dréau A, Aldás I, et al.. Haplotype tagging reveals parallel formation of hybrid races in two butterfly species. *Proc Natl Acad Sci U S A*. National Academy of Sciences; 2021; doi: 10.1073/PNAS.2015005118/SUPPL_FILE/PNAS.2015005118.SD06.XLSX.

50. Redin D, Frick T, Aghelpasand H, Käller M, Borgström E, Olsen RA, et al.. High throughput barcoding method for genome-scale phasing. *Sci Reports 2019 91*. Nature Publishing Group; 2019; doi: 10.1038/s41598-019-54446-x.

51. Zheng W, Zhao S, Yin Y, Zhang H, Needham DM, Evans ED, et al.. High-throughput, single-microbe genomics with strain resolution, applied to a human gut microbiome. *Science (80-)*. American Association for the Advancement of Science; 2022; doi: 10.1126/SCIENCE.ABM1483/SUPPL_FILE/SCIENCE.ABM1483_MOVIES_S1_TO_S10.ZIP.

52. Chen S, Zhou Y, Chen Y, Gu J. fastp: an ultra-fast all-in-one FASTQ preprocessor. *Bioinformatics*. Oxford Academic; 2018; doi: 10.1093/BIOINFORMATICS/BTY560.

53. Bowers RM, Kyrpides NC, Stepanauskas R, Harmon-Smith M, Doud D, Reddy TBK, et al.. Minimum information about a single amplified genome (MISAG) and a metagenome-assembled genome (MIMAG) of bacteria and archaea. *Nat Biotechnol 2017 358*. Nature Publishing Group; 2017; doi: 10.1038/nbt.3893.

54. Schneider VA, Graves-Lindsay T, Howe K, Bouk N, Chen HC, Kitts PA, et al.. Evaluation of GRCh38 and de novo haploid genome assemblies demonstrates the

enduring quality of the reference assembly. *Genome Res*. Cold Spring Harbor Laboratory Press; 2017; doi: 10.1101/GR.213611.116.

55. Parks DH, Chuvochina M, Rinke C, Mussig AJ, Chaumeil P-A, Hugenholtz P. GTDB: an ongoing census of bacterial and archaeal diversity through a phylogenetically consistent, rank normalized and complete genome-based taxonomy. *Nucleic Acids Res*. Oxford University Press (OUP); 2021; doi: 10.1093/NAR/GKAB776.

56. Murillo GH, You N, Su X, Cui W, Reilly MP, Li M, et al.. MultiGeMS: detection of SNVs from multiple samples using model selection on high-throughput sequencing data. *Bioinformatics*. Oxford Academic; 2016; doi: 10.1093/BIOINFORMATICS/BTV753.

57. Majidian S, Sedlazeck FJ. PhaseME: Automatic rapid assessment of phasing quality and phasing improvement. *Gigascience*. Oxford University Press; 2020; doi: 10.1093/gigascience/giaa078.

58. Zhou Y, Browning SR, Browning BL. A Fast and Simple Method for Detecting Identity-by-Descent Segments in Large-Scale Data. *Am J Hum Genet*. Cell Press; 2020; doi: 10.1016/J.AJHG.2020.02.010.

59. Bishara A, Liu Y, Weng Z, Kashef-Haghighi D, Newburger DE, West R, et al.. Read clouds uncover variation in complex regions of the human genome. *Genome Res*. Genome Res; 2015; doi: 10.1101/GR.191189.115.

60. Yang C, Zhang Z, Huang Y, Xie X, Liao H, Xiao J et al. Supporting data for "LRTK: A platform agnostic toolkit for linked-read analysis of both human genomes and metagenomes" GigaScience Database. 2024. https://doi.org/10.5524/102524

61. . Yang C, Zhang Z, Huang Y, Xie X, Liao H, Xiao J et al. Linked-Read ToolKit (LRTK) [Software archive] https://archive.softwareheritage.org/swh:1:dir:bb79dff97f8d0c0212787b53c8b972eec8f4c1f5

Figures and Tables

Figure 1. Overview of LRTK. The LRTK workflow includes a metagenomic section (left panel) and human genomic section (right panel). The metagenomic section implements barcode correction, barcode-aware alignment and metagenome assembly, long DNA fragment reconstruction, taxonomic classification and quantification, as well as SNV detection and phasing. The human genomic section implements barcode correction, barcode-aware alignment, long DNA fragment reconstruction, and detection and phasing of SNVs, INDELs and SVs.

Figure 2. Distribution of quality metrics for different linked-read sequencing platforms. (A) metagenome sequencing. **(B)** human genome sequencing. The left panel displays the distribution of the number of long DNA fragments per barcode. The middle panel shows the length distributions of reconstructed long DNA fragments. The right panel shows the distribution of short-read coverage of fragments.

Figure 3. Comparison of linked-reads based metagenomic quantification, SNV identification and phasing. (A) Evaluation of tools to quantify taxonomic abundance based on the linked-reads from simulated dataset (left panel) and ATCC-MSA-1003 (right panel). **(B)** Comparing the performance of SAMtools, FreeBayes and inStrain to detect metagenomic SNVs. **(C)** Dynamic changes of taxonomic abundance and allele frequency. **(D)** SNV based strain phasing in pairwise samples. The right label shows the sample name in D1.

Figure 4. Evaluation of metagenome assemblies on linked-read sequencing. (A) Evaluation of the assembly performance for MEGAHIT, metaSPAdes, Athena, cloudSPAdes and Pangaea on 10x Genomics, stLFR and TELL-Seq linked-read sequencing data from ATCC-MSA-1003. Pangaea does not support 10x Genomics linked-reads. The left panel demonstrates the calculated NA50 values while the right panel shows the calculated N50 values. **(B)** Illustration of the two assembled circular contigs. **(C)** The distribution of completeness and contamination for reconstructed bins. **(D)** The number of contigs in each contig group. **(E)** The number of detected tRNAs in each contig group.

Figure 5. Evaluation of linked-read based detection of variation in the human genome. (A, B) Performance metrics on the detection of SNVs and INDELs using FreeBayes, GATK and SAMtools for 10x Genomics, stLFR and TELL-Seq. **(C, D)** The performance on phasing of small variants using HapCUT2 and WhatsHap for 10x Genomics, stLFR and TELL-Seq. **(E)** Illustration of the performance on IBD detection. **(F)** The performance on detection of deletions using Aquila, LinkedSV and

LongRanger. **(G)** The performance on detection of insertions using Aquila, Pamir and PopIns2.

Figure S1: LRTK text file specification. (A) An example of the unified FASTQ format. Each read contains a barcode field "BX:Z:barcode" after the read names. The lengths of barcodes are 16 bps, 30 bps and 18 bps for 10x Genomics linked-read, stLFR and TELL-Seq, respectively. **(B)** An example of the BAM file generated by LRTK. The barcode information is stored in the "BX:Z:barcode" field.

Figure S2. Ideogrammatic definitions of C_R, C_F, $N_{F/P}$, μ_{FL}, and $W\mu_{FL}$ metrics. (**A**) C_R: Average depth of short reads per fragment. (**B**) C_F: Average physical depth of the genome by long DNA fragments. (**C**) $N_{F/P}$: Number of fragments per barcode. (**D**) Length-weighted average (μ_{FL}) and unweighted average ($W\mu_{FL}$) of DNA fragment lengths. Here, μ FL was calculated as the mean DNA fragment length while W μ FL was calculated as the N50 value of all the DNA fragments.

Figure S3. Evaluation of taxonomic quantification performance for 10x Genomics and TELL-Seq linked-reads. (**A**) Evaluation of taxonomic quantification across LRTK, Bracken, KMCP, MetaPhlAn 2 and MIDAS 2 using the simulated 10x Genomics linked-reads. (**B**) Evaluation of taxonomic quantification across LRTK, Bracken, KMCP, MetaPhlAn 2 and MIDAS 2 using the 10x Genomics linked-reads from ATCC-MSA-1003. (**C**) Evaluation of taxonomic quantification across LRTK, Bracken, KMCP, MetaPhlAn 2 and MIDAS 2 using the TELL-Seq linked-reads from ATCC-MSA-1003.

Figure S4: Evaluation of assembly performance at the species level. The left panel demonstrates the average assembled genome fraction, NA50 and N50 for ATCC-MSA-1003. The right panel demonstrates the average assembled genome fraction, NA50 and N50 from the simulated dataset.

Figure S5: Evaluation of metagenomic assemblers on simulated linked-read data. The top panel illustrates the NA50 values across five assemblers based on the simulated data. The bottom panel illustrates the N50 values across five assemblers based on the simulated sequencing data.

Figure S6. Demo reports for metagenomic sequencing. (**A**) Per base sequencing quality scores along the reads. (**B**) Per Base GC content along the reads. (**C**) The number of fragments per barcode. (**D**) Fragment length distribution. (**E**). The abundance of the top 10 most abundant species. (**F**) The number of SNVs per species. (**G**) A principal component analysis using sample data from different groups.

Figure S7: Demo reports for human genome sequencing. (**A**) Per base sequencing quality scores along the reads. (**B**) Per Base GC content along the reads. (**C**) The number of fragments per barcode. (**D**) Fragment length distribution. (**E**) Sequencing depth frequency. (**F**) The distribution of sequencing depth along a whole genome. (**G**) The distribution of inferred insert sizes. (**H**) The distribution of detected deletions. (**I**) The distribution of detected insertions.

Figure S8: Computational requirements for linked-reads preprocessing and alignment between LRTK and Long Ranger on NA12878. (A) linked-reads preprocessing; **(B)** linked-reads alignment.

986

Table S1. Bioinformatics tools included in LRTK.

Categories	Tools
Linked read simulation	LRTK-SIM [29]
Read preprocessing	fastp [52]
Barcode-aware metagenome assembly	Pangaea [28]
Barcode-aware read alignment	Modified EMA [18] , lariat [59]
SNV detection	FreeBayes [20], GATK [22], inStrain [35], SAMtools [21]
Barcode-assited SV detection	Aquila [23], LinkedSV [24]
Barcode-assited phasing	HapCUT2 [26], WhatsHap [27]

Table S2. Linked-read sequencing data used in the manuscript.

Cohort	Sample	Sequencing Technologies	Data sources	Size
B1	ATCC-MSA-1003	10x Genomics	SRR12283286	37.7G
		stLFR	PRJNA875547	111G
		TELL-Seq	PRJNA875547	55G
D1	T10	10x Genomics	SRR14763277	14.8G
	T8		SRR14763279	6.9G
	T6		SRR14763281	8.2G
	T5		SRR14763282	40.6G
	T4		SRR14763283	39.5G
	T3		SRR14763284	4.1G
	T18		SRR14763286	10.4G
	T17		SRR14763287	18G
	T16		SRR14763288	20.4G
	T15		SRR14763289	8.4G
	T14		SRR14763290	38.5G
	T13		SRR14763291	10.3G

	T12		SRR14763292	11.6G
	T11		SRR14763293	75G
	T2		SRR14763294	38.1G
	T1		SRR14763295	38.6G
D2	S1	stLFR	CNP0003432 (CNR0585102)	98.97 G
B2	NA12878	10x Genomics	https://ftp-trace.ncbi.nlm.nih.gov/giab/ftp/data/NA12878/10Xgenomics_ChromiumGenome_LongRanger2.0_06202016/NA12878.fastqs/	142.1 G
		stLFR	https://ftp-trace.ncbi.nlm.nih.gov/giab/ftp/data/NA12878/stLFR/	251 G
		TELL-Seq	https://www.ncbi.nlm.nih.gov/sra/SRX7264479	100.4G
D3	NA24385	10x Genomics	https://ftp-trace.ncbi.nlm.nih.gov/giab/ftp/data/AshkenazimTrio/HG002_NA24385_son/10Xgenomics_ChromiumGenome/NA24385.fastqs/	160.3 G
		stLFR	https://ftp-trace.ncbi.nlm.nih.gov/giab/ftp/data/AshkenazimTrio/HG002_NA24385_son/stLFR/	227 G
		TELL-Seq	https://www.ncbi.nlm.nih.gov/sra/SRX7264481	109.5 G
	NA24143	10x Genomics	https://ftp-trace.ncbi.nlm.nih.gov/gia	83.3 G

			b/ftp/data/AshkenazimTrio/HG004_NA24143_mother/10Xgenomics_ChromiumGenome/NA24143.fastqs/	
		stLFR	https://ftp-trace.ncbi.nlm.nih.gov/giab/ftp/data/AshkenazimTrio/HG004_NA24143_mother/stLFR/	222 G
	NA24149	10x Genomics	https://ftp-trace.ncbi.nlm.nih.gov/giab/ftp/data/AshkenazimTrio/HG003_NA24149_father/10Xgenomics_ChromiumGenome/NA24149.fastqs/	78.3 G
		stLFR	https://ftp-trace.ncbi.nlm.nih.gov/giab/ftp/data/AshkenazimTrio/HG003_NA24149_father/stLFR/	248 G

Table S3. Data descriptions for the simulated and down-sampled linked-reads from ATCC-MSA-1003 and NA12878.

	Simulated dataset	
**Statistics**	**10x Genomics**	**stLFR**
Total Reads (Mb)	134.1	92.17
Total Bases (Gb)	11.87	9.22
Q20 Bases (Gb)	11.07	8.57
Q30 Bases (Gb)	10.41	8.02

GC content	54.87%	50.63%	
reads passed filters	99.99%	99.99%	
reads with low quality	0	0	
reads with too many N	< 0.01%	< 0.01%	
reads too short	< 0.01%	< 0.01%	
Total number of barcodes	228,824	1,887,428	
Mean number of read pairs per barcode	293.02	24.42	
Median number of read pairs per barcode	284	17	
	**ATCC1003**		
**Statistics**	**10x Genomics**	**stLFR**	**TELL-Seq**
Total Reads (Mb)	151.85	201.2	156.34
Total Bases (Gb)	19.27	20.12	20.25
Q20 Bases (Gb)	19.17	19.12	19.61
Q30 Bases (Gb)	18.9	17.07	18.69
GC content	53.14%	48.75%	46.20%
reads passed filters	99.59%	94.71%	99.99%
reads with low quality	0	0.0348	0
reads with too many N	< 0.01%	< 0.01%	< 0.01%
reads too short	< 0.01%	0.0186	< 0.01%
Total number of barcodes	710,283	8,894,042	3,652,328
Mean number of read pairs per barcode	106.89	12.0864	22.81
Median number of read pairs per barcode	26	6	3
	**NA12878**		
**Statistics**	**10x Genomics**	**stLFR**	**TELL-Seq**
Total Reads (Mb)	794.35	1,097.29	754.79
Total Bases (Gb)	108.85	109.73	110.2
Q20 Bases (Gb)	102.84	104.02	105.27
Q30 Bases (Gb)	96.57	92.3	99.73
GC content	41.50%	41.91%	45.00%
reads passed filters	99.99%	95.73%	94.15%
reads with low quality	< 0.01%	3.09%	0.43%
reads with too many N	< 0.01%	< 0.01%	< 0.01%
reads too short	< 0.01%	1.18%	5.41%
Total number of barcodes	2,331,331	15,826,079	16,909,555
Mean number of read pairs per barcode	170.36	35.61966	23.42
Median number of read pairs per barcode	7	2	1

Table S4. Reference genomes for the ATCC-MSA-1003 mock sample.

Organism name	Relative abundance	Reference genome	Genome size (bp)	ATCC links
Acinetobacter_baumannii	0.18%	ATCC 17978	4,075,779	https://genomes.atcc.org/genomes/e1d18ea4273549a0
Bacillus_pacificus	1.80%	ATCC 10987	5,442,819	https://genomes.atcc.org/genomes/687931d9b06b4cb4
Bifidobacterium_adolescentis	0.02%	ATCC 15703	2,089,630	https://genomes.atcc.org/genomes/90eb97d11e4b445f
Cereibacter_sphaeroides	18.00%	ATCC 17029	2,089,630	https://genomes.atcc.org/genomes/3a2ecd8337b14710
Clostridium_beijerinckii	1.80%	ATCC 35702	6,007,460	https://genomes.atcc.org/genomes/3210fc7fdeb14ad2
Cutibacterium_acnes	0.18%	ATCC 11828	2,497,484	https://genomes.atcc.org/genomes/070cc66203ff4f5a
Deinococcus_radiodurans	0.02%	ATCC BAA-816	3,280,465	https://genomes.atcc.org/genomes/2c9f475933234e78
Enterococcus_faecalis	0.02%	ATCC 47077	2,738,556	https://genomes.atcc.org/genomes/d8b30e7d0fd74a12
Escherichia_coli	18.00%	ATCC 700926	4,642,497	https://genomes.atcc.org/genomes/8f5bf4f0d4a04c50
Helicobacter_pylori	0.18%	ATCC 700392	1,667,794	https://genomes.atcc.org/genomes/9038b5a9e94245e8
Lactobacillus_gasseri	0.18%	ATCC 33323	1,854,273	https://genomes.atcc.org/genomes/b926c8c7fe4440b5

Neisseria_meningitidis	0.18%	ATCC BAA-335	2,243,896	https://genomes.atcc.org/genomes/261b0e41db924d0f
Phocaeicola_vulgatus	0.02%	ATCC 8482	5,163,177	https://genomes.atcc.org/genomes/b7dcc8d57632456b
Porphyromonas_gingivalis	18.00%	ATCC 33277	2,399,479	https://genomes.atcc.org/genomes/0472781aae86424a
Pseudomonas_paraeruginosa	1.80%	ATCC 9027	6,374,461	https://genomes.atcc.org/genomes/c1a92e4fc09a4ed2
Schaalia_odontolytica	0.02%	ATCC 17982	2,396,044	https://genomes.atcc.org/genomes/6823ab7475dd4769
Staphylococcus_aureus	1.80%	ATCC BAA-1556	2,923,627	https://genomes.atcc.org/genomes/63b4b3239efa42df
Staphylococcus_epidermidis	18.00%	ATCC 12228	2,575,951	https://genomes.atcc.org/genomes/d1ef0271f5b14846
Streptococcus_agalactiae	1.80%	ATCC BAA-611	2,159,783	https://genomes.atcc.org/genomes/e74f122703624e54
Streptococcus_mutans	18.00%	ATCC 700610	2,031,444	https://genomes.atcc.org/genomes/d0622592e00d4ee0

Table S5. Reference genomes for the simulated dataset.

Organism name	Relative abundance	Reference genome	Genome size (bp)
Thermoanaerobacter pseudethanolicus ATCC 33223	0.003055997	NC_010321.1	2,362,816
Desulfitobacterium hafniense DCB-2	0.00607231	NC_011830.1	5,279,134

Desulfofarcimen acetoxidans DSM 771	0.004800511	NC_013216.1	4,545,624
Xylanimonas cellulosilytica DSM 15894	0.039646357	NC_013530.1	3,831,380
Sulfurimonas autotrophica DSM 16294	0.088701172	NC_014506.1	2,153,198
Corynebacterium resistens DSM 45100	0.002236753	NC_015673.1	2,601,311
Bartonella clarridgeiae 73	0.020130366	NC_014932.1	1,522,743
Isoalcanivorax pacificus W11-5	0.015516627	NZ_CP004387.1	4,168,427
Pseudobdellovibrio exovorus JSS	0.003212947	NC_020813.1	2,657,893
Psychromicrobium lacuslunae	0.010557049	NZ_CP011005.1	3,599,434
Helicobacter typhlonius	0.020004482	NZ_LN907858.1	1,920,832
Wolbachia endosymbiont of Folsomia candida	0.05823542	NZ_CP015510.2	1,801,626
Sporosarcina ureae	0.017837164	NZ_CP015108.1	3,362,333
Sedimentisphaera salicampi	0.003382715	NZ_CP021023.1	3,192,293
Fuscovulum blasticum	0.027337676	NZ_CP020470.1	3,706,095
Dongshaea marina	0.030624348	NZ_CP028897.1	5,112,152
Runella rosea	0.006440272	NZ_CP030850.1	7,162,093
Desulfovibrio ferrophilus	0.010084579	NZ_AP017378.1	3,720,107
Bradyrhizobium guangzhouense	0.018027951	NZ_CP030053.1	8,138,177
Haematobacter massiliensis	0.004961852	NZ_CP035510.1	4,232,912
Cellulomonas shaoxiangyii	0.09751048	NZ_CP039291.1	3,909,366

Microlunatus elymi	0.011982578	NZ_CP041692.1	5,121,194
Kushneria phosphatilytica	0.033312055	NZ_CP043420.1	3,624,619
Lactiplantibacillus plantarum	0.007816798	NZ_CP028221.1	3,223,643
Mycolicibacterium tokaiense	0.013904496	NZ_AP022600.1	6,328,149
Mycolicibacillus koreensis	0.008194722	NZ_AP022594.1	4,155,701
Lactococcus garvieae	0.00591555	NZ_CP065637.1	2,084,337
Rhizorhabdus wittichii	0.036707087	NZ_CP059319.1	6,073,182
Parabacteroides goldsteinii	0.006468574	NZ_CP081906.1	7,053,599
Pseudomonas savastanoi	0.007014885	NZ_CP076652.1	5,999,881
Escherichia coli str. K-12 substr. MG1655	0.175270285	NZ_CP097882.1	4,675,188
Salinimicrobium tongyeongense	0.013594968	NZ_CP069620.1	3,509,958
Iamia majanohamensis	0.005263885	NZ_CP116942.1	4,576,919
Ligilactobacillus faecis	0.012418841	NZ_CP123639.1	2,382,213
Borreliella kurtenbachii	0.014520015	NZ_CP124058.1	1,015,378
Staphylococcus schweitzeri	0.004400918	NZ_LR134304.1	2,784,939
Cardiobacterium hominis	0.00908479	NZ_LR134365.1	2,670,755
Slackia heliotrinireducens	0.005737567	NZ_LR134379.1	3,166,158
Wolbachia endosymbiont (group A) of Icerya purchasi	0.007627304	NZ_OX366357.1	1,372,034
Wolbachia endosymbiont (group A) of Hylaeus communis	0.132387656	NZ_OX366396.1	1,526,905

997

Figure1

Click here to access/download;Figure;Figure1.pdf

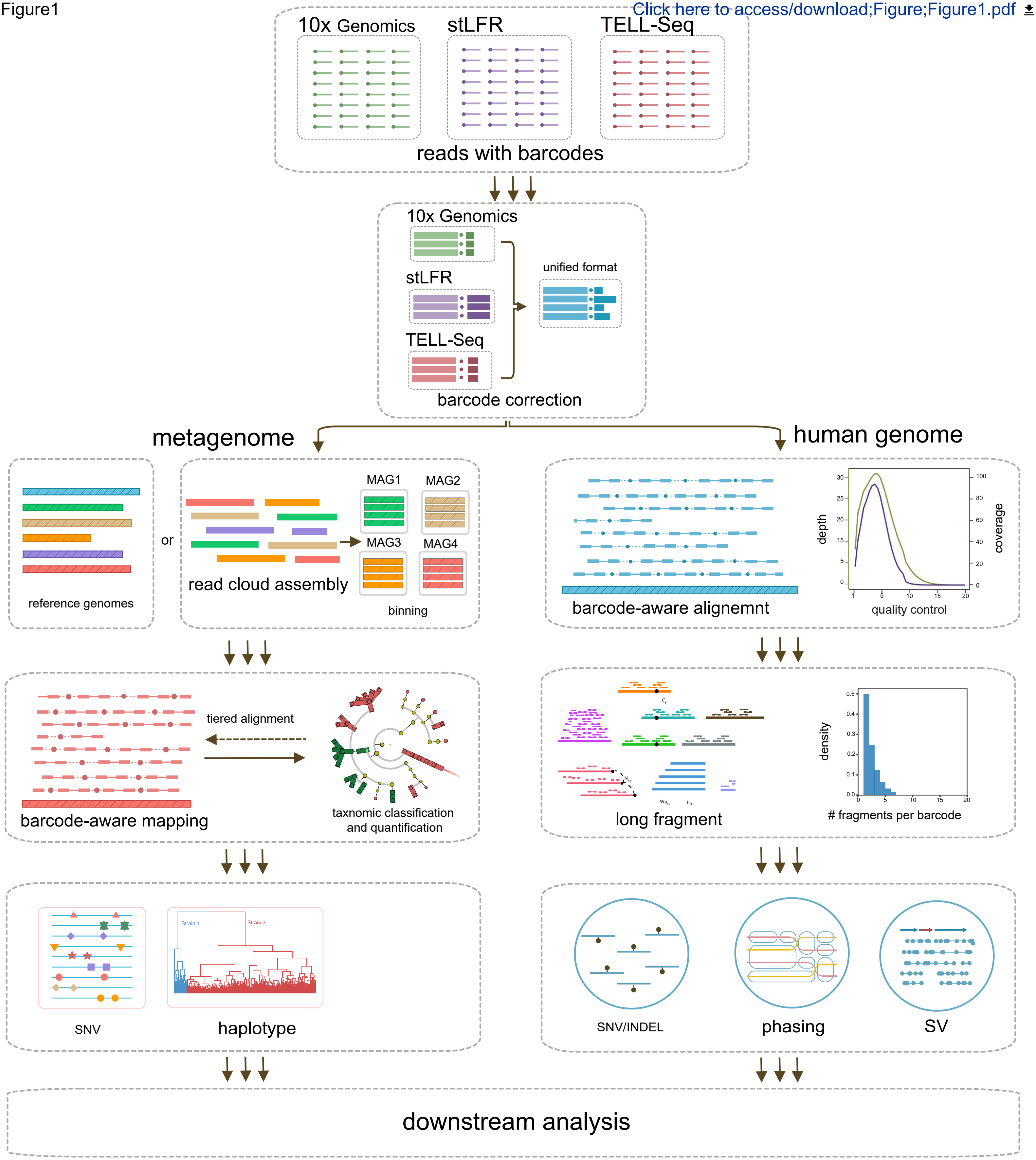

Figure2

Click here to access/download;Figure;Figure2.pdf

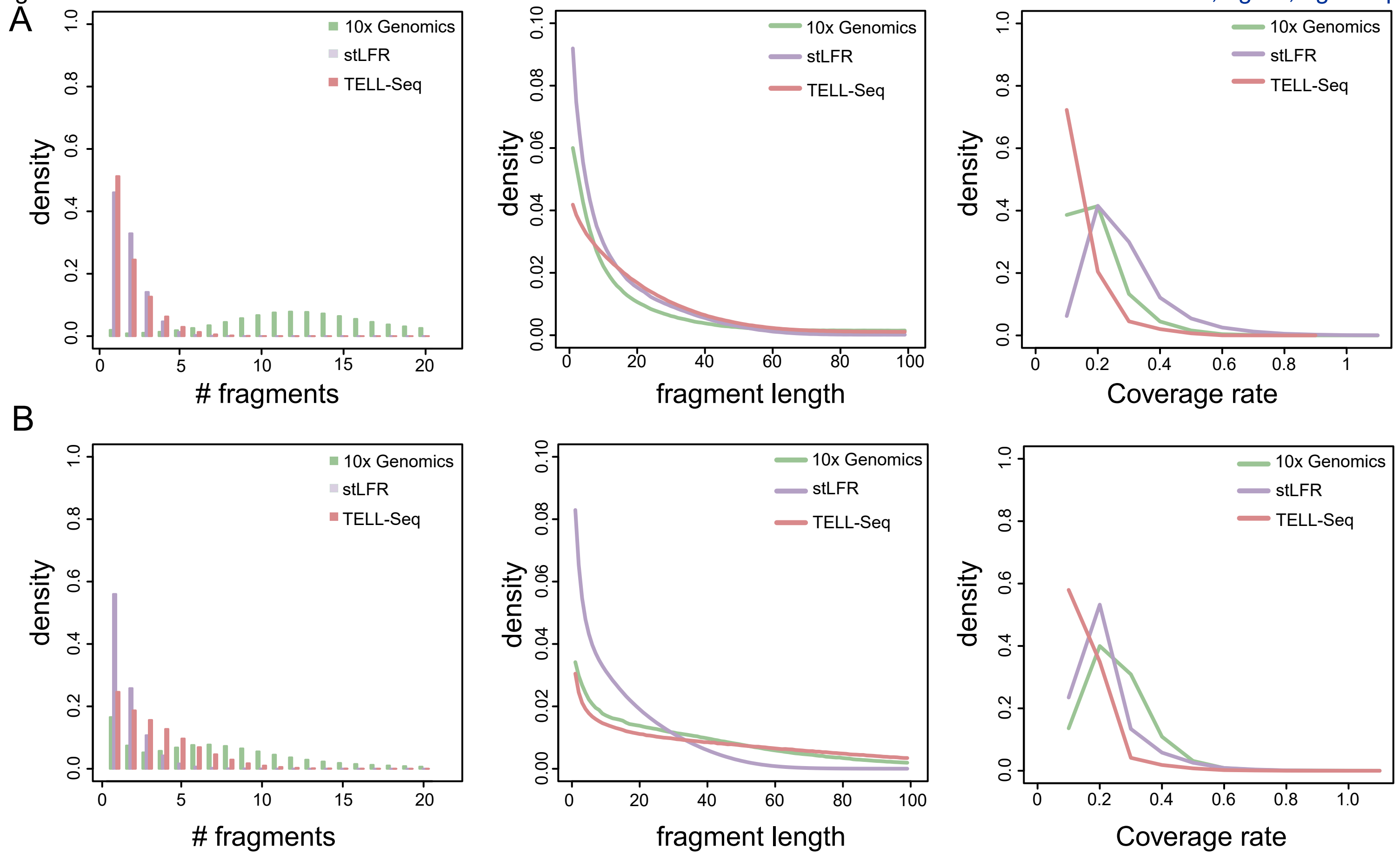

Figure3

Click here to access/download;Figure;Figure3.pdf

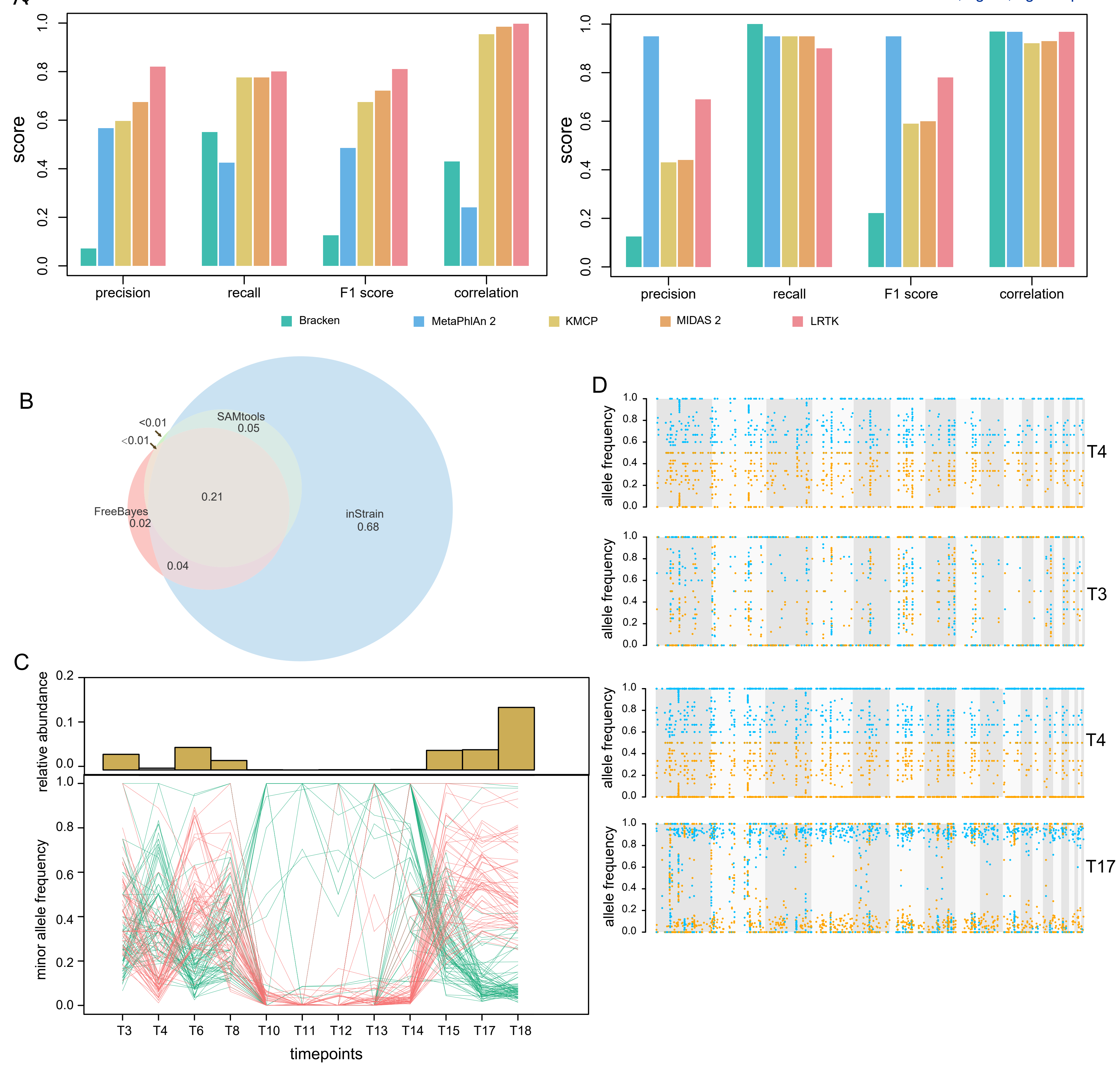

Figure4

Click here to access/download;Figure;Figure4.pdf

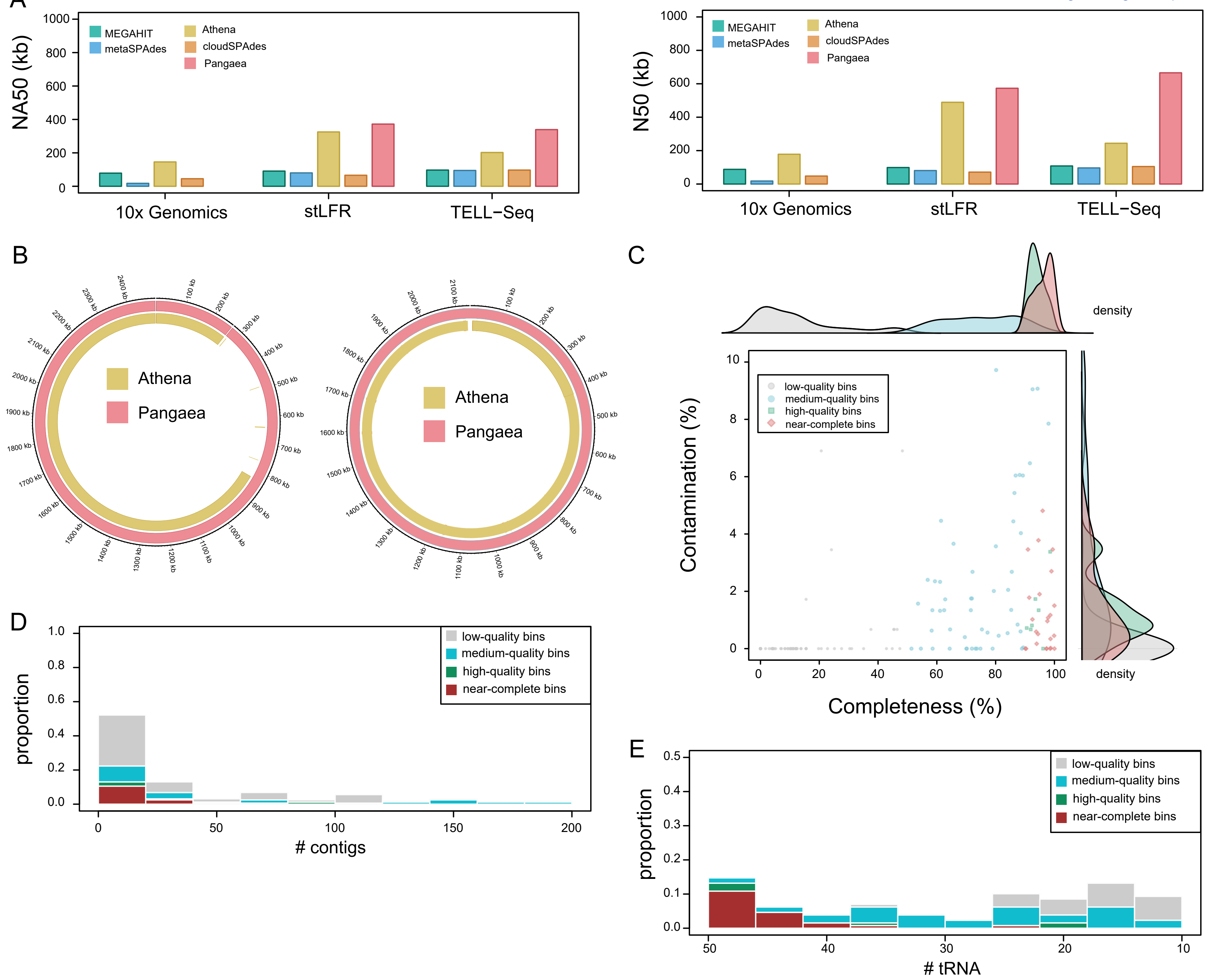

Figure5

Click here to access/download;Figure;Figure5.pdf

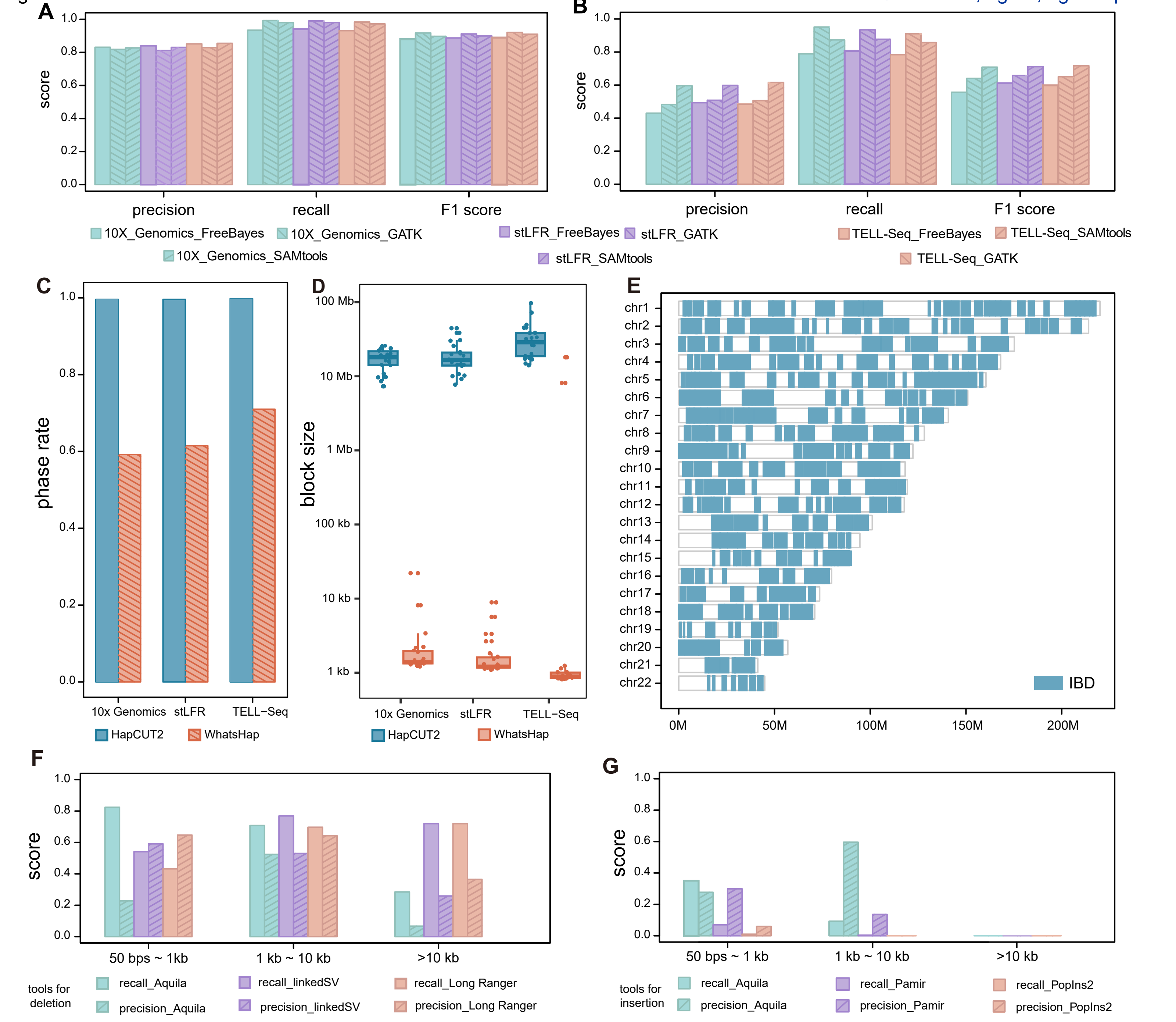

FigureS1

Click here to access/download
Supplementary Material
FigureS1.pdf

FigureS2

Click here to access/download
Supplementary Material
FigureS2.pdf

FigureS3

Click here to access/download
Supplementary Material
FigureS3.pdf

FigureS4

Click here to access/download
Supplementary Material
FigureS4.pdf

FigureS5

Click here to access/download
Supplementary Material
FigureS5.pdf

FigureS6

Click here to access/download
Supplementary Material
FigureS6.pdf

FigureS7

Click here to access/download

Supplementary Material

FigureS7.pdf

FigureS8

Click here to access/download
Supplementary Material
FigureS8.pdf

SupplementaryTables1_5

Click here to access/download
Supplementary Material
SupplementaryTables.xlsx

response letter

Click here to access/download;Personal Cover;LRTK.revised.ResponseLetter.docx

Cover Letter

Dear editors and reviewers,

Many thanks indeed for all your hard work on our manuscript, and thanks a lot for the constructive and valuable comments that providing us insights and inspirations. We have made careful revisions on the manuscript and supplementary data. All the comments from editors and reviewers have been addressed point by point as shown below, and corresponding corrections have been made in the revised manuscript.

Kindly let us know if there's any further explanation needed.

Sincerely,

Lu Zhang

Department of Computer Science, Faculty of Science,

Hong Kong Baptist University, Hong Kong

E-mail: ericluzhang@hkbu.edu.hk

Phone: +852 3411 5880

Response to Editor and Reviewers

GIGA-D-23-00278R1

LRTK: A platform agnostic toolkit for linked-read analysis of both human genome and metagenome

Lu Zhang; Chao Yang; Zhenmiao Zhang; Yufen Huang; Xuefeng Xie; Herui Liao; Jin Xiao; Werner Pieter Veldsman; Kejing Yin; Xiaodong Fang

GigaScience

Dear Dr. Zhang,

Your revised manuscript "LRTK: A platform agnostic toolkit for linked-read analysis of both human genome and metagenome" (GIGA-D-23-00278R1) has been assessed again by reviewer #2. I am pleased to inform you that it is acceptable for publication in GigaScience, in principle, once you have carried out some minor follow-up revisions suggested by the reviewer (see the comments below).

Responses to Editor

Please also address the following editorial points in the revised manuscript:

1) Please structure your abstract (Background", "Findings", "Conclusions").

Response:

We have reorganized the abstract to align with the required structure.

Background: Linked-read sequencing technologies generate high-base quality short-reads that contain extrapolative information on long-range DNA connectedness. These advantages of linked-read technologies are well-known and have been demonstrated in many human genomic and

metagenomic studies. However, existing linked-read analysis pipelines (e.g., Long Ranger) were primarily developed to process sequencing data from the human genome and are not suited for analyzing metagenomic sequencing data. Moreover, linked-read analysis pipelines are typically limited to one specific sequencing platform.

Findings: To address these limitations, we present the Linked-Read ToolKit (LRTK), a unified and versatile toolkit for platform agnostic processing of linked-read sequencing data from both human genome and metagenome. LRTK provides functions to perform linked-read simulation, barcode sequencing error correction, barcode-aware read alignment and metagenome assembly, reconstruction of long DNA fragments, taxonomic classification and quantification, as well as barcode-assisted genomic variant calling and phasing. LRTK has the ability to process multiple samples automatically, and provides users with the option to generate reproducible reports during processing of raw sequencing data and at multiple checkpoints throughout downstream analysis. We applied LRTK on linked-reads from simulation, mock community and real datasets for both human genome and metagenome. We showcased LRTK's ability to generate comparative performance results from preceding benchmark studies and to report these results in publication-ready HTML document plots.

Conclusions: LRTK provides comprehensive and flexible modules along with an easy-to-use Python-based workflow for processing linked-read sequencing datasets, thereby filling the current gap in the field caused by platform-centric genome-specific linked-read data analysis tools.

2) Pleas add ORCIDs to the title page, where available. We have the following on our files: ORCID iDs: Lu Zhang [0000-0002-2794-7371]; Chao Yang [0000-0001-6518-4574]; Zhenmiao Zhang [0000-0003-3748-1664]; Yufen Huang [0000-0001-5939-1091]; Xuefeng Xie [0000-0001-6876-673X]; Herui Liao [0000-0001-8871-3483]; Jin Xiao [0000-0001-5059-1492]; Werner Pieter Veldsman [0000-0001-9837-8332]; Kejing Yin [0000-0003-4146-3338]; Xiaodong Fang [0000-0001-7061-3337]

Response:

We have included the ORCID of each author on the title page in the revised manuscript.

ORCID iDs

Chao Yang https://orcid.org/0000-0001-6518-4574

Zhenmiao Zhang https://orcid.org/0000-0003-3748-1664

Yufen Huang https://orcid.org/0000-0001-5939-1091

Xuefeng Xie https://orcid.org/0000-0001-6876-673X

Herui Liao https://orcid.org/0000-0001-8871-3483

Jin Xiao https://orcid.org/0000-0001-5059-1492

Werner Pieter Veldsman https://orcid.org/0000-0001-9837-8332

Kejing Yin https://orcid.org/0000-0003-4146-3338

Xiaodong Fang https://orcid.org/0000-0001-7061-3337

Lu Zhang https://orcid.org/0000-0002-2794-7371

3) Please apply for a biotools ID identifier, and include it in your manuscript, in the code availability section. (https://bio.tools/)

Response:

We have incorporated the biotools ID for LRTK in the code availability section.

"Biotools ID: biotools:lrtk "

4) Our data curators will contact you shortly to prepare the release of supporting data via our database GigaDB, if applicable. If a GigaDB set is prepared, please cite this from the data availability section.

Response:

We will include GigaDB set in our manuscript once it is ready.

Responses to Reviewer2:

Reviewer reports:

Reviewer #2: The authors have responded to all of the referees' comments and suggestions thoroughly. I have a few remaining suggestions and questions.

Documentation:

The purpose of multiple tool options:

By 'Its straightforward, easy to use and timesaving.', do you mean that FreeBayes is more computationally efficient than GATK?

Response:

In our study, FreeBayes took less running time than GATK to detect SNVs and INDELs from the alignment files. For NA12878, FreeBayes and GATK spend ~4 hours and 25 hours using 64 threads. This observation is consistent with a previous study [1]. We revised the manuscript as (from **line 547 to line 549**):

FreeBayes is a Bayesian genetic variant detection tool designed to identify SNVs, INDELs, multinucleotide polymorphisms, and more complex events, which is an easy-to-use and time-saving tool.

Provenance of ATCC-MSA-1003:

The sentence 'The sequencing data volumes are 37.7 Gb, 111 Gb and 55 Gb for...' contains information that already exists in Table S3 and does not need to be repeated.

Response:

Thank you for the comment and we have removed the descriptions from the manuscript.

Table S2: There is no 10x download link for NA24385, NA24143 or NA24149.

Response:

We have included 10x Genomics data download links for NA24385, NA24143 and NA24149 in Table S2.

NA24385
https://ftp-trace.ncbi.nlm.nih.gov/giab/ftp/data/AshkenazimTrio/HG002_NA24385_son/10Xgenomics_ChromiumGenome/NA24385.fastqs/

NA24143
https://ftp-trace.ncbi.nlm.nih.gov/giab/ftp/data/AshkenazimTrio/HG004_NA24143_mother/10Xgenomics_ChromiumGenome/NA24143.fastqs/
NA24149
https://ftp-trace.ncbi.nlm.nih.gov/giab/ftp/data/AshkenazimTrio/HG003_NA24149_father/10Xgenomics_ChromiumGenome/NA24149.fastqs/

Analysis:

Lines 166-169:

The paragraph starting 'We observed, for 10x Genomics, the peak barcode count is around 7...' should be included in the main text to explain the differences in the number of fragments per barcode observed when comparing Figure 2 A1 vs. B1.

Response:

Thank you for the suggestions. We have updated the related results in the revised manuscript **from line 383 to line 401**:

For human genome sequencing, the average $N_{F/P}$ values were approximately 7 for 10x Genomics linked-reads and were declined to around 2 and 4 for stLFR and TELL-Seq linked-reads, respectively (**Figure 2B**). For metagenomic sequencing, the average values were around 14 for 10x Genomics linked-reads, 1 for stLFR and 5 for TELL-Seq linked-reads (**Figure 2A**). These differences may be attributed to the barcoding approaches employed. In the case of 10x Genomics, barcoding takes place within water-in-oil droplets, which require a specialized instrument for droplet generation. The number of DNA fragments present in each droplet is influenced by the size of the DNA fragments. The DNA fragments from the human genome are long, resulting in fewer fragments being included in each droplet. The microbial DNA fragments are relatively shorter, allowing for more fragments to be accommodated within a droplet. In contrast, neither TELL-Seq nor stLFR utilizes droplets for barcoding. Instead, the barcoding reactions occur in an open environment and are partitioned using beads alone. Generally, each bead is conjugated with at least one unique barcode sequence on its surface and can capture 1-2 DNA fragments. For stLFR and TELL-Seq, the number of DNA fragments is not dependent on the length of the DNA fragments, resulting in a similar number of fragments per barcode for both human and metagenomic sequencing.

Lines 187-188:

'For FreeBayes and SAMtools, we further removed the SNVs if the total depths were less than 6, the number of reads supporting alternative allele was less than 2, and SNV qualities were below 15.'- was this included in the manuscript?

Response:

Thank you for the comments. We have included the descriptions in the **Methods** section from **line 570 to line 574**.

For FreeBayes and SAMtools, LRTK further removes the SNVs if the total depths are less than 6, the number of reads supporting alternative allele is less than 2, and SNV qualities are below 15. LRTK does not perform quality control for the SNVs called by inStrain as it does not output SNV quality scores.

'inStrain is the most sensitive tool to detect SNVs.'- Is sensitive the right word to use? Sensitive implies that most of SNVs detected are true positives. Is that necessarily true for inStrain?

Response:

Sorry for the confusion. We revised the sentence as "inStrain is able to detect many unique microbial SNVs" from **line 243** in the revised manuscript.

Lines 192-193:

It seems that MAI implies a somatic copy number change in the maternal and paternal alleles of two haplotypes. Are you also implying that each sample should be treated as a diploid organism, and any time that only the reference or alternate allele appears alone in the sample, that it counts as a gain or loss?

I'm still not sure why this term (especially with the word 'imbalance') is being used when it seems to be a cancer-specific phenomenon, and relative abundance change, or strain frequency change, or some other phrase is more applicable for metagenomics.

Response:

Thanks for the comment, we believe "strain frequency change" is more appropriate here. We have replaced "mirrored allelic imbalance / MAI" to "strain frequency change" in the revised manuscript.

Lines 201-208:

The line 'We observed that there was a consistent decrease in the number of fragments per barcode (N_{F/P}) for the stLFR and TELL-Seq technologies.' should be added to the main text.

Response:

Thanks for the comments. We have added the descriptions in the **Discussion** section from **line 382 to line 383**.

Our findings revealed that there was a consistent decrease of $N_{F/P}$ for stLFR and TELL-Seq technologies than those obtained from 10x Genomics.

Miscellaneous:

Lines 399-405:

It seems like you're using some kind of unsupervised clustering method to infer strains i.e.: SNVs with allele frequencies that seem to be correlated. There are many methods to infer bacterial strains- why weren't any of those used?

Response:

We agree that there are many microbial strain inference tools, such as StrainFinder, STRONG, MetaMaps. However, these tools do not consider the long-range connectedness provided by linked-reads. The challenge of developing a strain inference method tailored to linked-read sequencing is still unsolved. In LRTK, we employed an unsupervised clustering method to group SNVs with similar allele frequencies. The SNVs from the same cluster were considered to be from the same strain. This strategy has been successfully applied by Roodgar M, *et al.* on longitudinal 10x Genomics linked-read sequencing dataset [2].

Line 665:

Does the 'B' in BAF (B allele frequency) refer to the alternate and/or minor allele?

Please standardize your terminology.

Response:

Thank you for the comment. We have replaced **"B allele frequency"** with **"minor allele frequency"** in the revised manuscript.

Reference

1. Murillo GH, You N, Su X, Cui W, Reilly MP, Li M, et al.. MultiGeMS: detection of SNVs from multiple samples using model selection on high-throughput sequencing data. *Bioinformatics*. Oxford Academic; 2016; doi: 10.1093/BIOINFORMATICS/BTV753.
2. Roodgar M, Good BH, Garud NR, Martis S, Avula M, Zhou W, et al.. Longitudinal linked-read sequencing reveals ecological and evolutionary responses of a human gut microbiome during antibiotic treatment. *Genome Res*. Cold Spring Harbor Laboratory Press; 2021; doi: 10.1101/GR.265058.120/-/DC1.
