# Supplementary material for: LRTK: a platform agnostic toolkit for linked-read analysis of both human genome and metagenome
Source: Gigascience. 2024 Jun 13;13:giae028. doi: 10.1093/gigascience/giae028 (PMC11170215; doi:10.1093/gigascience/giae028)
Supplement: giae028_Response_to_Reviewer_Comments_Original_Submission [file giae028_response_to_reviewer_comments_original_submission.pdf]

Author's Response To Reviewer Comments

Cover Letter
Dear editor and reviewers,
Many thanks indeed for all your hard work on our manuscript. The comments raised by reviewers are very constructive and valuable, providing us lots of insights and inspirations. We have made careful data re-analyzing and literature research, and made extensive revision to our manuscript and supplemented data. All the comments from reviewers have been addressed point by point as shown below, and corresponding corrections have been made in the revised manuscript.
Thanks for your consideration of our paper and kindly let us know if there’s any further explanation needed!

Sincerely,
Lu Zhang
Department of Computer Science, Faculty of Science, Hong Kong Baptist University, Hong Kong
E-mail: ericluzhang@hkbu.edu.hk
Phone: +852 3411 5880
