# Supplementary material for: LRTK: a platform agnostic toolkit for linked-read analysis of both human genome and metagenome
Source: Gigascience. 2024 Jun 13;13:giae028. doi: 10.1093/gigascience/giae028 (PMC11170215; doi:10.1093/gigascience/giae028)
Supplement: giae028_Response_to_Reviewer_Comments_Revision_1 [file giae028_response_to_reviewer_comments_revision_1.pdf]

Author's Response To Reviewer Comments

Dear editors and reviewers,
Many thanks indeed for all your hard work on our manuscript, and thanks a lot for the constructive and valuable comments th providing us insights and inspirations. We have made careful revisions on the manuscript and supplementary data. All the comments from editors and reviewers have been addressed point by point as shown below, and corresponding corrections ha been made in the revised manuscript.
Kindly let us know if there's any further explanation needed.

Sincerely,

Lu Zhang
Department of Computer Science, Faculty of Science,
Hong Kong Baptist University, Hong Kong
E-mail: ericluzhang@hkbu.edu.hk
Phone: +852 3411 5880

Response to Editor and Reviewers
GIGA-D-23-00278R1
LRTK: A platform agnostic toolkit for linked-read analysis of both human genome and metagenome
Lu Zhang; Chao Yang; Zhenmiao Zhang; Yufen Huang; Xuefeng Xie; Herui Liao; Jin Xiao; Werner Pieter Veldsman; Kejing Yin Xiaodong Fang
GigaScience

Dear Dr. Zhang,

Your revised manuscript "LRTK: A platform agnostic toolkit for linked-read analysis of both human genome and metagenome" (GIGA-D-23-00278R1) has been assessed again by reviewer #2. I am pleased to inform you that it is acceptable for publicati GigaScience, in principle, once you have carried out some minor follow-up revisions suggested by the reviewer (see the comm below).
Responses to Editor
Please also address the following editorial points in the revised manuscript:
1) Please structure your abstract (Background", "Findings", "Conclusions").
Response:
We have reorganized the abstract to align with the required structure.
Background: Linked-read sequencing technologies generate high-base quality short-reads that contain extrapolative informat on long-range DNA connectedness. These advantages of linked-read technologies are well-known and have been demonstrate many human genomic and metagenomic studies. However, existing linked-read analysis pipelines (e.g., Long Ranger) were primarily developed to process sequencing data from the human genome and are not suited for analyzing metagenomic sequencing data. Moreover, linked-read analysis pipelines are typically limited to one specific sequencing platform.
Findings: To address these limitations, we present the Linked-Read ToolKit (LRTK), a unified and versatile toolkit for platform agnostic processing of linked-read sequencing data from both human genome and metagenome. LRTK provides functions to perform linked-read simulation, barcode sequencing error correction, barcode-aware read alignment and metagenome assem reconstruction of long DNA fragments, taxonomic classification and quantification, as well as barcode-assisted genomic variar calling and phasing. LRTK has the ability to process multiple samples automatically, and provides users with the option to ger reproducible reports during processing of raw sequencing data and at multiple checkpoints throughout downstream analysis. applied LRTK on linked-reads from simulation, mock community and real datasets for both human genome and metagenome. showcased LRTK's ability to generate comparative performance results from preceding benchmark studies and to report these results in publication-ready HTML document plots.
Conclusions: LRTK provides comprehensive and flexible modules along with an easy-to-use Python-based workflow for proces linked-read sequencing datasets, thereby filling the current gap in the field caused by platform-centric genome-specific linked data analysis tools.

2) Pleas add ORCIDs to the title page, where available. We have the following on our files: ORCID iDs: Lu Zhang [0000-0002 2794-7371]; Chao Yang [0000-0001-6518-4574]; Zhenmiao Zhang [0000-0003-3748-1664]; Yufen Huang [0000-0001-5939 1091]; Xuefeng Xie [0000-0001-6876-673X]; Herui Liao [0000-0001-8871-3483]; Jin Xiao [0000-0001-5059-1492]; Werner

Pieter Veldsman [0000-0001-9837-8332]; Kejing Yin [0000-0003-4146-3338]; Xiaodong Fang [0000-0001-7061-3337]
Response:
We have included the ORCID of each author on the title page in the revised manuscript.
ORCID iDs
Chao Yang https://orcid.org/0000-0001-6518-4574
Zhenmiao Zhang https://orcid.org/0000-0003-3748-1664
Yufen Huang https://orcid.org/0000-0001-5939-1091
Xuefeng Xie https://orcid.org/0000-0001-6876-673X
Herui Liao https://orcid.org/0000-0001-8871-3483
Jin Xiao https://orcid.org/0000-0001-5059-1492
Werner Pieter Veldsman https://orcid.org/0000-0001-9837-8332
Kejing Yin https://orcid.org/0000-0003-4146-3338
Xiaodong Fang https://orcid.org/0000-0001-7061-3337
Lu Zhang https://orcid.org/0000-0002-2794-7371

3) Please apply for a biotools ID identifier, and include it in your manuscript, in the code availability section. (https://bio.tool
Response:
We have incorporated the biotools ID for LRTK in the code availability section.
"Biotools ID: biotools:lrtk "

4) Our data curators will contact you shortly to prepare the release of supporting data via our database GigaDB, if applicable.
GigaDB set is prepared, please cite this from the data availability section.
Response:
We will include GigaDB set in our manuscript once it is ready.

Responses to Reviewer2:
Reviewer reports:

Reviewer #2: The authors have responded to all of the referees' comments and suggestions thoroughly. I have a few remain
suggestions and questions.
Documentation:

The purpose of multiple tool options:
By 'Its straightforward, easy to use and timesaving.', do you mean that FreeBayes is more computationally efficient than GAT
Response:
In our study, FreeBayes took less running time than GATK to detect SNVs and INDELs from the alignment files. For NA12878
FreeBayes and GATK spend ~4 hours and 25 hours using 64 threads. This observation is consistent with a previous study [1]
revised the manuscript as (from line 547 to line 549):
FreeBayes is a Bayesian genetic variant detection tool designed to identify SNVs, INDELs, multinucleotide polymorphisms, an
more complex events, which is an easy-to-use and time-saving tool.

Provenance of ATCC-MSA-1003:
The sentence 'The sequencing data volumes are 37.7 Gb, 111 Gb and 55 Gb for...' contains information that already exists in
Table S3 and does not need to be repeated.
Response:
Thank you for the comment and we have removed the descriptions from the manuscript.

Table S2: There is no 10x download link for NA24385, NA24143 or NA24149.
Response:
We have included 10x Genomics data download links for NA24385, NA24143 and NA24149 in Table S2.

NA24385
https://ftp-trace.ncbi.nlm.nih.gov/giab/ftp/data/AshkenazimTrio/HG002_NA24385_son/10Xgenomics_ChromiumGenome/NA24385.fastq
NA24143
https://ftp-trace.ncbi.nlm.nih.gov/giab/ftp/data/AshkenazimTrio/HG004_NA24143_mother/10Xgenomics_ChromiumGenome/NA24143.fa

NA24149
https://ftp-
trace.ncbi.nlm.nih.gov/giab/ftp/data/AshkenazimTrio/HG003_NA24149_father/10Xgenomics_ChromiumGenome/NA24149.fas

Analysis:
Lines 166-169:
The paragraph starting 'We observed, for 10x Genomics, the peak barcode count is around 7...' should be included in the ma
text to explain the differences in the number of fragments per barcode observed when comparing Figure 2 A1 vs. B1.
Response:
Thank you for the suggestions. We have updated the related results in the revised manuscript from line 383 to line 401:

For human genome sequencing, the average NF/P values were approximately 7 for 10x Genomics linked-reads and were decl
to around 2 and 4 for stLFR and TELL-Seq linked-reads, respectively (Figure 2B). For metagenomic sequencing, the average
values were around 14 for 10x Genomics linked-reads, 1 for stLFR and 5 for TELL-Seq linked-reads (Figure 2A). These differe
may be attributed to the barcoding approaches employed. In the case of 10x Genomics, barcoding takes place within water-i
droplets, which require a specialized instrument for droplet generation. The number of DNA fragments present in each drople
influenced by the size of the DNA fragments. The DNA fragments from the human genome are long, resulting in fewer fragme
being included in each droplet. The microbial DNA fragments are relatively shorter, allowing for more fragments to be
accommodated within a droplet. In contrast, neither TELL-Seq nor stLFR utilizes droplets for barcoding. Instead, the barcodin
reactions occur in an open environment and are partitioned using beads alone. Generally, each bead is conjugated with at lea
one unique barcode sequence on its surface and can capture 1-2 DNA fragments. For stLFR and TELL-Seq, the number of DN
fragments is not dependent on the length of the DNA fragments, resulting in a similar number of fragments per barcode for b
human and metagenomic sequencing.

Lines 187-188:
'For FreeBayes and SAMtools, we further removed the SNVs if the total depths were less than 6, the number of reads support
alternative allele was less than 2, and SNV qualities were below 15.'- was this included in the manuscript?
Response:
Thank you for the comments. We have included the descriptions in the Methods section from line 570 to line 574.

For FreeBayes and SAMtools, LRTK further removes the SNVs if the total depths are less than 6, the number of reads support
alternative allele is less than 2, and SNV qualities are below 15. LRTK does not perform quality control for the SNVs called by
inStrain as it does not output SNV quality scores.

'inStrain is the most sensitive tool to detect SNVs.'- Is sensitive the right word to use? Sensitive implies that most of SNVs
detected are true positives. Is that necessarily true for inStrain?
Response:
Sorry for the confusion. We revised the sentence as "inStrain is able to detect many unique microbial SNVs" from line 243 in
revised manuscript.

Lines 192-193:
It seems that MAI implies a somatic copy number change in the maternal and paternal alleles of two haplotypes. Are you also
implying that each sample should be treated as a diploid organism, and any time that only the reference or alternate allele
appears alone in the sample, that it counts as a gain or loss? I'm still not sure why this term (especially with the word 'imbala
is being used when it seems to be a cancer-specific phenomenon, and relative abundance change, or strain frequency change
some other phrase is more applicable for metagenomics.
Response:
Thanks for the comment, we believe "strain frequency change" is more appropriate here. We have replaced "mirrored allelic
imbalance / MAI" to "strain frequency change" in the revised manuscript.

Lines 201-208:
The line 'We observed that there was a consistent decrease in the number of fragments per barcode (N_{F/P}) for the stLFR
TELL-Seq technologies.' should be added to the main text.
Response:
Thanks for the comments. We have added the descriptions in the Discussion section from line 382 to line 383.

Our findings revealed that there was a consistent decrease of NF/P for stLFR and TELL-Seq technologies than those obtained

10x Genomics.

Miscellaneous:

Lines 399-405:
It seems like you're using some kind of unsupervised clustering method to infer strains i.e.: SNVs with allele frequencies that [illegible] seem to be correlated. There are many methods to infer bacterial strains- why weren't any of those used?
Response:
We agree that there are many microbial strain inference tools, such as StrainFinder, STRONG, MetaMaps. However, these too[illegible] not consider the long-range connectedness provided by linked-reads. The challenge of developing a strain inference method tailored to linked-read sequencing is still unsolved. In LRTK, we employed an unsupervised clustering method to group SNVs [illegible] similar allele frequencies. The SNVs from the same cluster were considered to be from the same strain. This strategy has bee[illegible] successfully applied by Roodgar M, et al. on longitudinal 10x Genomics linked-read sequencing dataset [2].

Line 665:
Does the 'B' in BAF (B allele frequency) refer to the alternate and/or minor allele? Please standardize your terminology.
Response:
Thank you for the comment. We have replaced “B allele frequency” with “minor allele frequency” in the revised manuscript.

Reference
1. Murillo GH, You N, Su X, Cui W, Reilly MP, Li M, et al.. MultiGeMS: detection of SNVs from multiple samples using model selection on high-throughput sequencing data. Bioinformatics. Oxford Academic; 2016; doi: 10.1093/BIOINFORMATICS/BTV7[illegible]
2. Roodgar M, Good BH, Garud NR, Martis S, Avula M, Zhou W, et al.. Longitudinal linked-read sequencing reveals ecological [illegible] evolutionary responses of a human gut microbiome during antibiotic treatment. Genome Res. Cold Spring Harbor Laboratory Press; 2021; doi: 10.1101/GR.265058.120/-/DC1.
