# Supplementary material for: LRTK: a platform agnostic toolkit for linked-read analysis of both human genome and metagenome
Source: Gigascience. 2024 Jun 13;13:giae028. doi: 10.1093/gigascience/giae028 (PMC11170215; doi:10.1093/gigascience/giae028)
Supplement: giae028_Reviewer_1_Report_Original_Submission — Brock Peters, Ph.D. -- 10/20/2023 [file giae028_reviewer_1_report_original_submission.pdf]

Reviewer Report

Title: LRTK: A platform agnostic toolkit for linked-read analysis of both human genome and metagenome

Version: Original Submission **Date:** 10/20/2023

Reviewer name: Brock Peters, Ph.D.

Reviewer Comments to Author:

Yang et al. describe a package of tools, LRTK, for cobarcoded reads (linked reads) agnostic of library preparation methods and sequencing platforms. In general, it appears to be a very useful tool. I have a few concerns with the manuscript as it is currently written:1. Line 203 "With Pangaea,LRTK achieves NA50 values of 1.8 Mb and 1.2 Mb for stLFR and TELL-Seq sequencing data, respectively. On 10x Genomics sequencing data, Athena exhibited superior assembly performance, with a NGA50 of 245 Kb."This is a bit of an awkward two sentences as you are comparing NA50 values for stLFR and TELL-Seq and then NGA50 for 10X Genomics and it makes it sound like 10X Genomics performed the best. Also, these numbers don't seem to agree with the figure.2. How long does an average run take to process? Say a 35X human genome coverage sample? Are there requirements for memory? A figure and metrics around this sort of thing would be helpful.3. How much data was used per library? What was the total coverage? Was the data normalized to have the same coverage per library? If not, it's very difficult to make fair comparisons between the different technologies.4. There's a section on reconstruction of long fragments, but then there really isn't any evaluation of this result and it's not clear if these are even used for anything. For all of these sequencing types I would assume that you can't really do much in the way of seed extension since the coverage across long fragments for these methods is much less than 1X. I think this needs to be developed a little more or it needs to be explained how these are used in your process or you just need to say you didn't use them for anything but here's some potential applications they could be used for. What type of file is output from this process? I think it's interesting, but just not clear how to use this data.5. I did try to install the software using Conda, but it failed and it's not clear to me why. Perhaps it's something about my environment, but you might want to have some colleagues located in different institutions try to install it to make sure it is easy to do so.

Level of Interest

Please indicate how interesting you found the manuscript: Choose an item.

Quality of Written English

Please indicate the quality of language in the manuscript: Choose an item.

Declaration of Competing Interests

Please complete a declaration of competing interests, considering the following questions:

- Have you in the past five years received reimbursements, fees, funding, or salary from an organisation that may in any way gain or lose financially from the publication of this manuscript, either now or in the future?
- Do you hold any stocks or shares in an organisation that may in any way gain or lose financially from the publication of this manuscript, either now or in the future?
- Do you hold or are you currently applying for any patents relating to the content of the manuscript?
- Have you received reimbursements, fees, funding, or salary from an organization that holds or has applied for patents relating to the content of the manuscript?
- Do you have any other financial competing interests?
- Do you have any non-financial competing interests in relation to this paper?

If you can answer no to all of the above, write 'I declare that I have no competing interests' below. If your reply is yes to any, please give details below.

I declare that I have no competing interests

I agree to the open peer review policy of the journal. I understand that my name will be included on my report to the authors and, if the manuscript is accepted for publication, my named report including any attachments I upload will be posted on the website along with the authors' responses. I agree for my report to be made available under an Open Access Creative Commons CC-BY license (http://creativecommons.org/licenses/by/4.0/). I understand that any comments which I do not wish to be included in my named report can be included as confidential comments to the editors, which will not be published.

Choose an item.

To further support our reviewers, we have joined with Publons, where you can gain additional credit to further highlight your hard work (see: https://publons.com/journal/530/gigascience). On publication of this paper, your review will be automatically added to Publons, you can then choose whether or not to claim your Publons credit. I understand this statement.

Yes Choose an item.
