# Supplementary material for: LRTK: a platform agnostic toolkit for linked-read analysis of both human genome and metagenome
Source: Gigascience. 2024 Jun 13;13:giae028. doi: 10.1093/gigascience/giae028 (PMC11170215; doi:10.1093/gigascience/giae028)
Supplement: giae028_Reviewer_2_Report_Original_Submission — Lauren Mak, MSc -- 11/12/2023 [file giae028_reviewer_2_report_original_submission.pdf]

Reviewer Report

Title: LRTK: A platform agnostic toolkit for linked-read analysis of both human genome and metagenome

Version: Original Submission **Date:** 11/12/2023

Reviewer name: Lauren Mak, MSc

Reviewer Comments to Author:

Summary: This manuscript describes the need for a generalized linked-read (LR) analysis package and showcases the package the authors developed to address this need. Overall, the workflow is well-designed but there are major gaps in the benchmarking, analysis, and documentation process that need to be addressed before publication.Documentation:The purpose of multiple tool options: While the analysis package is technically sound, one major aspect is left unexplained- why are there so many algorithm options included without guidance as to which one to use? There are clearly performance differences by different algorithms (combinations of 2+ not considered either) on different types of LR sequence.Provenance of ATCC-MSA-1003: Nowhere in the manuscript is the biological and technical composition of the metagenomics control described. It would be helpful to mention that this is specifically a mock gut microbiome sample, as well as the relative abundances of the originating species as well as the absolute amounts of genetic material per species (ex. as measured by genomic coverage) in the actual dataset. As a corollary, there should be standard deviations in any figures that display a summary statistic (ex. Figure 3A- precision, recall, etc.) that seems to be averaged across the species in a sample. This includes Figure 3A and Figure 4A.Dataset details: There is no table indicating the number of reads for each dataset, which would be helpful in interpreting Figures 3 and 4.Open source?: However, there was no Github link provided, only a link to the Conda landing page. Are there thorough instructions provided for the package's installation, input, output, and environment management?Benchmarking:The lack of simulated tests: The above concern (expected performance on idealized datasets) is best addressed with simulated data, which was not done despite the fact that LR-Sim exists (and apparently the authors have written a tool for stLFR as well previously).Indels: What are the sizes of the indels detected? Why were newer tools, such as PopIns2, Pamir, or Novel-X not tried as well?Analysis:Lines 166-169: Figure 1 panel A1 vs. B1- why do the distribution of estimated fragment sizes from the 10x datasets look so different in metagenomic vs. human samplees, when there is reasonable consistency in TELL-Seq and stLFR datasets?Lines 182-184: Figure 3A- why is LRTK's taxonomic classification quality generally lower than the of the tools? At least in terms of recall, it should perform better as mapping reads to reference genomes should have a lower false negative rate than k-mer-based tools. Also, what is the threshold for having detect a taxon? Is it just any number of reads or is there a minimum bound?Lines 187-188: Figure 3B- at least 15% of each caller's set of variants is unique to the variant, while a maximum of 50% is universal. I'd not interpret that as consistency.Lines 192-193: Are you referring to allelic imbalance as it is popularly used to refer to expression variation between the two haplotypes of a diploid organism? This clearly doesn't apply in the case of bacteria. If this is not what you're referring to, please define and/or cite the applicable definition.Lines 201-208: It's

odd that despite the 10x datasets having the largest estimated fragment size, they have some of the smallest genome fractions, NGA50, and NA50. Why is this? Are they just smaller datasets, on average?Miscellaneous:UHGG: Please mention the fact that the UHGG is the default database, as well as whether or not the user will be able to supply their own databases.Line 363: What does `{M}` refer to?Line 369: What does `U` mean here? Is this the number of uniquely aligned reads in one of the windows N that a multi-aligned read aligns to?Lines 371-372: What does 'n% most closely covered windows' refer to?Lines 399-405: How are SNVs chosen for MAI analysis from the three available SNV callers?Lines 653-656: Which dataset was used for quality evaluation?Line 665: What do the abbreviations BAF and T stand for?

Level of Interest

Please indicate how interesting you found the manuscript: Choose an item.

Quality of Written English

Please indicate the quality of language in the manuscript: Choose an item.

Declaration of Competing Interests

Please complete a declaration of competing interests, considering the following questions:

- Have you in the past five years received reimbursements, fees, funding, or salary from an organisation that may in any way gain or lose financially from the publication of this manuscript, either now or in the future?
- Do you hold any stocks or shares in an organisation that may in any way gain or lose financially from the publication of this manuscript, either now or in the future?
- Do you hold or are you currently applying for any patents relating to the content of the manuscript?
- Have you received reimbursements, fees, funding, or salary from an organization that holds or has applied for patents relating to the content of the manuscript?
- Do you have any other financial competing interests?
- Do you have any non-financial competing interests in relation to this paper?

If you can answer no to all of the above, write 'I declare that I have no competing interests' below. If your reply is yes to any, please give details below.

I declare that I have no competing interests' below.

I agree to the open peer review policy of the journal. I understand that my name will be included on my report to the authors and, if the manuscript is accepted for publication, my named report including any attachments I upload will be posted on the website along with the authors' responses. I agree for my

report to be made available under an Open Access Creative Commons CC-BY license (http://creativecommons.org/licenses/by/4.0/). I understand that any comments which I do not wish to be included in my named report can be included as confidential comments to the editors, which will not be published.

Choose an item.

To further support our reviewers, we have joined with Publons, where you can gain additional credit to further highlight your hard work (see: https://publons.com/journal/530/gigascience). On publication of this paper, your review will be automatically added to Publons, you can then choose whether or not to claim your Publons credit. I understand this statement.

Yes Choose an item.
