# Supplementary material for: LRTK: a platform agnostic toolkit for linked-read analysis of both human genome and metagenome
Source: Gigascience. 2024 Jun 13;13:giae028. doi: 10.1093/gigascience/giae028 (PMC11170215; doi:10.1093/gigascience/giae028)
Supplement: giae028_Reviewer_2_Report_Revision_1 — Lauren Mak, MSc -- 3/29/2024 [file giae028_reviewer_2_report_revision_1.pdf]

Reviewer Report

Title: LRTK: A platform agnostic toolkit for linked-read analysis of both human genome and metagenome

Version: Revision 1 **Date:** 3/29/2024

Reviewer name: Lauren Mak, MSc

Reviewer Comments to Author:

The authors have responded to all of the referees' comments and suggestions thoroughly. I have a few remaining suggestions and questions.Documentation:The purpose of multiple tool options:By 'Its straightforward, easy to use and timesaving.', do you mean that FreeBayes is more computationally efficient than GATK?Provenance of ATCC-MSA-1003:The sentence 'The sequencing data volumes are 37.7 Gb, 111 Gb and 55 Gb for...' contains information that already exists in Table S3 and does not need to be repeated.Table S2: There is no 10x download link for NA24385, NA24143 or NA24149.Analysis:Lines 166-169:The paragraph starting 'We observed, for 10x Genomics, the peak barcode count is around 7...' should be included in the main text to explain the differences in the number of fragments per barcode observed when comparing Figure 2 A1 vs. B1.Lines 187-188:'For FreeBayes and SAMtools, we further removed the SNVs if the total depths were less than 6, the number of reads supporting alternative allele was less than 2, and SNV qualities were below 15.'- was this included in the manuscript?'inStrain is the most sensitive tool to detect microbial SNVs.'- Is sensitive the right word to use? Sensitive implies that most of SNVs detected are true positives. Is that necessarily true for inStrain?Lines 192-193:It seems that MAI implies a somatic copy number change in the maternal and paternal alleles of two haplotypes. Are you also implying that each sample should be treated as a diploid organism, and any time that only the reference or alternate allele appears alone in the sample, that it counts as a gain or loss? I'm still not sure why this term (especially with the word 'imbalance') is being used when it seems to be a cancer-specific phenomenon, and relative abundance change, or strain frequency change, or some other phrase is more applicable for metagenomics.Lines 201-208:The line 'We observed that there was a consistent decrease in the number of fragments per barcode (N_{F/P}) for the stLFR and TELL-Seq technologies.' should be added to the main text.Miscellaneous:Lines 399-405:It seems like you're using some kind of unsupervised clustering method to infer strains i.e.: SNVs with allele frequencies that seem to be correlated. There are many methods to infer bacterial strains- why weren't any of those used?Line 665:Does the 'B' in BAF (B allele frequency) refer to the alternate and/or minor allele? Please standardize your terminology.

Level of Interest

Please indicate how interesting you found the manuscript: Choose an item.

Quality of Written English

Please indicate the quality of language in the manuscript: Choose an item.

Declaration of Competing Interests

Please complete a declaration of competing interests, considering the following questions:

- Have you in the past five years received reimbursements, fees, funding, or salary from an organisation that may in any way gain or lose financially from the publication of this manuscript, either now or in the future?
- Do you hold any stocks or shares in an organisation that may in any way gain or lose financially from the publication of this manuscript, either now or in the future?
- Do you hold or are you currently applying for any patents relating to the content of the manuscript?
- Have you received reimbursements, fees, funding, or salary from an organization that holds or has applied for patents relating to the content of the manuscript?
- Do you have any other financial competing interests?
- Do you have any non-financial competing interests in relation to this paper?

If you can answer no to all of the above, write 'I declare that I have no competing interests' below. If your reply is yes to any, please give details below.

I declare that I have no competing interests

I agree to the open peer review policy of the journal. I understand that my name will be included on my report to the authors and, if the manuscript is accepted for publication, my named report including any attachments I upload will be posted on the website along with the authors' responses. I agree for my report to be made available under an Open Access Creative Commons CC-BY license (http://creativecommons.org/licenses/by/4.0/). I understand that any comments which I do not wish to be included in my named report can be included as confidential comments to the editors, which will not be published.

Choose an item.

To further support our reviewers, we have joined with Publons, where you can gain additional credit to further highlight your hard work (see: https://publons.com/journal/530/gigascience). On publication of this paper, your review will be automatically added to Publons, you can then choose whether or not to claim your Publons credit. I understand this statement.

Yes Choose an item.
