# Supplementary material for: LRTK: a platform agnostic toolkit for linked-read analysis of both human genome and metagenome
Source: Gigascience. 2024 Jun 13;13:giae028. doi: 10.1093/gigascience/giae028 (PMC11170215; doi:10.1093/gigascience/giae028)
Supplement: giae028_Reviewer_3_Report_Original_Submission — Dmitrii Meleshko -- 11/13/2023 [file giae028_reviewer_3_report_original_submission.pdf]

Reviewer Report

Title: LRTK: A platform agnostic toolkit for linked-read analysis of both human genome and metagenome

Version: Original Submission **Date:** 11/13/2023

Reviewer name: Dmitrii Meleshko

Reviewer Comments to Author:

The paper titled "LRTK: A Platform-Agnostic Toolkit for Linked-Read Analysis of Both Human Genomes and Metagenomes" by Yang et al. is dedicated to the development of a unified interface for linked-read data processing.The problem described in the paper indeed exists; each linked-read technology requires complex preprocessing steps that are not straightforward or efficient. The idea of consolidating multiple tools in one place, with some of them modified to handle multiple data types, is commendable. Overall, I am supportive of this paper. My main concern, however, is that the impact of linked-read applications in the paper appears to be exaggerated, and the authors need to provide more context in their presentation. Also, some parts of the paper are vague described. I will elaborate on my concerns in more detail below.X) Linked-read sequencing generates reads with high base quality and extrapolative 64 information on long-range DNA connectedness, which has led to significant 65 advancements in human genome and metagenome research[1-3]. - Citations 1-3 do not really tell about advancements in human genome and metagenome research, these are technologies papers. Similar problem can be found in "Despite the limitations that genome specificity…" paragraph. Authors cited and described several algorithms, that are not really genomic studies. E.g. "stLFR[2] has found application in a customized pipeline that has been developed to first convert its raw reads into a 10x-compatible format, after which Long Ranger is applied for downstream analysis." is not an example of genomic study, but a pipeline description.X) Table S1 does not improve the paper, I would say it does completely the opposite. LongRanger is not a toolkit, it should be considered as read alignment tool that outputs some SVs and haplotypes along the way. So LongRanger vs LRTK comparison does not make sense to me. There are other tools that solve metagenome assembly problem, human assembly problem, call certain classes of SVs etc.x) I think incorporating longranger is important, since its performance is reported to be better than EMA for human samples and it is also more popular than EMA. Is it possible and have you tried doing it?x) I would remove exaggerations such as "myriad" from the text. The scope of linked-reads is pretty limited nowadays. I agree that linked-reads might be useful in metagenomics/transcriptomics and other scenarios that were mentionedin the text, but the number of studies is very limited especially nowadays, and was not really big when 10X platform was on the risex) "LRTK reconstructs long DNA fragments" - when people talk about long fragment reconstruction, they usually mean moleculo-style reconstruction through assembly. This reconstruction resemble "barcode deconvolution", described in Danko et al, and Mak et al. So I would stick to this terminologyx) it is important to note that, Aquila, LinkedSV and VALOR2 are linked-read specific tools, while FreeBayes, Samtools and GATK are short-read tools. Also, provide target SV length for both groups of tools.x) There are some minor problems with Github readme. E.g. "*parameters". Also, I don't understand how to use conversion in real life… E.g. 10X

Genomics data often comes as a folder with multiple gzipped R1/R2/I1 files. I don't understand how would I use it in that case.x) Please cite or explain why this is happening (not only when) - "A known concern with stLFR linked-read sequencing is the loss of barcode specificity during analysis."x) I don't understand what is "Length-weighted average (Î¼FL) and unweighted average (WÎ¼FL) of DNA 688 fragment lengths." from the figure. One of them is just an average and what about second? Figure looks confusingx) LRTK supports reconstruction of long DNA fragments - this section describes something else. More about statistics and data QCx) LRTK promotes metagenome assembly using barcode specificity - please remove supernova, it was never a metagenomic assembler. Check cloudSPAdes insteadx) "The superior assembly performance we have observed" - superior compared to what? If so, some short-read benchmark should be included.x) "LRTK improves human genome variant phasing using long range information" - What dataset is this? What callset was used for ground truth? Briefly describe how comparisons were done?x) Figures 5F-G together are very confusing.First I don't expect tools like LinkedSV to have high recall (around 1.0) and low precision. Also, figure G is kind of subset of figure F, but results are completely different. Also use explicit notation. E.g. 50-1kbp and 1-10kbp mean completely different thingsx) We curated one benchmarking dataset and two real datasets to demonstrate the 307 performance of LRTK - what do you mean by "curation" herex) Why don't you use Tell-Seq barcode whitelist mentioned here - https://sagescience.com/wp-content/uploads/2020/10/TELL-Seq-Software-Roadmap-User-Guide-2.pdfx) Tiered alignment approach is vaguely introduced. It is not clear what "n% most closely covered windows." mean, or how do we select a subset of reference genomes for the second phase

Level of Interest

Please indicate how interesting you found the manuscript: Choose an item.

Quality of Written English

Please indicate the quality of language in the manuscript: Choose an item.

Declaration of Competing Interests

Please complete a declaration of competing interests, considering the following questions:

- Have you in the past five years received reimbursements, fees, funding, or salary from an organisation that may in any way gain or lose financially from the publication of this manuscript, either now or in the future?
- Do you hold any stocks or shares in an organisation that may in any way gain or lose financially from the publication of this manuscript, either now or in the future?
- Do you hold or are you currently applying for any patents relating to the content of the manuscript?
- Have you received reimbursements, fees, funding, or salary from an organization that holds or has applied for patents relating to the content of the manuscript?

- Do you have any other financial competing interests?
- Do you have any non-financial competing interests in relation to this paper?

If you can answer no to all of the above, write 'I declare that I have no competing interests' below. If your reply is yes to any, please give details below.

I declare that I have no competing interests

I agree to the open peer review policy of the journal. I understand that my name will be included on my report to the authors and, if the manuscript is accepted for publication, my named report including any attachments I upload will be posted on the website along with the authors' responses. I agree for my report to be made available under an Open Access Creative Commons CC-BY license (http://creativecommons.org/licenses/by/4.0/). I understand that any comments which I do not wish to be included in my named report can be included as confidential comments to the editors, which will not be published.

Choose an item.

To further support our reviewers, we have joined with Publons, where you can gain additional credit to further highlight your hard work (see: https://publons.com/journal/530/gigascience). On publication of this paper, your review will be automatically added to Publons, you can then choose whether or not to claim your Publons credit. I understand this statement.

Yes Choose an item.
